# Enantioselective difunctionalization of alkenes by a palladium-catalyzed Heck/borylation sequence†

**DOI:** 10.1039/d1sc06229h

**Published:** 2022-01-22

**Authors:** Yuanqi Wu, Lizuo Wu, Zhan-Ming Zhang, Bing Xu, Yu Liu, Junliang Zhang

**Affiliations:** Jilin Provincial Key Laboratory of Carbon Fiber Development and Application, College of Chemistry and Life Science, Advanced Institute of Materials Science, Changchun University of Technology Changchun 130012 P. R. China yuliu@ccut.edu.cn; Department of Chemistry, Fudan University 2005 Songhu Road Shanghai 200438 P. R. China zhanmingzhang@fudan.edu.cn junliangzhang@fudan.edu.cn

## Abstract

A palladium catalyzed enantioselective Heck/borylation reaction of alkene-tethered aryl iodides was realized, delivering a variety of 2,3-dihydrobenzofuranyl boronic esters in high yield with excellent enantioselectivity. Asymmetric synthesis of chromane boronic ester, indane boronic ester and indoline boronic ester was also accomplished. The protocol offers an efficient access to the corresponding chiral benzocyclic boronic esters, which are notably important chemical motifs in synthetic transformations.

## Abstract 2

A palladium catalyzed enantioselective Heck/borylation reaction of alkene-tethered aryl iodides was realized, delivering a variety of 2,3-dihydrobenzofuranyl boronic esters in high yield with excellent enantioselectivity.

## Graphical abstract

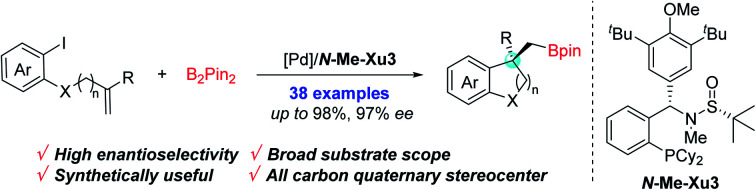

## Introduction

**Scheme 1:**
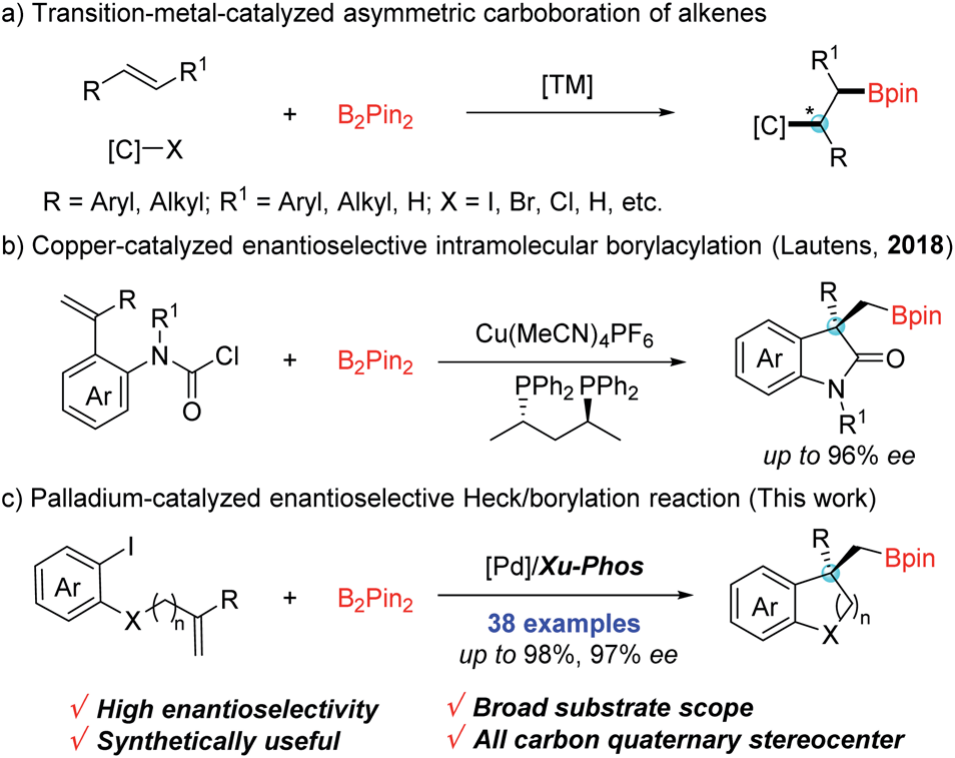
Transition metal catalyzed asymmetric carboboration of alkenes.

Versatile transformations of carbon–boron (C–B) bonds have been recognized as an ideal platform for the preparation of value-added molecules.^1^ Much effort has been devoted to developing efficient methods for the construction of C–B bonds,^2^ in which, transition-metal-catalyzed asymmetric carboboration of alkenes represents one of the most step- and atom-economic tools for facile access to enantioenriched borylated compounds.^3^ Over the past few years, asymmetric carboboration of alkenes has been elegantly achieved by using dual metal synergistic or single metal catalysis, such as Cu/Pd-,^4^ Pd-,^5^ Cu-^6^ or Ni^7^-catalysis (Scheme 1a). Despite the breakthroughs in this process, its application in the construction of chiral boron-containing benzocycles is underdeveloped.

Benzocyclic skeletons are important building blocks prevalently found in biologic and pharmaceutical molecules.^8^ Owing to organoboron compounds' high propensity of functionalization, the introduction of boron into benzocyclic skeletons can provide a promising avenue to expediently access valuable functionalized molecules bearing benzocyclic skeletons by downstream synthetic manipulation. In 2018, an enantioselective intermolecular Cu-catalyzed borylacylation to access chiral borylated 3,3-disubstituted oxindoles was remarkably demonstrated by Lautens *et al.* (Scheme 1b).^9^ As another appealing alternative, the palladium-catalyzed domino Heck/borylation process which involves carbopalladation of a double bond and boron trapping of σ-alkylpalladium species, has provided an elegant strategy to form borylated compounds bearing benzocyclic skeletons.^10^ Despite major progress in the racemic Heck/borylation sequence, the exploration of its asymmetric variants to efficiently furnish chiral boron-containing benzocyclic compounds has remained an unresolved issue. Recently, Hooper's group realized the synthesis of borylated indanes in moderate enantioselectivity through the palladium-catalysed carboborylation reaction.^11^ Tong *et al.* developed a Pd-catalyzed asymmetric vinylborylation of (*Z*)-1-iodo-dienes with B_2_Pin_2_ for enantioselective construction of 3,3-disubstituted tetrahydropyridines.^12^ On the basis of these findings and the studies toward asymmetric cascade Heck reactions in our group,^13^ we tended to exploit a highly enantioselective Heck/borylation sequence for the construction of the C–B bond starting from alkene-tethered aryl iodides and boronic esters. However several issues make the protocol challenging: (1) a direct Miyaura-type borylation seems to have an edge;^14^ (2) competitive side reactions such as the reductive Heck reaction and carboiodination should be avoided;^13*a*,*b*^ (3) the transmetalation step may take place prior to the alkene insertion, which could alter the steric environment and affect the enantioselectivity. Herein, we report a highly enantioselective Pd-catalyzed tandem Heck/borylation sequence, conveniently accessing various chiral benzocyclic boronic esters in good yields (Scheme 1c).

## Results and discussion

**Fig. 1:**
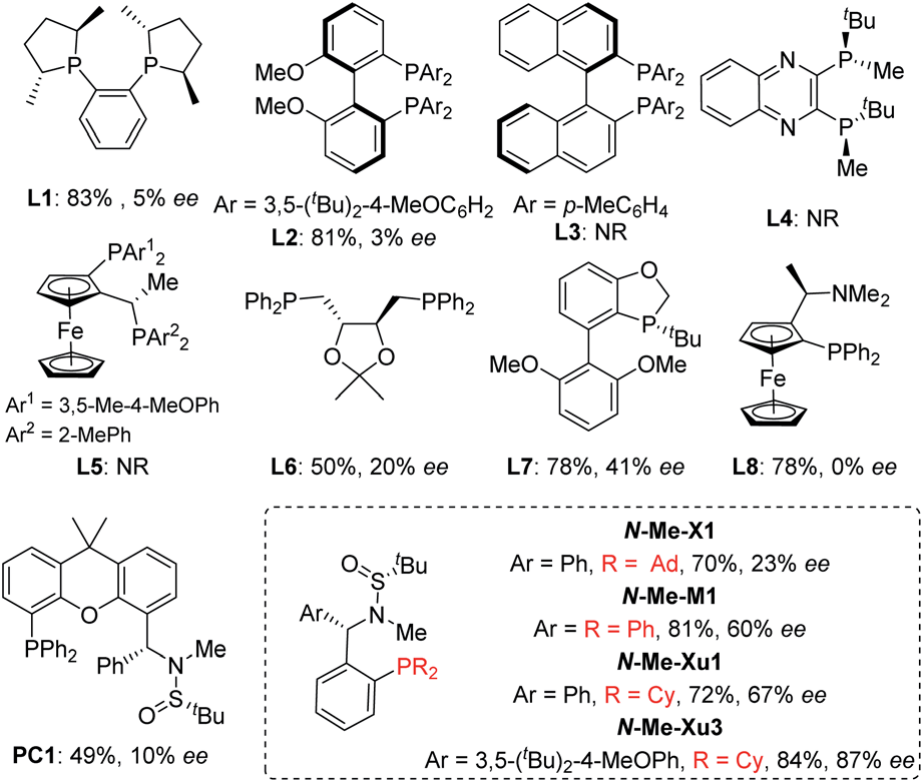
Screening of the ligands.

With *ortho*-iodophenol-derived allyl ether 1a and B_2_Pin_2_ as model substrates, commercially available chiral ligands were initially investigated in this tandem Heck/borylation system with Pd_2_(dba)_3_·CHCl_3_ as the precatalyst, and Cs_2_CO_3_ as the base in MTBE (Fig. 1). Enantioselectivities were nearly suppressed with (*R*,*R*)-Me-Duphos (L1) and (*R*)-DTBM-BIPHEP (L2) used as chiral ligands. The reactions were completely suppressed with (*R*)-XylBINAP (L3) (*R*,*R*)-QuinoxP* (L4) and Josiphos (L5). (*S*,*S*)-DIOP (L6) and (*R*)-BIDIME (L7) gave slightly better results, delivering 2a with 20% and 41% enantioselectivity, respectively. Racemic product 2a was obtained when (*R*)-(*S*)-PPFA (L8) was used. Over the past few years, we have been focusing on the development of chiral sulfonamide phosphine ligands (Sadphos) for transition-metal-catalyzed asymmetric reactions.^13,15^ Consequently, we examined the performance of a series of Sadphos (PC-Phos, Xiang-Phos, Ming-Phos and Xu-Phos) in this asymmetric reaction. The reaction using Ming-Phos (*N*-Me-M1) as the chiral ligand could provide the desired product in 81% yield with 60% *ee*, whereas the use of PC-Phos and Xiang-Phos still failed to improve the enantioselectivity. When the Xu-Phos ligand (*N*-Me-Xu1) bearing the dicyclohexyl phosphine moiety was used, the enantioselectivity of 2a was further increased to 67%. To our delight, *N*-Me-Xu3 with a bulky 3,5-^*t*^Bu_2_-4-OMe-C_6_H_2_ group showed significant efficacy, delivering 2a in 84% yield with 87% *ee*.

Further screening of other reaction factors with (*S*,*R*_S_)-*N*-Me-Xu3 as the chiral ligand showed a subtle effect of solvents. Inferior results were obtained when the reaction was conducted in THF (Table 1, entry 2). However, both the reactivity and enantioselectivity were obviously promoted using Et_2_O as the solvent (Table 1, entry 3). Both toluene and chlorinated solvents failed to provide better results (Table 1, entries 4–6). Bases turned out to exert a profound influence on the reaction outcomes, and Cs_2_CO_3_ was still the best choice (Table 1, entries 7–10). The choice of the palladium source seemed not pivotal to the system, since relatively similar results were obtained when other palladium salts such as Pd(OAc)_2_, [PdCl(ally)]_2_ and Pd(dba)_2_ were employed (Table 1, entries 11–13). Noteworthy, the *ee* value was further increased to 91% when H_2_O was added (Table 1, entry 14). Finally, the loading of the catalyst and the reaction temperature could be lowered down without compromising the yield and the enantioselectivity (Table 1, entry 15).

**Table 1:** Optimization of the reaction conditions^a^

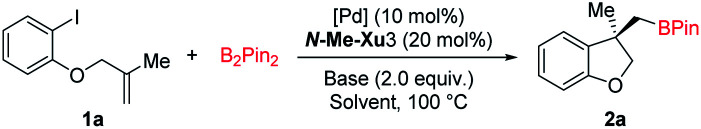				
Entry	[Pd]	Solvent	Base	Yield(*Ee*) [%]^b^^,^^c^
1	Pd_2_(dba)_3_·CHCl_3_	MTBE	Cs_2_CO_3_	84(87)
2	Pd_2_(dba)_3_·CHCl_3_	THF	Cs_2_CO_3_	83(81)
3	Pd_2_(dba)_3_·CHCl_3_	Et_2_O	Cs_2_CO_3_	98(89)
4	Pd_2_(dba)_3_·CHCl_3_	PhMe	Cs_2_CO_3_	66(81)
5	Pd_2_(dba)_3_·CHCl_3_	DCM	Cs_2_CO_3_	80(87)
6	Pd_2_(dba)_3_·CHCl_3_	DCE	Cs_2_CO_3_	60(82)
7	Pd_2_(dba)_3_·CHCl_3_	Et_2_O	KOH	73(89)
8	Pd_2_(dba)_3_·CHCl_3_	Et_2_O	^*t*^BuONa	38(62)
9	Pd_2_(dba)_3_·CHCl_3_	Et_2_O	K_3_PO_4_	85(89)
10	Pd_2_(dba)_3_·CHCl_3_	Et_2_O	K_2_CO_3_	33(60)
11	Pd(OAc)_2_	Et_2_O	Cs_2_CO_3_	95(88)
12	[PdCl(allyl)]_2_	Et_2_O	Cs_2_CO_3_	97(88)
13	Pd(dba)_2_	Et_2_O	Cs_2_CO_3_	90(89)
14^d^	Pd_2_(dba)_3_·CHCl_3_	Et_2_O	Cs_2_CO_3_	97(91)
15^d^^,^^e^^,^^f^	Pd_2_(dba)_3_·CHCl_3_	Et_2_O	Cs_2_CO_3_	98(91)

a Reaction conditions: 1a (0.1 mmol), B_2_Pin_2_ (1.1 equiv), [Pd] (5 mol%), *N*-Me-Xu3 (10 mol%), Cs_2_CO_3_ (2.0 equiv), 1 mL of solvent under a N_2_ atmosphere at 100 °C for 12 h.

b Determined by ^1^H NMR analysis with CH_2_Br_2_ as an internal standard.

c The *ee* value of 2a was determined by HPLC analysis.

d H_2_O (4.0 equiv.) was added.

e 2.5 mol% of Pd_2_(dba)_3_·CHCl_3_, and 7.5 mol% of *N*-Me-Xu3 was used.

f 80 °C.

**Scheme 2:**
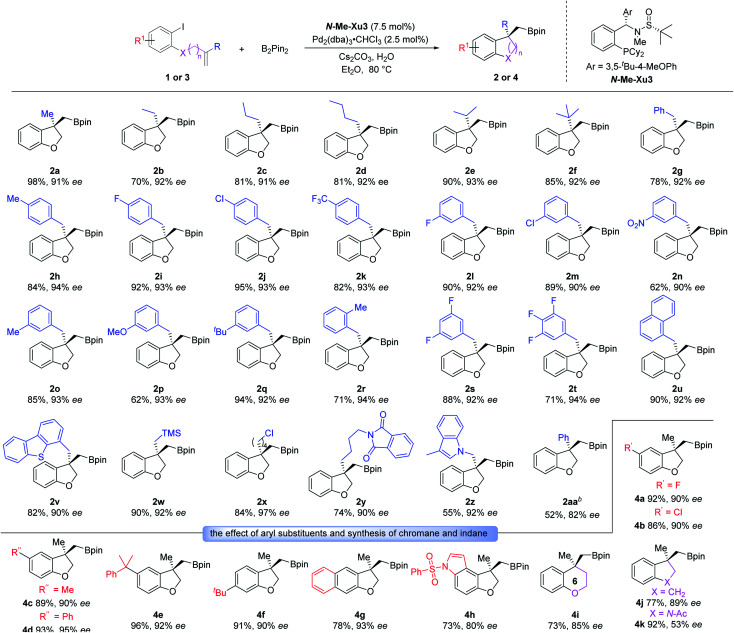
Exploration of the substrate scope.

Having optimized the enantioselective tandem Heck/borylation protocol, we evaluated the compatability and stereochemical fidelity. Excitingly, the optimized reaction conditions were generally compatible with various substituents having different electronic properties on the allyl moiety (Scheme 2). Besides the methyl substrate 1a, alkenes with other linear alkyl groups including Et, ^*n*^Pr and ^*n*^Bu all worked well to furnish the desired products 2b–2d. Substrates equipped with sterically hindered branched groups such as ^*i*^Pr and ^*t*^Bu substituents all worked smoothly under the standard conditions, offering products 2e and 2f with high yields and *ee* values, suggesting the insensitivity of the system to the steric effect. Moreover, benzyl allyl ethers bearing diverse functional groups including methyl, halogens (F and Cl) and CF_3_ substituents at the *para*-position of the phenyl ring underwent this tandem Heck/Borylation smoothly, delivering the corresponding products 2g–2k in good yields with 92–94% *ees*. Substituents at the *meta*- and *ortho*-positions of the phenyl ring were also tolerated, and the desired products 2l–2r were formed in good yields with 90–94% *ees*. Besides, boronic esters 2s and 2t with di- or tri-fluorine-substituted phenyl rings were prepared in 71–88% yields with 92–94% *ees*. Moreover, naphthyl and dibenzothienyl ancillaries reacted smoothly, delivering the corresponding compounds 2u and 2v in good yields with excellent *ee* values. Benzofuranyl boronic esters with the TMS moiety could also be achieved uneventfully (2w). The chlorinated alkyl group was also accommodated without any side reaction (2x). Notably, the reactions of substrates derived from phthalimide (1y) and indole (1z) worked equally well. Besides, the 2-phenylallyl substrate (1aa) could also deliver the desired product in moderate yield and with good enantioselectivity.

Furthermore, we investigated the substrate scope with respect to various 2-iodophenol derivatives. As shown in Scheme 2, halogens (F and Cl), methyl, phenyl and cumyl located at the C5 position of 2,3-dihydrobenzofuranyl boronic esters (4a–4e) were obtained in good yields with excellent enantioselectivities. The desired product 4f with ^*t*^Bu substituted at the C6 position could be smoothly generated (91% yield and 90% *ee*). The 2-naphthol- and indol-5-ol-derived allyl ethers 3g and 3h also worked well, leading to 4g and 4h with 93% and 80% *ee*, respectively. Next, we tried to assemble a six-membered product employing *o*-iodophenol-derived homoallyl ether 3i. Gratifyingly, the desired chromane 4i was generated with good yield and enantioselectivity. Further attempt to produce the indane product was found to be completely successful. Specifically, the substrate with a carbon linker (3j) showed excellent reactivity, providing 4j with the satisfactory result. The generality of this system was also demonstrated by the tolerance of the substrate with AcN as a tether, and the indoline adduct 4k was produced in 92% yield with 53% *ee*.

**Scheme 3:**
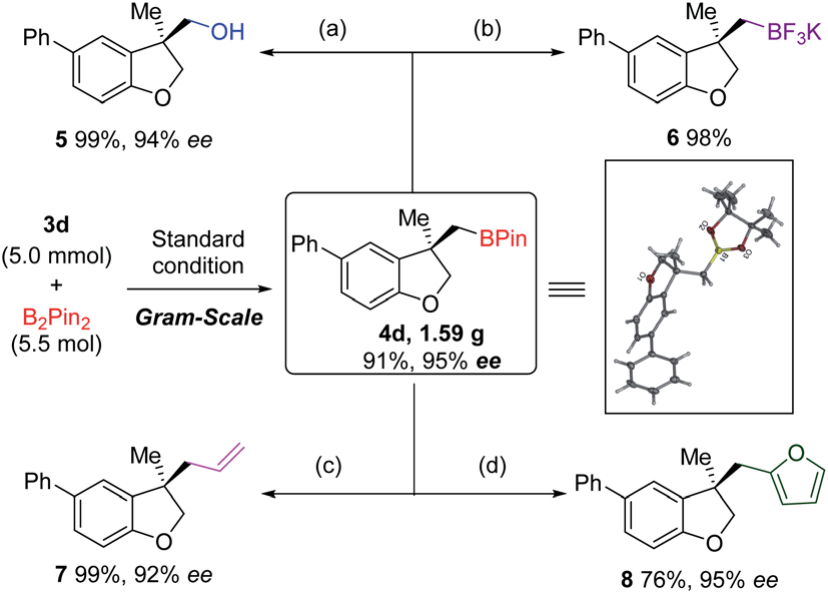
Synthetic transformations of 4d.

A gram-scale reaction was conducted to verify the practicability of our methodology. Starting from 5.0 mmol of allyl ether 3d, dihydrobenzofuran 4d was obtained in 91% yield and with 95% *ee* with half-loading of the Pd-catalyst and (*S*,*R*_S_)-*N*-Me-Xu3 (Scheme 3). The structure of 4d was confirmed by single crystal X-ray diffraction, and its absolute configuration was unambiguously determined. The versatility of the borylated 3,3-disubstituted 2,3-dihydrobenzofuran scaffold was demonstrated by carbon–boron bond construction. Oxidation with hydrogen peroxide and sodium phosphate monobasic afforded alcohol 5 in 99% yield with 94% *ee* (Scheme 3a). Treatment with KHF_2_ converted 4d into the corresponding organotrifluoroborate 6 in 98% yield (Scheme 3b). Allyl and furyl groups were successfully introduced by stereospecific coupling of the boronic ester 4d with vinyl magnesium bromide and lithiated furan (Scheme 3c and d).

## Conclusions

In summary, we have developed a palladium-catalyzed enantioselective 1,2-carboboration of alkenes by a tandem Heck/borylation approach *via* trapping of the *σ*-alkylpalladium intermediate with a boron reagent. The methodology performs well over a broad scope of substrates, providing facile access to a series of 2,3-dihydrobenzofuran, chromane, indane and indoline in high yields with excellent enantioselectivities. The synthetic potential of our protocol was illustrated by further functionalization *via* transformations of the boron moiety to generate a library of compounds that could serve as medicinally relevant building blocks.

## Data availability

Full experimental and characterisation data are provided as part of the ESI.†

## Author contributions

Y. W. carried out most of the experiments and wrote the initial manuscript draft. L. W. screened the initial reaction conditions and supported the synthesis of substrates. B. X. performed part of the experiments. Z. Z. conceived the project. Z. Z., Y. L. and J. Z. directed the project and finalized the manuscript. All the authors co-wrote the paper. All authors discussed the results and commented on the manuscript.

## Conflicts of interest

The authors declare no competing financial interests.

## Supplementary Material

SC-013-D1SC06229H-s001

SC-013-D1SC06229H-s002
