# Supplementary material for: Enantioselective difunctionalization of alkenes by a palladium-catalyzed Heck/borylation sequence
Source: Chem Sci. 2022 Jan 22;13(7):2021–5. doi: 10.1039/d1sc06229h (PMC8848999; doi:10.1039/d1sc06229h)
Supplement: SC-013-D1SC06229H-s001 [file SC-013-D1SC06229H-s001.pdf]

Electronic Supplementary Material (ESI) for Chemical Science.
This journal is © The Royal Society of Chemistry 2022

*Supporting Information*

# Enantioselective Difunctionalization of Alkenes by a Palladium-Catalyzed Heck/Borylation Sequence

Yuanqi Wu,[a] Lizuo Wu,[a] Zhanming Zhang*,[b] Bing Xu,[b] Yu Liu*,[a] Junliang Zhang*[b]

[a]Jilin Provincial Key Laboratory of Carbon Fiber Development and Application, College of Chemistry and Life Science, Advanced Institute of Materials Science, Changchun University of Technology, Changchun 130012, P.R. China;

[b]Department of Chemistry, Fudan University, 2005 Songhu Road, Shanghai, 200438, P.R. China

## Contents

1. General Information........................................................................................................2
2. Table S1. Screening of the ligands .........................................................................................2
3. Table S2. Screening of the Amount of $H_2O$ for Heck/Borylation Sequence.........................3
4. Typical procedure for Palladium-Catalyzed Enantioselective Heck/Borylation Sequence...3
5. Synthetic Transformations..............................................................................................21
6. X-ray structure of **4d**.............................................................................................23
7. $^1$H , $^{19}$F, $^{13}$C NMR and HPLC Spectra................................................................23
8. References......................................................................................................................110

# 1. General Information

All reactions were carried out under an atmosphere of nitrogen in flame-dried glassware with magnetic stirring. $^{1}H$ NMR spectra, $^{19}F$ NMR spectra, $^{13}C$ NMR spectra were recorded on a Bruker 300, 400 and 500 MHz spectrometer in $CDCl_3$ or acetone-*d6*. All signals are reported in ppm with the internal TMS signal at 0 ppm as a standard. Data for $^{1}H$ NMR spectra are reported as follows: chemical shift (ppm, referenced to TMS; s = singlet, d = doublet, t = triplet, q = quarte, dd = doublet of doublets, dt = doublet of triplets, m = multiplet), coupling constant (Hz), and integration. Data for $^{13}C$ NMR are reported in terms of chemical shift (ppm) relative to residual solvent peak ($CDCl_3$: 77.0 ppm). Reactions were monitored by thin layer chromatography (TLC) using silica gel plates. Flash column chromatography was performed over silica gel (300-400 mesh). The ligands (*R*, *Rs*)-***N*-Me-PC1**,[1] **Wei-Phos**,[2] (*S*, *Rs*)-**X1**, (*S*, *Rs*)-***N*-Me-X1**,[3] (*R*, *Rs*)-**M1**, (*R*, *Rs*)-***N*-Me-M1**, (*S*, *Rs*)-**M1**, (*S*, *Rs*)-***N*-Me-M1**,[4] (*R*, *Rs*)-**Xu1**, (*R*, *Rs*)-***N*-Me-Xu1**, (*S*, *Rs*)-**Xu1**, (*S*, *Rs*)-***N*-Me-Xu1**, (*S*, *Rs*)-***N*-Me- Xu3**[5] and substrates **1a-1aa**, **3a-3g**, **3i**, **3j**[6] ,**3h**[7], **3k**[8], were synthesized according to published procedures. The spectral data of the substrates were consisted with that reported in the literature. The enantionmeric excesses of the products were determined by chiral stationary phase HPLC using a Chiralpak AD-3, IC, AD-H, OJH.

# 2. Table S1. Screening of the ligands

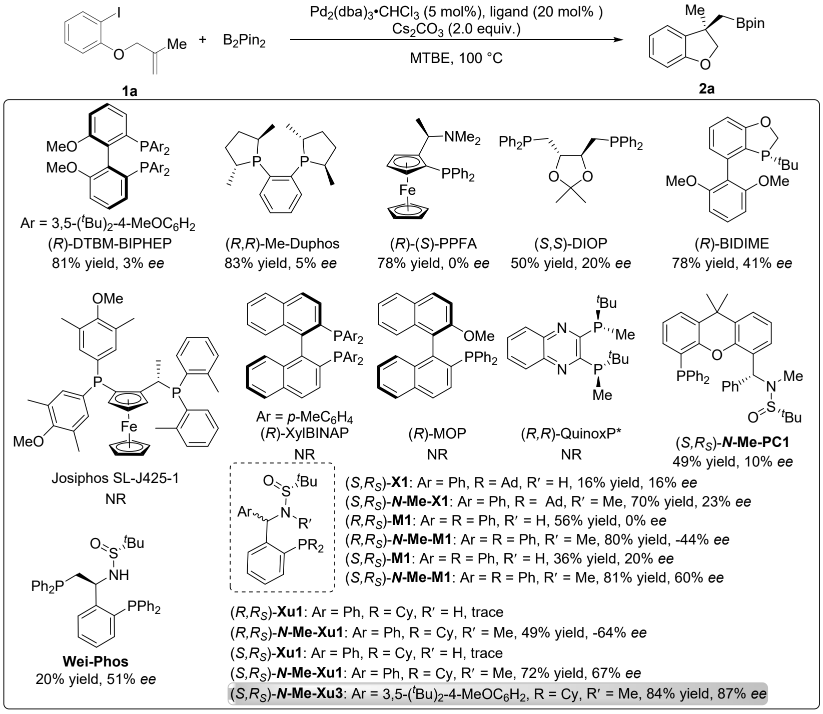


[a]Reaction conditions: **1a** (0.1 mmol), $B_2Pin_2$ (1.1 equiv.), $Pd_2(dba)_3$•$CHCl_3$ (5 mol%), Ligand (20 mol%), $Cs_2CO_3$ (2.0 equiv.), 1 mL of solvent under $N_2$ atmosphere at 100 °C for 12 h. [b]Determined by $^{1}H$ NMR analysis with $CH_2Br_2$ as an internal standard. [c]The *ee* value of **2a** was determined by HPLC analysis.

## 3. Table S2. Screening of the Amount of $H_2O$ for Heck/Borylation Sequence

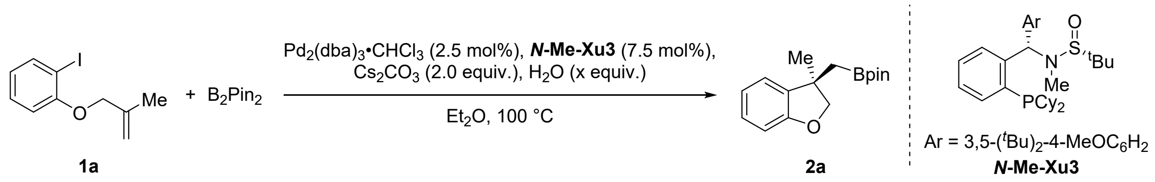


| Entry | $H_2O$ (x) | Yield (%)[b] | *ee* (%)[c] |
|---|---|---|---|
| 1 | 1.0 | 97 | 88 |
| 2 | 2.0 | 98 | 89 |
| 3 | 3.0 | 97 | 90 |
| 4 | 4.0 | 97 | 91 |
| 5 | 5.0 | 95 | 91 |
| 6 | 6.0 | 96 | 90 |

[a]Reaction conditions: **1a** (0.1 mmol), $B_2Pin_2$ (1.1 equiv), $Pd_2(dba)_3{\cdot}CHCl_3$ (2.5 mol%), ***N*-Me-Xu3** (10 mol%), $Cs_2CO_3$ (2.0 equiv), 1 mL of $Et_2O$ under $N_2$ atmosphere at 100 °C for 12h. [b]Determined by $^1$H NMR analysis with $CH_2Br_2$ as the internal standard. [c]Determined by HPLC analysis.

## 4. Typical procedure for Palladium-Catalyzed Enantioselective Heck/Borylation Sequence.

To a sealed tube was added $Pd_2(dba)_3$•$CHCl_3$ (2.5 mol%), ***N*-Me-Xu3** (7.5 mol%), $B_2Pin_2$ (0.33 mmol) and $Cs_2CO_3$ (0.6 mmol). The flask was evacuated and refilled with argon. Then *o*-iodophenol-derived allyl ether **1** (0.3 mmol), $Et_2O$ (3 mL) and $H_2O$ (22 μL) was added to the tube, and stirred at 80 °C. Following complete consumption of the allyl ether **1** (monitored by TLC), solvent was removed under reduced pressure. The crude product was then purified by flash column chromatography on silica gel to afford the desired product.

4.1 Synthesis of (*R*)-4,4,5,5-tetramethyl-2-((3-methyl-2,3-dihydrobenzofuran-3-yl)methyl)-1,3,2-dioxaborolane (**2a**).

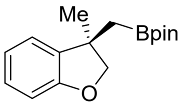


**2a**

Prepared according to typical procedure at 80 °C for 12 h by using $Pd_2(dba)_3$•$CHCl_3$ (2.5 mol%), ***N*-Me-Xu3** (7.5 mol%) from allyl ether **1a** (82.2 mg, 0.3 mmol) , $B_2Pin_2$ (83.8 mg, 0.33 mmol),

$Cs_2CO_3$ (195.5 mg, 0.6 mmol) and $H_2O$ (22 μL, 1.2 mmol), after a flash column chromatography (hexanes: EA = 100:1) afforded the product **2a** as a colorless ropy liquid (81 mg, 98% yield) with 91% *ee*. $^1$H NMR (400 MHz, $CDCl_3$) δ 7.14 (dd, *J* = 7.4, 1.4 Hz, 1H), 7.08 (td, *J* = 7.7, 1.5 Hz, 1H), 6.84 (td, *J* = 7.4, 1.0 Hz, 1H), 6.79-6.72 (m, 1H), 4.41 (d, *J* = 8.6 Hz, 1H), 4.25 (d, *J* = 8.6 Hz, 1H), 1.37 (s, 3H), 1.30 (d, *J* = 15.5 Hz, 1H), 1.24 (d, *J* = 13.7 Hz, 1H), 1.19 (s, 6H), 1.18 (s, 6H). $^{13}$C NMR (101 MHz, $CDCl_3$) δ 158.95, 137.29, 127.64, 122.54, 120.31, 109.40, 84.04, 83.13, 43.11, 28.04, 24.75, 24.69. MS (EI): m/z (%) = 275 ($M+H^+$, 2.75), 133 (100); HRMS calculated for $[C_{16}H_{24}BO_3]^+$: 275.1803 found: 275.1807. Enantiomeric excess was determined by HPLC with a Chiralpak AD-3 column (hexanes : 2-propanol = 99:1, 0.5 mL/min, 220 nm); minor enantiomer tr = 9.8 min, major enantiomer tr = 10.3 min. $[\alpha]_D^{25}$ = 20.6 (c = 0.4, $CHCl_3$).

4.2 Synthesis of (*S*)-2-((3-ethyl-2,3-dihydrobenzofuran-3-yl)methyl)-4,4,5,5-tetramethyl-1,3,2-dioxaborolane (**2b**).

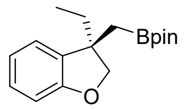


**2b**

Prepared according to typical procedure at 80 °C for 12 h by using $Pd_2(dba)_3$•$CHCl_3$ (2.5 mol%), ***N*-Me-Xu3** (7.5 mol%) from allyl ether **1b** (86.5 mg, 0.3 mmol) , $B_2Pin_2$ (83.8 mg, 0.33 mmol), $Cs_2CO_3$ (195.5 mg, 0.6 mmol) and $H_2O$ (22 μL, 1.2 mmol), after a flash column chromatography (hexanes: EA = 100:1) afforded the product **2b** as a colorless ropy liquid (60.5 mg, 70% yield) with 92% *ee*. $^1$H NMR (400 MHz, $CDCl_3$) δ 7.07-6.96 (m, 2H), 6.79-6.73 (m, 1H), 6.66 (d, *J* = 8.0 Hz, 1H), 4.38-4.25 (m, 2H), 1.70-1.53 (m, 2H), 1.24 (d, *J* = 15.4 Hz, 1H), 1.13 (d, *J* = 15.4 Hz, 1H), 1.08 (s, 6H), 1.05 (s, 6H), 0.72 (t, *J* = 7.4 Hz, 3H). $^{13}$C NMR (101 MHz, $CDCl_3$) δ 159.70, 135.07, 127.70, 123.33, 120.04, 109.17, 83.08, 81.81, 46.90, 34.09, 24.69, 24.63, 8.89. MS (EI): m/z (%) = 289 ($M+H^+$, 7.85), 147 (100); HRMS calculated for $[C_{17}H_{26}BO_3]^+$: 289.1970 found: 289.1964. Enantiomeric excess was determined by HPLC with a Chiralpak IC column (hexanes : 2-propanol = 99:1, 0.5 mL/min, 220 nm); minor enantiomer tr = 9.5 min, major enantiomer tr = 13.0 min. $[\alpha]_D^{25}$ = 25.4 (c = 0.4, $CHCl_3$).

4.3 Synthesis of (*S*)-4,4,5,5-tetramethyl-2-((3-propyl-2,3-dihydrobenzofuran-3-yl)methyl)-1,3,2-dioxaborolane (**2c**).

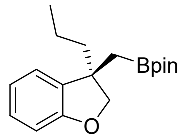


**2c**

Prepared according to typical procedure at 80 °C for 12 h by using $Pd_2(dba)_3$•$CHCl_3$ (2.5 mol%), ***N*-Me-Xu3** (7.5 mol%) from allyl ether **1c** (90.6 mg, 0.3 mmol) , $B_2Pin_2$ (83.8 mg, 0.33 mmol), $Cs_2CO_3$ (195.5 mg, 0.6 mmol) and $H_2O$ (22 μL, 1.2 mmol), after a flash column chromatography (hexanes: EA = 100:1) afforded the product **2c** as a colorless ropy liquid (73 mg, 81% yield) with 91% *ee*. $^1$H NMR (400 MHz, $CDCl_3$) δ 7.08-6.95 (m, 2H), 6.79-6.71 (m, 1H), 6.69-6.62 (m, 1H), 4.35-4.26 (m, 2H), 1.64-1.45 (m, 2H), 1.35-1.22 (m, 2H), 1.22-1.10 (m, 2H), 1.08 (s, 6H), 1.06 (s, 6H), 0.77 (t, *J* = 7.3 Hz, 3H). $^{13}$C NMR (101 MHz, $CDCl_3$) δ 159.59, 135.50, 127.67, 123.30, 120.05, 109.19, 83.07, 82.26, 46.60, 44.10, 24.68, 24.63, 17.79, 14.49. MS (EI): m/z (%) = 303 ($M+H^+$, 7.22), 83 (100); HRMS calculated for $[C_{18}H_{28}BO_3]^+$: 303.2126 found: 303.2120.

Enantiomeric excess was determined by HPLC with a Chiralpak IC column (hexanes : 2-propanol = 99:1, 0.5 mL/min, 220 nm); minor enantiomer tr = 9.1 min, major enantiomer tr = 12.7 min. $[\alpha]_D^{25}$ = 27.7 (c = 0.4, $CHCl_3$).

4.4 Synthesis of (*S*)-2-((3-butyl-2,3-dihydrobenzofuran-3-yl)methyl)-4,4,5,5-tetramethyl-1,3,2-dioxaborolane (**2d**).

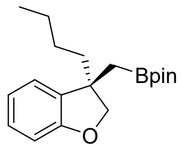


**2d**

Prepared according to typical procedure at 80 °C for 12 h by using $Pd_2(dba)_3$•$CHCl_3$ (2.5 mol%), ***N*-Me-Xu3** (7.5 mol%) from allyl ether **1d** (94.9 mg, 0.3 mmol), $B_2Pin_2$ (83.8 mg, 0.33 mmol), $Cs_2CO_3$ (195.5 mg, 0.6 mmol) and $H_2O$ (22 μL, 1.2 mmol), after a flash column chromatography (hexanes: EA = 100:1) afforded the product **2d** as a colorless ropy liquid (76.6 mg, 81% yield) with 92% *ee*. $^1$H NMR (400 MHz, $CDCl_3$) δ 7.08-6.96 (m, 2H), 6.80-6.72 (m, 1H), 6.69-6.63 (m, 1H), 4.35 -4.27 (m, 2H), 1.67-1.47 (m, 2H), 1.25 (d, *J* = 15.5 Hz, 2H), 1.20-1.10 (m, 4H), 1.09 (s, 6H), 1.06 (s, 6H), 0.77 (t, *J* = 7.2 Hz, 3H). $^{13}$C NMR (101 MHz, $CDCl_3$) δ 159.60, 135.58, 127.68, 123.33, 120.08, 109.23, 83.10, 82.28, 46.54, 41.38, 26.70, 24.70, 24.67, 23.13, 13.96. MS (EI): m/z (%) = 317 ($M+H^+$, 4.40), 83 (100); HRMS calculated for $[C_{19}H_{30}BO_3]^+$: 317.2283 found: 317.2275. Enantiomeric excess was determined by HPLC with a Chiralpak IC column (hexanes : 2-propanol = 99:1, 0.5 mL/min, 220 nm); minor enantiomer tr = 8.9 min, major enantiomer tr = 12.3 min. $[\alpha]_D^{25}$ = 21.8 (c = 0.4, $CHCl_3$).

4.5 Synthesis of (*S*)-2-((3-isopropyl-2,3-dihydrobenzofuran-3-yl)methyl)-4,4,5,5-tetramethyl-1,3,2-dioxaborolane (**2e**).

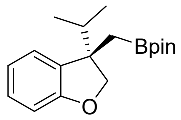


**2e**

Prepared according to typical procedure at 80 °C for 12 h by using $Pd_2(dba)_3$•$CHCl_3$ (2.5 mol%), ***N*-Me-Xu3** (7.5 mol%) from allyl ether **1e** (90.7 mg, 0.3 mmol) , $B_2Pin_2$ (83.8 mg, 0.33 mmol), $Cs_2CO_3$ (195.5 mg, 0.6 mmol) and $H_2O$ (22 μL, 1.2 mmol), after a flash column chromatography (hexanes: EA = 100:1) afforded the product **2e** as a colorless ropy liquid (77.7 mg, 90% yield) with 93% *ee*. $^1$H NMR (400 MHz, $CDCl_3$) δ 7.06-6.95 (m, 2H), 6.77-6.70 (m, 1H), 6.66-6.60 (m, 1H), 4.42-4.30 (m, 2H), 1.91-1.78 (m, 1H), 1.26 (d, *J* = 15.2 Hz, 1H), 1.14 (d, *J* = 15.2 Hz, 1H), 1.02 (s, 6H), 0.97 (s, 6H), 0.78 (d, *J* = 6.8 Hz, 3H), 0.72 (d, *J* = 6.8 Hz, 3H). $^{13}$C NMR (101 MHz, $CDCl_3$) δ 160.08, 133.84, 127.73, 124.05, 119.73, 108.96, 82.94, 79.73, 49.96, 37.15, 24.65, 24.43, 17.74, 17.57. MS (EI): m/z (%) = 303 ($M+H^+$, 3.19), 259 (100); HRMS calculated for $[C_{18}H_{27}BO_3]^+$: 303.2126 found: 303.2119. Enantiomeric excess was determined by HPLC with a Chiralpak AD-3 column (hexanes : 2-propanol = 99:1, 0.5 mL/min, 220 nm); minor enantiomer tr = 9.4 min, major enantiomer tr = 10.1 min. $[\alpha]_D^{25}$ = 32.8 (c = 0.4, $CHCl_3$).

4.6 Synthesis of (*S*)-2-((3-(tert-butyl)-2,3-dihydrobenzofuran-3-yl)methyl)-4,4,5,5-tetramethyl-1,3,2-dioxaborolane (**2f**).

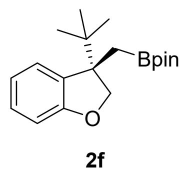

**2f**

Prepared according to typical procedure at 80 °C for 12 h by using $Pd_2(dba)_3$•$CHCl_3$ (2.5 mol%), ***N*-Me-Xu3** (7.5 mol%) from allyl ether **1f** (94.9 mg, 0.3 mmol) , $B_2Pin_2$ (83.8 mg, 0.33 mmol), $Cs_2CO_3$ (195.5 mg, 0.6 mmol) and $H_2O$ (22 µL, 1.2 mmol), after a flash column chromatography (hexanes: EA = 100:1) afforded the product **2f** as a colorless ropy liquid (80.4 mg, 85% yield) with 92% *ee*. $^1$H NMR (400 MHz, $CDCl_3$) δ 7.08-7.02 (m, 1H), 7.02-6.95 (m, 1H), 6.75-6.67 (m, 1H), 6.64 -6.58(m, 1H), 4.55-4.45 (m, 2H), 1.39 (d, *J* = 14.9 Hz, 1H), 1.02 (d, *J* = 14.7 Hz, 1H), 0.93 (s, 6H), 0.82 (s, 15H). $^{13}$C NMR (101 MHz, $CDCl_3$) δ 160.56, 132.61, 127.78, 125.75, 119.19, 108.83, 82.80, 78.75, 52.43, 37.02, 25.22, 24.64, 24.17. MS (EI): m/z (%) = 317 ($M+H^+$, 0.97), 83 (100); HRMS calculated for $[C_{19}H_{30}BO_3]^+$: 317.2283 found: 317.2276. Enantiomeric excess was determined by HPLC with a Chiralpak AD-3 column (hexanes : 2-propanol = 99:1, 0.5 mL/min, 220 nm); minor enantiomer tr = 10.5 min, major enantiomer tr = 8.5 min. $[\alpha]_D^{25}$ = 41.5 (c = 0.4, $CHCl_3$).

4.7 Synthesis of (*S*)-2-((3-benzyl-2,3-dihydrobenzofuran-3-yl)methyl)-4,4,5,5-tetramethyl-1,3,2-dioxaborolane (**2g**).

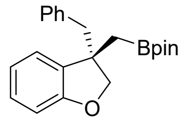

**2g**

Prepared according to typical procedure at 80 °C for 12 h by using $Pd_2(dba)_3$•$CHCl_3$ (2.5 mol%), ***N*-Me-Xu3** (7.5 mol%) from allyl ether **1g** (105.1 mg, 0.3 mmol) , $B_2Pin_2$ (83.8 mg, 0.33 mmol), $Cs_2CO_3$ (195.5 mg, 0.6 mmol) and $H_2O$ (22 µL, 1.2 mmol), after a flash column chromatography (hexanes: EA = 100:1) afforded the product **2g** as a colorless ropy liquid (81.8 mg, 78% yield) with 92% *ee*. $^1$H NMR (400 MHz, $CDCl_3$) δ 7.14-7.07 (m, 3H), 7.05-6.95 (m, 1H), 6.88-6.76 (m, 3H), 6.71 (t, *J* = 7.4 Hz, 1H), 6.62 (d, *J* = 8.0 Hz, 1H), 4.44 (d, *J* = 8.8 Hz, 1H), 4.26 (d, *J* = 8.8 Hz, 1H), 2.86 (s, 2H), 1.26 (d, *J* = 15.6 Hz, 1H), 1.15 (d, *J* = 15.6 Hz, 1H), 1.09 (s, 6H), 1.06 (s, 6H). $^{13}$C NMR (101 MHz, $CDCl_3$) δ 159.58, 137.65, 134.64, 130.50, 127.94, 127.68, 126.24, 124.05, 119.80, 109.32, 83.17, 81.72, 47.51, 46.96, 24.72, 24.64. MS (EI): m/z (%) = 350 ($M^+$, 1.84), 259 (100); HRMS calculated for $[C_{22}H_{27}BO_3Na]^+$: 373.1945 found: 373.1938. Enantiomeric excess was determined by HPLC with a Chiralpak IC column (hexanes : 2-propanol = 99:1, 0.5 mL/min, 220 nm); minor enantiomer tr = 12.1 min, major enantiomer tr = 16.1 min. $[\alpha]_D^{25}$ = 27.5 (c = 0.4, $CHCl_3$).

4.8 Synthesis of (*S*)-4,4,5,5-tetramethyl-2-((3-(4-methylbenzyl)-2,3-dihydrobenzofuran-3-yl)methyl)-1,3,2-dioxaborolane (**2h**).

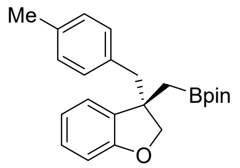

**2h**

Prepared according to typical procedure at 80 °C for 12 h by using $Pd_2(dba)_3$•$CHCl_3$ (2.5 mol%), ***N*-Me-Xu3** (7.5 mol%) from allyl ether **1h** (109.2 mg, 0.3 mmol) , $B_2Pin_2$ (83.8 mg, 0.33 mmol), $Cs_2CO_3$ (195.5 mg, 0.6 mmol) and $H_2O$ (22 μL, 1.2 mmol), after a flash column chromatography (hexanes: EA = 100:1) afforded the product **2h** as a colorless ropy liquid (91.8 mg, 84% yield) with 94% *ee*. $^1H$ NMR (400 MHz, $CDCl_3$) δ 7.11-7.03 (m, 1H), 6.99 (d, *J* = 7.7 Hz, 2H), 6.93-6.87 (m, 1H), 6.85-6.74 (m, 3H), 6.69 (d, *J* = 7.9 Hz, 1H), 4.51 (d, *J* = 8.8 Hz, 1H), 4.33 (d, *J* = 8.8 Hz, 1H), 2.90 (s, 2H), 2.28 (s, 3H), 1.32 (d, *J* = 15.7 Hz, 1H), 1.22 (d, *J* = 15.6 Hz, 1H), 1.15 (s, 6H), 1.13 (s, 6H). $^{13}C$ NMR (101 MHz, $CDCl_3$) δ 159.60, 135.65, 134.79, 134.54, 130.36, 128.41, 127.87, 124.05, 119.78, 109.28, 83.12, 81.69, 47.49, 46.56, 24.71, 24.62, 20.96. MS (EI): m/z (%) = 365 ($M+H^+$, 0.33), 259 (100); HRMS calculated for $[C_{23}H_{30}BO_3]^+$: 365.2283 found: 365.2275. Enantiomeric excess was determined by HPLC with a Chiralpak IC column (hexanes : 2-propanol = 99:1, 0.5 mL/min, 220 nm); minor enantiomer tr = 13.1 min, major enantiomer tr = 16.7 min. $[\alpha]_D^{25}$ = 32.6 (c = 0.4, $CHCl_3$).

4.9 Synthesis of (*S*)-2-((3-(4-fluorobenzyl)-2,3-dihydrobenzofuran-3-yl)methyl)-4,4,5,5-tetramethyl-1,3,2-dioxaborolane (**2i**).

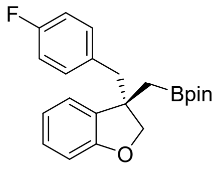


**2i**

Prepared according to typical procedure at 80 °C for 12 h by using $Pd_2(dba)_3$•$CHCl_3$ (2.5 mol%), ***N*-Me-Xu3** (7.5 mol%) from allyl ether **1i** (110.5 mg, 0.3 mmol) , $B_2Pin_2$ (83.8 mg, 0.33 mmol), $Cs_2CO_3$ (195.5 mg, 0.6 mmol) and $H_2O$ (22 μL, 1.2 mmol), after a flash column chromatography (hexanes: EA = 100:1) afforded the product **2i** as a colorless ropy liquid (101.2 mg, 92% yield) with 93% *ee*. $^1H$ NMR (400 MHz, $CDCl_3$) δ 7.11-7.04 (m, 1H), 6.92-6.76 (m, 6H), 6.69 (d, *J* = 8.0 Hz, 1H), 4.48 (d, *J* = 8.9 Hz, 1H), 4.33 (d, *J* = 8.9 Hz, 1H), 2.90 (s, 2H), 1.34 (d, *J* = 15.6 Hz, 1H), 1.22 (d, *J* = 15.6 Hz, 1H), 1.18 (s, 6H), 1.15 (s, 6H). $^{13}C$ NMR (101 MHz, $CDCl_3$) δ 161.65 (d, *J* = 244.3 Hz), 159.60, 134.31, 133.33 (d, *J* = 3.3 Hz), 131.81 (d, *J* = 7.8 Hz), 128.07, 123.99, 119.86, 114.45 (d, *J* = 21.0 Hz), 109.40, 83.27, 81.66, 47.51, 46.11, 24.74, 24.67. $^{19}F$ NMR (376 MHz, $CDCl_3$) δ -116.91. MS (EI): m/z (%) = 369 ($M+H^+$, 0.28), 259 (100); HRMS calculated for $[C_{22}H_{27}BFO_3]^+$: 369.2032 found: 369.2015. Enantiomeric excess was determined by HPLC with a Chiralpak IC column (hexanes : 2-propanol = 99:1, 0.5 mL/min, 220 nm); minor enantiomer tr = 11.0 min, major enantiomer tr = 17.6 min. $[\alpha]_D^{25}$ = 27.4 (c = 0.4, $CHCl_3$).

4.10 Synthesis of (*S*)-2-((3-(4-chlorobenzyl)-2,3-dihydrobenzofuran-3-yl)methyl)-4,4,5,5-tetramethyl-1,3,2-dioxaborolane (**2j**).

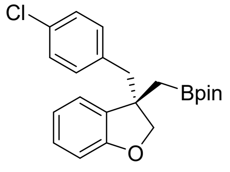


**2j**

Prepared according to typical procedure at 80 °C for 12 h by using $Pd_2(dba)_3$•$CHCl_3$ (2.5 mol%), ***N*-Me-Xu3** (7.5 mol%) from allyl ether **1j** (115.4 mg, 0.3 mmol) , $B_2Pin_2$ (83.8 mg, 0.33 mmol),

$Cs_2CO_3$ (195.5 mg, 0.6 mmol) and $H_2O$ (22 μL, 1.2 mmol), after a flash column chromatography (hexanes: EA = 100:1) afforded the product **2j** as a colorless ropy liquid (110.2 mg, 95% yield) with 93% *ee*. $^1$H NMR (400 MHz, $CDCl_3$) δ 7.18-7.11 (m, 2H), 7.10-7.05 (m, 1H), 6.88-6.75 (m, 4H), 6.69 (d, *J* = 8.0 Hz, 1H), 4.47 (d, *J* = 8.9 Hz, 1H), 4.33 (d, *J* = 8.9 Hz, 1H), 2.89 (s, 2H), 1.30 (d, *J* = 9.7 Hz, 1H), 1.24 (d, *J* = 7.8 Hz, 1H), 1.17 (s, 6H), 1.15 (s, 6H). $^{13}$C NMR (101 MHz, $CDCl_3$) δ 159.55, 136.09, 134.17, 132.18, 131.72, 128.10, 127.77, 123.94, 119.87, 109.43, 83.25, 81.61, 47.44, 46.23, 24.72, 24.63. MS (EI): m/z (%) = 384 ($M^+$, 0.56), 259 (100); HRMS calculated for $[C_{22}H_{27}BClO_3]^+$: 385.1736 found: 385.1725. Enantiomeric excess was determined by HPLC with a Chiralpak IC column (hexanes : 2-propanol = 99:1, 0.5 mL/min, 220 nm); minor enantiomer tr = 11.0 min, major enantiomer tr = 17.8 min. $[\alpha]_D^{25}$ = 38.7 (c = 0.4, $CHCl_3$).

4.11 Synthesis of (*S*)-4,4,5,5-tetramethyl-2-((3-(4-(trifluoromethyl)benzyl)-2,3-dihydrobenzofuran-3-yl)methyl)-1,3,2-dioxaborolane (**2k**).

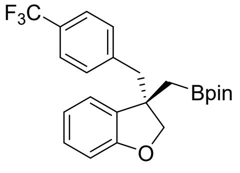


**2k**

Prepared according to typical procedure at 80 °C for 12 h by using $Pd_2(dba)_3$•$CHCl_3$ (2.5 mol%), ***N*-Me-Xu3** (7.5 mol%) from allyl ether **1k** (125.5 mg, 0.3 mmol) , $B_2Pin_2$ (83.8 mg, 0.33 mmol), $Cs_2CO_3$ (195.5 mg, 0.6 mmol) and $H_2O$ (22 μL, 1.2 mmol), after a flash column chromatography (hexanes: EA = 100:1) afforded the product **2k** as a colorless ropy liquid (103.4 mg, 82% yield) with 93% *ee*. $^1$H NMR (400 MHz, $CDCl_3$) δ 7.44 (d, *J* = 8.0 Hz, 2H), 7.14-7.05 (m, 1H), 6.99 (d, *J* = 8.0 Hz, 2H), 6.88-6.77 (m, 2H), 6.71 (d, *J* = 8.0 Hz, 1H), 4.50 (d, *J* = 9.0 Hz, 1H), 4.35 (d, *J* = 8.9 Hz, 1H), 2.99 (s, 2H), 1.35 (d, *J* = 15.7 Hz, 1H), 1.27 (d, *J* = 15.3 Hz, 1H), 1.19 (s, 6H), 1.16 (s, 6H). $^{13}$C NMR (101 MHz, $CDCl_3$) δ 159.51, 141.76, 133.99, 130.73, 128.53 (d, *J* = 32.2 Hz), 128.25, 124.55 (q, *J* = 3.7 Hz), 124.29 (q, *J* = 272.9 Hz), 123.94, 119.98, 109.55, 83.34, 81.66, 47.48, 46.65, 24.74, 24.65. $^{19}$F NMR (376 MHz, $CDCl_3$) δ -62.25. MS (EI): m/z (%) = 418 ($M^+$, 1.40), 259 (100); HRMS calculated for $[C_{23}H_{26}BF_3O_3]^+$: 418.1972 found: 418.1930. Enantiomeric excess was determined by HPLC with a Chiralpak IC column (hexanes : 2-propanol = 99:1, 0.5 mL/min, 220 nm); minor enantiomer tr = 9.3 min, major enantiomer tr = 16.0 min. $[\alpha]_D^{25}$ = 24.7 (c = 0.4, $CHCl_3$).

4.12 Synthesis of (*S*)-2-((3-(3-fluorobenzyl)-2,3-dihydrobenzofuran-3-yl)methyl)-4,4,5,5-tetramethyl-1,3,2-dioxaborolane (**2l**).

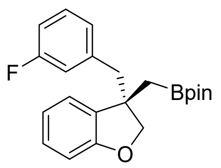


**2l**

Prepared according to typical procedure at 80 °C for 12 h by using $Pd_2(dba)_3$•$CHCl_3$ (2.5 mol%), ***N*-Me-Xu3** (7.5 mol%) from allyl ether **1l** (110.5 mg, 0.3 mmol) , $B_2Pin_2$ (83.8 mg, 0.33 mmol), $Cs_2CO_3$ (195.5 mg, 0.6 mmol) and $H_2O$ (22 μL, 1.2 mmol), after a flash column chromatography (hexanes: EA = 100:1) afforded the product **2l** as a colorless ropy liquid (99.5 mg, 90% yield)

with 92% *ee*. $^1$H NMR (400 MHz, $CDCl_3$) δ 7.10-6.95 (m, 2H), 6.83-6.75 (m, 2H), 6.72 (t, *J* = 7.4 Hz, 1H), 6.60 (t, *J* = 7.4 Hz, 2H), 6.52 (d, *J* = 10.1 Hz, 1H), 4.41 (d, *J* = 8.9 Hz, 1H), 4.24 (d, *J* = 9.0 Hz, 1H), 2.85 (s, 2H), 1.26 (d, *J* = 15.7 Hz, 1H), 1.18 (d, *J* = 16.4 Hz, 1H), 1.09 (s, 6H), 1.07 (s, 6H). $^{13}$C NMR (101 MHz, $CDCl_3$) δ 162.25 (d, *J* = 245.0 Hz), 159.54, 140.20 (d, *J* = 7.3 Hz), 134.26, 128.98 (d, *J* = 8.4 Hz), 128.13, 126.11 (d, *J* = 2.9 Hz), 123.88, 119.92, 117.23 (d, *J* = 21.0 Hz), 113.12 (d, *J* = 20.9 Hz), 109.43, 83.25, 81.65, 47.46, 46.54 (d, *J* = 2.0 Hz), 24.71, 24.62. $^{19}$F NMR (376 MHz, $CDCl_3$) δ -114.10. MS (EI): m/z (%) = 369 (M+H$^+$, 0.32), 259 (100); HRMS calculated for $[C_{22}H_{27}BFO_3]^+$: 369.2032 found: 369.2022. Enantiomeric excess was determined by HPLC with a Chiralpak IC column (hexanes : 2-propanol = 99:1, 0.5 mL/min, 220 nm); minor enantiomer tr = 10.7 min, major enantiomer tr = 15.4 min. $[\alpha]_D^{25}$ = 28.9 (c = 0.4, $CHCl_3$).

4.13 Synthesis of (*S*)-2-((3-(3-chlorobenzyl)-2,3-dihydrobenzofuran-3-yl)methyl)-4,4,5,5-tetramethyl-1,3,2-dioxaborolane (**2m**).

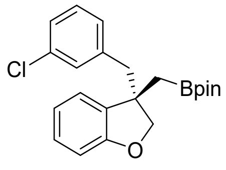


**2m**

Prepared according to typical procedure at 80 °C for 12 h by using $Pd_2(dba)_3$•$CHCl_3$ (2.5 mol%), ***N*-Me-Xu3** (7.5 mol%) from allyl ether **1m** (115.4 mg, 0.3 mmol) , $B_2Pin_2$ (83.8 mg, 0.33 mmol), $Cs_2CO_3$ (195.5 mg, 0.6 mmol) and $H_2O$ (22 μL, 1.2 mmol), after a flash column chromatography (hexanes: EA = 100:1) afforded the product **2m** as a colorless ropy liquid (102.9 mg, 89% yield) with 90% *ee*. $^1$H NMR (400 MHz, $CDCl_3$) δ 7.07 (d, *J* = 8.1 Hz, 1H), 7.01 (t, *J* = 7.7 Hz, 2H), 6.81 (d, *J* = 9.3 Hz, 2H), 6.73 (t, *J* = 7.4 Hz, 1H), 6.68 (d, *J* = 7.5 Hz, 1H), 6.62 (d, *J* = 8.0 Hz, 1H), 4.40 (d, *J* = 8.9 Hz, 1H), 4.24 (d, *J* = 9.1 Hz, 1H), 2.82 (s, 2H), 1.24 (d, *J* = 15.4 Hz, 1H), 1.13 (d, *J* = 16.0 Hz, 1H), 1.10 (s, 6H), 1.07 (s, 6H). $^{13}$C NMR (101 MHz, $CDCl_3$) δ 159.51, 139.70, 134.19, 133.41, 130.50, 128.85, 128.55, 128.17, 126.41, 123.89, 119.91, 109.45, 83.26, 81.65, 47.43, 46.45, 24.74, 24.63. MS (EI): m/z (%) = 385 (M+H$^+$, 0.33), 259 (100); HRMS calculated for $[C_{22}H_{27}BClO_3]^+$: 385.1736 found: 385.1726. Enantiomeric excess was determined by HPLC with a Chiralpak IC column (hexanes : 2-propanol = 99:1, 0.5 mL/min, 220 nm); minor enantiomer tr = 10.6 min, major enantiomer tr = 15.3 min. $[\alpha]_D^{25}$ = 30.0 (c = 0.4, $CHCl_3$).

4.14 Synthesis of (*S*)-4,4,5,5-tetramethyl-2-((3-(3-nitrobenzyl)-2,3-dihydrobenzofuran-3-yl)methyl)-1,3,2-dioxaborolane (**2n**).

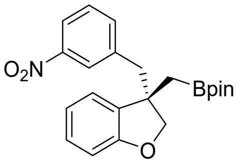


**2n**

Prepared according to typical procedure at 80 °C for 12 h by using $Pd_2(dba)_3$•$CHCl_3$ (2.5 mol%), ***N*-Me-Xu3** (7.5 mol%) from allyl ether **1n** (118.6 mg, 0.3 mmol) , $B_2Pin_2$ (83.8 mg, 0.33 mmol), $Cs_2CO_3$ (195.5 mg, 0.6 mmol) and $H_2O$ (22 μL, 1.2 mmol), after a flash column chromatography (hexanes: EA = 100:1) afforded the product **2n** as a white soild (73.6 mg, 62% yield) with 90% *ee*. $^1$H NMR (400 MHz, $CDCl_3$) δ 8.02-7.86 (m, 1H), 7.71 (t, *J* = 2.0 Hz, 1H), 7.23 (t, *J* = 7.9 Hz, 1H),

7.09-6.97 (m, 2H), 6.83-6.71 (m, 2H), 6.59 (d, $J$ = 8.0 Hz, 1H), 4.42 (d, $J$ = 9.0 Hz, 1H), 4.25 (d, $J$ = 9.0 Hz, 1H), 3.10-2.77 (m, 2H), 1.29 (d, $J$ = 15.8 Hz, 1H), 1.19 (d, $J$ = 11.3 Hz, 1H), 1.15 (s, 6H), 1.12 (s, 6H). $^{13}$C NMR (101 MHz, Chloroform-*d*) δ 159.50, 147.68, 139.67, 136.44, 133.58, 128.50, 128.40, 125.13, 123.70, 121.45, 120.16, 109.64, 83.48, 81.58, 47.52, 46.37, 24.76, 24.68. MS (EI): m/z (%) = 395 (M+H$^+$, 1.63), 259 (100); HRMS calculated for $[C_{22}H_{27}BNO_5]^+$: 395.1904 found: 395.1908. Enantiomeric excess was determined by HPLC with a Chiralpak IC column (hexanes : 2-propanol = 95:5, 0.5 mL/min, 220 nm); minor enantiomer tr = 14.9 min, major enantiomer tr = 19.3 min. $[\alpha]_D^{20}$ = 24.6 (c = 0.4, acetone).

4.15 Synthesis of (*S*)-4,4,5,5-tetramethyl-2-((3-(3-methylbenzyl)-2,3-dihydrobenzofuran-3-yl)methyl)-1,3,2-dioxaborolane (**2o**).

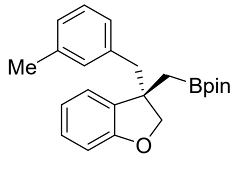


**2o**

Prepared according to typical procedure at 80 °C for 12 h by using $Pd_2(dba)_3$•$CHCl_3$ (2.5 mol%), ***N*-Me-Xu3** (7.5 mol%) from allyl ether **1o** (109.2 mg, 0.3 mmol) , $B_2Pin_2$ (83.8 mg, 0.33 mmol), $Cs_2CO_3$ (195.5 mg, 0.6 mmol) and $H_2O$ (22 μL, 1.2 mmol), after a flash column chromatography (hexanes: EA = 100:1) afforded the product **2o** as a colorless ropy liquid (92.3 mg, 85% yield) with 93% *ee*. $^1$H NMR (400 MHz, $CDCl_3$) δ 7.11-7.03 (m, 2H), 6.99 (d, $J$ = 7.6 Hz, 1H), 6.93-6.88 (m, 1H), 6.83-6.77 (m, 1H), 6.76-6.68 (m, 3H), 4.52 (d, $J$ = 8.8 Hz, 1H), 4.33 (d, $J$ = 8.8 Hz, 1H), 2.90 (s, 2H), 2.24 (s, 3H), 1.32 (d, $J$ = 15.7 Hz, 1H), 1.23 (d, $J$ = 15.6 Hz, 1H), 1.16 (s, 6H), 1.13 (s, 6H). $^{13}$C NMR (101 MHz, $CDCl_3$) δ 159.61, 137.56, 137.08, 134.82, 131.41, 127.91, 127.57, 127.50, 126.95, 124.07, 119.75, 109.29, 83.14, 81.72, 47.47, 46.92, 24.73, 24.63, 21.29. MS (EI): m/z (%) = 365 (M+H$^+$, 0.50), 259 (100); HRMS calculated for $[C_{23}H_{30}BO_3]^+$: 365.2283 found: 365.2274. Enantiomeric excess was determined by HPLC with a Chiralpak IC column (hexanes : 2-propanol = 99:1, 0.5 mL/min, 220 nm); minor enantiomer tr = 11.5 min, major enantiomer tr = 14.9 min. $[\alpha]_D^{25}$ = 28.8 (c = 0.4, $CHCl_3$).

4.16 Synthesis of (*S*)-2-((3-(3-methoxybenzyl)-2,3-dihydrobenzofuran-3-yl)methyl)-4,4,5,5-tetramethyl-1,3,2-dioxaborolane (**2p**).

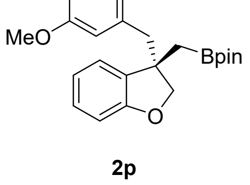


**2p**

Prepared according to typical procedure at 80 °C for 12 h by using $Pd_2(dba)_3$•$CHCl_3$ (2.5 mol%), ***N*-Me-Xu3** (7.5 mol%) from allyl ether **1p** (114.1 mg, 0.3 mmol) , $B_2Pin_2$ (83.8 mg, 0.33 mmol), $Cs_2CO_3$ (195.5 mg, 0.6 mmol) and $H_2O$ (22 μL, 1.2 mmol), after a flash column chromatography (hexanes: EA = 100:1) afforded the product **2p** as a colorless ropy liquid (70.2 mg, 62% yield) with 93% *ee*. $^1$H NMR (400 MHz, $CDCl_3$) δ 7.07-6.97 (m, 2H), 6.88-6.80 (m, 1H), 6.78-6.69 (m, 1H), 6.68-6.59 (m, 2H), 6.48 (d, $J$ = 7.6 Hz, 1H), 6.35-6.28 (m, 1H), 4.44 (d, $J$ = 8.9 Hz, 1H), 4.25 (d, $J$ = 8.8 Hz, 1H), 3.57 (s, 3H), 2.84 (s, 2H), 1.32-1.25 (m, 1H), 1.18 (s, 1H), 1.09 (s, 6H), 1.07

(s, 6H). $^{13}C$ NMR (101 MHz, $CDCl_3$) δ 159.63, 158.93, 139.19, 134.69, 128.58, 127.97, 124.06, 122.94, 119.80, 115.71, 112.23, 109.37, 83.19, 81.70, 54.95, 47.52, 46.99, 24.72, 24.67. MS (EI): m/z (%) = 380 ($M^+$, 1.94), 259 (100); HRMS calculated for $[C_{23}H_{29}BO_4]^+$: 381.2232 found: 381.2221. Enantiomeric excess was determined by HPLC with a Chiralpak IC column (hexanes : 2-propanol = 99:1, 0.5 mL/min, 220 nm); minor enantiomer tr = 9.4 min, major enantiomer tr = 12.1 min. $[\alpha]_D^{25}$ = 21.4 (c = 0.4, $CHCl_3$).

4.17 Synthesis of (*S*)-2-((3-(3-(tert-butyl)benzyl)-2,3-dihydrobenzofuran-3-yl)methyl)-4,4,5,5-tetramethyl-1,3,2-dioxaborolane (**2q**).

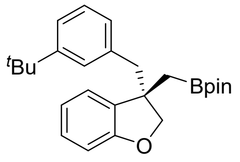


**2q**

Prepared according to typical procedure at 80 °C for 12 h by using $Pd_2(dba)_3$•$CHCl_3$ (2.5 mol%), ***N*-Me-Xu3** (7.5 mol%) from allyl ether **1q** (121.9 mg, 0.3 mmol), $B_2Pin_2$ (83.8 mg, 0.33 mmol), $Cs_2CO_3$ (195.5 mg, 0.6 mmol) and $H_2O$ (22 μL, 1.2 mmol), after a flash column chromatography (hexanes: EA = 100:1) afforded the product **2q** as a colorless ropy liquid (114.6 mg, 94% yield) with 92% *ee*. $^1H$ NMR (400 MHz, $CDCl_3$) δ 7.25-7.11 (m, 2H), 7.10-7.03 (m, 1H), 6.89-6.74 (m, 4H), 6.72-6.66 (m, 1H), 4.52 (d, *J* = 8.8 Hz, 1H), 4.34 (d, *J* = 8.8 Hz, 1H), 3.12-2.83 (m, 2H), 1.32 (d, *J* = 7.2 Hz, 1H), 1.25-1.22 (m, 1H), 1.20 (s, 9H), 1.18 (s, 6H), 1.15 (s, 6H). $^{13}C$ NMR (101 MHz, $CDCl_3$) δ 159.60, 150.22, 137.08, 134.62, 127.88, 127.82, 127.60, 127.32, 124.23, 123.08, 119.77, 109.30, 83.16, 81.92, 47.54, 47.12, 34.36, 31.22, 24.72. MS (EI): m/z (%) = 407 ($M+H^+$, 0.33), 259 (100); HRMS calculated for $[C_{26}H_{36}BO_3]^+$: 407.2752 found: 404.2744. Enantiomeric excess was determined by HPLC with a Chiralpak IC column (hexanes : 2-propanol = 99:1, 0.5 mL/min, 220 nm); minor enantiomer tr = 9.3 min, major enantiomer tr = 12.6 min. $[\alpha]_D^{25}$ = 24.6 (c = 0.4, $CHCl_3$).

4.18 Synthesis of (*S*)-4,4,5,5-tetramethyl-2-((3-(2-methylbenzyl)-2,3-dihydrobenzofuran-3-yl)methyl)-1,3,2-dioxaborolane (**2r**).

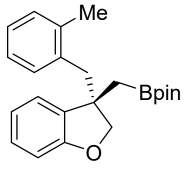


**2r**

Prepared according to typical procedure at 80 °C for 12 h by using $Pd_2(dba)_3$•$CHCl_3$ (2.5 mol%), ***N*-Me-Xu3** (7.5 mol%) from allyl ether **1r** (109.2 mg, 0.3 mmol) , $B_2Pin_2$ (83.8 mg, 0.33 mmol), $Cs_2CO_3$ (195.5 mg, 0.6 mmol) and $H_2O$ (22 μL, 1.2 mmol), after a flash column chromatography (hexanes: EA = 100:1) afforded the product **2r** as a white soild (77.7 mg, 71% yield) with 94% *ee*. $^1H$ NMR (400 MHz, $CDCl_3$) δ 7.12-7.00 (m, 4H), 6.96-6.92 (m, 1H), 6.74-6.69 (m, 2H), 6.65-6.54 (m, 1H), 4.48 (d, *J* = 8.8 Hz, 1H), 4.39 (d, *J* = 8.9 Hz, 1H), 3.09 (d, *J* = 13.5 Hz, 1H), 2.88 (d, *J* = 13.5 Hz, 1H), 1.81 (s, 3H), 1.47 (d, *J* = 15.8 Hz, 1H), 1.27 (d, *J* = 12.7 Hz, 1H), 1.18 (s, 6H), 1.15 (s, 6H). $^{13}C$ NMR (101 MHz, $CDCl_3$) δ 159.57, 137.65, 135.99, 134.64, 131.06, 130.14, 127.91, 126.30, 125.13, 124.00, 119.93, 109.23, 83.23, 82.38, 47.84, 42.35, 24.76, 24.62, 19.47. MS (EI):

m/z (%) = 365 ($M+H^+$, 0.34), 259 (100); HRMS calculated for $[C_{23}H_{30}BO_3]^+$: 365.2283 found: 365.2277. Enantiomeric excess was determined by HPLC with a Chiralpak IC column (hexanes : 2-propanol = 99:1, 0.5 mL/min, 220 nm); minor enantiomer tr = 10.6 min, major enantiomer tr = 15.8 min. $[\alpha]_D^{25}$ = 34.2 (c = 0.4, $CHCl_3$).

4.19 Synthesis of (*S*)-2-((3-(3,5-difluorobenzyl)-2,3-dihydrobenzofuran-3-yl)methyl)-4,4,5,5-tetramethyl-1,3,2-dioxaborolane (**2s**).

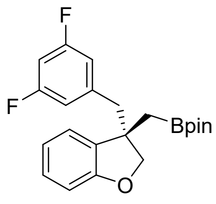


**2s**

Prepared according to typical procedure at 80 °C for 12 h by using $Pd_2(dba)_3$•$CHCl_3$ (2.5 mol%), ***N*-Me-Xu3** (7.5 mol%) from allyl ether **1s** (115.9 mg, 0.3 mmol) , $B_2Pin_2$ (83.8 mg, 0.33 mmol), $Cs_2CO_3$ (195.5 mg, 0.6 mmol) and $H_2O$ (22 μL, 1.2 mmol), after a flash column chromatography (hexanes: EA = 100:1) afforded the product **2s** as a colorless ropy liquid (101.7 mg, 88% yield) with 92% *ee*. $^1H$ NMR (400 MHz, $CDCl_3$) δ 7.12 (d, *J* = 7.9 Hz, 1H), 6.91 (d, *J* = 7.3 Hz, 1H), 6.83 (t, *J* = 7.3 Hz, 1H), 6.72 (s, 1H), 6.63 (t, *J* = 9.0 Hz, 1H), 6.41 (d, *J* = 6.5 Hz, 2H), 4.47 (d, *J* = 9.0 Hz, 1H), 4.32 (d, *J* = 8.9 Hz, 1H), 2.92 (d, *J* = 1.7 Hz, 2H), 1.36 (d, *J* = 16.2 Hz, 1H), 1.27 (d, *J* = 15.9 Hz, 1H), 1.20 (s, 6H), 1.17 (s, 6H). $^{13}C$ NMR (101 MHz, $CDCl_3$) δ 162.28 (dd, *J* = 247.4, 12.9 Hz), 159.48, 141.53 (t, *J* = 9.1 Hz), 133.88, 128.38, 123.74, 120.11, 113.15 (dd, *J* = 24.6, 11.9 Hz), 109.58, 101.80 (t, *J* = 25.3 Hz), 83.38, 81.59, 47.46, 46.47, 24.76, 24.66. $^{19}F$ NMR (376 MHz, $CDCl_3$) δ -111.05. MS (EI): m/z (%) = 386 ($M^+$, 2.08), 259 (100); HRMS calculated for $[C_{22}H_{25}BF_2O_3]^+$: 386.1865 found: 386.1867. Enantiomeric excess was determined by HPLC with a Chiralpak IC column (hexanes : 2-propanol = 99:1, 0.5 mL/min, 220 nm); minor enantiomer tr = 9.6 min, major enantiomer tr = 15.3 min. $[\alpha]_D^{25}$ = 21.8 (c = 0.4, $CHCl_3$).

4.20 Synthesis of (*S*)-4,4,5,5-tetramethyl-2-((3-(3,4,5-trifluorobenzyl)-2,3-dihydrobenzofuran-3-yl)methyl)-1,3,2-dioxaborolane (**2t**).

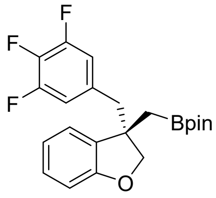


**2t**

Prepared according to typical procedure at 80 °C for 12 h by using $Pd_2(dba)_3$•$CHCl_3$ (2.5 mol%), ***N*-Me-Xu3** (7.5 mol%) from allyl ether **1t** (121.3 mg, 0.3 mmol) , $B_2Pin_2$ (83.8 mg, 0.33 mmol), $Cs_2CO_3$ (195.5 mg, 0.6 mmol) and $H_2O$ (22 μL, 1.2 mmol), after a flash column chromatography (hexanes: EA = 100:1) afforded the product **2t** as a colorless ropy liquid (86 mg, 71% yield) with 94% *ee*. $^1H$ NMR (400 MHz, $CDCl_3$) δ 7.12 (t, *J* = 7.6 Hz, 1H), 6.95-6.79 (m, 2H), 6.70 (d, *J* = 8.0 Hz, 1H), 6.52-6.40 (m, 2H), 4.44 (d, *J* = 9.0 Hz, 1H), 4.31 (d, *J* = 9.0 Hz, 1H), 2.86 (s, 2H), 1.36 (d, *J* = 15.6 Hz, 1H), 1.25 (s, 1H), 1.21 (s, 6H), 1.18 (s, 6H). $^{13}C$ NMR (101 MHz, $CDCl_3$) δ 159.50, 150.34 (ddd, *J* = 248.8, 9.8, 4.0 Hz), 138.47 (dt, *J* = 249.8, 15.3 Hz), 133.93 (td, *J* = 7.3, 4.8 Hz), 133.57, 128.51, 123.63, 120.18, 114.14 (dd, *J* = 15.5, 5.3 Hz), 109.65, 83.45, 81.42, 47.44, 46.13, 24.75, 24.66. $^{19}F$ NMR (376 MHz, $CDCl_3$) δ -135.68 (d, *J* = 20.6 Hz), -163.79 (t, *J* =

20.7 Hz). MS (EI): m/z (%) = 404 ($M^+$, 1.58), 259 (100); HRMS calculated for $[C_{22}H_{24}BF_3O_3]^+$: 404.1771 found: 404.1766. Enantiomeric excess was determined by HPLC with a Chiralpak IC column (hexanes : 2-propanol = 99:1, 0.5 mL/min, 220 nm); minor enantiomer tr = 9.8 min, major enantiomer tr = 18.1 min. $[\alpha]_D^{25}$ = 19.2 (c = 0.4, $CHCl_3$).

4.21 Synthesis of (*S*)-4,4,5,5-tetramethyl-2-((3-(naphthalen-1-ylmethyl)-2,3-dihydrobenzofuran-3-yl)methyl)-1,3,2-dioxaborolane (**2u**).

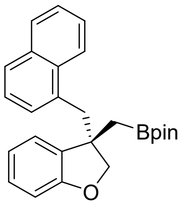

**2u**

Prepared according to typical procedure at 80 °C for 12 h by using $Pd_2(dba)_3$•$CHCl_3$ (2.5 mol%), ***N*-Me-Xu3** (7.5 mol%) from allyl ether **1u** (120.0 mg, 0.3 mmol) , $B_2Pin_2$ (83.8 mg, 0.33 mmol), $Cs_2CO_3$ (195.5 mg, 0.6 mmol) and $H_2O$ (22 μL, 1.2 mmol), after a flash column chromatography (hexanes: EA = 100:1) afforded the product **2u** as a white soild (107.8 mg, 90% yield) with 92% *ee*. $^1H$ NMR (400 MHz, $CDCl_3$) δ 7.79 (s, 1H), 7.68 (d, *J* = 7.7 Hz, 1H), 7.58 (d, *J* = 8.2 Hz, 1H), 7.34-7.25 (m, 1H), 7.25-7.19 (m, 1H), 7.19-7.13 (m, 1H), 7.00-6.92 (m, 1H), 6.88 (d, *J* = 6.6 Hz, 1H), 6.67 (d, *J* = 7.2 Hz, 1H), 6.59 (t, *J* = 8.6 Hz, 2H), 4.46 (d, *J* = 8.9 Hz, 1H), 4.22 (d, *J* = 8.9 Hz, 1H), 3.52 (d, *J* = 13.8 Hz, 1H), 3.20 (d, *J* = 13.7 Hz, 1H), 1.39 (d, *J* = 15.9 Hz, 1H), 1.25 (d, *J* = 15.8 Hz, 1H), 1.10 (s, 6H), 1.07 (s, 6H). $^{13}C$ NMR (101 MHz, $CDCl_3$) δ 159.44, 135.01, 134.07, 133.65, 133.29, 128.66, 128.46, 127.92, 127.03, 125.27, 124.99, 124.83, 124.34, 123.96, 119.96, 109.34, 83.26, 81.60, 48.28, 41.82, 24.83, 24.60. MS (EI): m/z (%) = 401 ($M+H^+$, 0.21), 259 (100); HRMS calculated for $[C_{26}H_{30}BO_3]^+$: 401.2283 found: 401.2273. Enantiomeric excess was determined by HPLC with a Chiralpak IC column (hexanes : 2-propanol = 99:1, 0.5 mL/min, 220 nm); minor enantiomer tr = 14.4 min, major enantiomer tr = 24.9 min. $[\alpha]_D^{25}$ = -34.7 (c = 0.4, $CHCl_3$).

4.22 Synthesis of (*S*)-2-((3-(dibenzo[b,d]thiophen-4-ylmethyl)-2,3-dihydrobenzofuran-3-yl)methyl)-4,4,5,5-tetramethyl-1,3,2-dioxaborolane (**2v**).

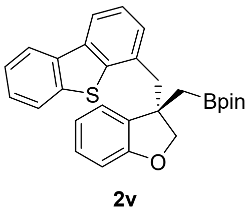

**2v**

Prepared according to typical procedure at 80 °C for 12 h by using $Pd_2(dba)_3$•$CHCl_3$ (2.5 mol%), ***N*-Me-Xu3** (7.5 mol%) from allyl ether **1v** (136.9 mg, 0.3 mmol) , $B_2Pin_2$ (83.8 mg, 0.33 mmol), $Cs_2CO_3$ (195.5 mg, 0.6 mmol) and $H_2O$ (22 μL, 1.2 mmol), after a flash column chromatography (hexanes: EA = 100:1) afforded the product **2v** as a colorless ropy liquid (112.2 mg, 82% yield) with 90% *ee*. $^1H$ NMR (400 MHz, $CDCl_3$) δ 8.12-8.01 (m, 1H), 7.95 (s, 1H), 7.83-7.70 (m, 1H), 7.47-7.32 (m, 2H), 7.23 (t, *J* = 7.8 Hz, 1H), 7.08 (t, *J* = 7.7 Hz, 1H), 6.97 (d, *J* = 7.3 Hz, 1H), 6.86-6.73 (m, 2H), 6.70 (d, *J* = 8.0 Hz, 1H), 4.65 (d, *J* = 9.0 Hz, 1H), 4.38 (d, *J* = 9.0 Hz, 1H), 3.32-3.17 (m, 2H), 1.54-1.38 (m, 2H), 1.17 (s, 6H), 1.13 (s, 6H). $^{13}C$ NMR (101 MHz, $CDCl_3$) δ 159.68, 141.40, 138.99, 136.07, 135.51, 134.56, 132.57, 128.38, 128.16, 126.46, 124.19, 123.83,

122.50, 121.55, 120.10, 119.74, 109.49, 83.27, 81.62, 48.39, 46.10, 24.81, 24.60. MS (EI): m/z (%) = 456 ($M^+$, 1.11), 259 (100); HRMS calculated for $[C_{28}H_{30}BO_3S]^+$: 457.2003 found: 457.1996. Enantiomeric excess was determined by HPLC with a Chiralpak IC column (hexanes : 2-propanol = 99:1, 0.5 mL/min, 220 nm); minor enantiomer tr = 20.6 min, major enantiomer tr = 30.2 min. $[\alpha]_D^{25}$ = -46.1 (c = 0.4, $CHCl_3$).

4.23 Synthesis of (*S*)-trimethyl((3-((4,4,5,5-tetramethyl-1,3,2-dioxaborolan-2-yl)methyl)-2,3-dihydrobenzofuran-3-yl)methyl)silane (**2w**).

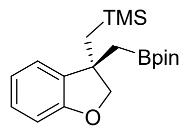


**2w**

Prepared according to typical procedure at 80 °C for 12 h by using $Pd_2(dba)_3$•$CHCl_3$ (2.5 mol%), ***N*-Me-Xu3** (7.5 mol%) from allyl ether **1w** (103.8 mg, 0.3 mmol) , $B_2Pin_2$ (83.8 mg, 0.33 mmol), $Cs_2CO_3$ (195.5 mg, 0.6 mmol) and $H_2O$ (22 μL, 1.2 mmol), after a flash column chromatography (hexanes: EA = 100:1) afforded the product **2w** as a colorless ropy liquid (93.3 mg, 90% yield) with 92% *ee*. $^1$H NMR (400 MHz, $CDCl_3$) δ 7.30-7.15 (m, 2H), 7.00-6.81 (m, 2H), 4.54 (d, *J* = 8.7 Hz, 1H), 4.45 (d, *J* = 8.7 Hz, 1H), 1.52 (d, *J* = 15.2 Hz, 1H), 1.45-1.36 (m, 2H), 1.28 (s, 6H), 1.25 (s, 6H), 1.19 (d, *J* = 14.7 Hz, 1H), 0.00 (s, 9H). $^{13}$C NMR (101 MHz, $CDCl_3$) δ 158.97, 137.32, 127.62, 123.23, 120.16, 109.35, 83.71, 83.04, 45.56, 32.05, 24.70. MS (EI): m/z (%) = 346 ($M^+$, 5.71), 259 (100); HRMS calculated for $[C_{19}H_{31}BO_3Si]^+$: 346.2136 found: 346.2139. Enantiomeric excess was determined by HPLC with a Chiralpak IC column (hexanes : 2-propanol = 99:1, 0.5 mL/min, 220 nm); minor enantiomer tr = 9.0 min, major enantiomer tr = 11.8 min. $[\alpha]_D^{25}$ = 6.1 (c = 0.4, $CHCl_3$).

4.24 Synthesis of (*S*)-2-((3-(4-chlorobutyl)-2,3-dihydrobenzofuran-3-yl)methyl)-4,4,5,5-tetramethyl-1,3,2-dioxaborolane (**2x**).

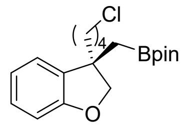


**2x**

Prepared according to typical procedure at 80 °C for 12 h by using $Pd_2(dba)_3$•$CHCl_3$ (2.5 mol%), ***N*-Me-Xu3** (7.5 mol%) from allyl ether **1x** (105.2 mg, 0.3 mmol) , $B_2Pin_2$ (83.8 mg, 0.33 mmol), $Cs_2CO_3$ (195.5 mg, 0.6 mmol) and $H_2O$ (22 μL, 1.2 mmol), after a flash column chromatography (hexanes: EA = 100:1) afforded the product **2x** as a colorless ropy liquid (87.9 mg, 84% yield) with 97% *ee*. $^1$H NMR (400 MHz, $CDCl_3$) δ 7.10 (dd, *J* = 12.2, 7.5 Hz, 2H), 6.84 (t, *J* = 7.4 Hz, 1H), 6.74 (d, *J* = 7.9 Hz, 1H), 4.38 (s, 2H), 3.46 (t, *J* = 6.8 Hz, 2H), 1.77-1.56 (m, 4H), 1.47 (q, *J* = 14.8, 13.5 Hz, 1H), 1.38-1.20 (m, 3H), 1.16 (s, 6H), 1.14 (s, 6H). $^{13}$C NMR (101 MHz, $CDCl_3$) δ 159.53, 135.06, 127.86, 123.21, 120.17, 109.33, 83.14, 82.09, 46.42, 44.63, 40.70, 32.93, 24.68, 24.64, 21.91. MS (EI): m/z (%) = 350 ($M^+$, 16.07), 259 (100); HRMS calculated for $[C_{19}H_{28}BClO_3]^+$: 350.1820 found: 350.1816. Enantiomeric excess was determined by HPLC with a Chiralpak IC column (hexanes : 2-propanol = 99:1, 0.5 mL/min, 220 nm); minor enantiomer tr = 11.6 min, major enantiomer tr = 19.0 min. $[\alpha]_D^{25}$ = 20.9 (c = 0.4, $CHCl_3$).

4.25 Synthesis of (*S*)-2-(4-(3-((4,4,5,5-tetramethyl-1,3,2-dioxaborolan-2-yl)methyl)-2,3-

dihydrobenzofuran-3-yl)butyl)isoindoline-1,3-dione (**2y**).

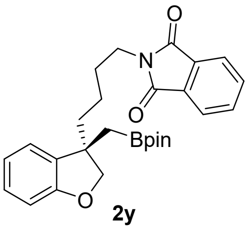


**2y**

Prepared according to typical procedure at 80 °C for 12 h by using $Pd_2(dba)_3$•$CHCl_3$ (2.5 mol%), ***N*-Me-Xu3** (7.5 mol%) from allyl ether **1y** (138.3 mg, 0.3 mmol) , $B_2Pin_2$ (83.8 mg, 0.33 mmol), $Cs_2CO_3$ (195.5 mg, 0.6 mmol) and $H_2O$ (22 μL, 1.2 mmol), after a flash column chromatography (hexanes: EA = 100:1) afforded the product **2y** as a colorless ropy liquid (101.6 mg, 74% yield) with 90% *ee*. $^1$H NMR (400 MHz, $CDCl_3$) δ 7.88-7.77 (m, 2H), 7.73-7.65 (m, 2H), 7.15-7.00 (m, 2H), 6.82 (t, $J$ = 7.3 Hz, 1H), 6.72 (d, $J$ = 7.9 Hz, 1H), 4.38 (s, 2H), 3.60 (t, $J$ = 7.5 Hz, 2H), 1.82-1.53 (m, 4H), 1.44-1.18 (m, 4H), 1.15 (s, 6H), 1.13 (s, 6H). $^{13}$C NMR (101 MHz, $CDCl_3$) δ 168.19, 159.47, 134.96, 133.73, 132.02, 127.74, 123.20, 123.03, 120.09, 109.19, 83.06, 81.97, 46.37, 40.95, 37.65, 28.85, 24.63, 24.57, 21.75. MS (EI): m/z (%) = 461 (M+H$^+$, 13.22), 259 (100); HRMS calculated for $[C_{27}H_{32}BNO_5]^+$: 461.2374 found: 461.2376. Enantiomeric excess was determined by HPLC with a Chiralpak IC column (hexanes : 2-propanol = 99:1, 0.5 mL/min, 220 nm); minor enantiomer tr = 18.0 min, major enantiomer tr = 21.8 min. $[\alpha]_D^{25}$ = 24.4 (c = 0.4, $CHCl_3$).

4.26 Synthesis of (*R*)-3-methyl-1-((3-((4,4,5,5-tetramethyl-1,3,2-dioxaborolan-2-yl)methyl)-2,3-dihydrobenzofuran-3-yl)methyl)-1H-indole (**2z**).

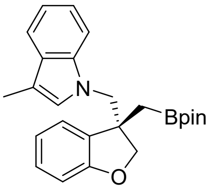


**2z**

Prepared according to typical procedure at 80 °C for 12 h by using $Pd_2(dba)_3$•$CHCl_3$ (2.5 mol%), ***N*-Me-Xu3** (7.5 mol%) from allyl ether **1z** (121.0 mg, 0.3 mmol) , $B_2Pin_2$ (83.8 mg, 0.33 mmol), $Cs_2CO_3$ (195.5 mg, 0.6 mmol) and $H_2O$ (22 μL, 1.2 mmol), after a flash column chromatography (hexanes: EA = 100:1) afforded the product **2z** as a colorless ropy liquid (66.0 mg, 55% yield) with 92% *ee*. $^1$H NMR (400 MHz, $CDCl_3$) δ 7.53-7.45 (m, 1H), 7.17-7.01 (m, 4H), 6.99-6.92 (m, 1H), 6.88-6.71 (m, 2H), 6.53 (s, 1H), 4.53 (d, $J$ = 9.2 Hz, 1H), 4.27 (ddd, $J$ = 29.2, 16.0, 9.6 Hz, 3H), 2.27-2.23 (m, 3H), 1.44 (d, $J$ = 16.0 Hz, 1H), 1.35 (d, $J$ = 15.9 Hz, 1H), 1.19 (s, 6H), 1.15 (s, 6H). $^{13}$C NMR (101 MHz, $CDCl_3$) δ 159.76, 137.71, 133.05, 128.68, 128.24, 126.62, 124.03, 121.34, 120.42, 118.71, 118.49, 110.47, 109.70, 109.39, 83.51, 80.20, 53.92, 49.24, 24.85, 24.59, 9.51. MS (EI): m/z (%) = 404 (M+H$^+$, 6.92), 145 (100); HRMS calculated for $[C_{25}H_{31}BNO_3]^+$: 404.2392 found: 404.2382. Enantiomeric excess was determined by HPLC with a Chiralpak IC column (hexanes : 2-propanol = 99:1, 0.5 mL/min, 220 nm); minor enantiomer tr = 13.5 min, major enantiomer tr = 19.5 min. $[\alpha]_D^{25}$ = -14.2 (c = 0.4, $CHCl_3$).

4.27 Synthesis of (*S*)-4,4,5,5-tetramethyl-2-((3-phenyl-2,3-dihydrobenzofuran-3-yl)methyl)-1,3,2-dioxaborolane (**2aa**).

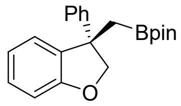

**2aa**

Prepared according to typical procedure at 80 °C for 12 h by using $Pd_2(dba)_3$•$CHCl_3$ (2.5 mol%), ***N*-Me-Xu3** (7.5 mol%) from allyl ether **1aa** (100.9 mg, 0.3 mmol) , $B_2Pin_2$ (83.8 mg, 0.33 mmol), $Cs_2CO_3$ (195.5 mg, 0.6 mmol), $H_2O$ (22 μL, 1.2 mmol) and EA (3 mL), after a flash column chromatography (hexanes: EA = 100:1) afforded the product **1aa** as a colorless ropy liquid (51.8 mg, 52% yield) with 82% *ee*. $^1$H NMR (400 MHz, $CDCl_3$) δ 7.32-7.22 (m, 4H), 7.19-7.09 (m, 3H), 6.92-6.77 (m, 2H), 4.77-4.68 (m, 2H), 1.84 (d, *J* = 15.4 Hz, 1H), 1.61 (d, *J* = 15.4 Hz, 1H), 1.05 (s, 12H). $^{13}$C NMR (101 MHz, $CDCl_3$) δ 159.60, 147.65, 135.70, 128.21, 128.13, 126.17, 126.07, 124.71, 120.60, 109.69, 85.44, 83.20, 50.94, 24.60, 24.48. ESI-MS calculated for $C_{21}H_{25}BNaO_3$: m/z (%): 359.1789 (M+Na$^+$), found: 359.1791. Enantiomeric excess was determined by HPLC with a Chiralpak OJH column (hexanes : 2-propanol = 90:10, 0.5 mL/min, 220 nm); minor enantiomer tr = 16.5 min, major enantiomer tr = 22.0 min. $[\alpha]_D^{20}$ = -3.1 (c = 0.3, CHCl3).

4.28 Synthesis of (*R*)-2-((5-fluoro-3-methyl-2,3-dihydrobenzofuran-3-yl)methyl)-4,4,5,5-tetramethyl-1,3,2-dioxaborolane (**4a**).

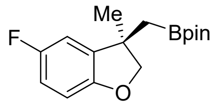

**4a**

Prepared according to typical procedure at 80 °C for 12 h by using $Pd_2(dba)_3$•$CHCl_3$ (2.5 mol%), ***N*-Me-Xu3** (7.5 mol%) from allyl ether **3a** (87.6 mg, 0.3 mmol) , $B_2Pin_2$ (83.8 mg, 0.33 mmol), $Cs_2CO_3$ (195.5 mg, 0.6 mmol) and $H_2O$ (22 μL, 1.2 mmol), after a flash column chromatography (hexanes: EA = 100:1) afforded the product **4a** as a colorless ropy liquid (80.2 mg, 92% yield) with 90% *ee*. $^1$H NMR (400 MHz, $CDCl_3$) δ 6.86 (dd, *J* = 8.2, 2.7 Hz, 1H), 6.75 (td, *J* = 8.8, 2.8 Hz, 1H), 6.64 (dd, *J* = 8.6, 4.1 Hz, 1H), 4.41 (d, *J* = 8.6 Hz, 1H), 4.25 (d, *J* = 8.6 Hz, 1H), 1.36 (s, 3H), 1.33-1.25 (m, 2H), 1.21 (s, 6H), 1.20 (s, 6H). $^{13}$C NMR (101 MHz, $CDCl_3$) δ 157.55 (d, *J* = 236.6 Hz), 154.84 (d, *J* = 1.5 Hz), 138.78 (d, *J* = 7.8 Hz), 113.67 (d, *J* = 24.2 Hz), 109.89 (d, *J* = 24.6 Hz), 109.49 (d, *J* = 8.4 Hz), 84.72, 83.25, 43.58, 27.61, 24.74, 24.70. $^{19}$F NMR (282 MHz, $CDCl_3$) δ -124.34. MS (EI): m/z (%) = 292 (M$^+$, 20.62), 151 (100); HRMS calculated for $[C_{16}H_{22}BFO_3]^+$: 292.1646 found: 292.1642. Enantiomeric excess was determined by HPLC with a Chiralpak IC column (hexanes : 2-propanol = 99:1, 0.5 mL/min, 220 nm); minor enantiomer tr = 8.9 min, major enantiomer tr = 9.7 min. $[\alpha]_D^{20}$ = 14.3 (c = 0.4, acetone).

4.29 Synthesis of (*R*)-2-((5-chloro-3-methyl-2,3-dihydrobenzofuran-3-yl)methyl)-4,4,5,5-tetramethyl-1,3,2-dioxaborolane (**4b**).

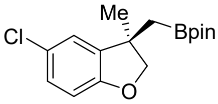

**4b**

Prepared according to typical procedure at 80 °C for 12 h by using $Pd_2(dba)_3$•$CHCl_3$ (2.5 mol%), ***N*-Me-Xu3** (7.5 mol%) from allyl ether **3b** (92.6 mg, 0.3 mmol) , $B_2Pin_2$ (83.8 mg, 0.33 mmol), $Cs_2CO_3$ (195.5 mg, 0.6 mmol) and $H_2O$ (22 μL, 1.2 mmol), after a flash column chromatography (hexanes: EA = 100:1) afforded the product **4b** as a colorless ropy liquid (79.7 mg, 86% yield)

with 90% *ee*. $^1$H NMR (400 MHz, $CDCl_3$) δ 7.04 (d, *J* = 2.3 Hz, 1H), 6.97-6.91 (m, 1H), 6.58 (d, *J* = 8.4 Hz, 1H), 4.33 (d, *J* = 8.7 Hz, 1H), 4.18 (d, *J* = 8.6 Hz, 1H), 1.29 (s, 3H), 1.20 (d, *J* = 15.5 Hz, 2H), 1.13 (s, 6H), 1.12 (s, 6H). $^{13}$C NMR (101 MHz, $CDCl_3$) δ 157.70, 139.20, 127.49, 124.92, 123.08, 110.41, 84.77, 83.27, 43.43, 27.70, 24.77, 24.69. MS (EI): m/z (%) = 309 (M+H$^+$, 6.67), 167 (100); HRMS calculated for $[C_{16}H_{23}BClO_3]^+$: 309.1423 found: 309.1427. Enantiomeric excess was determined by HPLC with a Chiralpak AD-3 column (hexanes : 2-propanol = 99:1, 0.5 mL/min, 220 nm); minor enantiomer tr = 10.3 min, major enantiomer tr = 11.1 min. $[\alpha]_D^{25}$ = 34.2 (c = 0.4, $CHCl_3$).

4.30 Synthesis of (*R*)-2-((3,5-dimethyl-2,3-dihydrobenzofuran-3-yl)methyl)-4,4,5,5-tetramethyl-1,3,2-dioxaborolane (**4c**).

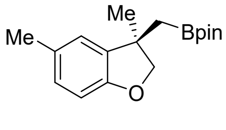


**4c**

Prepared according to typical procedure at 80 °C for 12 h by using $Pd_2(dba)_3$•$CHCl_3$ (2.5 mol%), ***N*-Me-Xu3** (7.5 mol%) from allyl ether **3c** (86.5 mg, 0.3 mmol) , $B_2Pin_2$ (83.8 mg, 0.33 mmol), $Cs_2CO_3$ (195.5 mg, 0.6 mmol) and $H_2O$ (22 μL, 1.2 mmol), after a flash column chromatography (hexanes: EA = 100:1) afforded the product **4c** as a colorless ropy liquid (77.3 mg, 89% yield) with 90% *ee*. $^1$H NMR (400 MHz, $CDCl_3$) δ 6.87 (d, *J* = 1.9 Hz, 1H), 6.82-6.76 (m, 1H), 6.56 (d, *J* = 8.1 Hz, 1H), 4.31 (d, *J* = 8.6 Hz, 1H), 4.14 (d, *J* = 8.6 Hz, 1H), 2.19 (s, 3H), 1.28 (s, 3H), 1.21 (d, *J* = 15.5 Hz, 2H), 1.12 (s, 6H), 1.11 (s, 6H). $^{13}$C NMR (101 MHz, $CDCl_3$) δ 156.86, 137.27, 129.44, 127.99, 123.14, 108.93, 84.21, 83.10, 43.14, 27.83, 24.76, 24.67, 20.82. MS (EI): m/z (%) = 289 (M+H$^+$, 3.42), 147 (100); HRMS calculated for $[C_{17}H_{26}BO_3]^+$: 289.1970 found: 289.1965. Enantiomeric excess was determined by HPLC with a Chiralpak IC column (hexanes : 2-propanol = 99:1, 0.5 mL/min, 220 nm); minor enantiomer tr = 11.2 min, major enantiomer tr = 15.5 min. $[\alpha]_D^{25}$ = 32.8 (c = 0.4, $CHCl_3$).

4.31 Synthesis of (*R*)-4,4,5,5-tetramethyl-2-((3-methyl-5-phenyl-2,3-dihydrobenzofuran-3-yl)methyl)-1,3,2-dioxaborolane (**4d**).

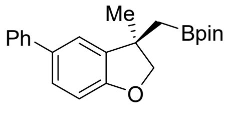


**4d**

Prepared according to typical procedure at 80 °C for 12 h by using $Pd_2(dba)_3$•$CHCl_3$ (2.5 mol%), ***N*-Me-Xu3** (7.5 mol%) from allyl ether **3d** (105.1 mg, 0.3 mmol) , $B_2Pin_2$ (83.8 mg, 0.33 mmol), $Cs_2CO_3$ (195.5 mg, 0.6 mmol) and $H_2O$ (22 μL, 1.2 mmol), after a flash column chromatography (hexanes: EA = 100:1) afforded the product **4d** as a white soild (97.7 mg, 93% yield) with 95% *ee*. $^1$H NMR (400 MHz, $CDCl_3$) δ 7.48-7.41 (m, 2H), 7.35-7.27 (m, 3H), 7.26-7.21 (m, 1H), 7.18 (t, *J* = 7.4 Hz, 1H), 6.73 (d, *J* = 8.3 Hz, 1H), 4.39 (d, *J* = 8.6 Hz, 1H), 4.22 (d, *J* = 8.6 Hz, 1H), 1.33 (s, 3H), 1.26 (d, *J* = 15.5 Hz, 1H), 1.18 (d, *J* = 15.2 Hz, 1H), 1.09 (s, 6H), 1.08 (s, 6H). $^{13}$C NMR (101 MHz, $CDCl_3$) δ 158.80, 141.56, 137.88, 133.88, 128.57, 126.77, 126.70, 126.31, 121.64, 109.54, 84.61, 83.16, 43.19, 28.22, 24.73. MS (EI): m/z (%) = 350 (M$^+$, 58.53), 209 (100); HRMS calculated for $[C_{22}H_{27}BO_3Na]^+$: 373.1945 found: 373.1938. Enantiomeric excess was determined by HPLC with a Chiralpak IC column (hexanes : 2-propanol = 99:1, 0.5 mL/min, 220 nm); minor enantiomer tr = 11.2 min, major enantiomer tr = 12.9 min. $[\alpha]_D^{25}$ = -65.5 (c = 0.4, $CHCl_3$).

4.32 Synthesis of (*R*)-4,4,5,5-tetramethyl-2-((3-methyl-5-(2-phenylpropan-2-yl)-2,3-dihydrobenzofuran-3-yl)methyl)-1,3,2-dioxaborolane (**4e**).

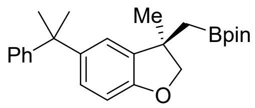


**4e**

Prepared according to typical procedure at 80 °C for 12 h by using $Pd_2(dba)_3$•$CHCl_3$ (2.5 mol%), ***N*-Me-Xu3** (7.5 mol%) from allyl ether **3e** (117.6 mg, 0.3 mmol) , $B_2Pin_2$ (83.8 mg, 0.33 mmol), $Cs_2CO_3$ (195.5 mg, 0.6 mmol) and $H_2O$ (22 μL, 1.2 mmol), after a flash column chromatography (hexanes: EA = 100:1) afforded the product **4e** as a colorless ropy liquid (113.1 mg, 96% yield) with 92% *ee*. $^1$H NMR (400 MHz, $CDCl_3$) δ 7.19-7.11 (m, 4H), 7.10-7.01 (m, 1H), 6.97 (d, *J* = 2.1 Hz, 1H), 6.88-6.80 (m, 1H), 6.55 (d, *J* = 8.3 Hz, 1H), 4.31 (d, *J* = 8.6 Hz, 1H), 4.15 (d, *J* = 8.5 Hz, 1H), 1.57 (s, 6H), 1.25 (s, 3H), 1.15 (t, *J* = 16.1 Hz, 2H), 1.09 (s, 6H), 1.08 (s, 6H). $^{13}$C NMR (101 MHz, $CDCl_3$) δ 156.90, 151.19, 142.80, 136.75, 127.81, 126.66, 126.27, 125.36, 120.94, 108.48, 84.47, 83.06, 43.22, 42.57, 31.14, 27.95, 24.76, 24.70. MS (EI): m/z (%) = 392 ($M^+$, 58.25), 377 (100); HRMS calculated for $[C_{25}H_{33}BO_3]^+$: 392.2523 found: 392.2518. Enantiomeric excess was determined by HPLC with a Chiralpak AD-H column (hexanes : 2-propanol = 99:1, 0.5 mL/min, 220 nm); minor enantiomer tr = 10.9 min, major enantiomer tr = 9.5 min. $[\alpha]_D^{20}$ = 23.5 (c = 0.4, acetone).

4.33 Synthesis of (*R*)-2-((5-(tert-butyl)-3-methyl-2,3-dihydrobenzofuran-3-yl)methyl)-4,4,5,5-tetramethyl-1,3,2-dioxaborolane (**4f**).

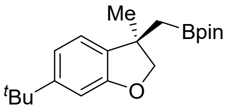


**4f**

Prepared according to typical procedure at 80 °C for 12 h by using $Pd_2(dba)_3$•$CHCl_3$ (2.5 mol%), ***N*-Me-Xu3** (7.5 mol%) from allyl ether **3f** (99.1 mg, 0.3 mmol) , $B_2Pin_2$ (83.8 mg, 0.33 mmol), $Cs_2CO_3$ (195.5 mg, 0.6 mmol) and $H_2O$ (22 μL, 1.2 mmol), after a flash column chromatography (hexanes: EA = 100:1) afforded the product **4f** as a colorless ropy liquid (90.2 mg, 91% yield) with 90% *ee*. $^1$H NMR (400 MHz, $CDCl_3$) δ 7.12 (d, *J* = 2.1 Hz, 1H), 7.06-7.00 (m, 1H), 6.60 (d, *J* = 8.3 Hz, 1H), 4.32 (d, *J* = 8.5 Hz, 1H), 4.16 (d, *J* = 8.5 Hz, 1H), 1.29 (s, 3H), 1.26 (d, *J* = 2.8 Hz, 1H), 1.21 (s, 9H), 1.16 (s, 1H), 1.12 (s, 6H), 1.10 (s, 6H). $^{13}$C NMR (101 MHz, $CDCl_3$) δ 156.79, 143.25, 136.72, 124.40, 119.55, 108.49, 84.48, 83.08, 43.27, 34.33, 31.75, 28.07, 24.78, 24.72. MS (EI): m/z (%) = 331 ($M+H^+$, 9.23), 315 (100); HRMS calculated for $[C_{20}H_{32}BO_3]^+$: 331.2439 found: 331.2431. Enantiomeric excess was determined by HPLC with a Chiralpak IC column (hexanes : 2-propanol = 99:1, 0.5 mL/min, 220 nm); minor enantiomer tr = 8.8 min, major enantiomer tr = 9.2 min. $[\alpha]_D^{25}$ = 31.8 (c = 0.4, $CHCl_3$).

4.34 Synthesis of (*R*)-4,4,5,5-tetramethyl-2-((3-methyl-2,3-dihydronaphtho[2,3-*b*]furan-3-yl)methyl)-1,3,2-dioxaborolane (**4g**).

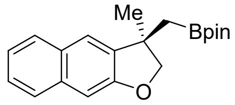


**4g**

Prepared according to typical procedure at 80 °C for 12 h by using $Pd_2(dba)_3$•$CHCl_3$ (2.5 mol%),

***N*-Me-Xu3** (7.5 mol%) from allyl ether **3g** (97.3 mg, 0.3 mmol) , $B_2Pin_2$ (83.8 mg, 0.33 mmol), $Cs_2CO_3$ (195.5 mg, 0.6 mmol) and $H_2O$ (22 μL, 1.2 mmol), after a flash column chromatography (hexanes: EA = 100:1) afforded the product **4g** as a white soild (76.1 mg, 78% yield) with 93% *ee*. $^1H$ NMR (400 MHz, $CDCl_3$) δ 7.66-7.54 (m, 2H), 7.48 (s, 1H), 7.26 (d, J = 6.9 Hz, 1H), 7.20-7.14 (m, 1H), 6.98 (s, 1H), 4.40 (d, J = 8.5 Hz, 1H), 4.24 (d, J = 8.5 Hz, 1H), 1.37 (d, J = 1.4 Hz, 3H), 1.35-1.19 (m, 2H), 1.10 (s, 6H), 1.08 (s, 6H). $^{13}C$ NMR (101 MHz, $CDCl_3$) δ 158.14, 140.29, 134.34, 129.63, 127.68, 126.66, 125.46, 122.94, 121.27, 103.71, 84.35, 83.23, 42.77, 27.98, 24.78, 24.69. MS (EI): m/z (%) = 325 ($M+H^+$, 11.31), 183 (100); HRMS calculated for $[C_{20}H_{26}BO_3]^+$: 325.1970 found: 325.1963. Enantiomeric excess was determined by HPLC with a Chiralpak IC column (hexanes : 2-propanol = 99:1, 0.5 mL/min, 220 nm); minor enantiomer tr = 12.0 min, major enantiomer tr = 14.5 min. $[\alpha]_D^{25}$ = 46.8 (c = 0.4, $CHCl_3$).

4.35 Synthesis of (*R*)-1-methyl-6-(phenylsulfonyl)-1-((4,4,5,5-tetramethyl-1,3,2-dioxaborolan-2-yl)methyl)-1,6-dihydro-2*H*-furo[3,2-e]indole (**4h**).

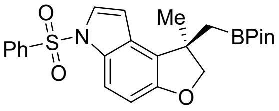


**4h**

Prepared according to typical procedure at 80 °C for 12 h by using $Pd_2(dba)_3$•$CHCl_3$ (2.5 mol%), ***N*-Me-Xu3** (7.5 mol%) from allyl ether **3h** (136 mg, 0.3 mmol) , $B_2Pin_2$ (83.8 mg, 0.33 mmol), $Cs_2CO_3$ (195.5 mg, 0.6 mmol) and $H_2O$ (22 μL, 1.2 mmol), after a flash column chromatography (hexanes: EA = 6:1) afforded the product **4h** as a white soild (98.3 mg, 73% yield) with 80% *ee*. $^1H$ NMR (400 MHz, $CDCl_3$) δ 7.86 (d, *J* = 7.5 Hz, 2H), 7.73 (d, *J* = 8.7 Hz, 1H), 7.56-7.47 (m, 2H), 7.41 (t, *J* = 7.5 Hz, 2H), 6.76 (d, *J* = 8.8 Hz, 1H), 6.66 (d, *J* = 2.7 Hz, 1H), 4.49 (d, *J* = 8.6 Hz, 1H), 4.27 (d, *J* = 8.6 Hz, 1H), 1.45 (s, 3H), 1.39 (d, *J* = 12.8 Hz, 1H), 1.32 (d, *J* = 15.6 Hz, 1H), 1.05 (s, 12H). $^{13}C$ NMR (101 MHz, $CDCl_3$) δ 155.43, 138.29, 133.58, 130.46, 129.13, 127.30, 127.13, 126.78, 126.57, 112.60, 107.37, 106.17, 83.88, 83.11, 44.21, 27.64, 24.59. ESI-MS calculated for $C_{24}H_{28}BNNaO_5S$: m/z (%): 476.1673 ($M+Na^+$), found: 476.1680. Enantiomeric excess was determined by HPLC with a Chiralpak ADH column (hexanes : 2-propanol = 80:20, 0.5 mL/min, 250 nm); major enantiomer tr = 16.7 min, minor enantiomer tr = 20.1 min. $[\alpha]_D^{20}$ = 34.1 (c = 0.3, $CHCl_3$).

4.36 Synthesis of (*S*)-4,4,5,5-tetramethyl-2-((4-methylchroman-4-yl)methyl)-1,3,2-dioxaborolane (**4i**).

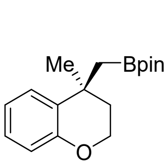


**4i**

Prepared according to typical procedure at 80 °C for 12 h by using $Pd_2(dba)_3$•$CHCl_3$ (2.5 mol%), ***N*-Me-Xu3** (7.5 mol%) from allyl ether **3i** (86.4 mg, 0.3 mmol) , $B_2Pin_2$ (83.8 mg, 0.33 mmol), $Cs_2CO_3$ (195.5 mg, 0.6 mmol) and $H_2O$ (22 μL, 1.2 mmol), after a flash column chromatography (hexanes: EA = 20:1) afforded the product **4i** as a colorless oil (63.2 mg, 73% yield) with 85% *ee*. $^1H$ NMR (400 MHz, $CDCl_3$) δ 7.29 (dd, *J* = 7.8, 1.6 Hz, 1 H), 7.03 (ddd, *J* = 8.2, 7.3, 1.6 Hz, 1 H), 6.85 (td, *J* = 7.7, 1.3 Hz, 1 H), 6.75 (dd, *J* = 8.1, 1.2 Hz, 1 H), 4.20 (dddd, *J* = 20.1, 13.9, 7.6, 3.2 Hz, 2 H), 2.25 (ddd, *J* = 13.6, 9.0, 3.6 Hz, 1 H), 1.80 (ddd, *J* = 13.9, 6.1, 2.9 Hz, 1 H), 1.40 (s, 3

H), 1.30 (s, 2 H), 1.16 (s, 6H), 1.12 (s, 6H). $^{13}C$ NMR (101 MHz, $CDCl_3$) δ 153.52, 132.53, 127.19, 126.79, 120.18, 116.63, 82.88, 63.05, 36.37, 32.06, 31.58, 24.72, 24.59. MS (EI): m/z (%) = 288 ($M+H^+$, 18.61), 147 (100); HRMS calculated for $[C_{17}H_{26}BO_3]^+$: 288.1897 found: 288.1902. Enantiomeric excess was determined by HPLC with a Chiralpak AD-3 column (hexanes : 2-propanol = 99.5:0.5, 0.3 mL/min, 254 nm); minor enantiomer tr = 18.2 min, major enantiomer tr = 19.4 min. $[\alpha]_D^{20}$ = 14.8 (c = 0.4, acetone).

4.37 Synthesis of (*S*)-4,4,5,5-tetramethyl-2-((1-methyl-2,3-dihydro-1H-inden-1-yl)methyl)-1,3,2-dioxaborolane (**4j**).

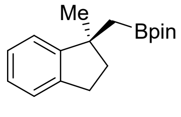


**4j**

Prepared according to typical procedure at 80 °C for 12 h by using $Pd_2(dba)_3$•$CHCl_3$ (2.5 mol%), ***N*-Me-Xu3** (7.5 mol%) from allyl ether **3j** (81.6 mg, 0.3 mmol) , $B_2Pin_2$ (83.8 mg, 0.33 mmol), $Cs_2CO_3$ (195.5 mg, 0.6 mmol) and $H_2O$ (22 μL, 1.2 mmol), after a flash column chromatography (hexanes: EA = 20:1) afforded the product **4j** as a colorless oil (62.1 mg, 77% yield) with 89% *ee*. $^1H$ NMR (400 MHz, $CDCl_3$) δ 7.30-7.02 (m, 4 H), 2.88 (td, *J* = 7.2, 3.2 Hz, 2 H), 2.21-2.06 (m, 1 H), 1.94 (ddd, *J* = 12.5, 7.6, 6.2 Hz, 1 H), 1.31 (s, 3 H), 1.26 (d, *J* = 14.9 Hz, 1 H), 1.19 (s, 6H), 1.18 (s, 6H), 1.11 (d, *J* = 14.9 Hz, 1 H). $^{13}C$ NMR (101 MHz, $CDCl_3$) δ 153.36, 142.44, 126.12, 125.96, 124.26, 122.29, 82.80, 45.32, 40.76, 30.08, 28.68, 24.81, 24.74. MS (EI): m/z (%) = 272 ($M+H^+$, 4.49), 131 (100); HRMS calculated for $[C_{17}H_{26}BO_2]^+$: 272.1948 found: 272.1950. Enantiomeric excess was determined by HPLC with a Chiralpak AD-3 column (hexanes : 2-propanol = 99.5:0.5, 0.3 mL/min, 254 nm); minor enantiomer tr = 12.3 min, major enantiomer tr = 13.9 min. $[\alpha]_D^{20}$ = 23.0 (c = 0.4, acetone).

4.38 Synthesis of (*R*)-1-(3-methyl-3-((4,4,5,5-tetramethyl-1,3,2-dioxaborolan-2-yl)methyl)indolin-1-yl)ethan-1-one (**4k**).

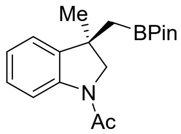


**4k**

Prepared according to typical procedure at 80 °C for 12 h by using $Pd_2(dba)_3$•$CHCl_3$ (2.5 mol%), ***N*-Me-Xu3** (7.5 mol%) from allyl ether **3k** (94.5 mg, 0.3 mmol) , $B_2Pin_2$ (99 mg, 0.39 mmol), $Cs_2CO_3$ (195.5 mg, 0.6 mmol) and $H_2O$ (22 μL, 1.2 mmol), after a flash column chromatography (hexanes: EA = 5:1) afforded the product **4k** as a yellow solid (87.1 mg, 92% yield) with 53% *ee*. $^1H$ NMR (400 MHz, $CDCl_3$) δ 8.15 (d, *J* = 7.9 Hz, 1H), 7.21-7.11 (m, 2H), 7.02 (t, *J* = 7.0 Hz, 1H), 4.06 (d, *J* = 10.2 Hz, 1H), 3.76 (d, *J* = 10.2 Hz, 1H), 2.22 (s, 3H), 1.39 (s, 3H), 1.26 (s, 1H), 1.24 (s, 1H), 1.11 (s, 12H). $^{13}C$ NMR (101 MHz, $CDCl_3$) δ 168.59, 141.52, 140.85, 127.47, 123.63, 122.04, 116.82, 83.14, 62.86, 41.48, 29.78, 24.70, 24.60, 24.18. ESI-MS calculated for $C_{18}H_{26}BNNaO_3$: m/z (%): 338.1898 ($M+Na^+$), found: 338.1905. Enantiomeric excess was determined by HPLC with a Chiralpak ADH column (hexanes : 2-propanol = 96:4, 0.5 mL/min, 220 nm); major enantiomer tr = 29.9 min, minor enantiomer tr = 33.6 min. $[\alpha]_D^{20}$ = -1.1 (c = 0.3, $CHCl_3$).

## 5. Synthetic Transformations

5.1 Synthesis of (*S*)-(3-methyl-5-phenyl-2,3-dihydrobenzofuran-3-yl)methanol (**5**).

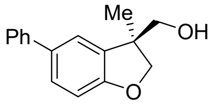

**5**

The synthesis was conducted according to a modified literature procedure.[9] An aqueous hydrogen peroxide solution (0.4 mL, 30% w/w) was added dropwise to a solution of **4d** (105 mg, 0.3 mmol) in THF and aqueous sodium phosphate monobasic (1.5 mL, 0.75 mmol, 0.5 M) at 0 °C. The mixture was stirred at 0 °C for 30 min and at room temperature for 1.5 h. Upon the completion of the reaction as determined by TLC, the mixture was cooled to 0 °C and quenched with water. The aqueous layer was extracted with EtOAc (3 x 5 mL). The combined organic layers were dried over magnesium sulfate, filtered and concentrated. The crude product was purified by column chromatography on silica gel (hexanes: EA = 5:1) to provide **5** as a colorless oil (45.4 mg, 99%) with 94% *ee.* $^{1}$H NMR (400 MHz, $CDCl_3$) δ 7.43 (d, *J* = 7.2 Hz, 2H), 7.36-7.26 (m, 3H), 7.25-7.16 (m, 2H), 6.77 (d, *J* = 8.3 Hz, 1H), 4.51 (d, *J* = 8.9 Hz, 1H), 4.12 (d, *J* = 8.8 Hz, 1H), 3.58 (d, *J* = 10.8 Hz, 1H), 3.49 (d, *J* = 10.9 Hz, 1H), 1.30 (s, 3H). $^{13}$C NMR (101 MHz, $CDCl_3$) δ 159.86, 141.08, 134.18, 132.53, 128.68, 127.81, 126.73, 126.59, 121.88, 109.93, 80.51, 68.91, 47.62, 21.82. ESI-MS calculated for $C_{16}H_{16}NaO_2$: m/z (%): 263.1042 ($M+Na^+$), found: 263.1043. Enantiomeric excess was determined by HPLC with a Chiralpak IC column (hexanes : 2-propanol = 95:5, 0.5 mL/min, 220 nm); minor enantiomer tr = 16.3 min, major enantiomer tr = 17.8 min. $[\alpha]_D^{20}$ = 29.2 (c = 0.4, acetone).

5.2 Synthesis of (*R*)-trifluoro((3-methyl-5-phenyl-2,3-dihydrobenzofuran-3-yl)methyl)-$\lambda^4$-borane, potassium salt (**6**).

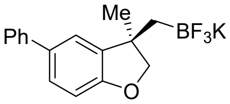

**6**

The synthesis was conducted according to a modified literature procedure.[10] The boronic ester **4d** (140 mg, 0.4 mmol) was dissolved in acetonitrile (2.5 mL) and saturated aqueous $KHF_2$ (4.5 M, 0.45 mL, 5.1 equiv) was added. The reaction mixture was stirred at room temperature for 3 h, concentrated, azeotroped with methanol, and placed on the vacuum for 3 h. The crude product was dissolved in hot acetone, filtered, and Then the solvent was removed under vacuum and the crude product was recrystallized from acetone to give the desired product **6** as a white solid (130.4 mg, 98%). $^{1}$H NMR (400 MHz, Acetone-*d6*) δ 7.61-7.51 (m, 2H), 7.41-7.35 (m, 3H), 7.31-7.19 (m, 2H), 6.71 (d, *J* = 8.2 Hz, 1H), 4.48 (d, *J* = 8.6 Hz, 1H), 4.23 (d, *J* = 8.6 Hz, 1H), 1.33 (s, 3H), 0.89 (dq, *J* = 13.8, 6.8 Hz, 1H), 0.63 (dq, *J* = 13.7, 6.8 Hz, 1H). $^{13}$C NMR (101 MHz, Acetone-*d6*) δ 159.87, 143.44, 142.75, 133.96, 129.47, 127.26, 126.91, 126.26, 122.11, 109.74, 109.65, 86.01, 44.91 (d, *J* = 1.9 Hz), 28.30. $^{19}$F NMR (376 MHz, Acetone-*d6*) δ -135.50. ESI-MS calculated for $C_{16}H_{15}BF_3KNaO$: m/z (%):353.0700 ($M+Na^+$), found: 353.0691. $[\alpha]_D^{20}$ = 33.2 (c = 0.4, acetone).

5.3 Synthesis of (*R*)-3-allyl-3-methyl-5-phenyl-2,3-dihydrobenzofuran (**7**).

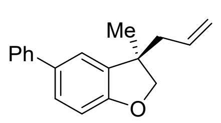

**7**

The synthesis was conducted according to a modified literature procedure.[11] The boronic ester **4d** (140 mg, 0.4 mmol) was dissolved in THF (2.0 mL) under argon. The mixture was cooled to -78 °C, vinyl magnesium bromide (1.0 M in THF, 1.2 mL, 1.2 mmol) was added dropwise and the solution was stirred at room temperature for 2 h. Then a solution of $I_2$ (152 mg, 1.2 mmol) in MeOH (3.0 mL) was added dropwise at -78 °C. After 2 h, a solution of MeONa (108 mg, 2.0 mmol) in MeOH (3.0 mL) was dropwised at room temperature for 2 h. The reaction was quenched by the addition of saturated aqueous $Na_2S_2O_3$ (10 mL). The organic layer was separated and the aqueous phase extracted with EtOAc (3 x 5 mL). The combined organic layers were washed with brine (10 mL), dried over magnesium sulfate and concentrated. The crude product was purified by column chromatography on silica gel (hexanes: EA = 50:1) to give **7** as a colorless oil (50 mg, 99%) with 92% *ee*.$^1$H NMR (400 MHz, $CDCl_3$) δ 7.46 (d, *J* = 7.4 Hz, 2H), 7.36-7.26 (m, 3H), 7.24-7.18 (m, 2H), 6.77 (d, *J* = 8.2 Hz, 1H), 5.75-5.54 (m, 1H), 5.03 (s, 1H), 4.99 (d, *J* = 6.7 Hz, 1H), 4.36 (d, *J* = 8.6 Hz, 1H), 4.10 (d, *J* = 8.6 Hz, 1H), 2.33 (d, *J* = 7.3 Hz, 2H), 1.31 (s, 3H). $^{13}$C NMR (101 MHz, $CDCl_3$) δ 159.25, 141.43, 135.53, 134.08, 133.89, 128.66, 127.23, 126.81, 126.50, 121.74, 118.45, 109.75, 82.32, 45.18, 45.10, 25.17. MS (ESI): m/z (%) = 250 ($M+H^+$, 17.78); HRMS calculated for $[C_{20}H_{25}BO_3]^+$: 250.1358 found: 250.1356. Enantiomeric excess was determined by HPLC with a Chiralpak IC column (hexanes : 2-propanol = 95:5, 0.5 mL/min, 220 nm); minor enantiomer tr = 11.5 min, major enantiomer tr = 10.6 min. $[\alpha]_D^{20}$ = 85.0 (c = 0.2, acetone).

5.4 Synthesis of (*R*)-3-(furan-2-ylmethyl)-3-methyl-5-phenyl-2,3-dihydrobenzofuran (**8**).

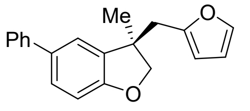

**8**

The synthesis was conducted according to a modified literature procedure.[11] *n*-BuLi (2.4 M in hexanes, 0.1 mL, 0.24 mmol) was added dropwise at -78 °C to a solution of furan (16.3 mg, 0.24 mmol) in THF (1.0 mL). The cooling bath was removed and the mixture was stirred at room temperature for 1 h. The mixture was cooled to -78 °C and boronic ester **4d** (70 mg, 0.2 mmol) was added dropwise as a solution in THF (1.0 mL). The mixture was stirred for 1 h at -78 °C and a solution of *N*-bromosuccinimide (42.7 mg, 0.24 mmol) in THF (1.0 mL) was added dropwise. After 1 h at -78 °C, a saturated aqueous solution of $Na_2S_2O_3$ was added and the reaction mixture was allowed to warm to room temperature. After addition of $Et_2O$ and water, the layers were separated and the aqueous layer was extracted with $Et_2O$ (3 x 5 mL). The combined organic layers were dried over $MgSO_4$ and concentrated in vacuo. The crude product was purified by column chromatography on silica gel (hexanes: EA = 50:1) to give **8** as a colorless oil (44 mg, 76%) with 95% *ee.* $^1$H NMR (400 MHz, $CDCl_3$) δ 7.56-7.47 (m, 2H), 7.43-7.35 (m, 3H), 7.34-7.32 (m, 1H), 7.31-7.25 (m, 1H), 7.23-7.19 (m, 1H), 6.85 (d, *J* = 8.2 Hz, 1H), 6.29 (t, *J* = 2.5 Hz, 1H), 5.97 (d, *J* = 3.1 Hz, 1H), 4.60 (d, *J* = 8.8 Hz, 1H), 4.19 (d, *J* = 8.8 Hz, 1H), 2.96 (s, 2H), 1.39 (s, 3H). $^{13}$C NMR (101 MHz, $CDCl_3$) δ 159.12, 152.50, 141.53, 141.35, 135.35, 134.06, 128.65, 127.38, 126.77, 126.51, 121.77, 110.21, 109.81, 107.88, 82.72, 45.84, 38.73, 24.82. MS (EI): m/z (%) =

290 ($M^+$, 9.31), 209 (100); HRMS calculated for $[C_{20}H_{25}BO_3]^+$: 290.1307 found: 290.1311. Enantiomeric excess was determined by HPLC with a Chiralpak IC column (hexanes : 2-propanol = 95:5, 0.5 mL/min, 220 nm); minor enantiomer tr = 9.5 min, major enantiomer tr = 8.1 min. $[\alpha]_D^{20}$ = 76.6 (c = 0.4, acetone).

# 6. X-ray structure of 4d.

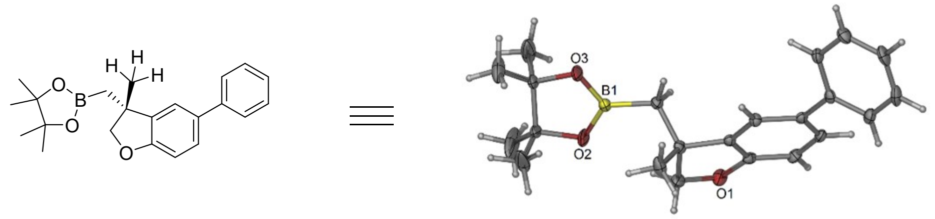


**4d**

# 7. $^1$H , $^{19}$F, $^{13}$C NMR and HPLC Spectra

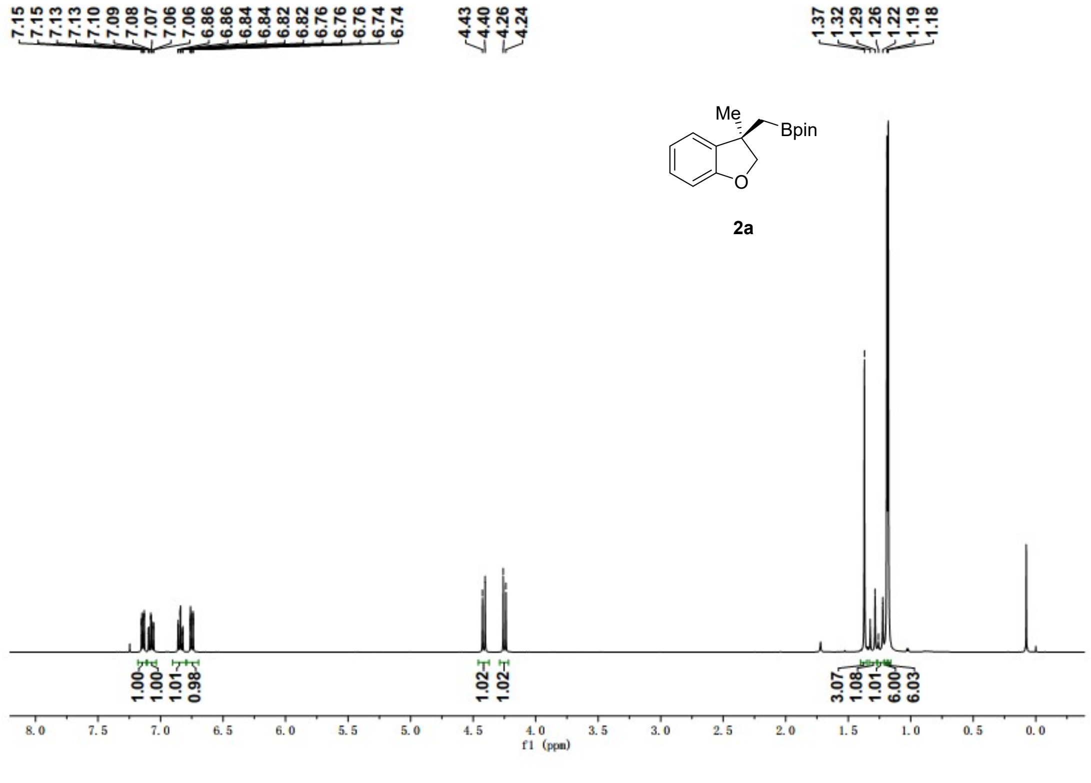

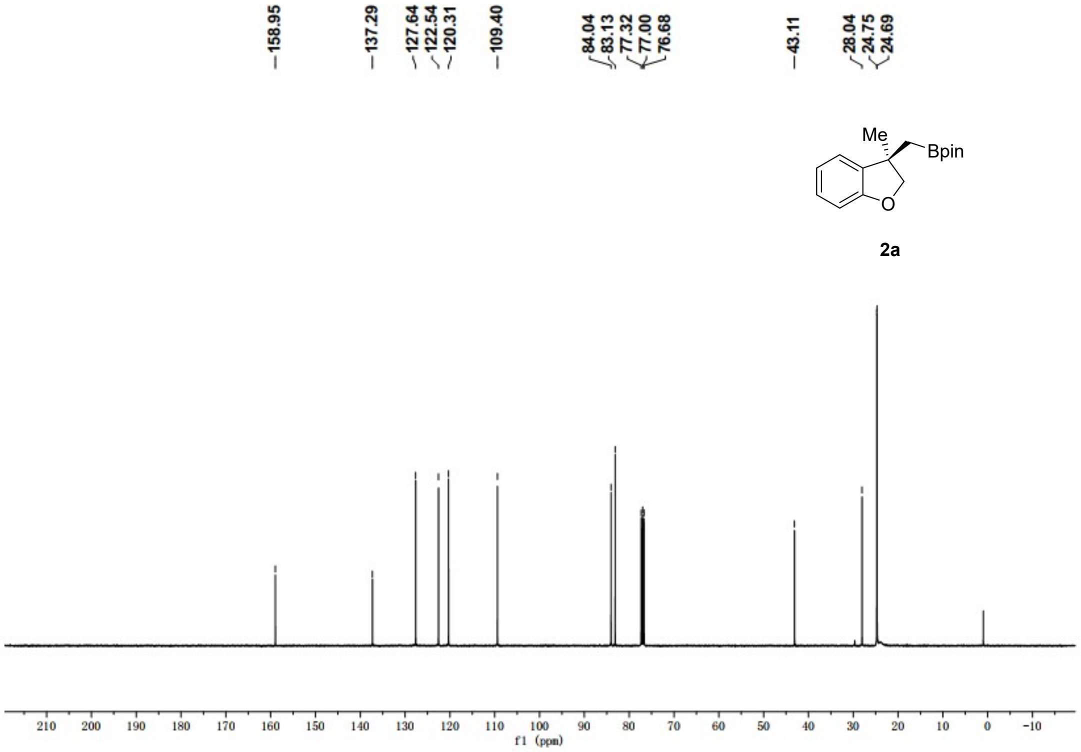


**Chromatogram**

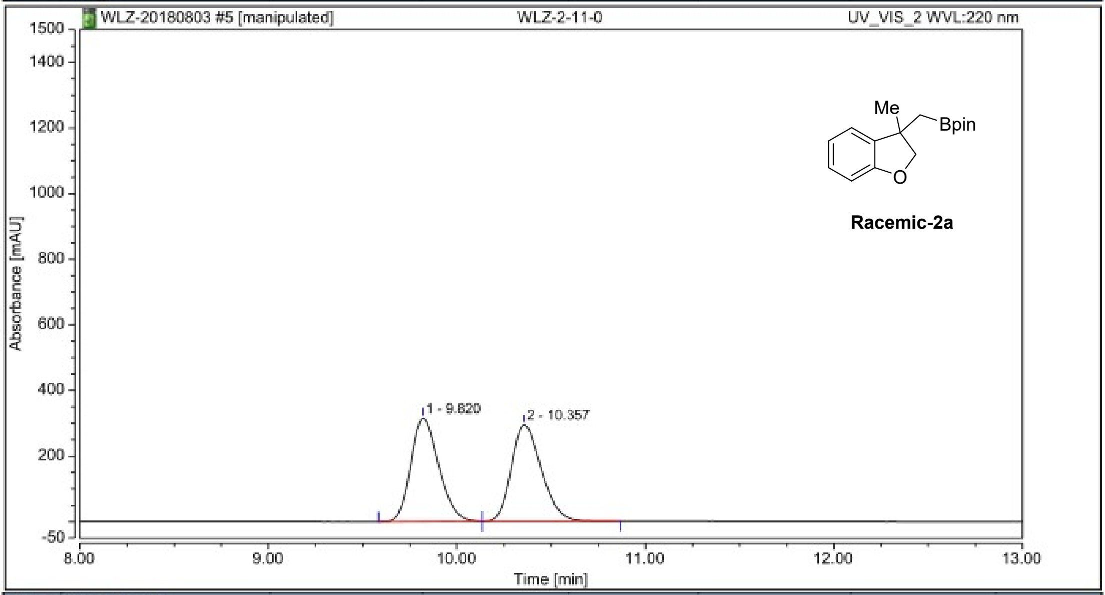


**Integration Results**

| No. | Peak Name | Retention Time min | Area mAU*min | Height mAU | Relative Area % | Relative Height % | Amount n.a. |
|---|---|---|---|---|---|---|---|
| 1 | | 9.820 | 54.440 | 315.543 | 50.01 | 51.70 | n.a. |
| 2 | | 10.357 | 54.412 | 294.762 | 49.99 | 48.30 | n.a. |
| **Total:** | | | **108.852** | **610.305** | **100.00** | **100.00** | |

## Chromatogram

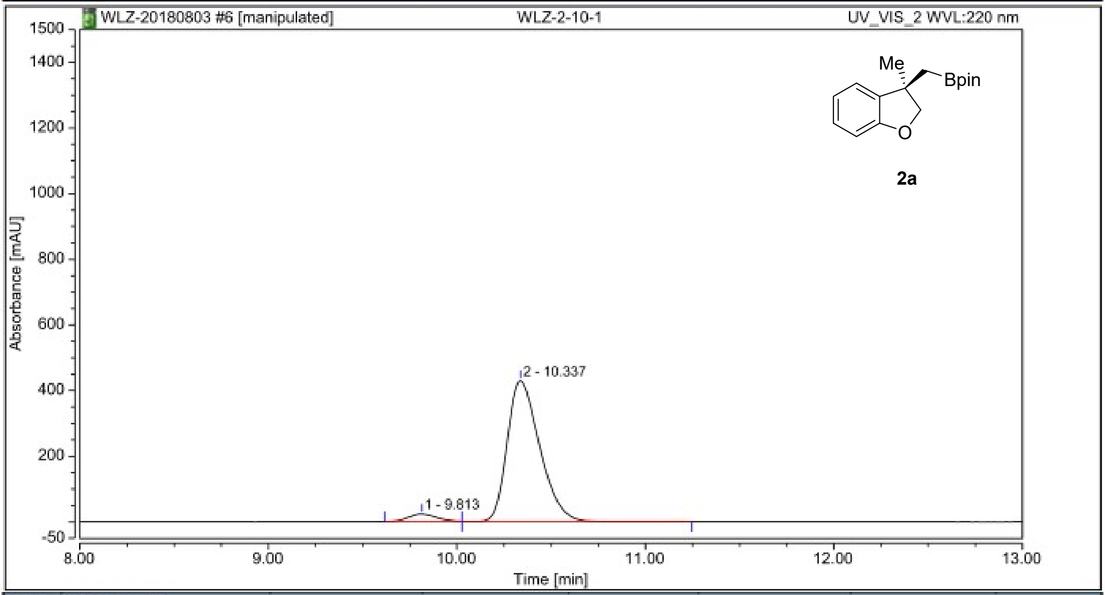


## Integration Results

| No. | Peak Name | Retention Time min | Area mAU*min | Height mAU | Relative Area % | Relative Height % | Amount n.a. |
|---|---|---|---|---|---|---|---|
| 1 | | 9.813 | 4.003 | 22.949 | 4.45 | 5.07 | n.a. |
| 2 | | 10.337 | 85.900 | 429.489 | 95.55 | 94.93 | n.a. |
| **Total:** | | | **89.903** | **452.437** | **100.00** | **100.00** | |

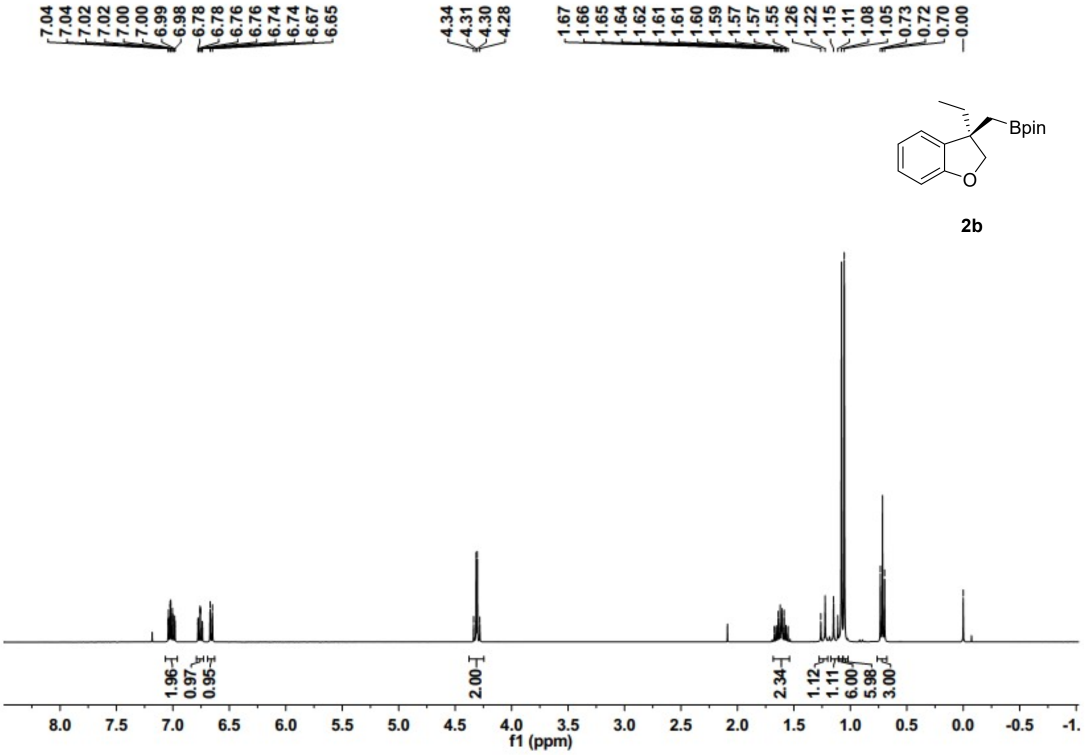


2b

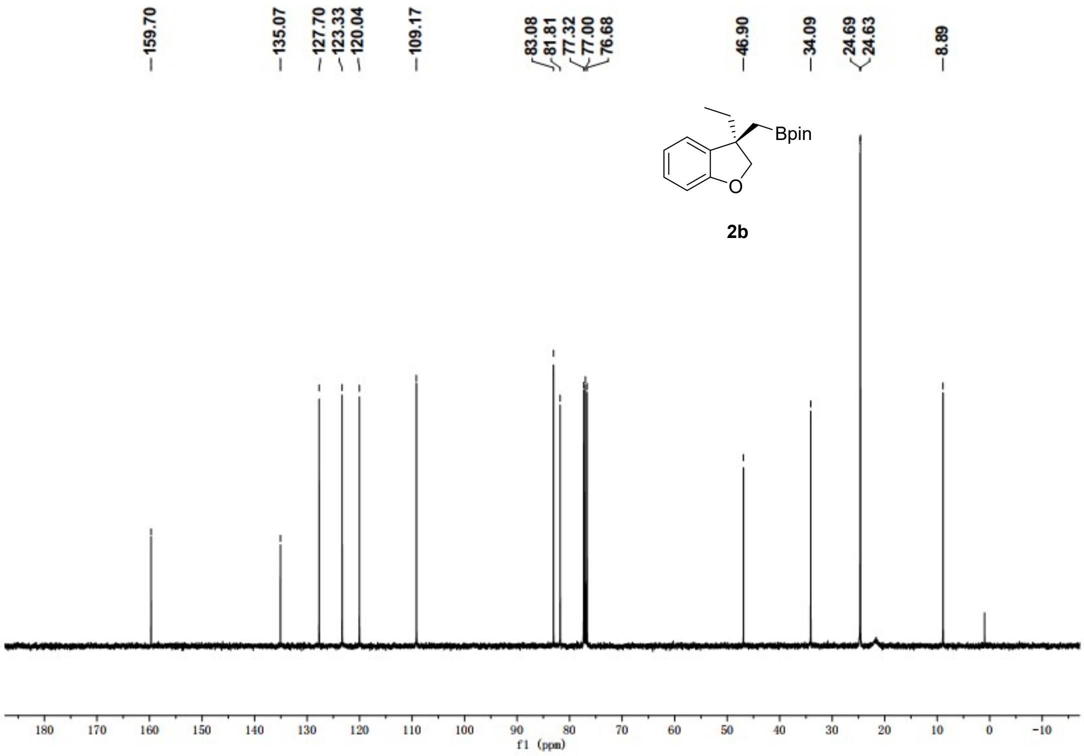


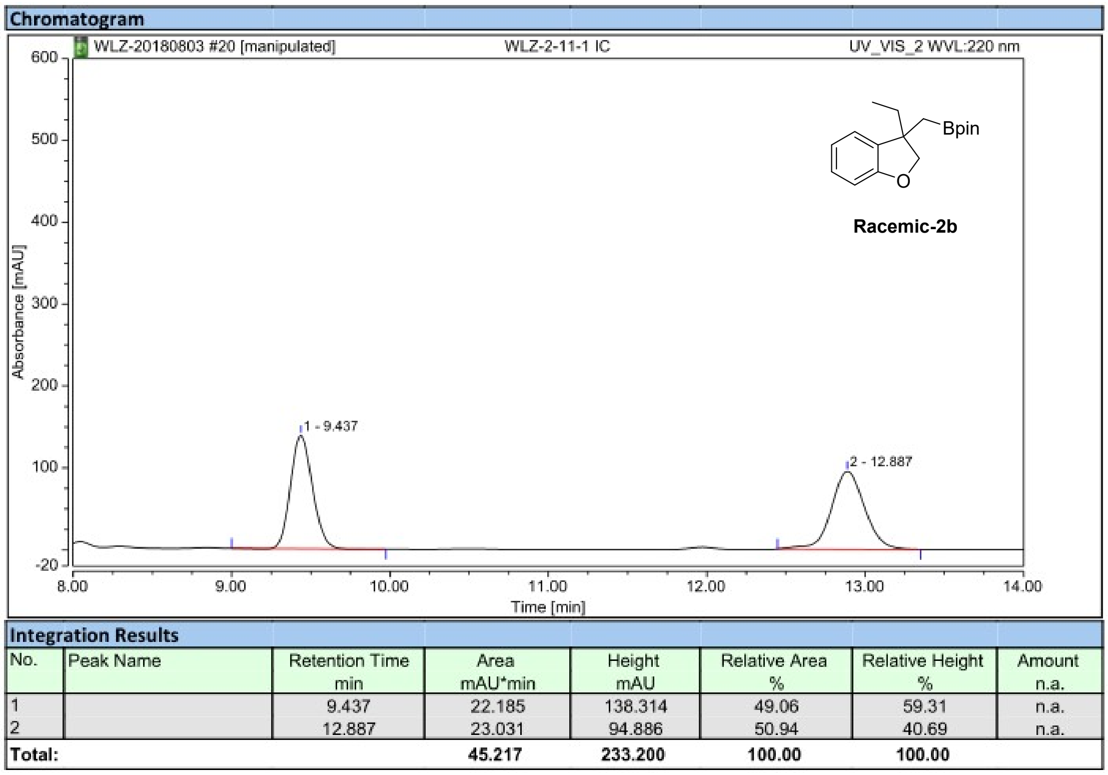


## Integration Results

| No. | Peak Name | Retention Time min | Area mAU*min | Height mAU | Relative Area % | Relative Height % | Amount n.a. |
|---|---|---|---|---|---|---|---|
| 1 | | 9.437 | 22.185 | 138.314 | 49.06 | 59.31 | n.a. |
| 2 | | 12.887 | 23.031 | 94.886 | 50.94 | 40.69 | n.a. |
| **Total:** | | | **45.217** | **233.200** | **100.00** | **100.00** | |

**Chromatogram**

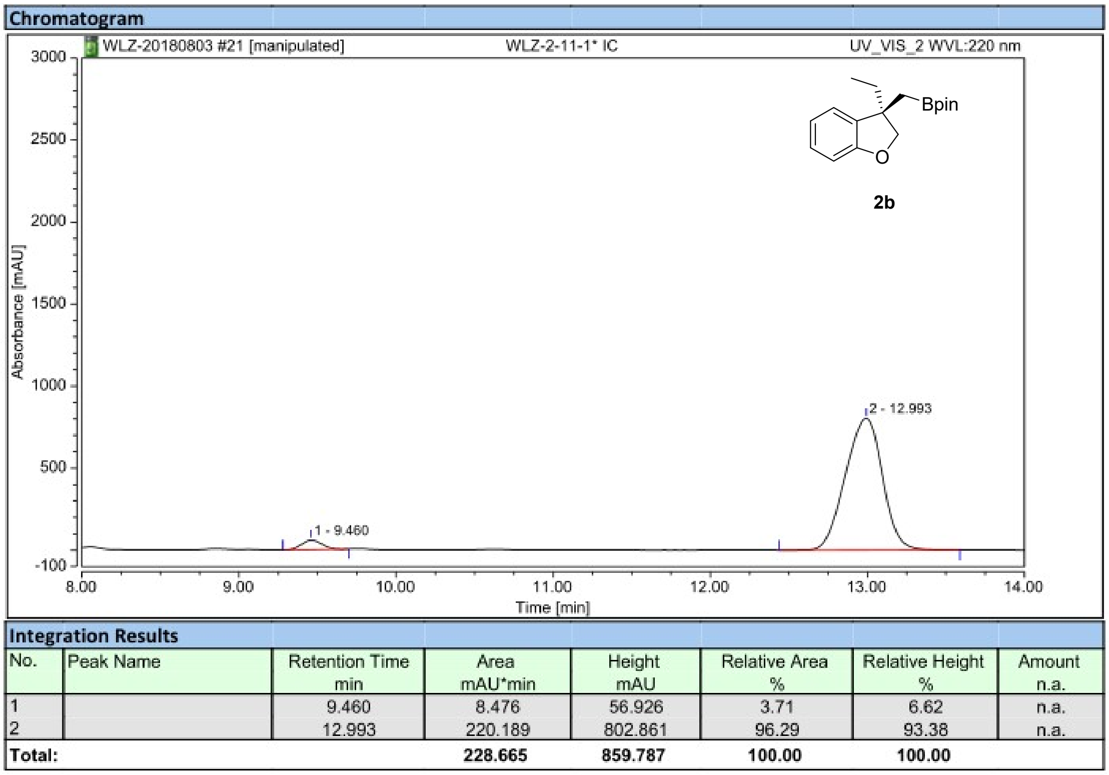


**Integration Results**

| No. | Peak Name | Retention Time min | Area mAU*min | Height mAU | Relative Area % | Relative Height % | Amount n.a. |
|---|---|---|---|---|---|---|---|
| 1 | | 9.460 | 8.476 | 56.926 | 3.71 | 6.62 | n.a. |
| 2 | | 12.993 | 220.189 | 802.861 | 96.29 | 93.38 | n.a. |
| **Total:** | | | **228.665** | **859.787** | **100.00** | **100.00** | |

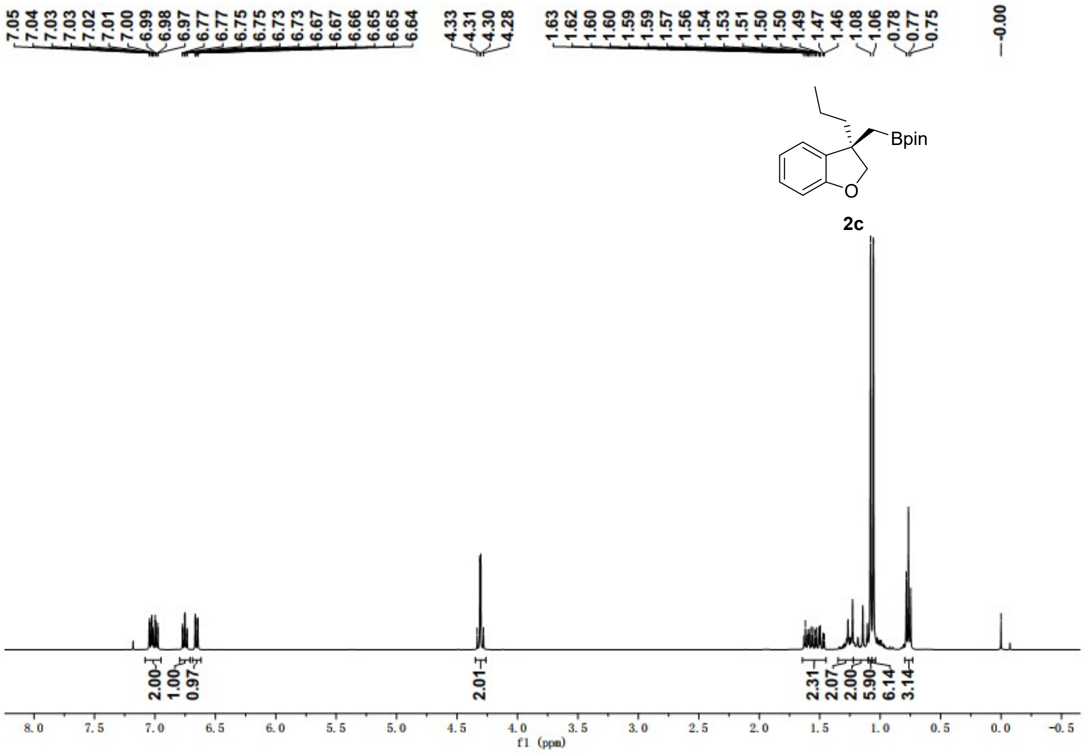

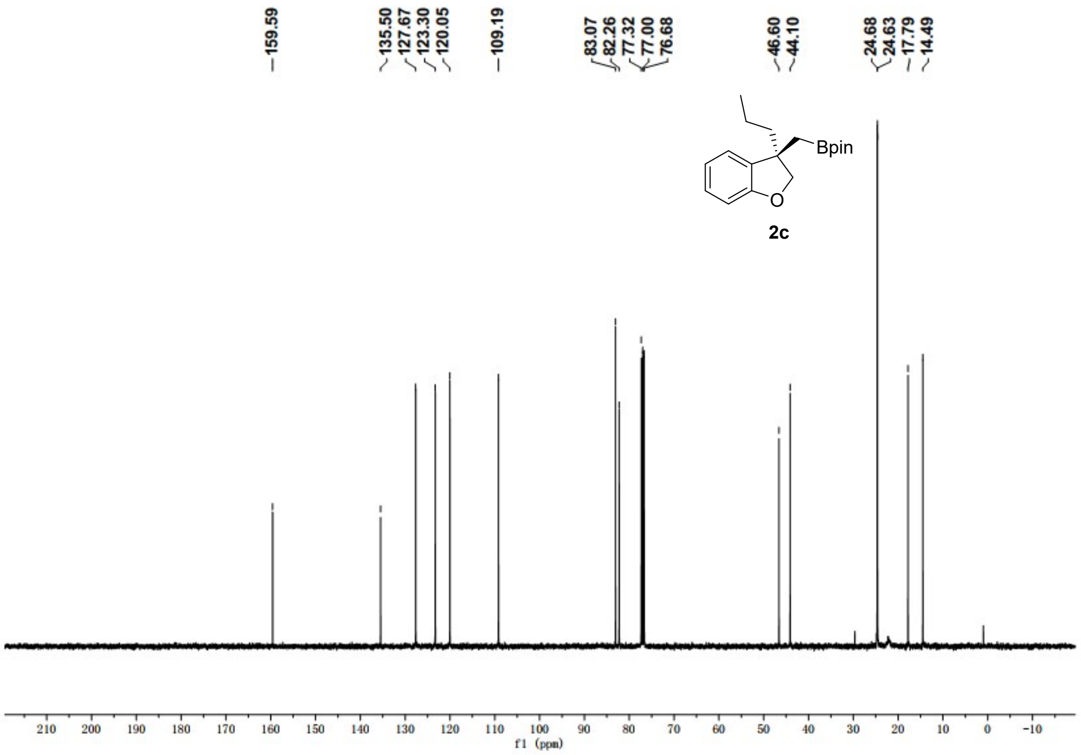


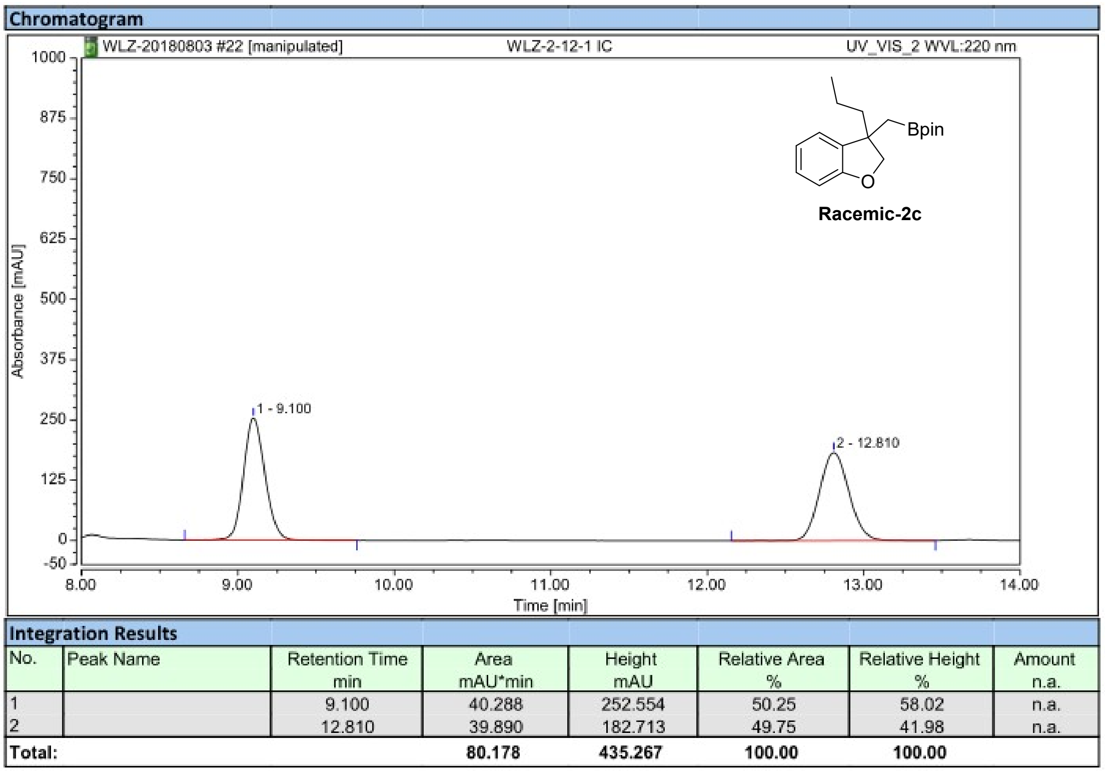


**Integration Results**

| No. | Peak Name | Retention Time<br>min | Area<br>mAU*min | Height<br>mAU | Relative Area<br>% | Relative Height<br>% | Amount<br>n.a. |
|---|---|---|---|---|---|---|---|
| 1 | | 9.100 | 40.288 | 252.554 | 50.25 | 58.02 | n.a. |
| 2 | | 12.810 | 39.890 | 182.713 | 49.75 | 41.98 | n.a. |
| **Total:** | | | **80.178** | **435.267** | **100.00** | **100.00** | |

**Chromatogram**

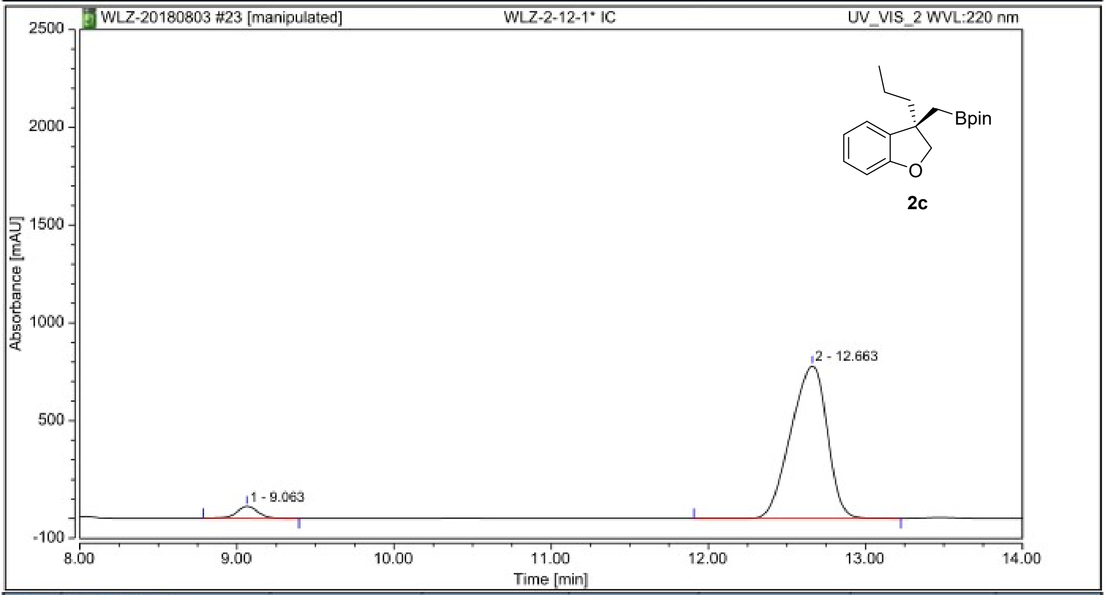


**Integration Results**

| No. | Peak Name | Retention Time min | Area mAU*min | Height mAU | Relative Area % | Relative Height % | Amount n.a. |
|---|---|---|---|---|---|---|---|
| 1 | | 9.063 | 9.922 | 62.510 | 4.51 | 7.41 | n.a. |
| 2 | | 12.663 | 209.903 | 780.820 | 95.49 | 92.59 | n.a. |
| **Total:** | | | **219.824** | **843.330** | **100.00** | **100.00** | |

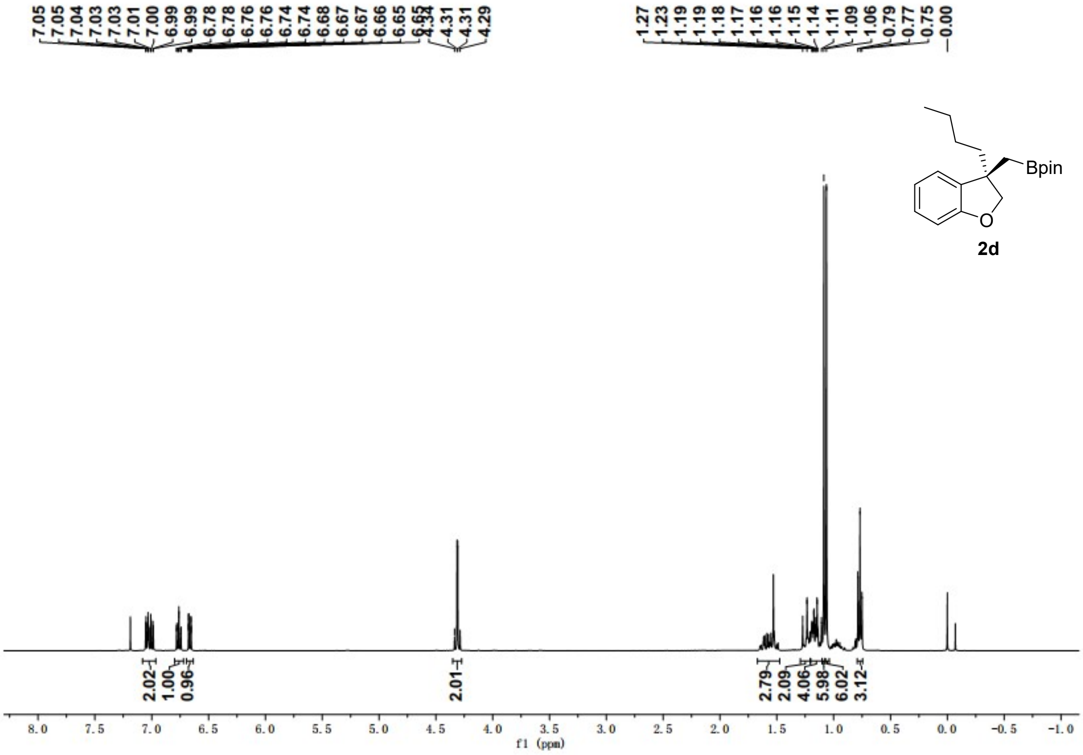

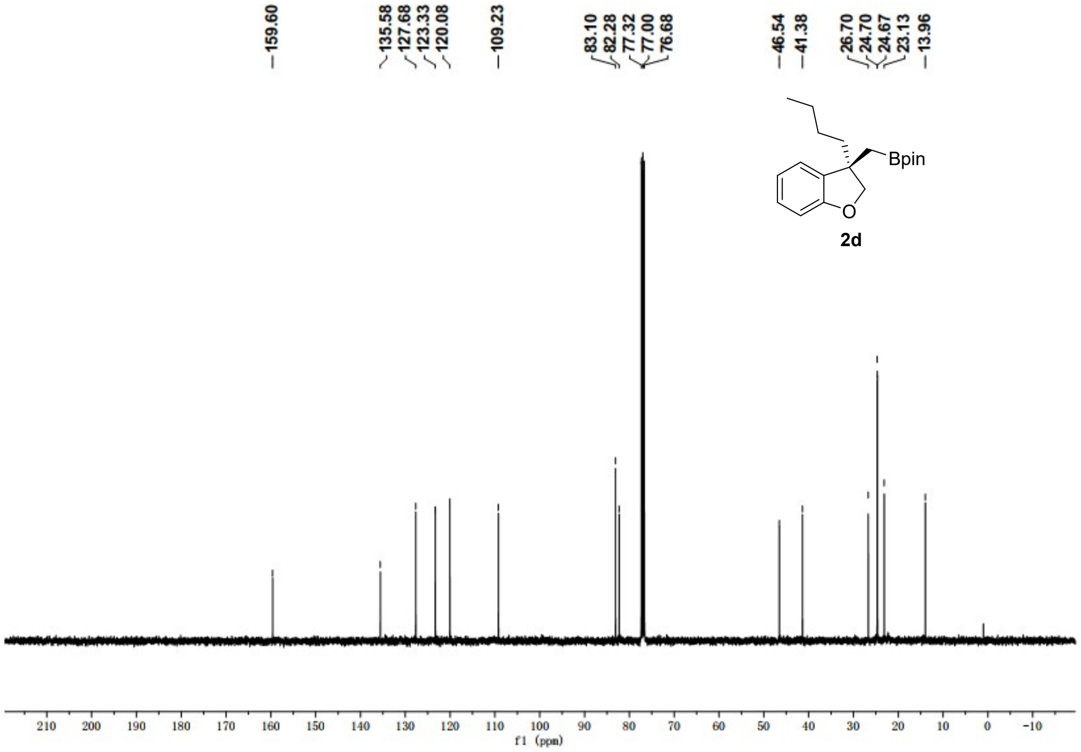


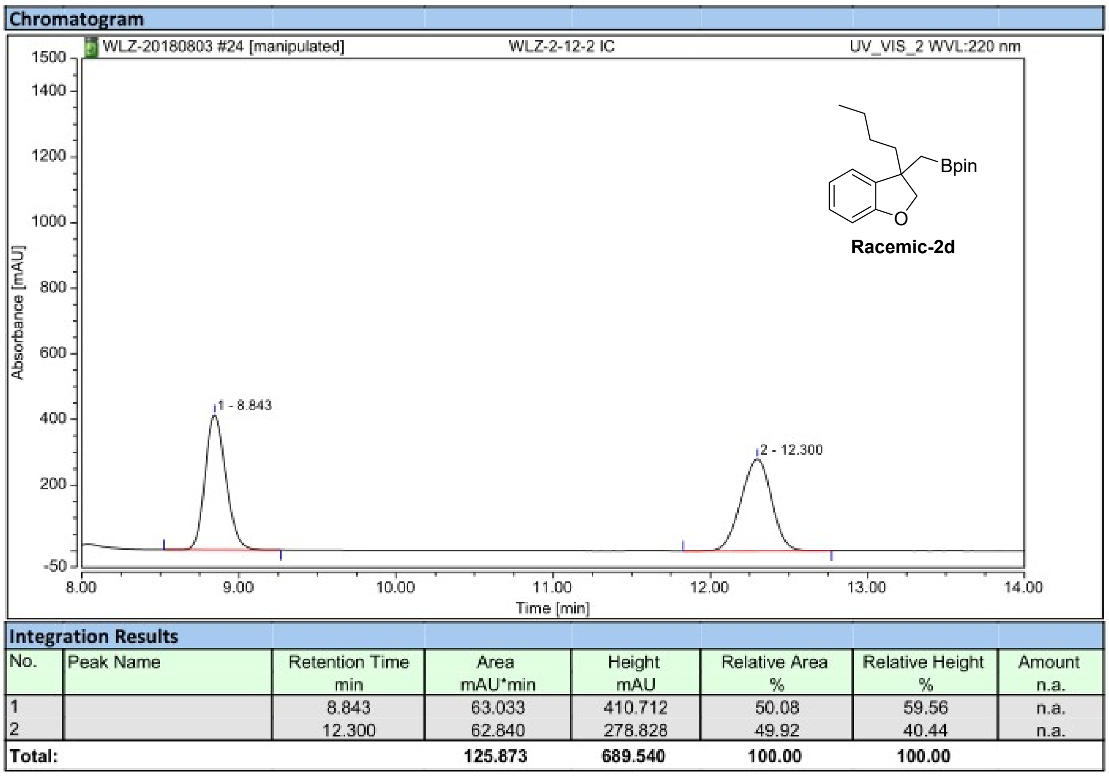


**Integration Results**

| No. | Peak Name | Retention Time min | Area mAU*min | Height mAU | Relative Area % | Relative Height % | Amount n.a. |
|---|---|---|---|---|---|---|---|
| 1 | | 8.843 | 63.033 | 410.712 | 50.08 | 59.56 | n.a. |
| 2 | | 12.300 | 62.840 | 278.828 | 49.92 | 40.44 | n.a. |
| **Total:** | | | **125.873** | **689.540** | **100.00** | **100.00** | |

## Chromatogram

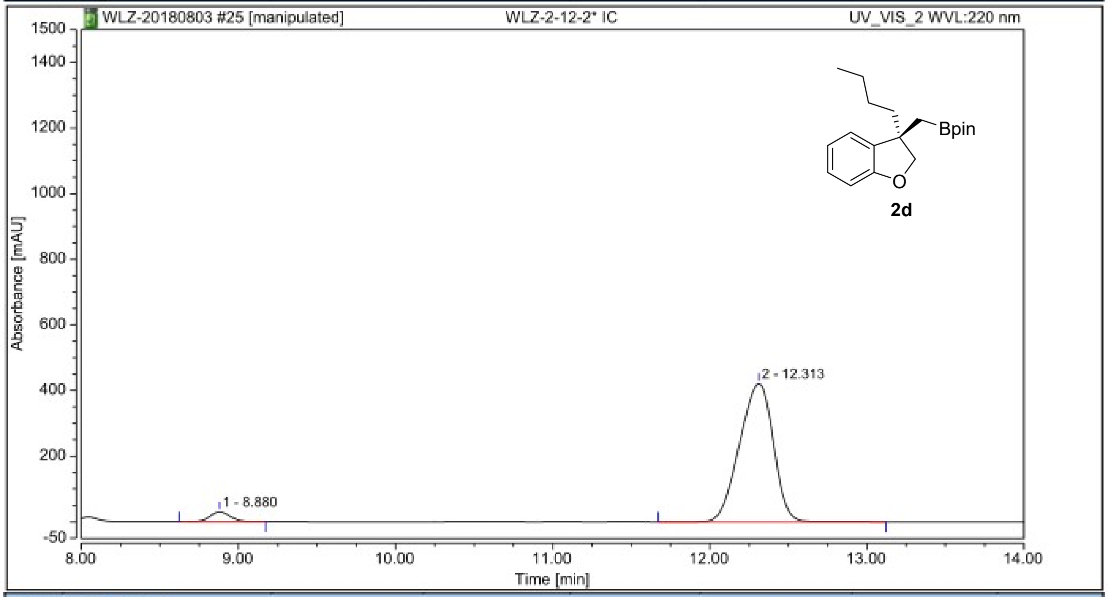


## Integration Results

| No. | Peak Name | Retention Time min | Area mAU*min | Height mAU | Relative Area % | Relative Height % | Amount n.a. |
|---|---|---|---|---|---|---|---|
| 1 | | 8.880 | 4.549 | 30.149 | 4.14 | 6.67 | n.a. |
| 2 | | 12.313 | 105.303 | 421.690 | 95.86 | 93.33 | n.a. |
| **Total:** | | | **109.852** | **451.839** | **100.00** | **100.00** | |

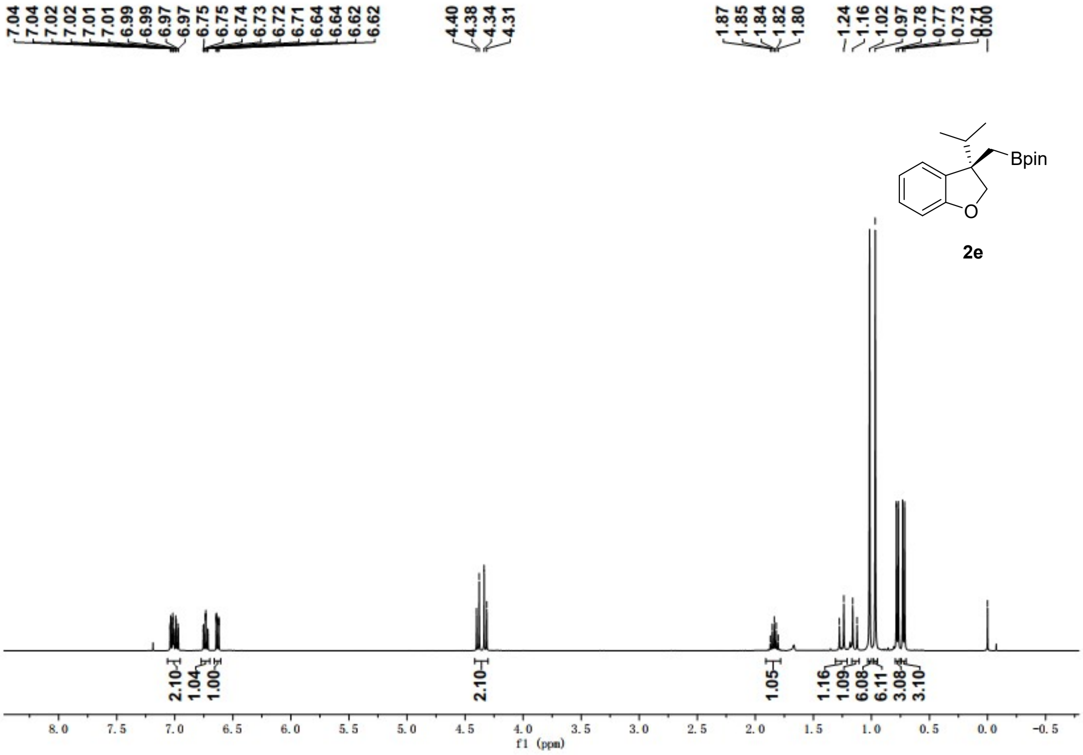

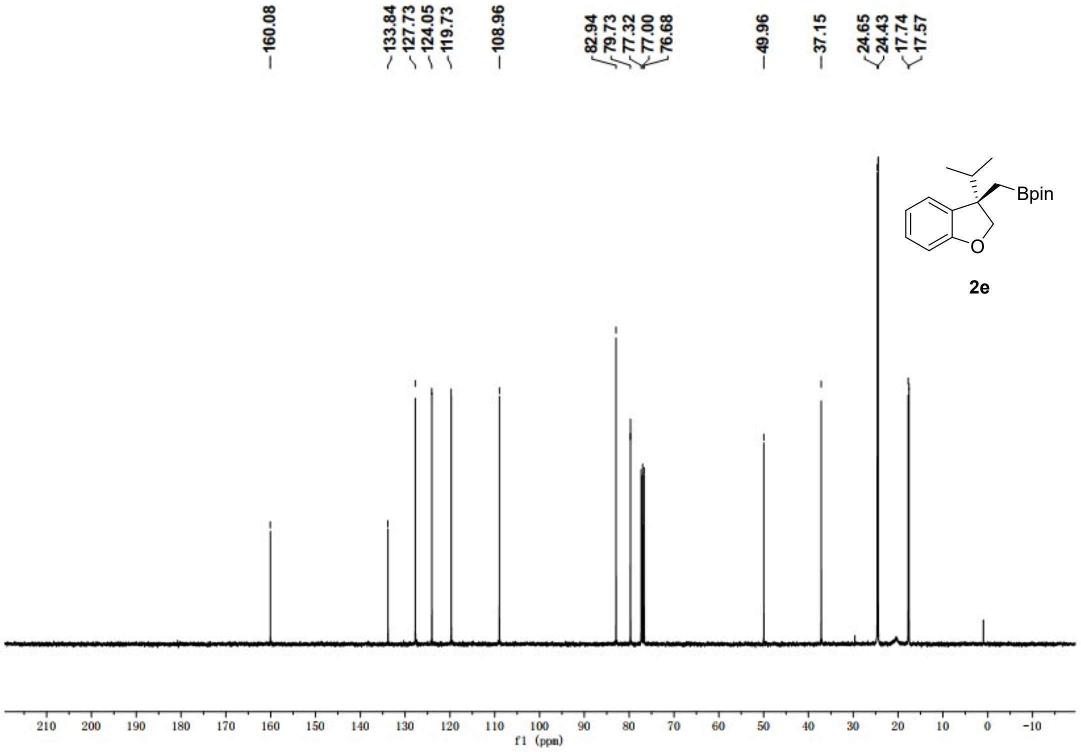


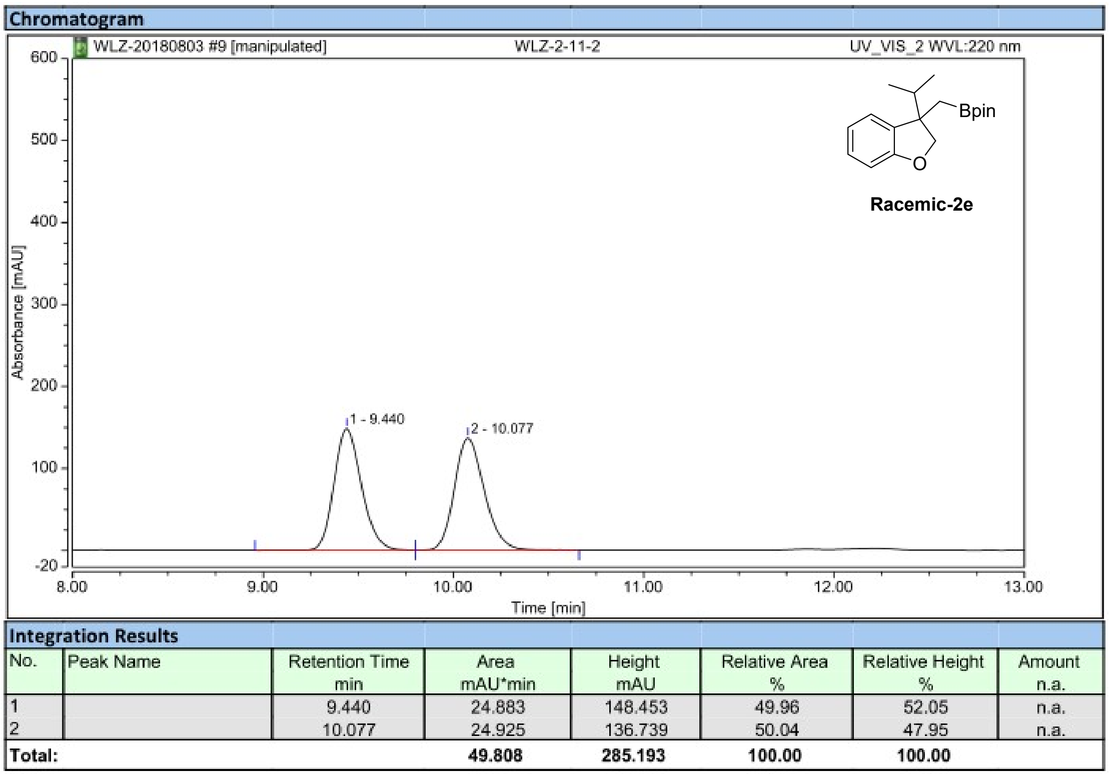


**Integration Results**

| No. | Peak Name | Retention Time min | Area mAU*min | Height mAU | Relative Area % | Relative Height % | Amount n.a. |
|---|---|---|---|---|---|---|---|
| 1 | | 9.440 | 24.883 | 148.453 | 49.96 | 52.05 | n.a. |
| 2 | | 10.077 | 24.925 | 136.739 | 50.04 | 47.95 | n.a. |
| **Total:** | | | **49.808** | **285.193** | **100.00** | **100.00** | |

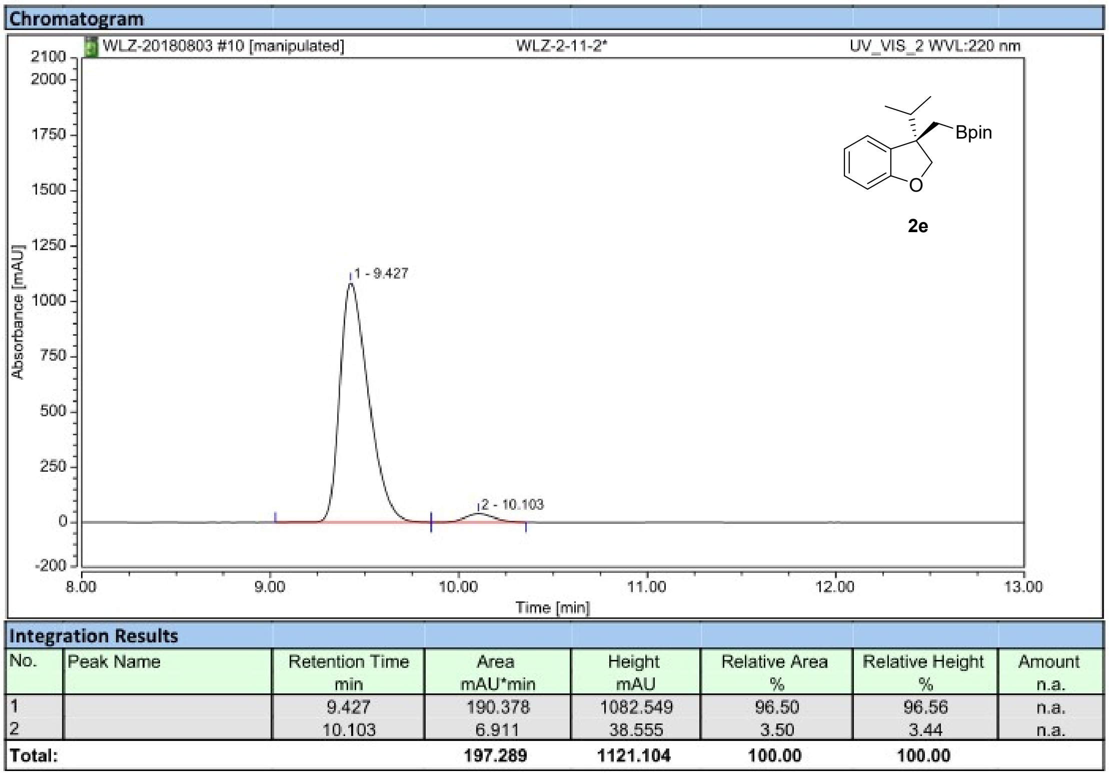


**Integration Results**

| No. | Peak Name | Retention Time<br>min | Area<br>mAU*min | Height<br>mAU | Relative Area<br>% | Relative Height<br>% | Amount<br>n.a. |
|---|---|---|---|---|---|---|---|
| 1 | | 9.427 | 190.378 | 1082.549 | 96.50 | 96.56 | n.a. |
| 2 | | 10.103 | 6.911 | 38.555 | 3.50 | 3.44 | n.a. |
| **Total:** | | | **197.289** | **1121.104** | **100.00** | **100.00** | |

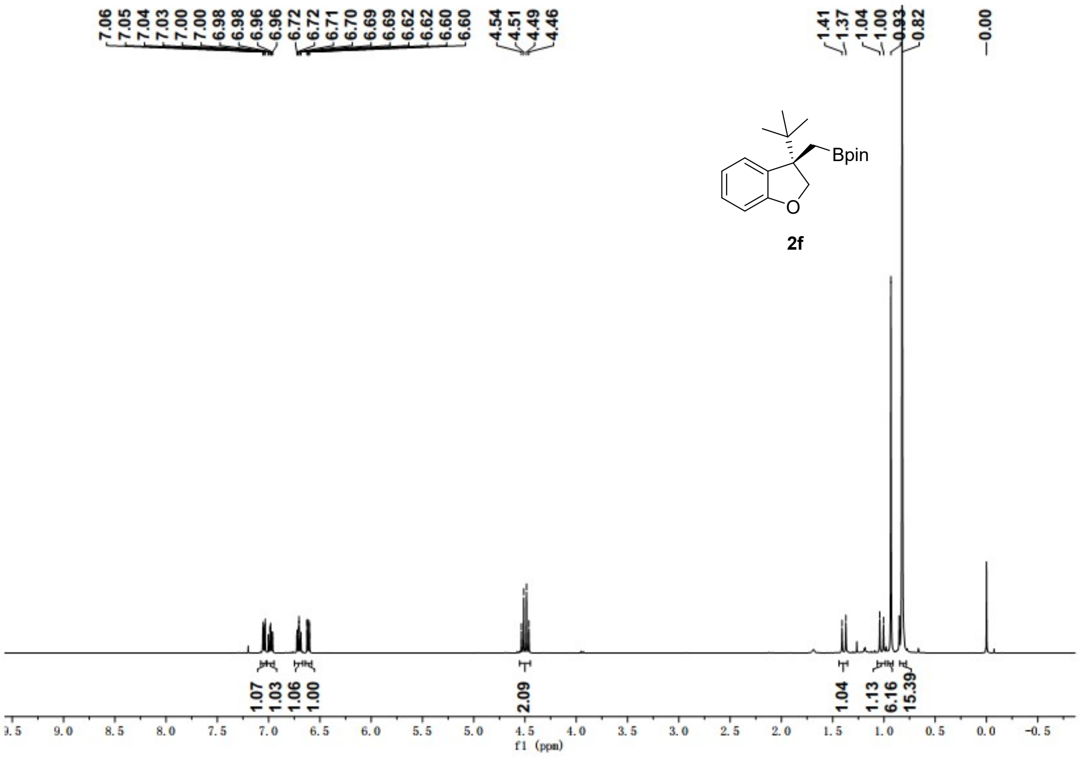

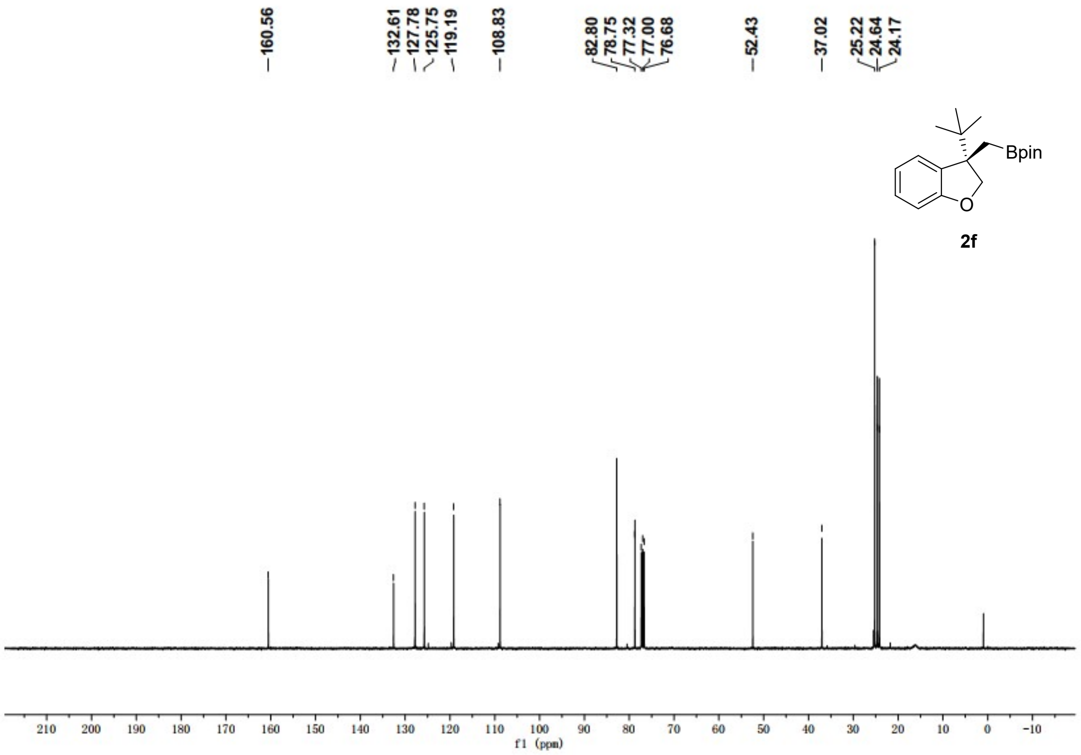


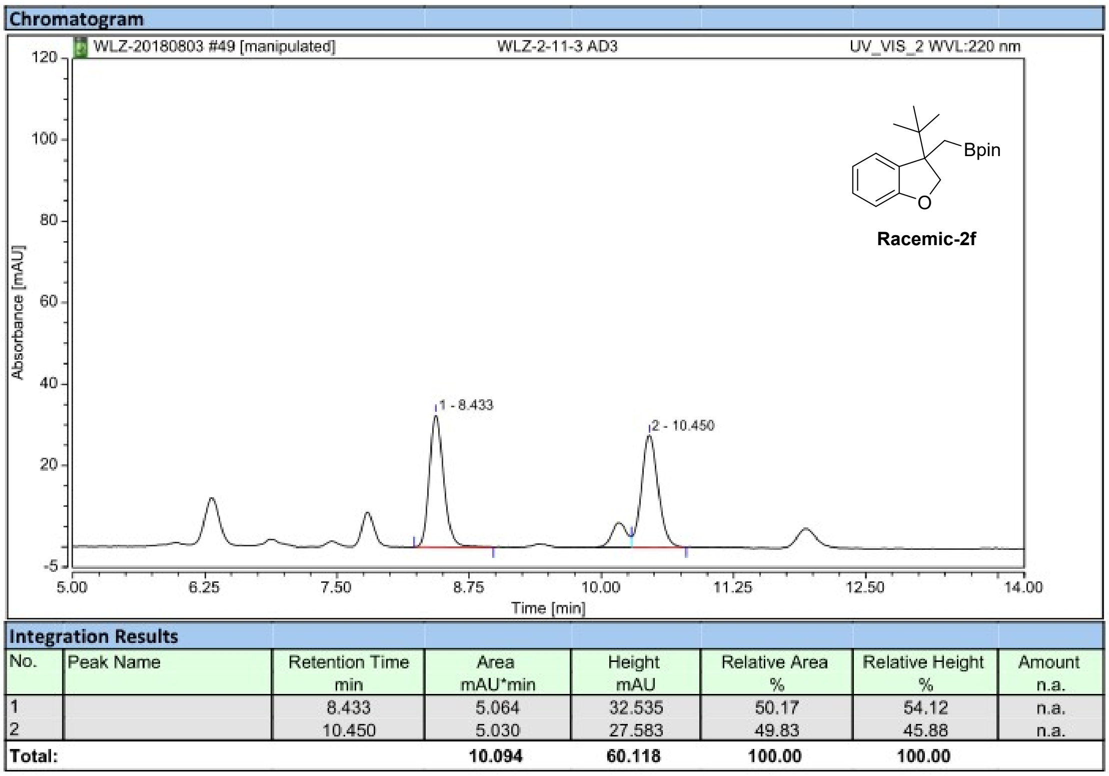


**Integration Results**

| No. | Peak Name | Retention Time min | Area mAU*min | Height mAU | Relative Area % | Relative Height % | Amount n.a. |
|---|---|---|---|---|---|---|---|
| 1 | | 8.433 | 5.064 | 32.535 | 50.17 | 54.12 | n.a. |
| 2 | | 10.450 | 5.030 | 27.583 | 49.83 | 45.88 | n.a. |
| **Total:** | | | **10.094** | **60.118** | **100.00** | **100.00** | |

## Chromatogram

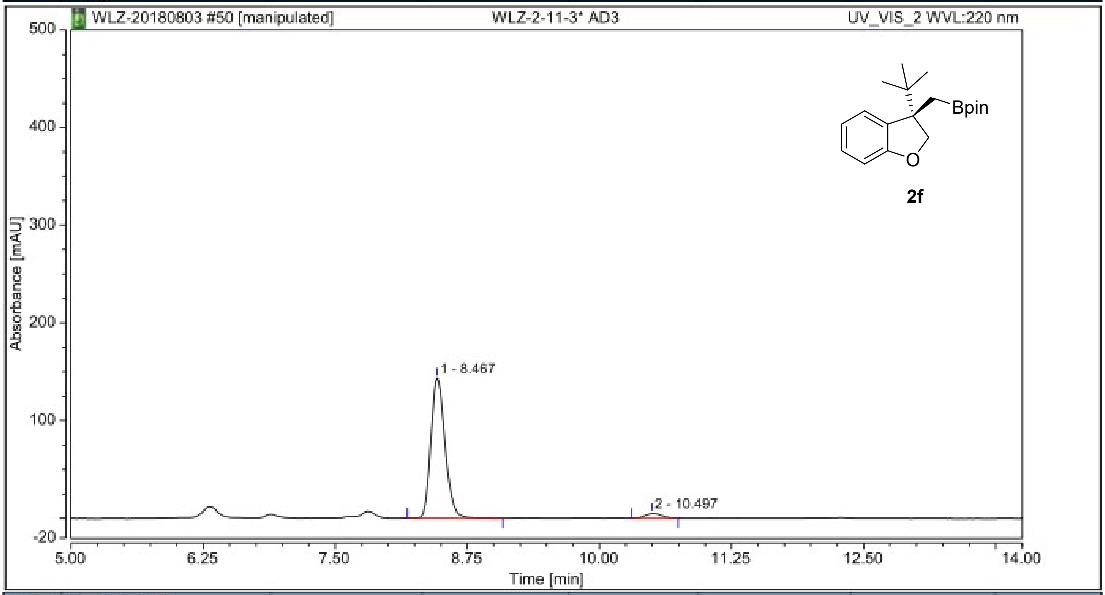


## Integration Results

| No. | Peak Name | Retention Time min | Area mAU*min | Height mAU | Relative Area % | Relative Height % | Amount n.a. |
|---|---|---|---|---|---|---|---|
| 1 | | 8.467 | 22.320 | 143.481 | 96.01 | 96.52 | n.a. |
| 2 | | 10.497 | 0.928 | 5.167 | 3.99 | 3.48 | n.a. |
| **Total:** | | | **23.248** | **148.648** | **100.00** | **100.00** | |

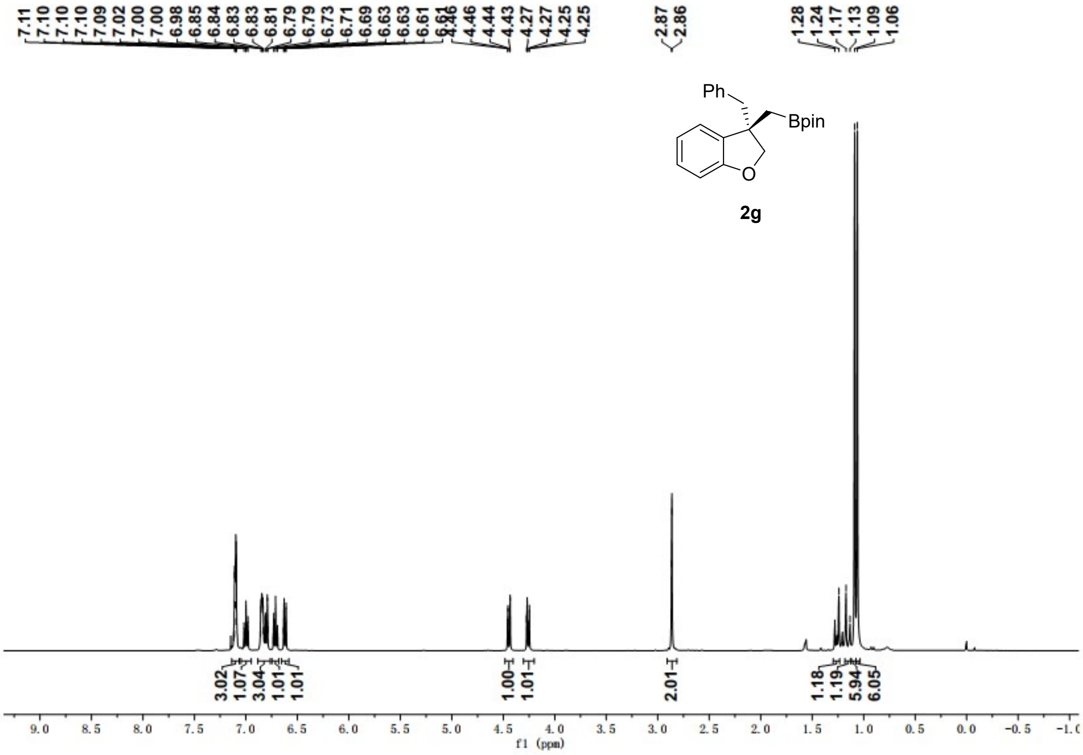

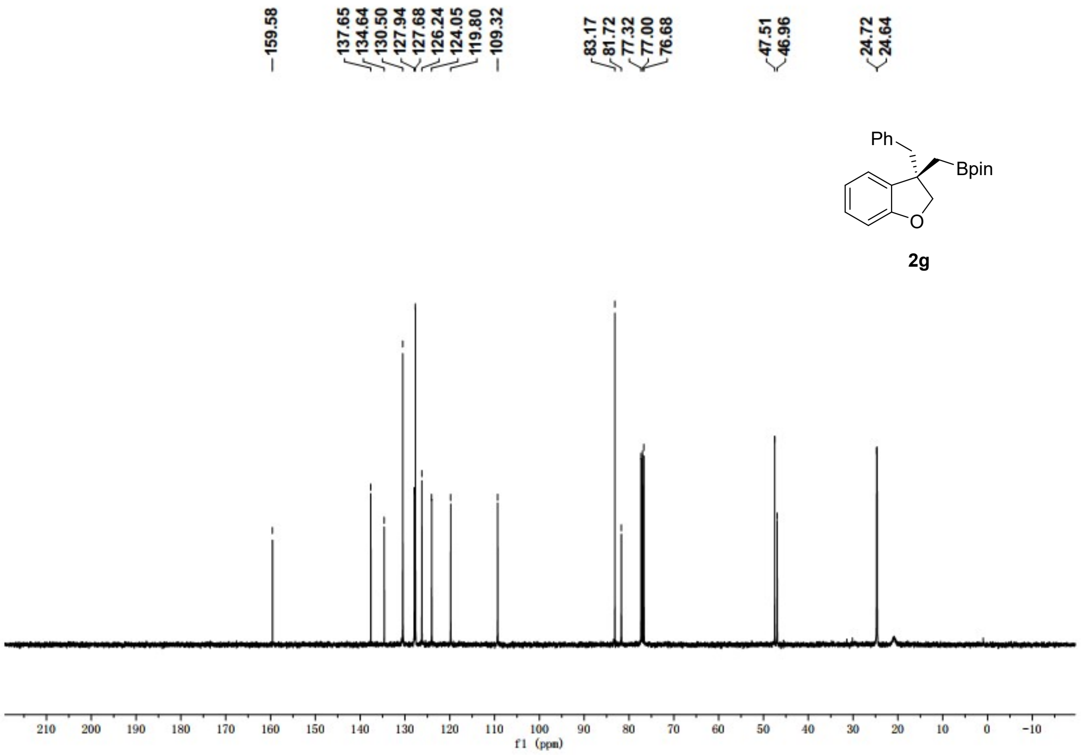


Chromatogram

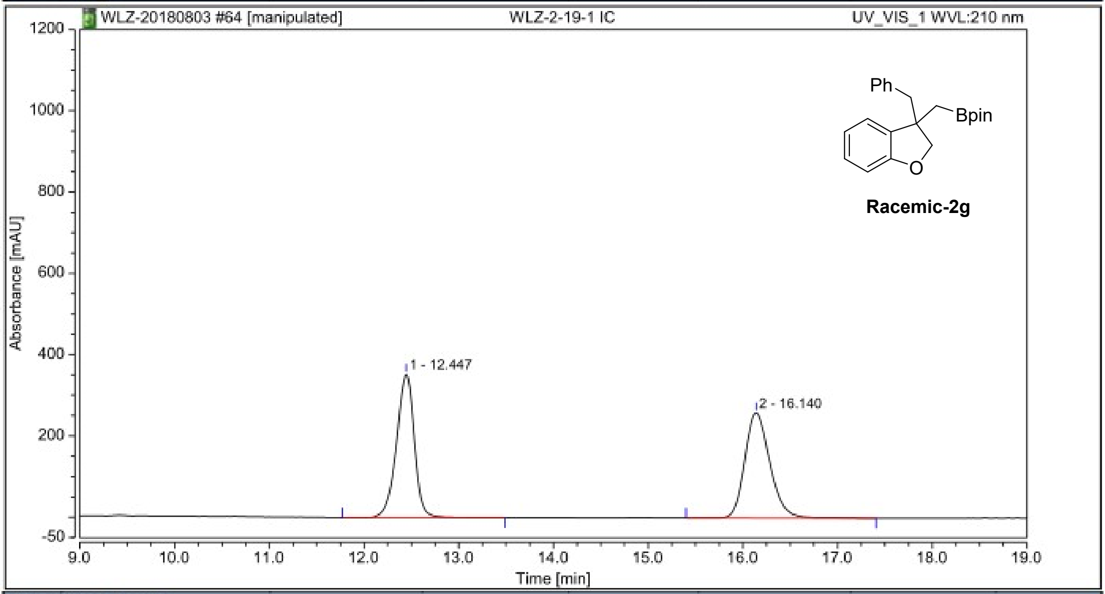


Integration Results

| No. | Peak Name | Retention Time min | Area mAU*min | Height mAU | Relative Area % | Relative Height % | Amount n.a. |
|---|---|---|---|---|---|---|---|
| 1 | | 12.447 | 77.316 | 353.226 | 49.32 | 57.71 | n.a. |
| 2 | | 16.140 | 79.453 | 258.850 | 50.68 | 42.29 | n.a. |
| **Total:** | | | **156.768** | **612.075** | **100.00** | **100.00** | |

## Chromatogram

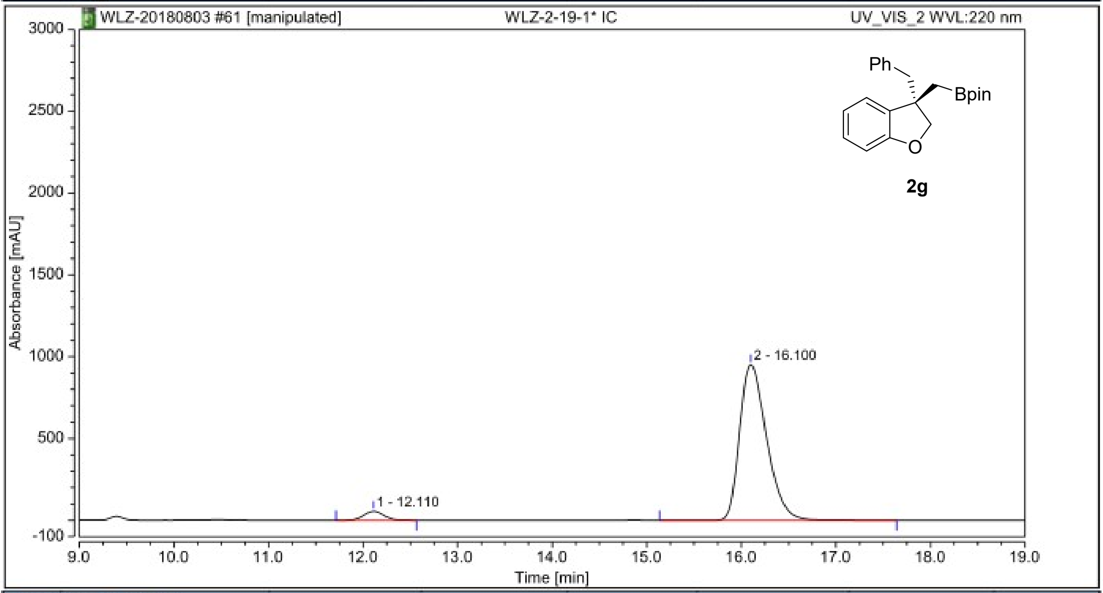


## Integration Results

| No. | Peak Name | Retention Time min | Area mAU*min | Height mAU | Relative Area % | Relative Height % | Amount n.a. |
|---|---|---|---|---|---|---|---|
| 1 | | 12.110 | 13.801 | 56.139 | 4.18 | 5.56 | n.a. |
| 2 | | 16.100 | 316.038 | 952.972 | 95.82 | 94.44 | n.a. |
| **Total:** | | | **329.839** | **1009.111** | **100.00** | **100.00** | |

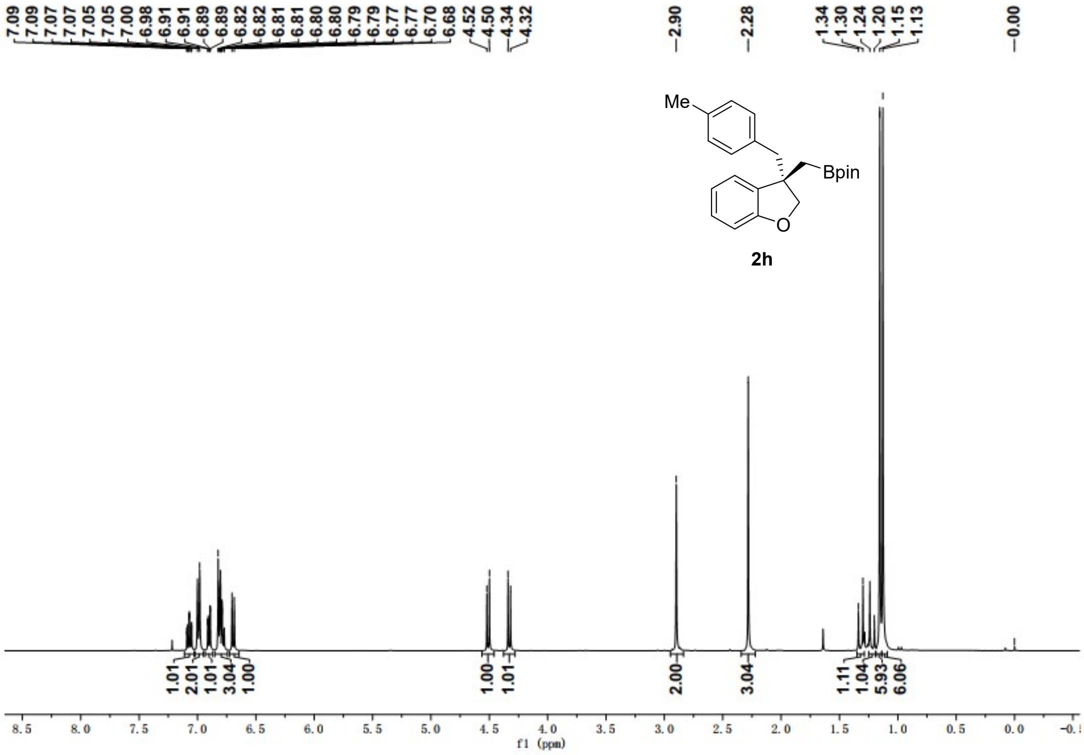

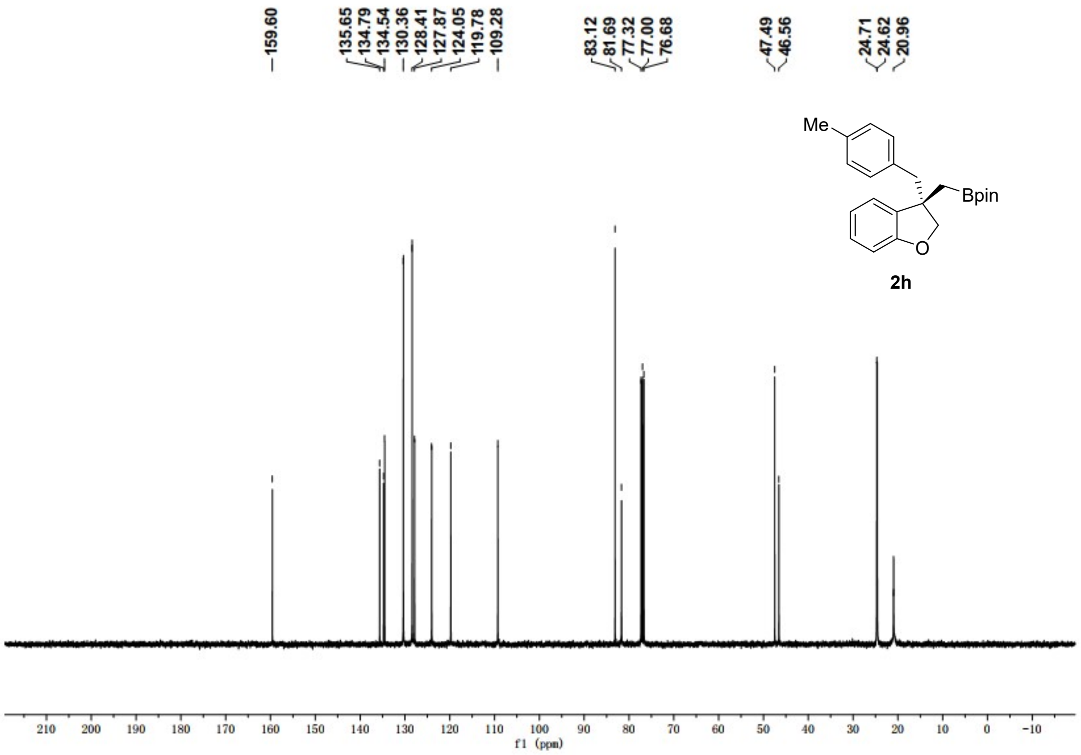


**Chromatogram**

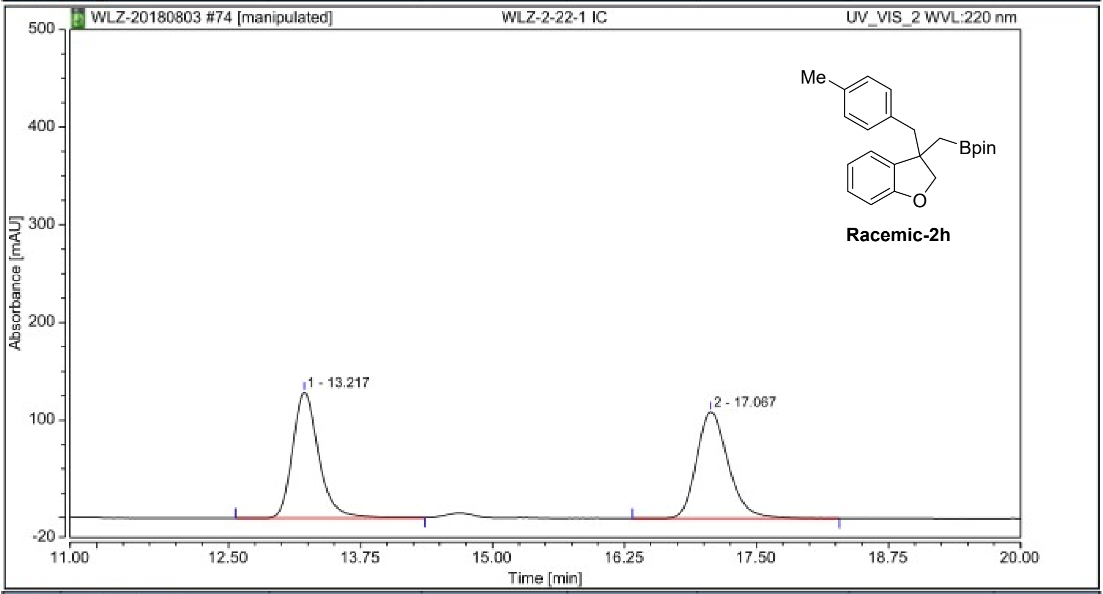


**Integration Results**

| No. | Peak Name | Retention Time min | Area mAU*min | Height mAU | Relative Area % | Relative Height % | Amount n.a. |
|---|---|---|---|---|---|---|---|
| 1 | | 13.217 | 37.349 | 128.937 | 49.54 | 54.19 | n.a. |
| 2 | | 17.067 | 38.036 | 108.979 | 50.46 | 45.81 | n.a. |
| **Total:** | | | **75.386** | **237.916** | **100.00** | **100.00** | |

Chromatogram

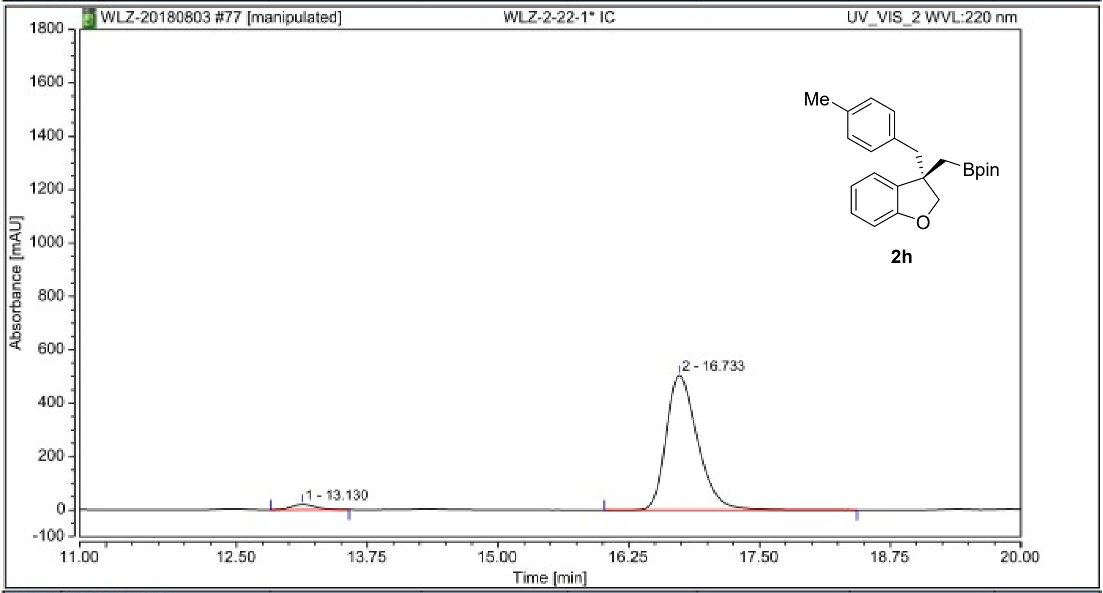


Integration Results

| No. | Peak Name | Retention Time min | Area mAU*min | Height mAU | Relative Area % | Relative Height % | Amount n.a. |
|---|---|---|---|---|---|---|---|
| 1 | | 13.130 | 5.316 | 19.704 | 2.92 | 3.76 | n.a. |
| 2 | | 16.733 | 176.673 | 504.250 | 97.08 | 96.24 | n.a. |
| **Total:** | | | **181.989** | **523.954** | **100.00** | **100.00** | |

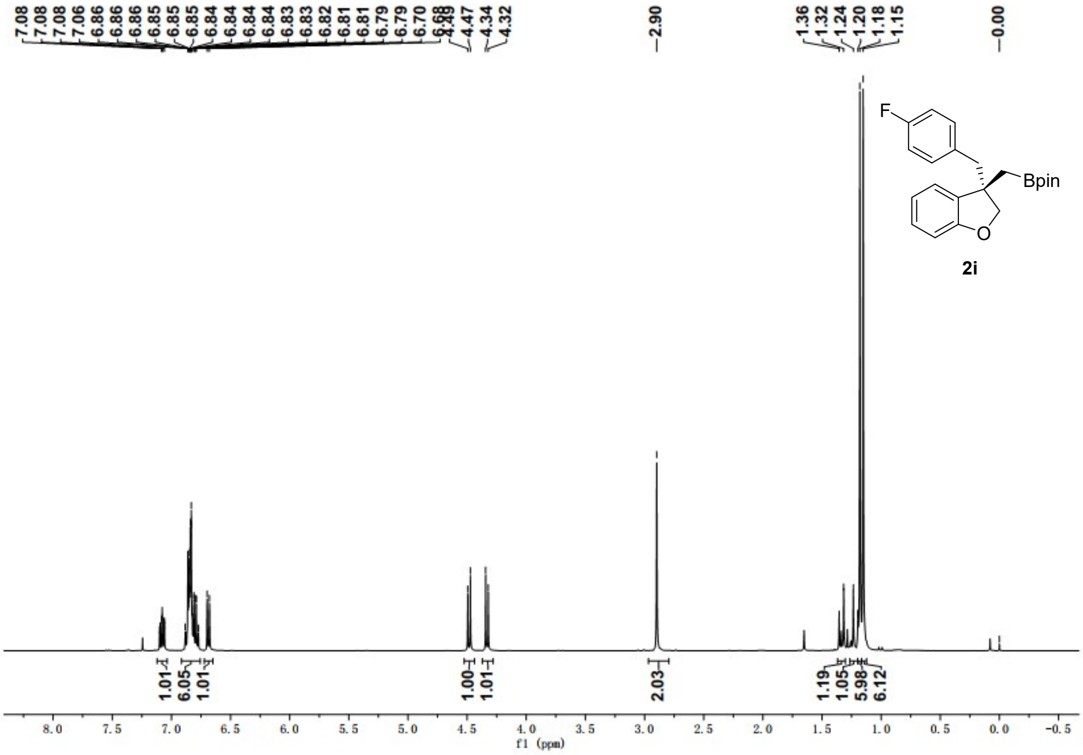

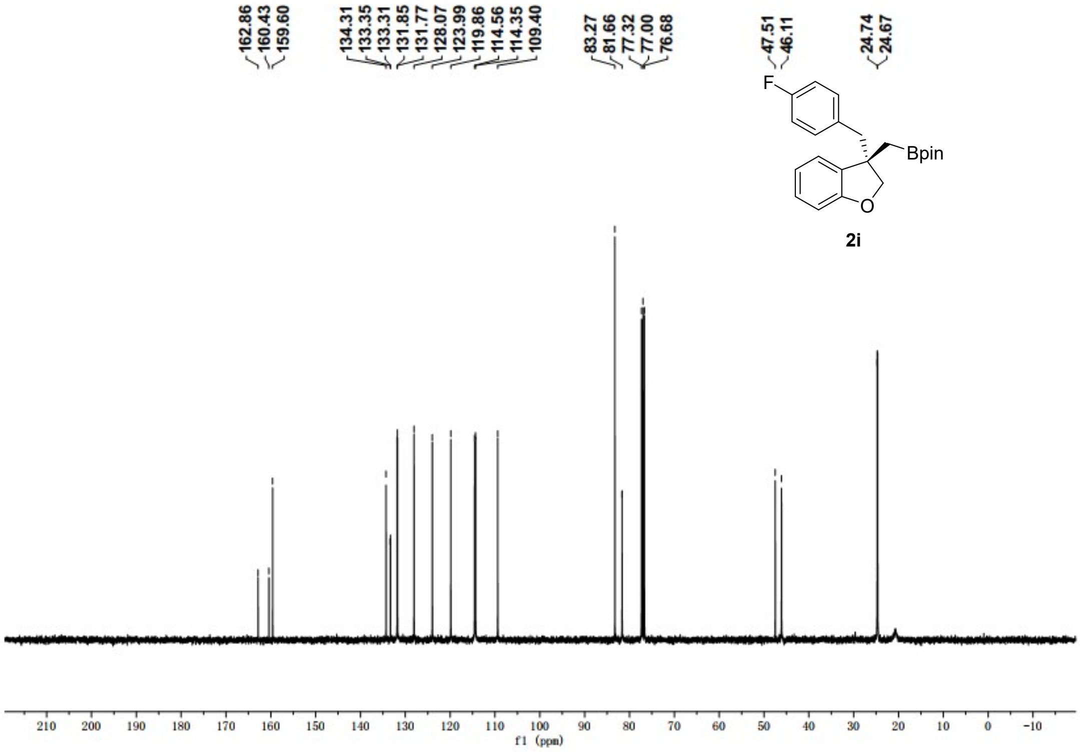
162.86
160.43
159.60
134.31
133.35
133.31
131.85
131.77
128.07
123.99
119.86
114.56
114.35
109.40
83.27
81.66
77.32
77.00
76.68
47.51
46.11
24.74
24.67
F
Bpin
O
2i
210 200 190 180 170 160 150 140 130 120 110 100 90 80 70 60 50 40 30 20 10 0 -10
f1 (ppm)

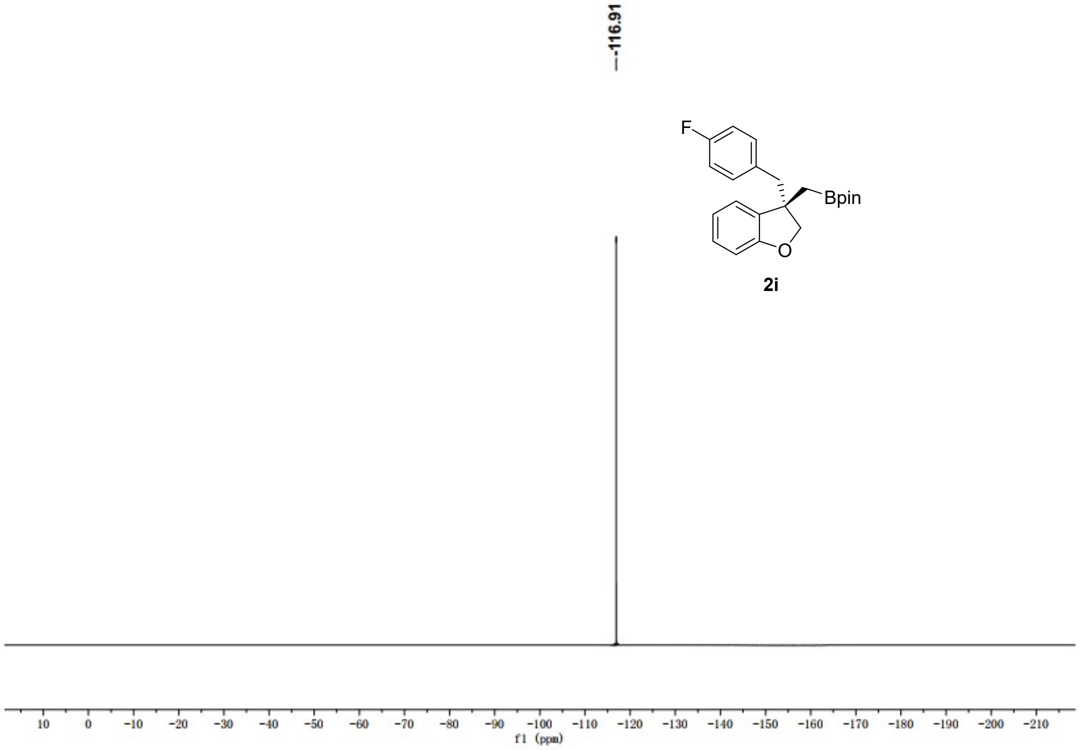
-116.91
F
Bpin
O
2i
10 0 -10 -20 -30 -40 -50 -60 -70 -80 -90 -100 -110 -120 -130 -140 -150 -160 -170 -180 -190 -200 -210
f1 (ppm)

## Chromatogram

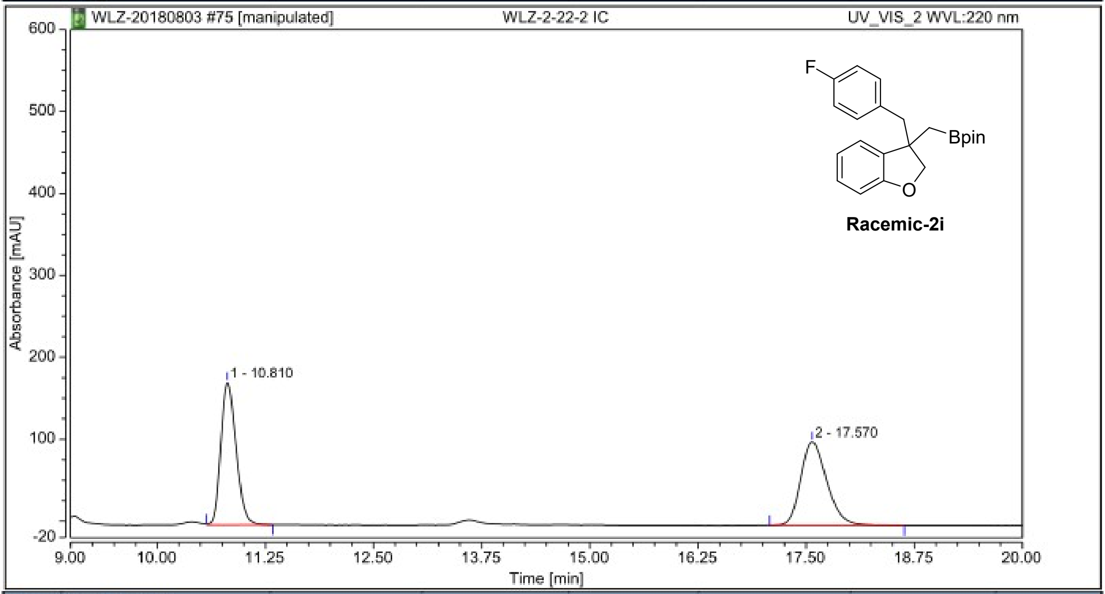


## Integration Results

| No. | Peak Name | Retention Time min | Area mAU*min | Height mAU | Relative Area % | Relative Height % | Amount n.a. |
|---|---|---|---|---|---|---|---|
| 1 | | 10.810 | 35.587 | 173.878 | 49.83 | 63.07 | n.a. |
| 2 | | 17.570 | 35.834 | 101.804 | 50.17 | 36.93 | n.a. |
| **Total:** | | | **71.421** | **275.683** | **100.00** | **100.00** | |

## Chromatogram

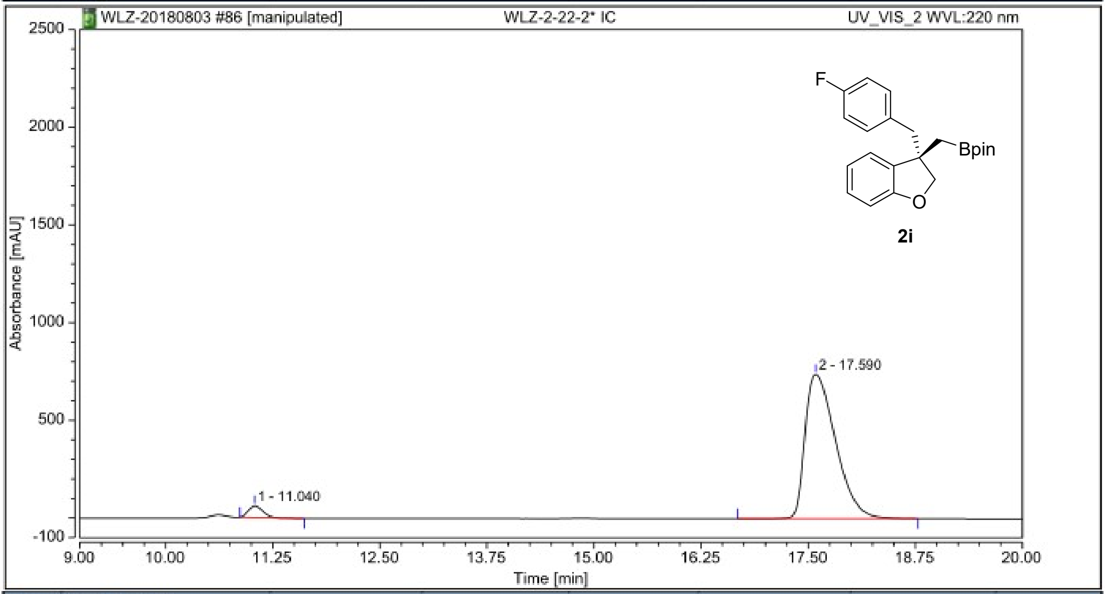


## Integration Results

| No. | Peak Name | Retention Time min | Area mAU*min | Height mAU | Relative Area % | Relative Height % | Amount n.a. |
|---|---|---|---|---|---|---|---|
| 1 | | 11.040 | 11.124 | 58.471 | 3.52 | 7.34 | n.a. |
| 2 | | 17.590 | 304.467 | 738.325 | 96.48 | 92.66 | n.a. |
| **Total:** | | | **315.591** | **796.795** | **100.00** | **100.00** | |

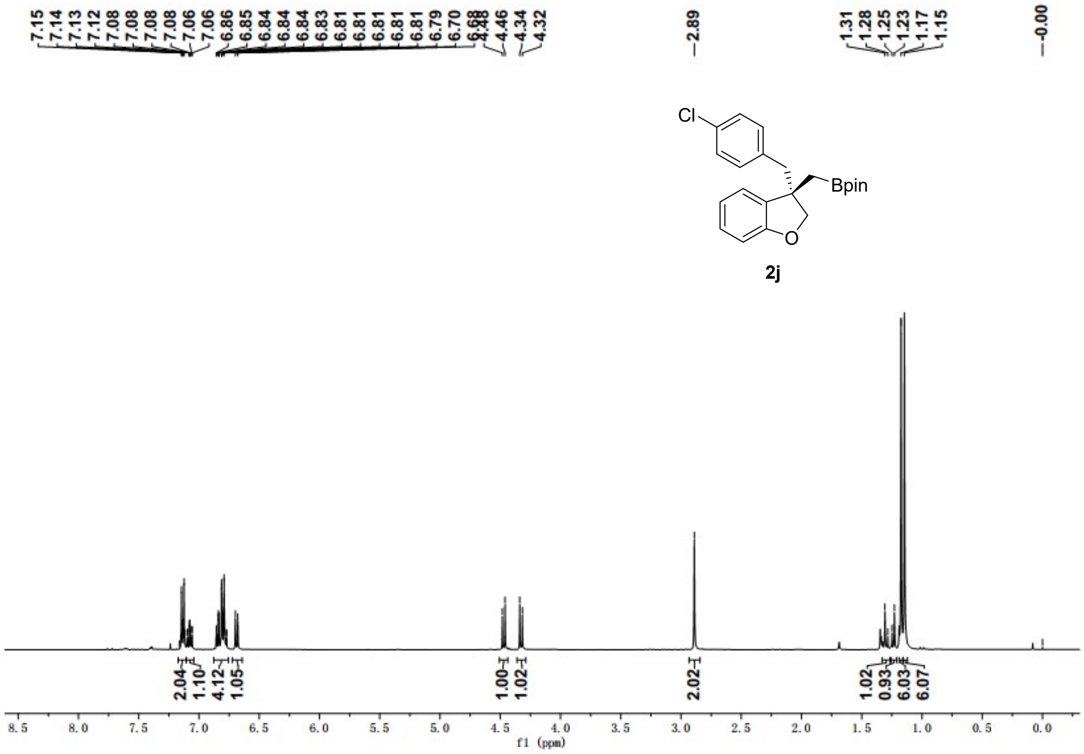
7.15
7.14
7.13
7.12
7.08
7.08
7.08
7.08
7.06
7.06
6.86
6.85
6.84
6.84
6.84
6.83
6.81
6.81
6.81
6.81
6.81
6.79
6.70
6.68
4.48
4.46
4.34
4.32
2.89
1.31
1.28
1.25
1.23
1.17
1.15
0.00
Cl
Bpin
O
2j
2.04
1.10
4.12
1.05
1.00
1.02
2.02
1.02
0.93
6.03
6.07
f1 (ppm)

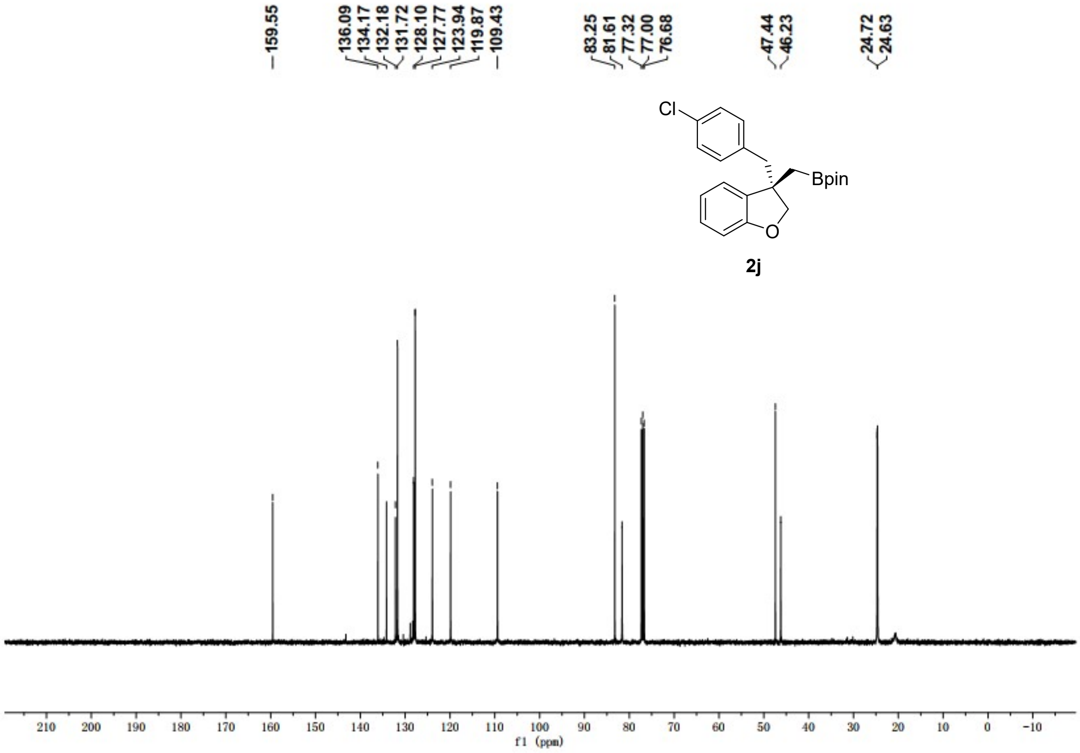
159.55
136.09
134.17
132.18
131.72
128.10
127.77
123.94
119.87
109.43
83.25
81.61
77.32
77.00
76.68
47.44
46.23
24.72
24.63
Cl
Bpin
O
2j
f1 (ppm)

## Chromatogram

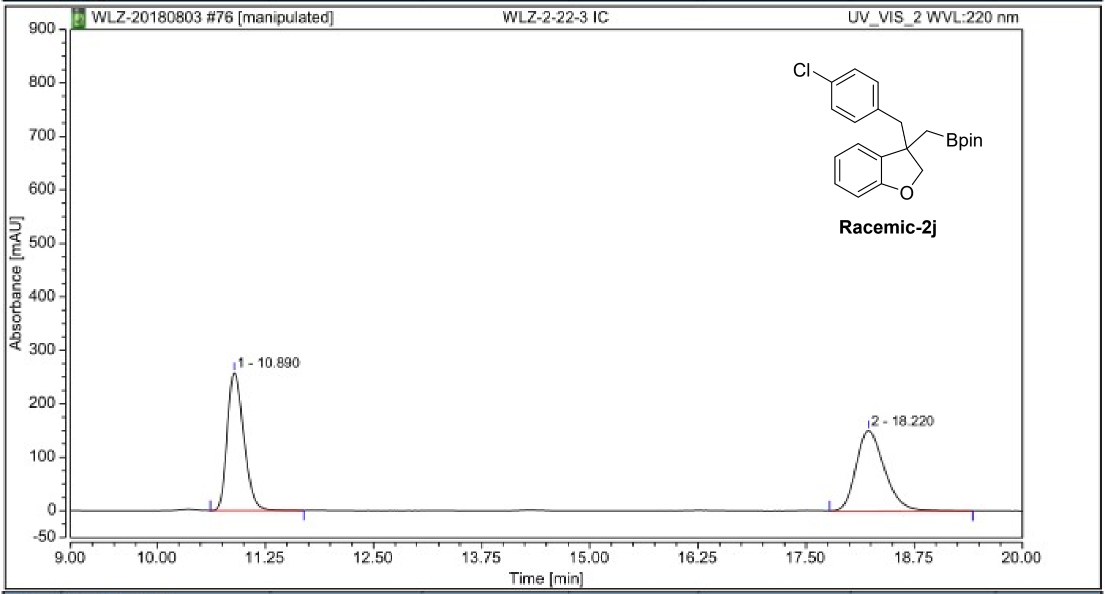


## Integration Results

| No. | Peak Name | Retention Time min | Area mAU*min | Height mAU | Relative Area % | Relative Height % | Amount n.a. |
|---|---|---|---|---|---|---|---|
| 1 | | 10.890 | 57.501 | 258.380 | 50.05 | 63.24 | n.a. |
| 2 | | 18.220 | 57.379 | 150.217 | 49.95 | 36.76 | n.a. |
| **Total:** | | | **114.880** | **408.598** | **100.00** | **100.00** | |

## Chromatogram

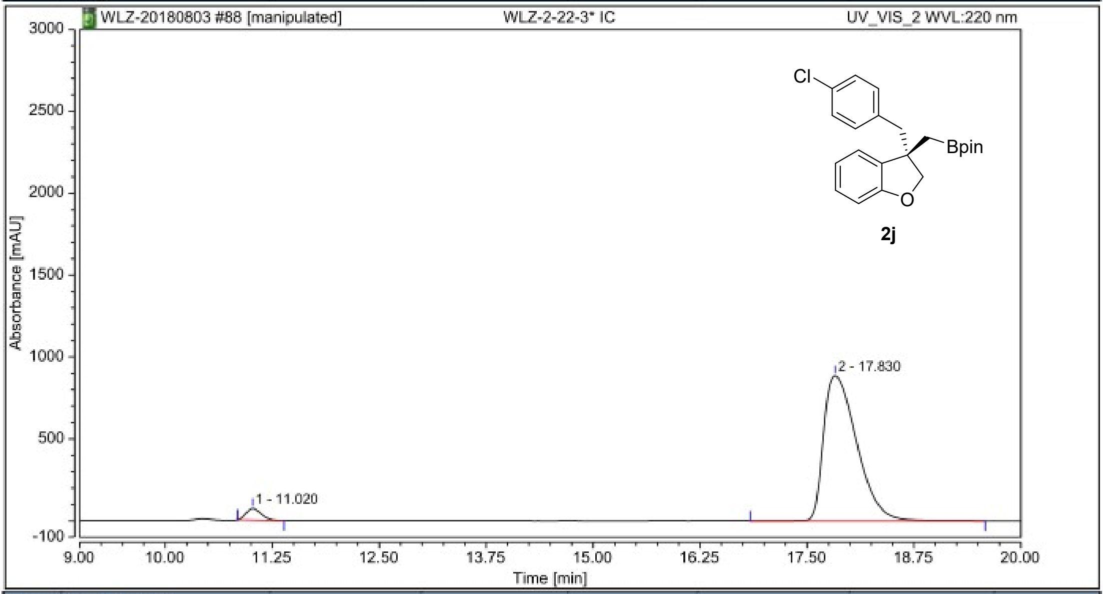


## Integration Results

| No. | Peak Name | Retention Time min | Area mAU*min | Height mAU | Relative Area % | Relative Height % | Amount n.a. |
|---|---|---|---|---|---|---|---|
| 1 | | 11.020 | 14.407 | 70.398 | 3.50 | 7.37 | n.a. |
| 2 | | 17.830 | 397.459 | 885.091 | 96.50 | 92.63 | n.a. |
| **Total:** | | | **411.866** | **955.489** | **100.00** | **100.00** | |

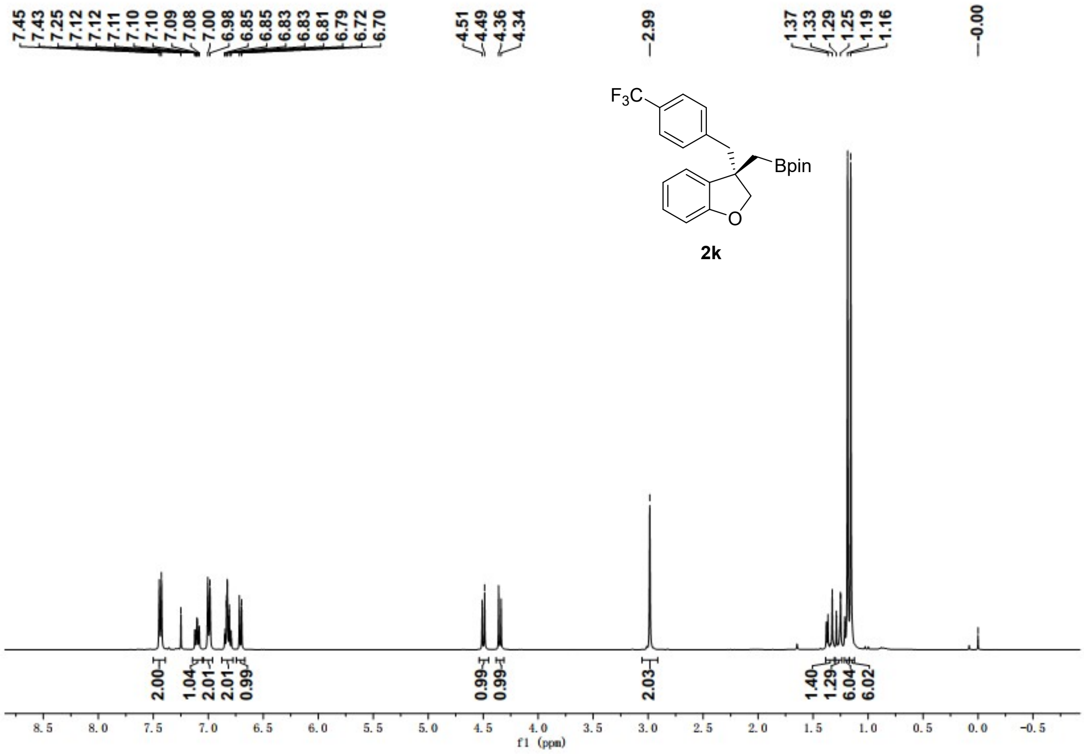
7.45 7.43 7.25 7.12 7.12 7.11 7.10 7.10 7.09 7.08 7.00 6.98 6.85 6.85 6.83 6.83 6.81 6.79 6.72 6.70
4.51 4.49 4.36 4.34
2.99
1.37 1.33 1.29 1.25 1.19 1.16
-0.00
F3C
Bpin
O
2k
2.00 1.04 2.01 2.01 0.99
0.99 0.99
2.03
1.40 1.29 6.04 6.02
8.5 8.0 7.5 7.0 6.5 6.0 5.5 5.0 4.5 4.0 3.5 3.0 2.5 2.0 1.5 1.0 0.5 0.0 -0.5
f1 (ppm)

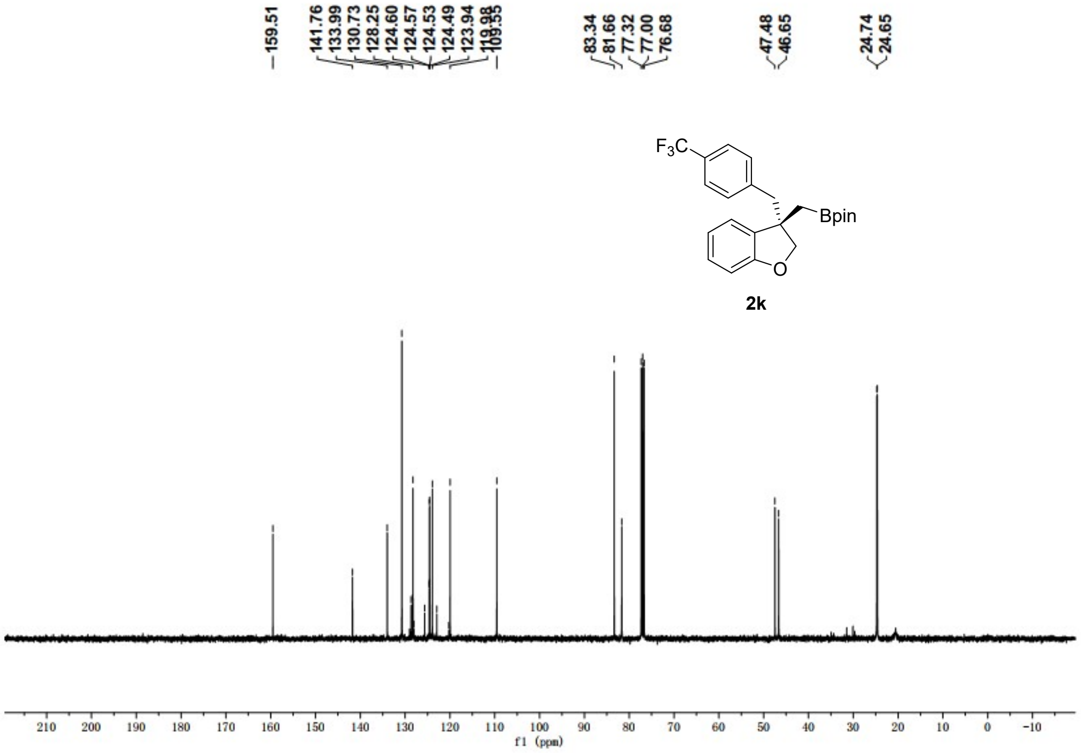
159.51
141.76 133.99 130.73 128.25 124.60 124.57 124.53 124.49 123.94 119.98 109.55
83.34 81.66 77.32 77.00 76.68
47.48 46.65
24.74 24.65
F3C
Bpin
O
2k
210 200 190 180 170 160 150 140 130 120 110 100 90 80 70 60 50 40 30 20 10 0 -10
f1 (ppm)

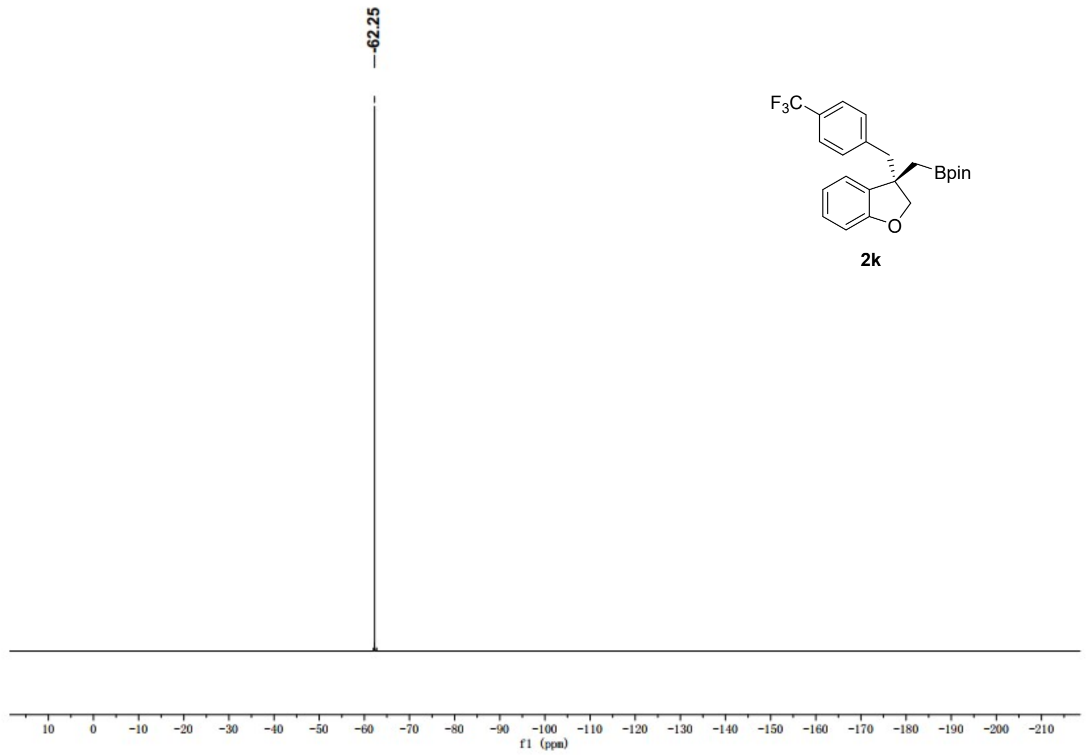


**Chromatogram**

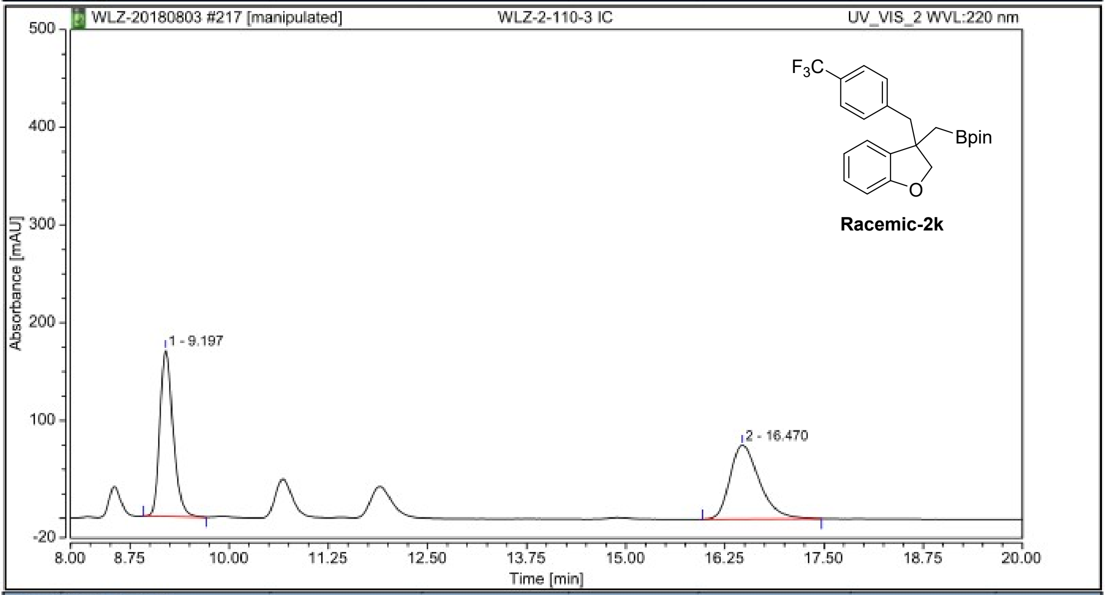


**Integration Results**

| No. | Peak Name | Retention Time min | Area mAU*min | Height mAU | Relative Area % | Relative Height % | Amount n.a. |
|---|---|---|---|---|---|---|---|
| 1 | | 9.197 | 32.857 | 170.105 | 50.24 | 69.22 | n.a. |
| 2 | | 16.470 | 32.547 | 75.637 | 49.76 | 30.78 | n.a. |
| **Total:** | | | **65.404** | **245.742** | **100.00** | **100.00** | |

## Chromatogram

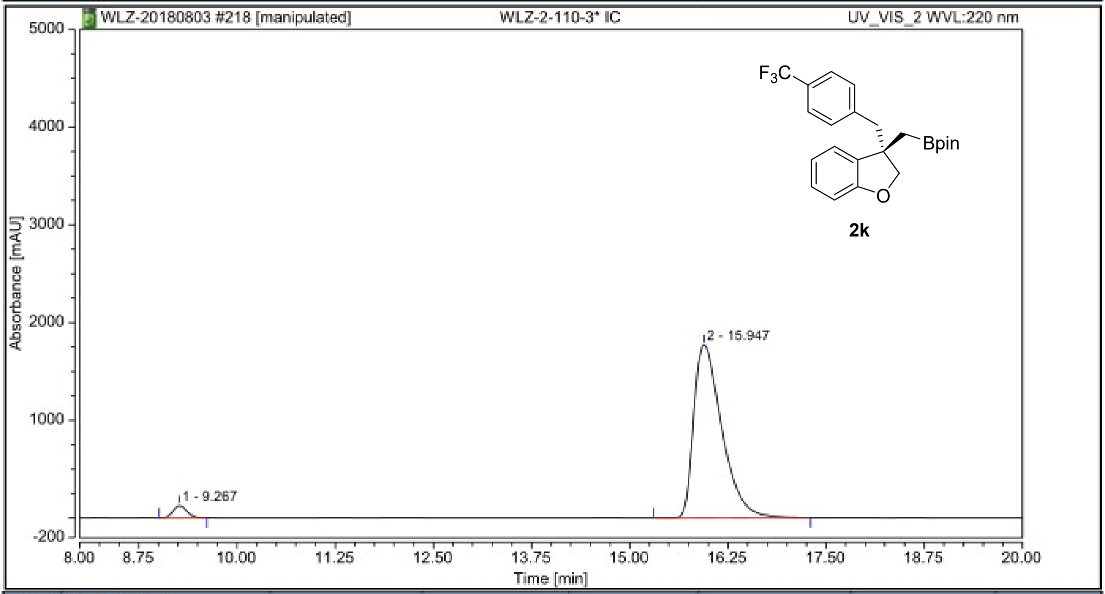


## Integration Results

| No. | Peak Name | Retention Time<br>min | Area<br>mAU*min | Height<br>mAU | Relative Area<br>% | Relative Height<br>% | Amount<br>n.a. |
|---|---|---|---|---|---|---|---|
| 1 | | 9.267 | 25.500 | 124.434 | 3.35 | 6.57 | n.a. |
| 2 | | 15.947 | 735.375 | 1769.338 | 96.65 | 93.43 | n.a. |
| **Total:** | | | **760.876** | **1893.772** | **100.00** | **100.00** | |

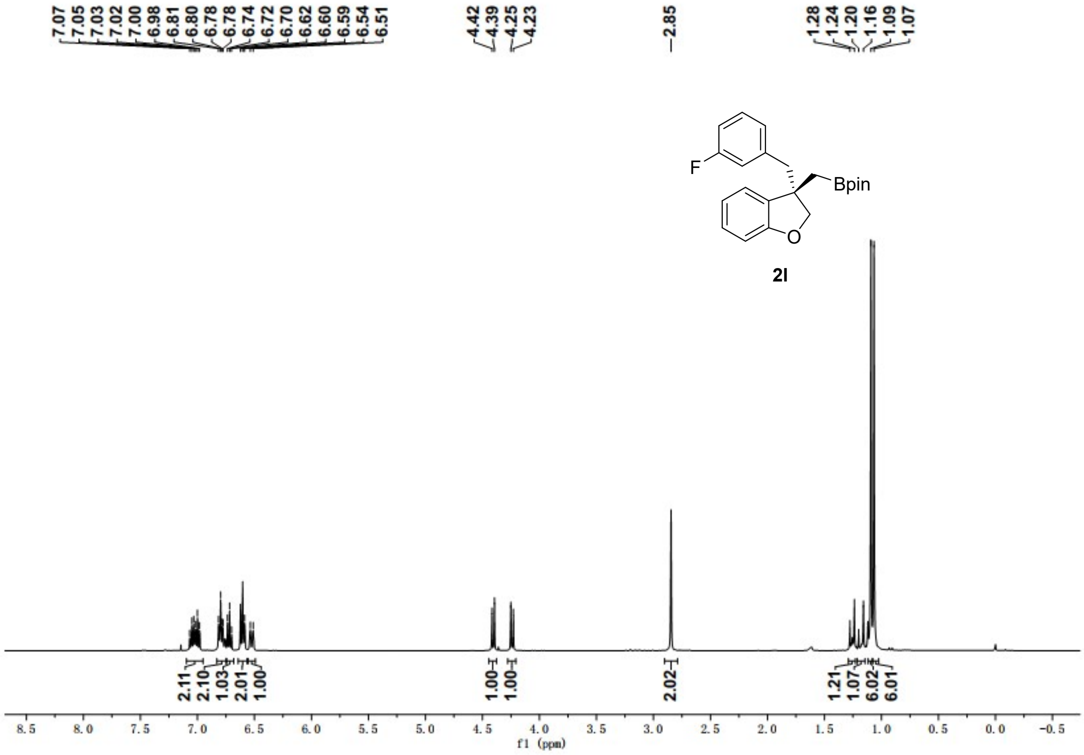

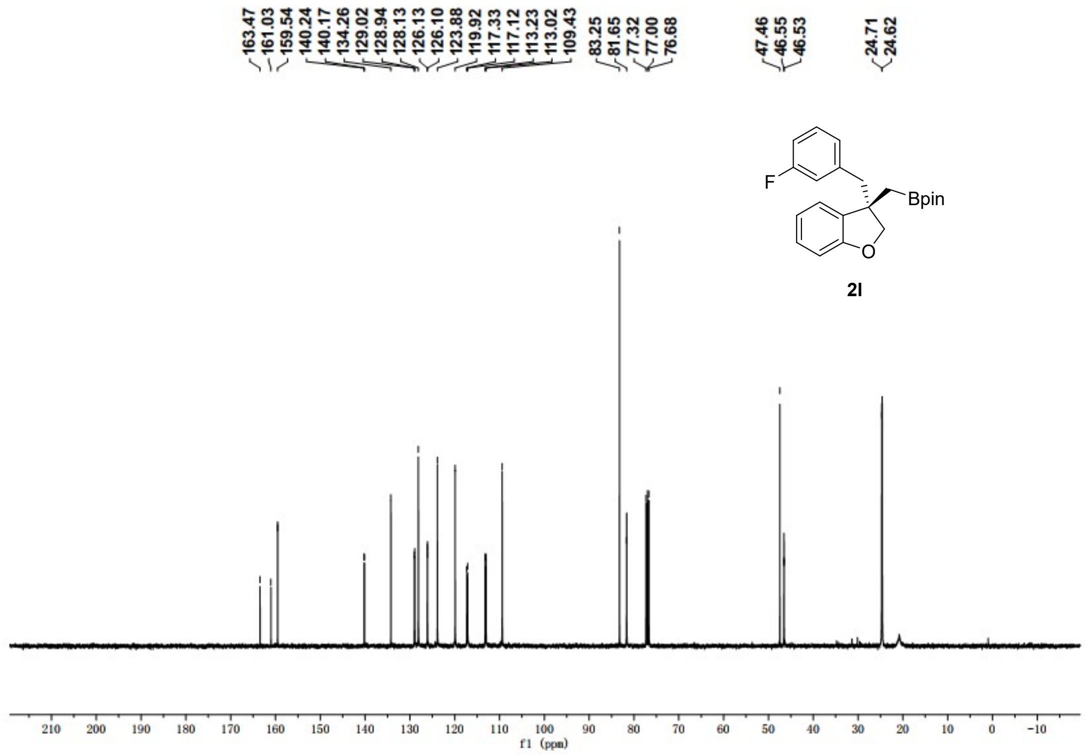
163.47
161.03
159.54
140.24
140.17
134.26
129.02
128.94
128.13
126.13
126.10
123.88
119.92
117.33
117.12
113.23
113.02
109.43
83.25
81.65
77.32
77.00
76.68
47.46
46.55
46.53
24.71
24.62
F
Bpin
O
2l
210 200 190 180 170 160 150 140 130 120 110 100 90 80 70 60 50 40 30 20 10 0 -10
f1 (ppm)

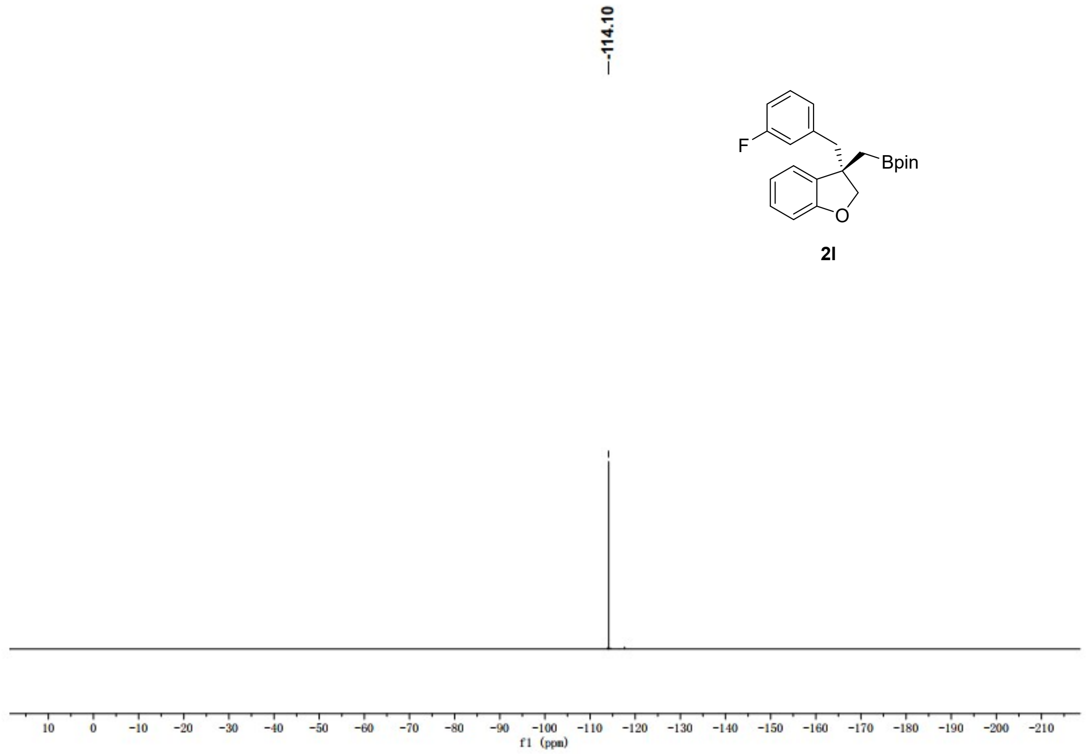
-114.10
F
Bpin
O
2l
10 0 -10 -20 -30 -40 -50 -60 -70 -80 -90 -100 -110 -120 -130 -140 -150 -160 -170 -180 -190 -200 -210
f1 (ppm)

## Chromatogram

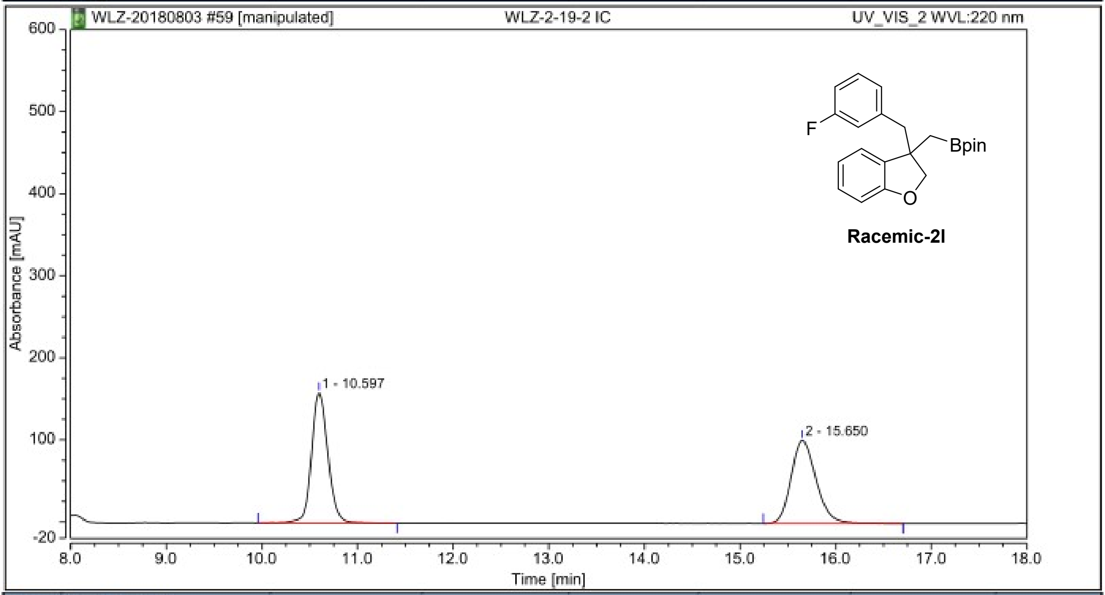


## Integration Results

| No. | Peak Name | Retention Time<br>min | Area<br>mAU*min | Height<br>mAU | Relative Area<br>% | Relative Height<br>% | Amount<br>n.a. |
|---|---|---|---|---|---|---|---|
| 1 | | 10.597 | 31.581 | 158.566 | 50.59 | 61.00 | n.a. |
| 2 | | 15.650 | 30.847 | 101.377 | 49.41 | 39.00 | n.a. |
| **Total:** | | | **62.428** | **259.943** | **100.00** | **100.00** | |

## Chromatogram

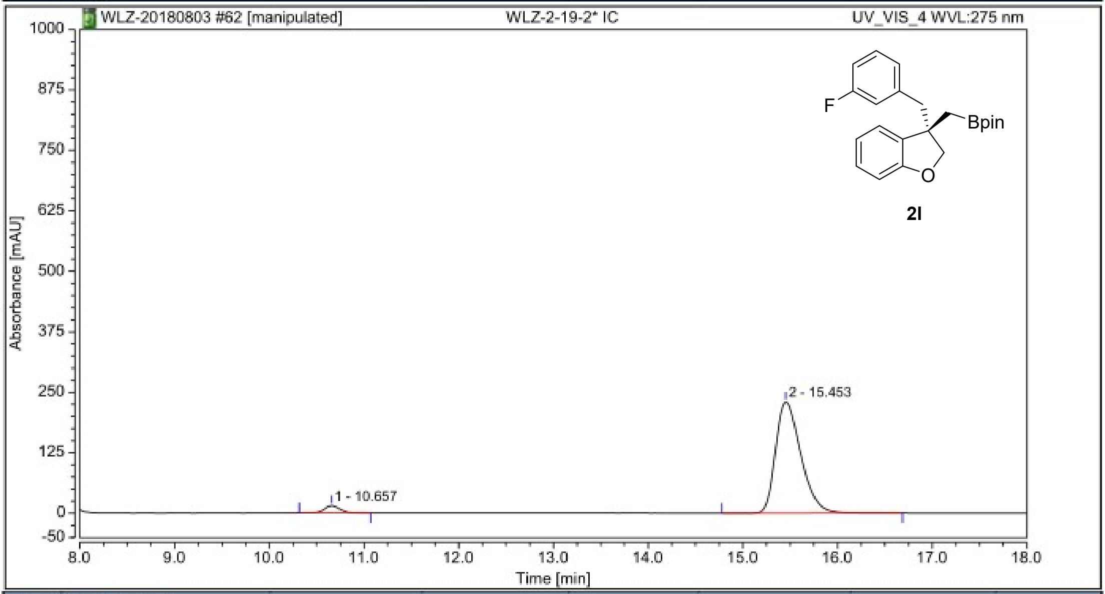


## Integration Results

| No. | Peak Name | Retention Time<br>min | Area<br>mAU*min | Height<br>mAU | Relative Area<br>% | Relative Height<br>% | Amount<br>n.a. |
|---|---|---|---|---|---|---|---|
| 1 | | 10.657 | 2.891 | 14.905 | 3.90 | 6.09 | n.a. |
| 2 | | 15.453 | 71.218 | 229.963 | 96.10 | 93.91 | n.a. |
| **Total:** | | | **74.109** | **244.868** | **100.00** | **100.00** | |

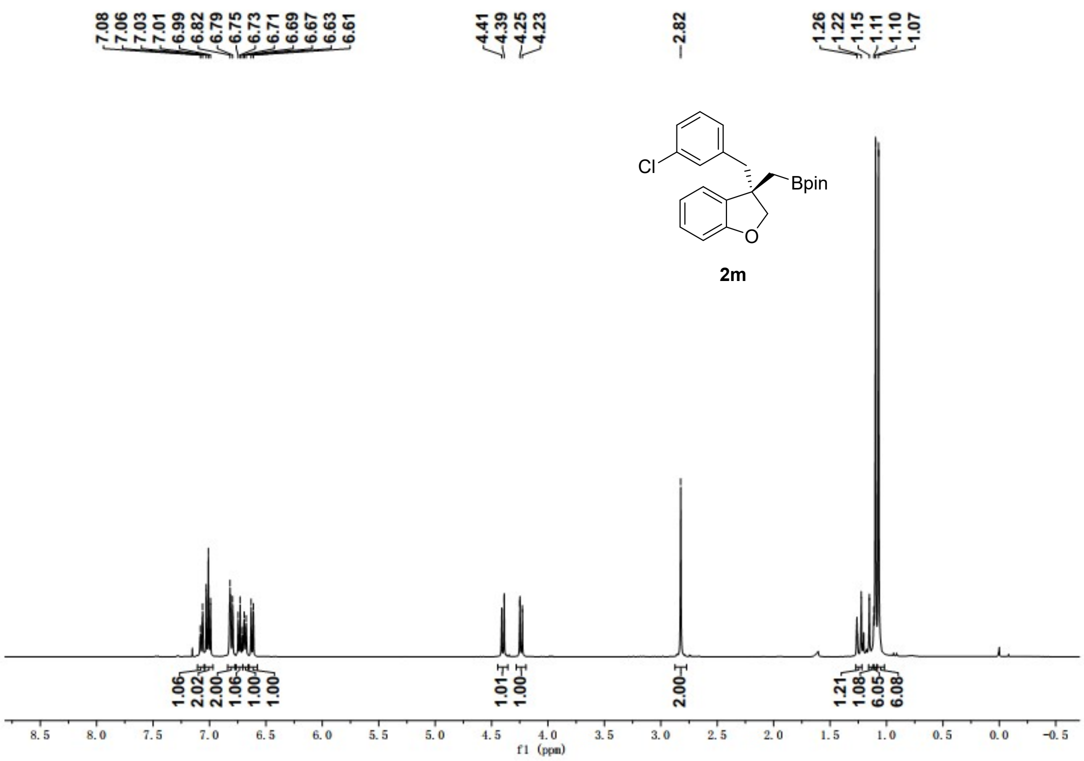
7.08
7.06
7.03
7.01
6.99
6.82
6.79
6.75
6.73
6.71
6.69
6.67
6.63
6.61
4.41
4.39
4.25
4.23
2.82
1.26
1.22
1.15
1.11
1.10
1.07
Cl
Bpin
O
2m
1.06
2.02
2.00
1.06
1.00
1.00
1.01
1.00
2.00
1.21
1.08
6.05
6.08
8.5
8.0
7.5
7.0
6.5
6.0
5.5
5.0
4.5
4.0
3.5
3.0
2.5
2.0
1.5
1.0
0.5
0.0
-0.5
f1 (ppm)

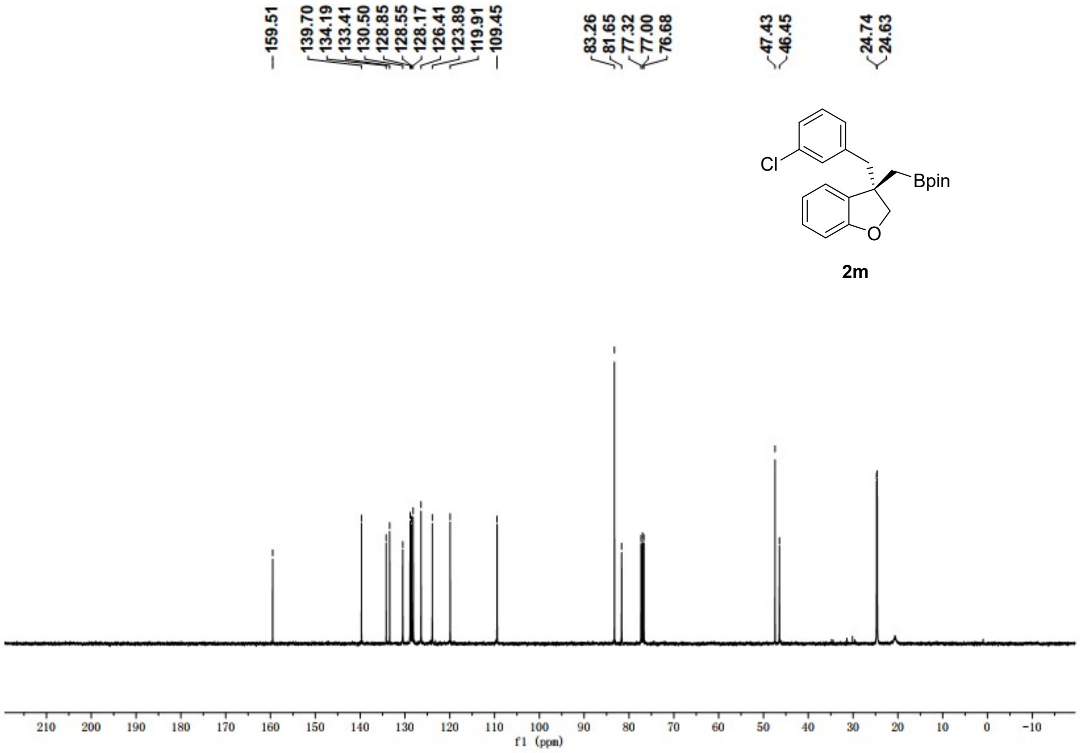
159.51
139.70
134.19
133.41
130.50
128.85
128.55
128.17
126.41
123.89
119.91
109.45
83.26
81.65
77.32
77.00
76.68
47.43
46.45
24.74
24.63
Cl
Bpin
O
2m
210
200
190
180
170
160
150
140
130
120
110
100
90
80
70
60
50
40
30
20
10
0
-10
f1 (ppm)

## Chromatogram

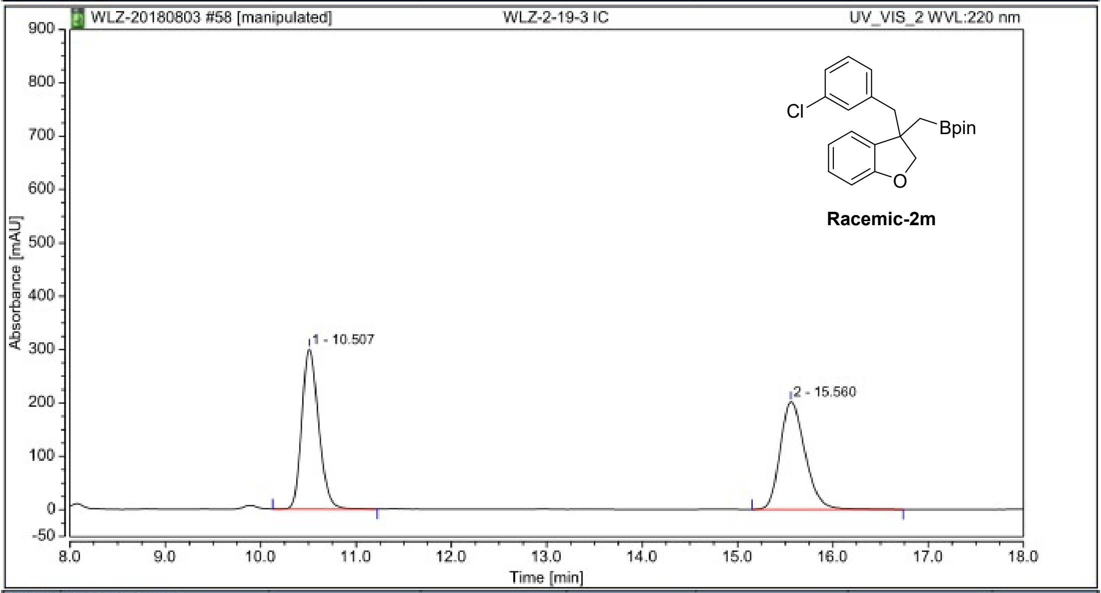


## Integration Results

| No. | Peak Name | Retention Time min | Area mAU*min | Height mAU | Relative Area % | Relative Height % | Amount n.a. |
|---|---|---|---|---|---|---|---|
| 1 | | 10.507 | 61.621 | 300.274 | 50.00 | 59.73 | n.a. |
| 2 | | 15.560 | 61.625 | 202.444 | 50.00 | 40.27 | n.a. |
| **Total:** | | | **123.245** | **502.718** | **100.00** | **100.00** | |

## Chromatogram

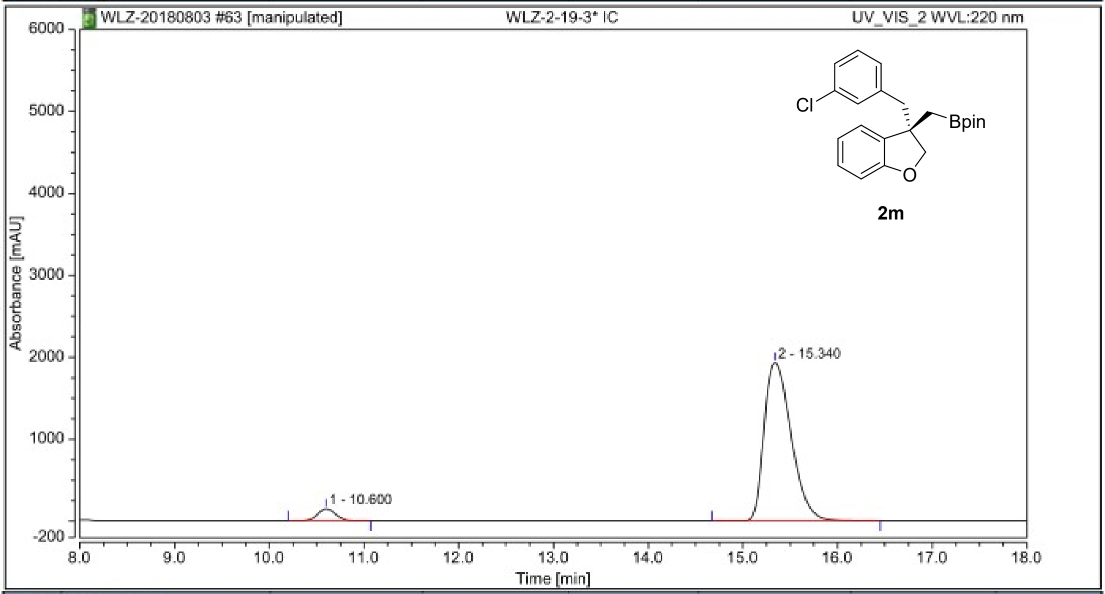


## Integration Results

| No. | Peak Name | Retention Time min | Area mAU*min | Height mAU | Relative Area % | Relative Height % | Amount n.a. |
|---|---|---|---|---|---|---|---|
| 1 | | 10.600 | 33.649 | 146.344 | 4.99 | 7.04 | n.a. |
| 2 | | 15.340 | 640.787 | 1931.901 | 95.01 | 92.96 | n.a. |
| **Total:** | | | **674.436** | **2078.244** | **100.00** | **100.00** | |

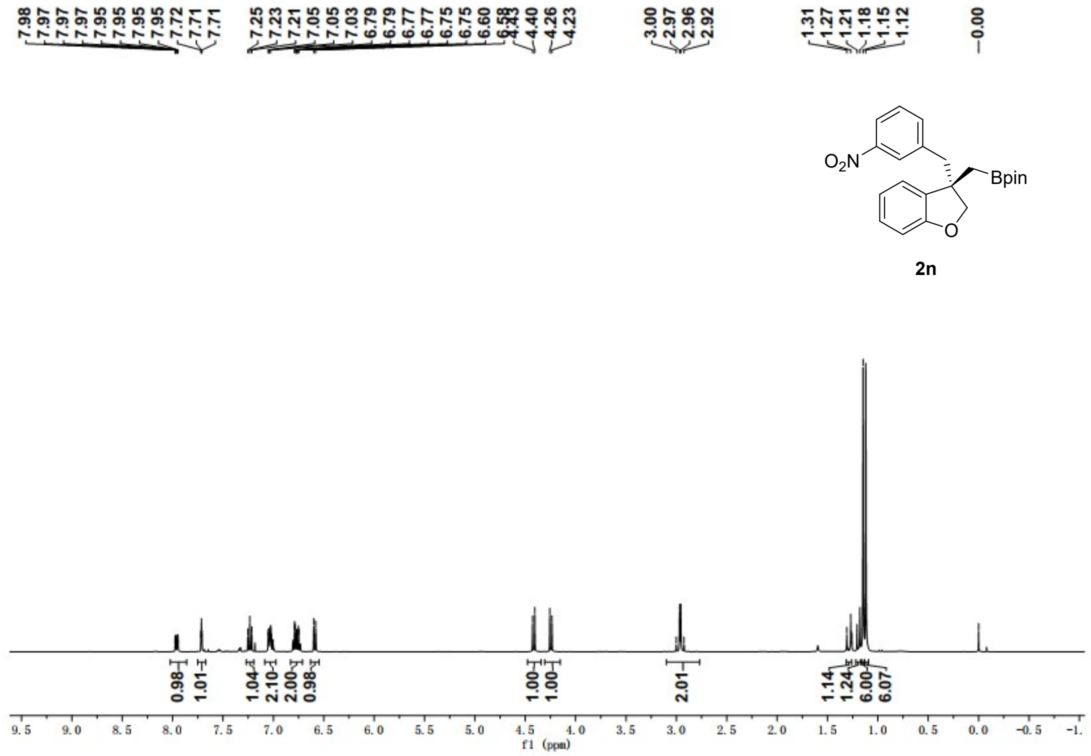
O2N
Bpin
O
2n
f1 (ppm)

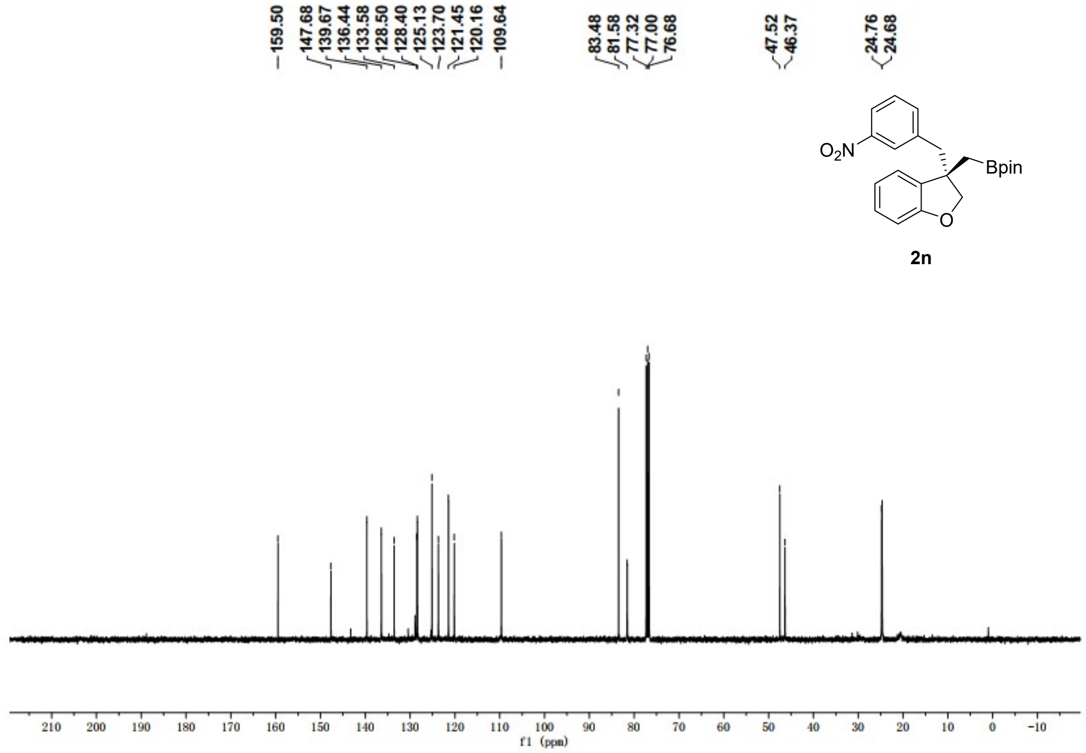
O2N
Bpin
O
2n
f1 (ppm)

## <Chromatogram>

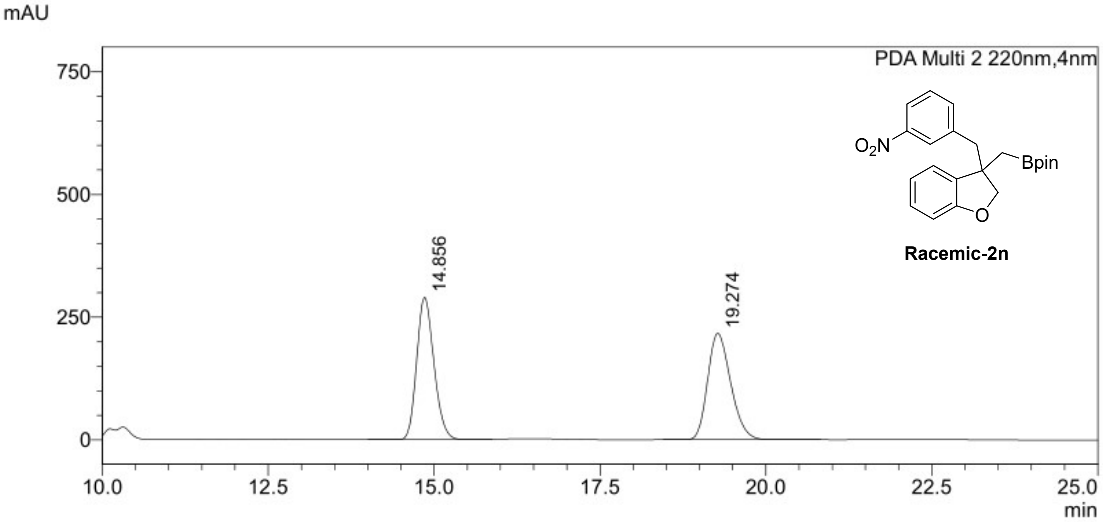


## <Peak Table>

PDA Ch2 220nm

| Peak# | Ret. Time | Area | Area% | Height | Height% |
|---|---|---|---|---|---|
| 1 | 14.856 | 5208712 | 50.096 | 289354 | 57.252 |
| 2 | 19.274 | 5188806 | 49.904 | 216049 | 42.748 |
| Total | | 10397517 | 100.000 | 505403 | 100.000 |

## <Chromatogram>

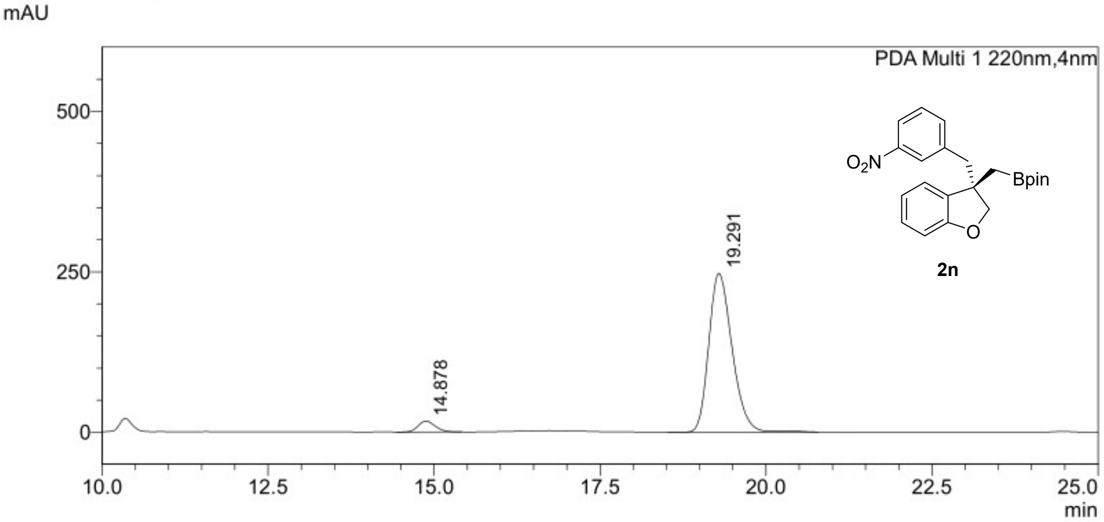


## <Peak Table>

PDA Ch1 220nm

| Peak# | Ret. Time | Area | Area% | Height | Height% |
|---|---|---|---|---|---|
| 1 | 14.878 | 306521 | 4.815 | 17077 | 6.459 |
| 2 | 19.291 | 6060051 | 95.185 | 247306 | 93.541 |
| Total | | 6366573 | 100.000 | 264383 | 100.000 |

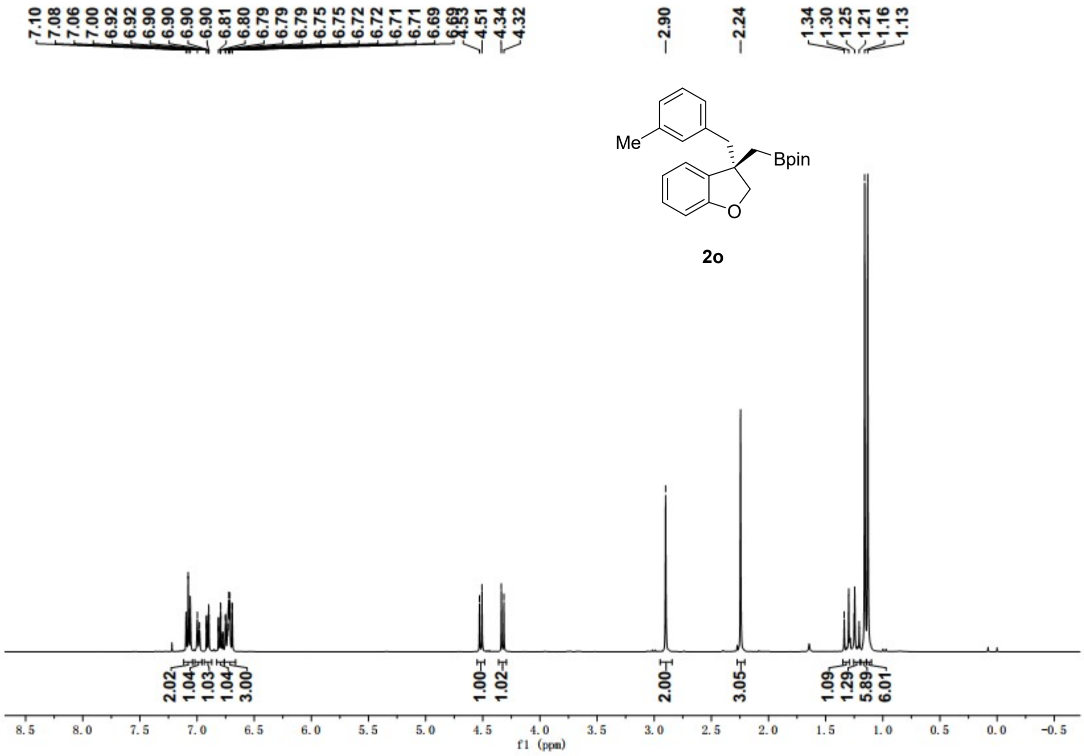
7.10 7.08 7.06 7.00 6.92 6.92 6.90 6.90 6.90 6.90 6.81 6.80 6.79 6.79 6.79 6.75 6.75 6.72 6.72 6.71 6.71 6.69 6.69 4.53 4.51 4.34 4.32
2.90
2.24
1.34 1.30 1.25 1.21 1.16 1.13
Me
Bpin
O
2o
2.02 1.04 1.03 1.04 3.00
1.00 1.02
2.00
3.05
1.09 1.29 5.89 6.01
f1 (ppm)

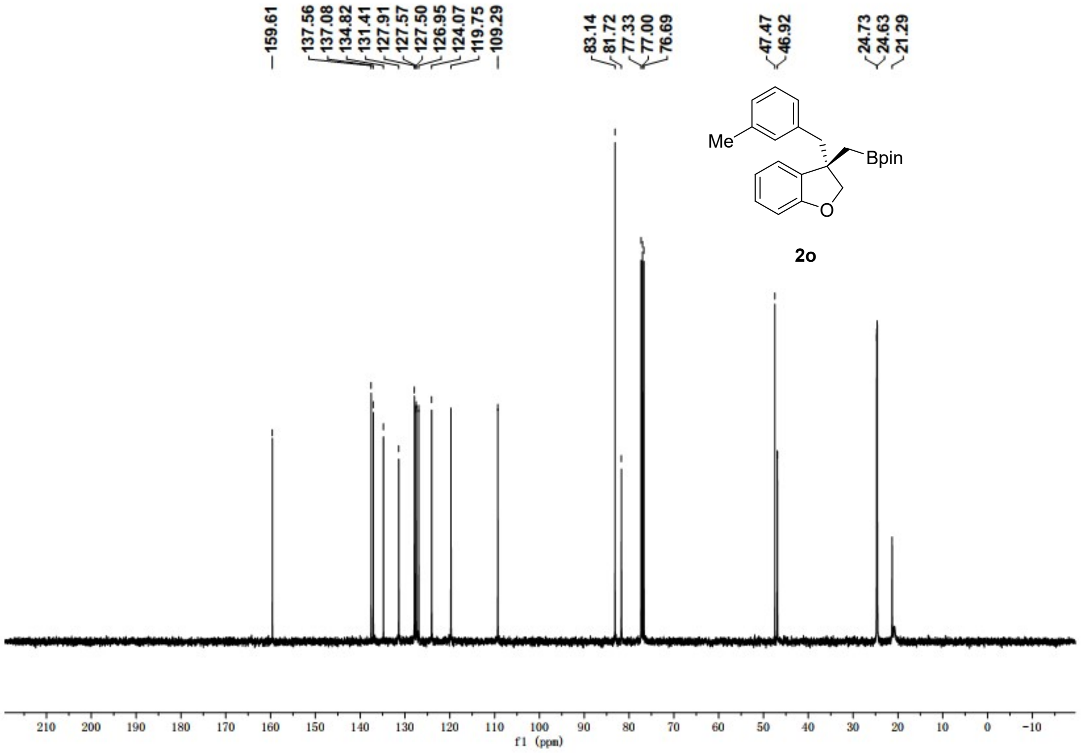
159.61
137.56 137.08 134.82 131.41 127.91 127.57 127.50 126.95 124.07 119.75
109.29
83.14 81.72 77.33 77.00 76.69
47.47 46.92
24.73 24.63 21.29
Me
Bpin
O
2o
f1 (ppm)

**Chromatogram**

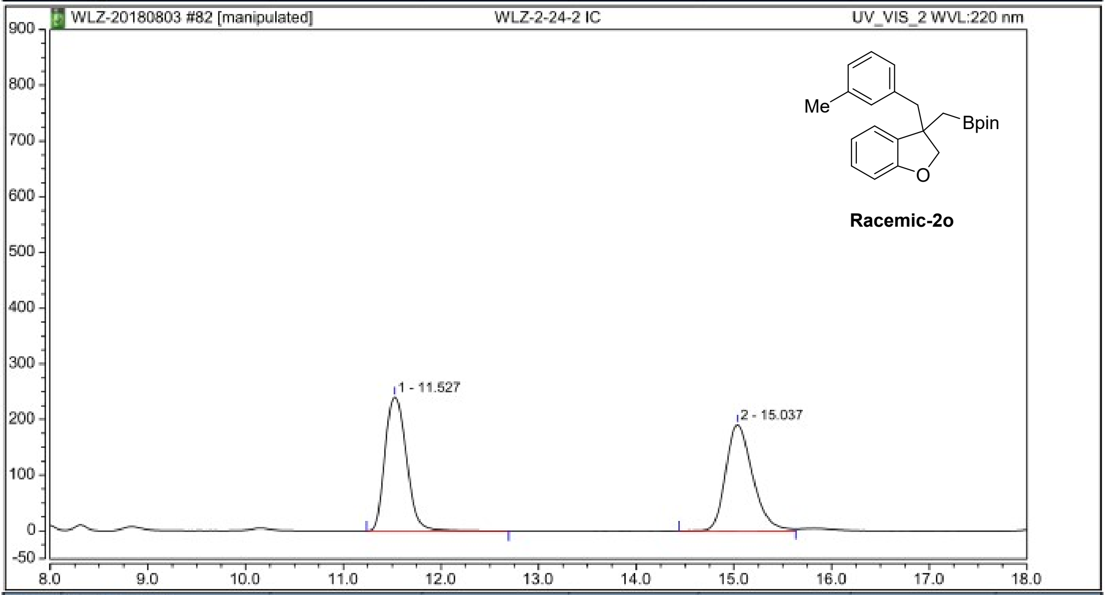


**Integration Results**

| No. | Peak Name | Retention Time min | Area mAU*min | Height mAU | Relative Area % | Relative Height % | Amount n.a. |
|---|---|---|---|---|---|---|---|
| 1 | | 11.527 | 61.189 | 240.428 | 49.99 | 55.76 | n.a. |
| 2 | | 15.037 | 61.221 | 190.718 | 50.01 | 44.24 | n.a. |
| **Total:** | | | **122.410** | **431.145** | **100.00** | **100.00** | |

**Chromatogram**

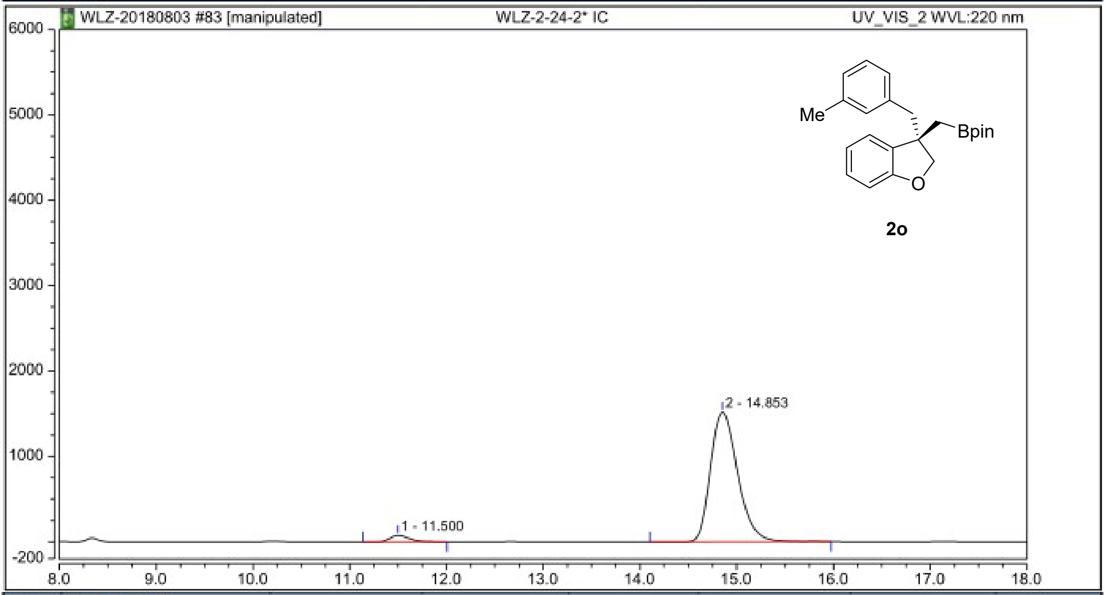


**Integration Results**

| No. | Peak Name | Retention Time min | Area mAU*min | Height mAU | Relative Area % | Relative Height % | Amount n.a. |
|---|---|---|---|---|---|---|---|
| 1 | | 11.500 | 17.135 | 75.749 | 3.35 | 4.76 | n.a. |
| 2 | | 14.853 | 494.869 | 1516.961 | 96.65 | 95.24 | n.a. |
| **Total:** | | | **512.004** | **1592.710** | **100.00** | **100.00** | |

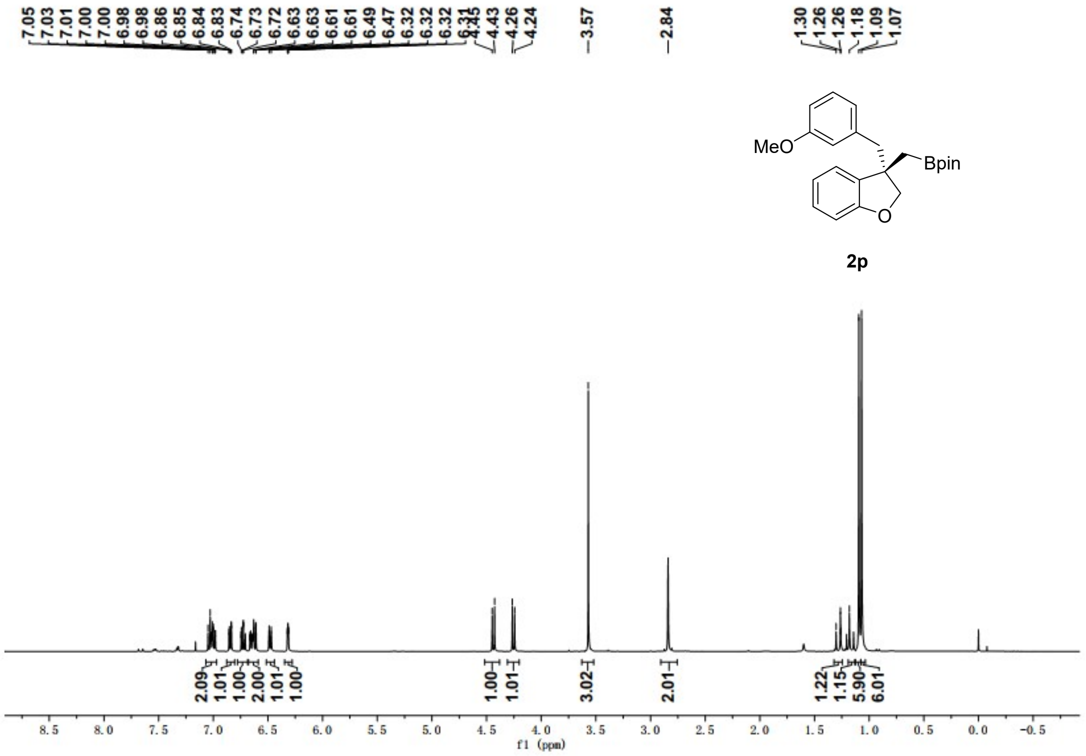
7.05 7.03 7.01 7.00 7.00 6.98 6.98 6.86 6.85 6.84 6.83 6.74 6.73 6.72 6.63 6.63 6.61 6.61 6.49 6.47 6.32 6.32 6.32 6.31 4.45 4.43 4.26 4.24
3.57
2.84
1.30 1.26 1.26 1.18 1.09 1.07
MeO
Bpin
O
2p
2.09 1.01 1.00 2.00 1.01 1.00
1.00 1.01
3.02
2.01
1.22 1.15 5.90 6.01
f1 (ppm)

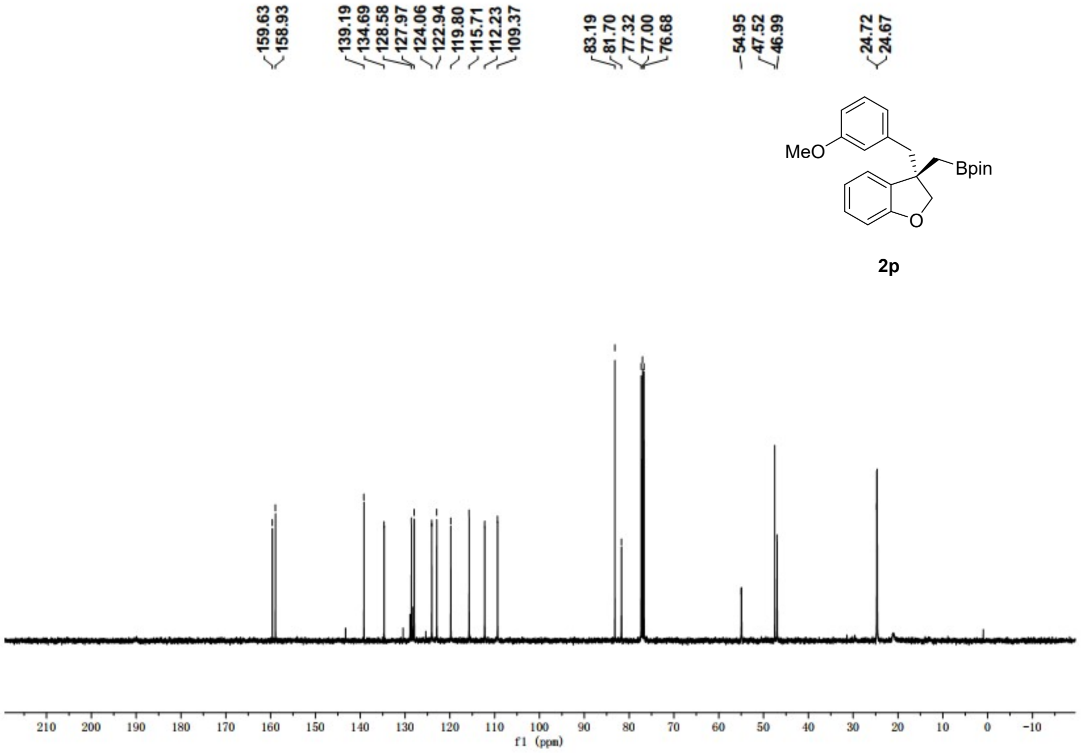
159.63 158.93
139.19 134.69 128.58 127.97 124.06 122.94 119.80 115.71 112.23 109.37
83.19 81.70 77.32 77.00 76.68
54.95 47.52 46.99
24.72 24.67
MeO
Bpin
O
2p
f1 (ppm)

**Chromatogram**

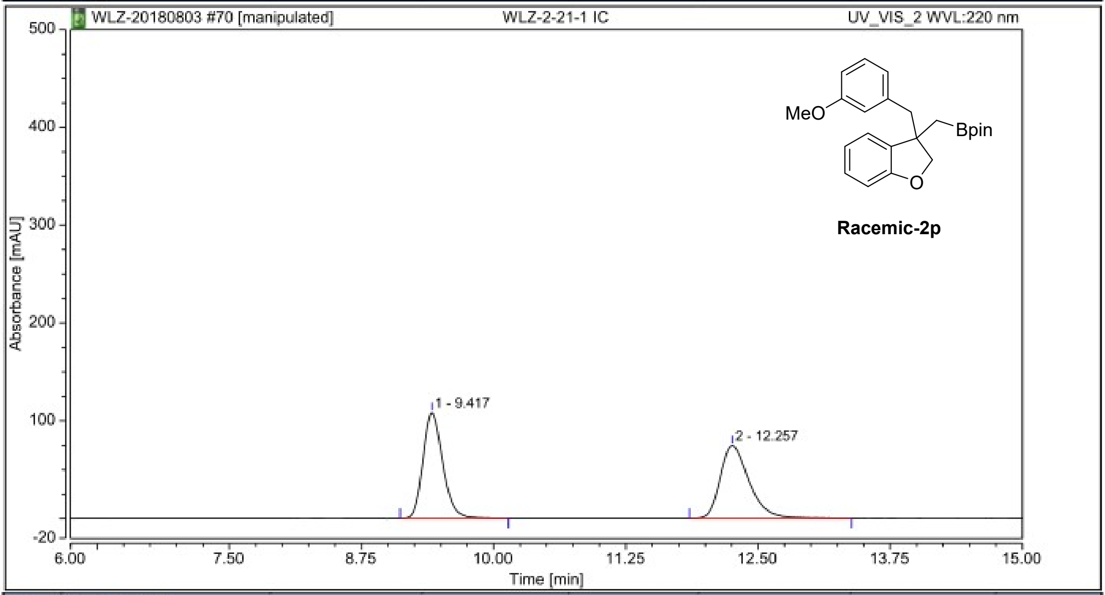


**Integration Results**

| No. | Peak Name | Retention Time min | Area mAU*min | Height mAU | Relative Area % | Relative Height % | Amount n.a. |
|---|---|---|---|---|---|---|---|
| 1 | | 9.417 | 23.532 | 107.978 | 49.83 | 59.21 | n.a. |
| 2 | | 12.257 | 23.696 | 74.381 | 50.17 | 40.79 | n.a. |
| **Total:** | | | **47.229** | **182.359** | **100.00** | **100.00** | |

**Chromatogram**

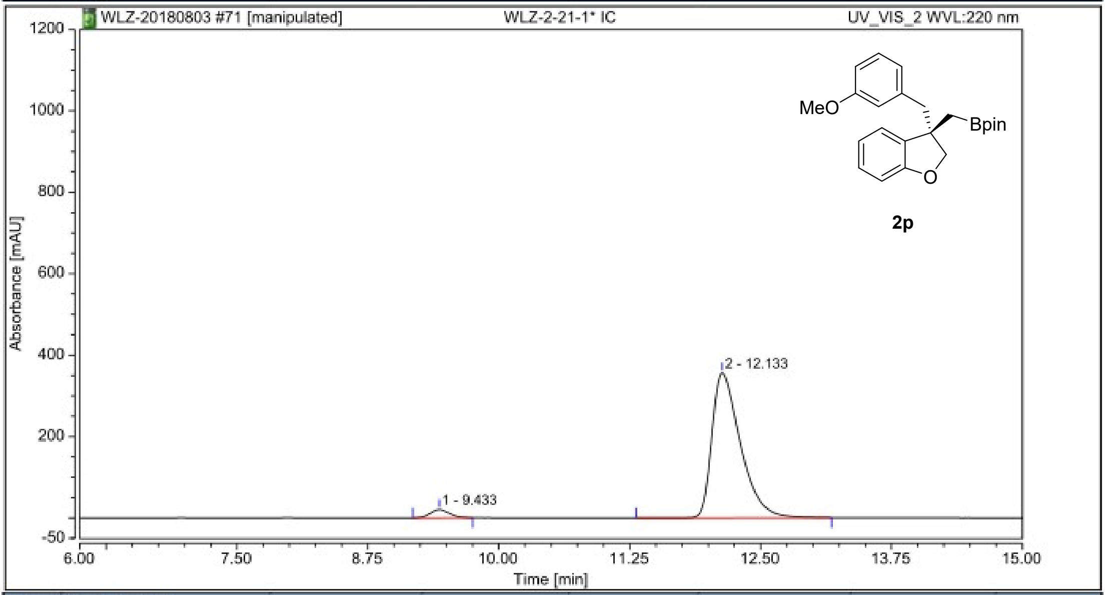


**Integration Results**

| No. | Peak Name | Retention Time min | Area mAU*min | Height mAU | Relative Area % | Relative Height % | Amount n.a. |
|---|---|---|---|---|---|---|---|
| 1 | | 9.433 | 4.260 | 20.257 | 3.65 | 5.38 | n.a. |
| 2 | | 12.133 | 112.354 | 355.933 | 96.35 | 94.62 | n.a. |
| **Total:** | | | **116.614** | **376.190** | **100.00** | **100.00** | |

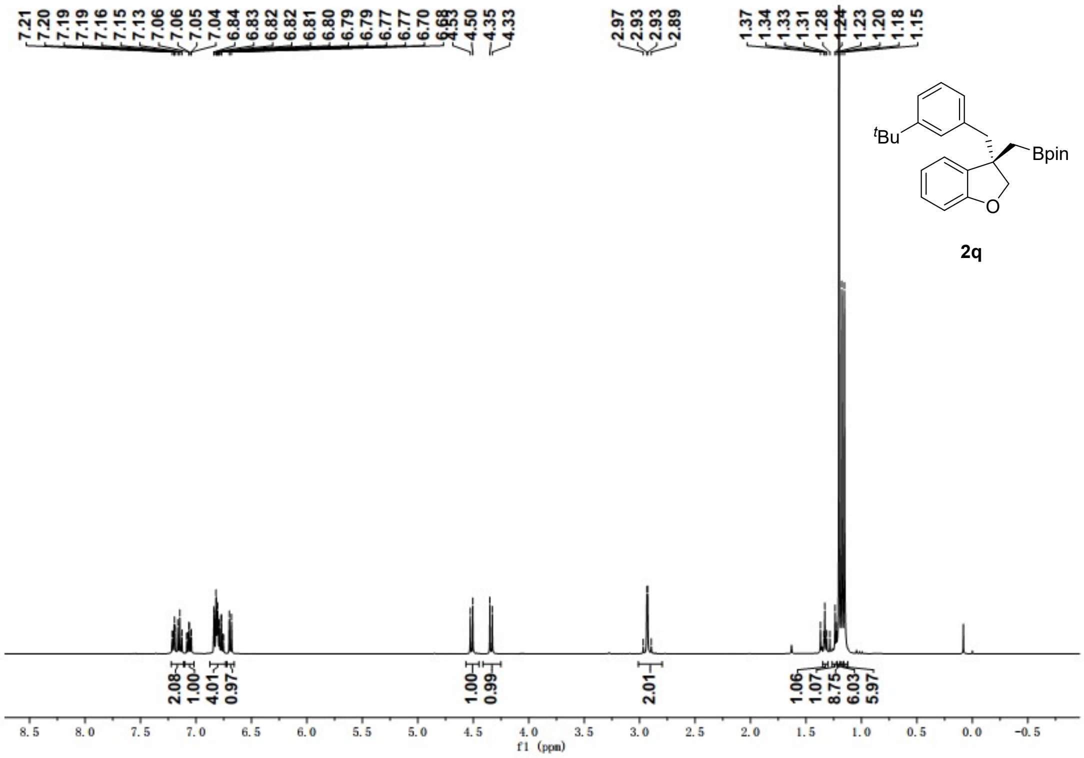
7.21 7.20 7.19 7.19 7.16 7.15 7.13 7.06 7.05 7.04 6.84 6.83 6.82 6.82 6.81 6.80 6.79 6.79 6.77 6.77 6.70 6.68 4.53 4.50 4.35 4.33
2.97 2.93 2.93 2.89
1.37 1.34 1.33 1.31 1.28 1.24 1.23 1.20 1.18 1.15
tBu
Bpin
O
2q
2.08 1.00 4.01 0.97 1.00 0.99 2.01 1.06 1.07 8.75 6.03 5.97
8.5 8.0 7.5 7.0 6.5 6.0 5.5 5.0 4.5 4.0 3.5 3.0 2.5 2.0 1.5 1.0 0.5 0.0 -0.5
f1 (ppm)

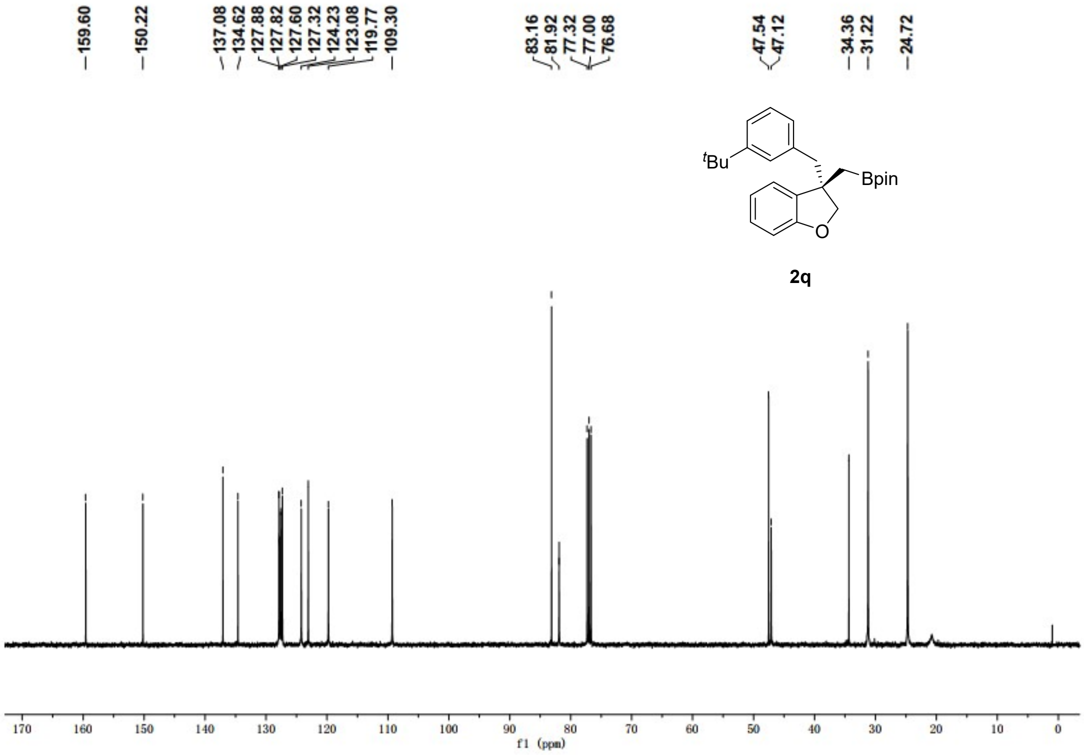
159.60
150.22
137.08 134.62 127.88 127.82 127.60 127.32 124.23 123.08 119.77 109.30
83.16 81.92 77.32 77.00 76.68
47.54 47.12
34.36 31.22 24.72
tBu
Bpin
O
2q
170 160 150 140 130 120 110 100 90 80 70 60 50 40 30 20 10 0
f1 (ppm)

**Chromatogram**

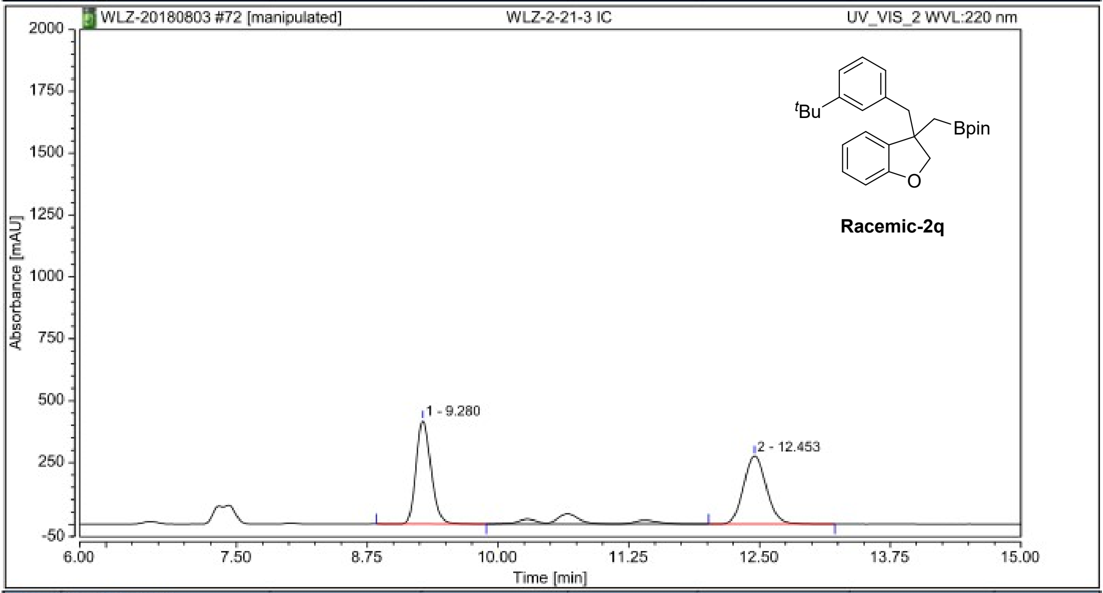


**Integration Results**

| No. | Peak Name | Retention Time min | Area mAU*min | Height mAU | Relative Area % | Relative Height % | Amount n.a. |
|---|---|---|---|---|---|---|---|
| 1 | | 9.280 | 68.817 | 417.182 | 49.77 | 60.34 | n.a. |
| 2 | | 12.453 | 69.448 | 274.232 | 50.23 | 39.66 | n.a. |
| **Total:** | | | **138.266** | **691.414** | **100.00** | **100.00** | |

**Chromatogram**

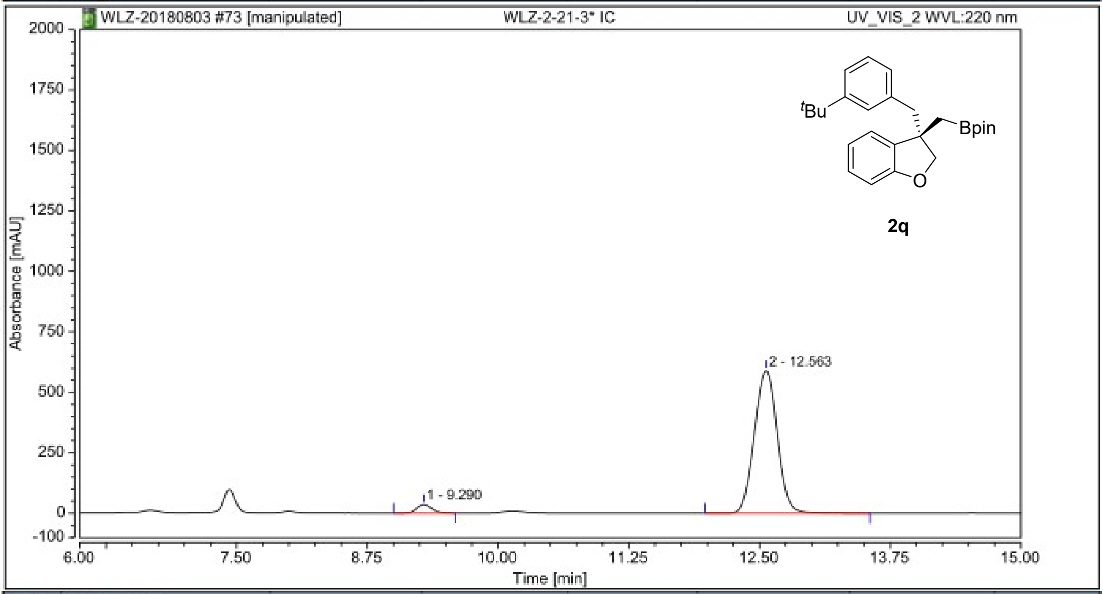


**Integration Results**

| No. | Peak Name | Retention Time min | Area mAU*min | Height mAU | Relative Area % | Relative Height % | Amount n.a. |
|---|---|---|---|---|---|---|---|
| 1 | | 9.290 | 6.145 | 36.716 | 3.94 | 5.86 | n.a. |
| 2 | | 12.563 | 149.734 | 589.390 | 96.06 | 94.14 | n.a. |
| **Total:** | | | **155.879** | **626.106** | **100.00** | **100.00** | |

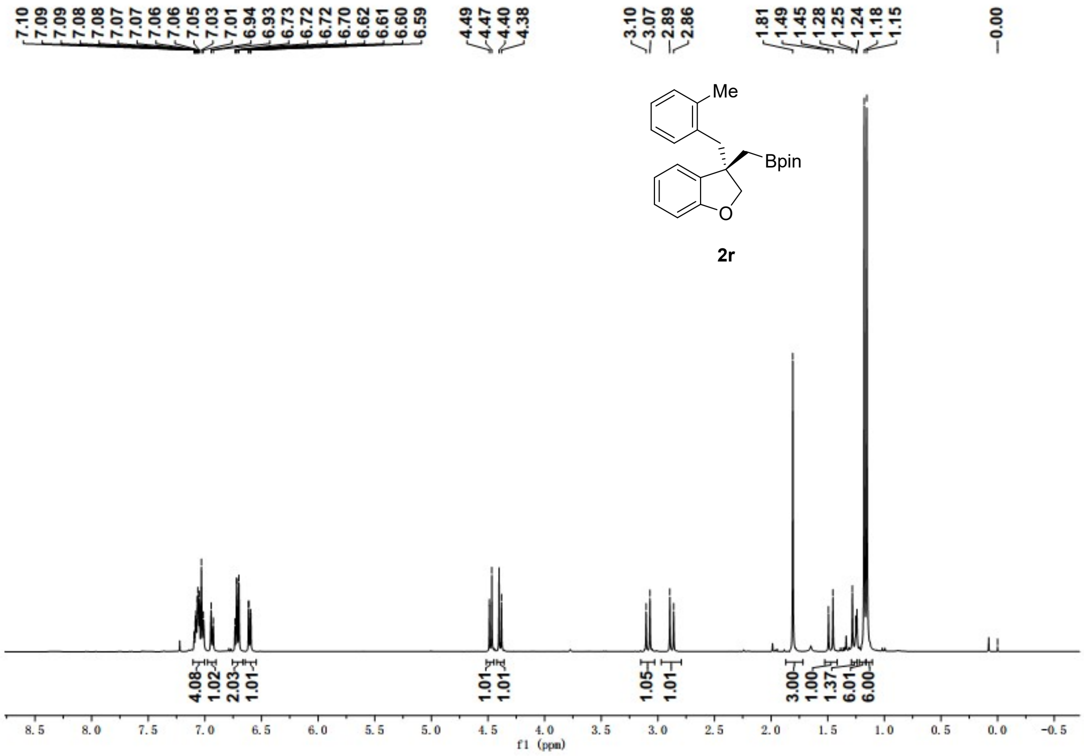
7.10 7.09 7.09 7.08 7.08 7.07 7.07 7.06 7.06 7.05 7.03 7.01 6.94 6.93 6.73 6.72 6.72 6.70 6.62 6.61 6.60 6.59
4.49 4.47 4.40 4.38
3.10 3.07 2.89 2.86
1.81 1.49 1.45 1.28 1.25 1.24 1.18 1.15
0.00
Me
Bpin
O
2r
4.08 1.02 2.03 1.01
1.01 1.01
1.05 1.01
3.00 1.00 1.37 6.01 6.00
8.5 8.0 7.5 7.0 6.5 6.0 5.5 5.0 4.5 4.0 3.5 3.0 2.5 2.0 1.5 1.0 0.5 0.0 -0.5
f1 (ppm)

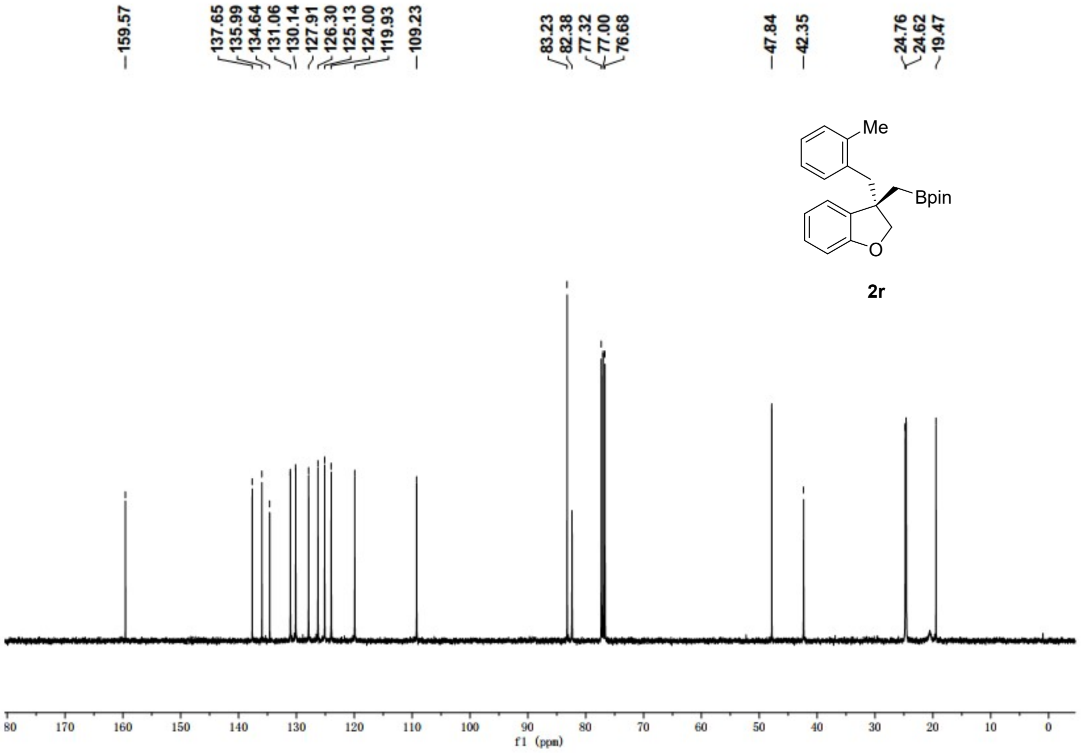
159.57
137.65 135.99 134.64 131.06 130.14 127.91 126.30 125.13 124.00 119.93
109.23
83.23 82.38 77.32 77.00 76.68
47.84
42.35
24.76 24.62 19.47
Me
Bpin
O
2r
170 160 150 140 130 120 110 100 90 80 70 60 50 40 30 20 10 0
f1 (ppm)

## Chromatogram

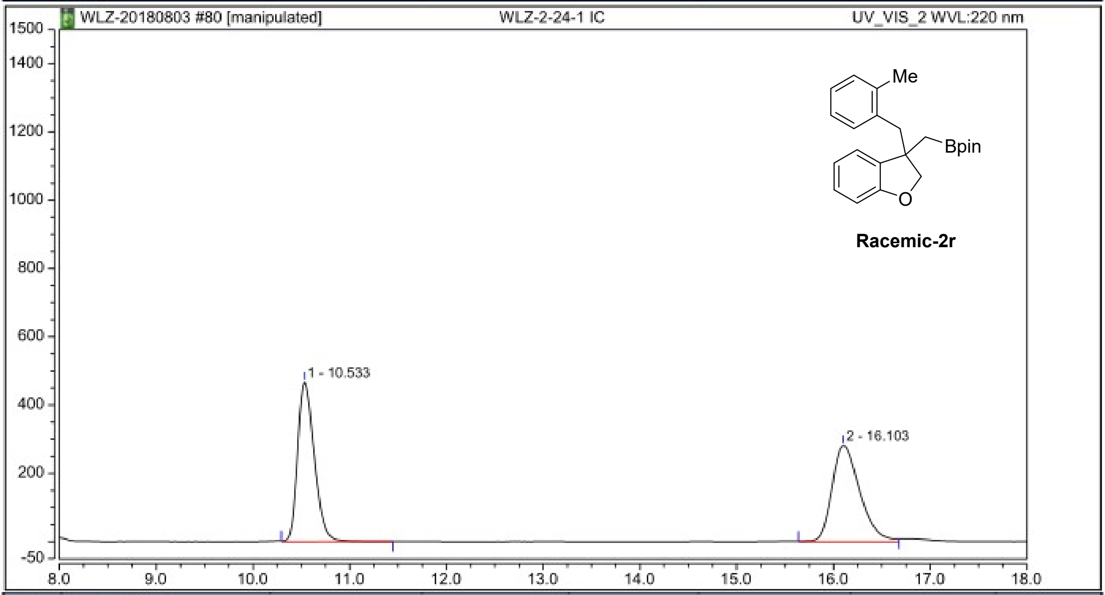


## Integration Results

| No. | Peak Name | Retention Time min | Area mAU*min | Height mAU | Relative Area % | Relative Height % | Amount n.a. |
|---|---|---|---|---|---|---|---|
| 1 | | 10.533 | 93.492 | 467.356 | 49.83 | 62.42 | n.a. |
| 2 | | 16.103 | 94.118 | 281.350 | 50.17 | 37.58 | n.a. |
| **Total:** | | | **187.610** | **748.706** | **100.00** | **100.00** | |

## Chromatogram

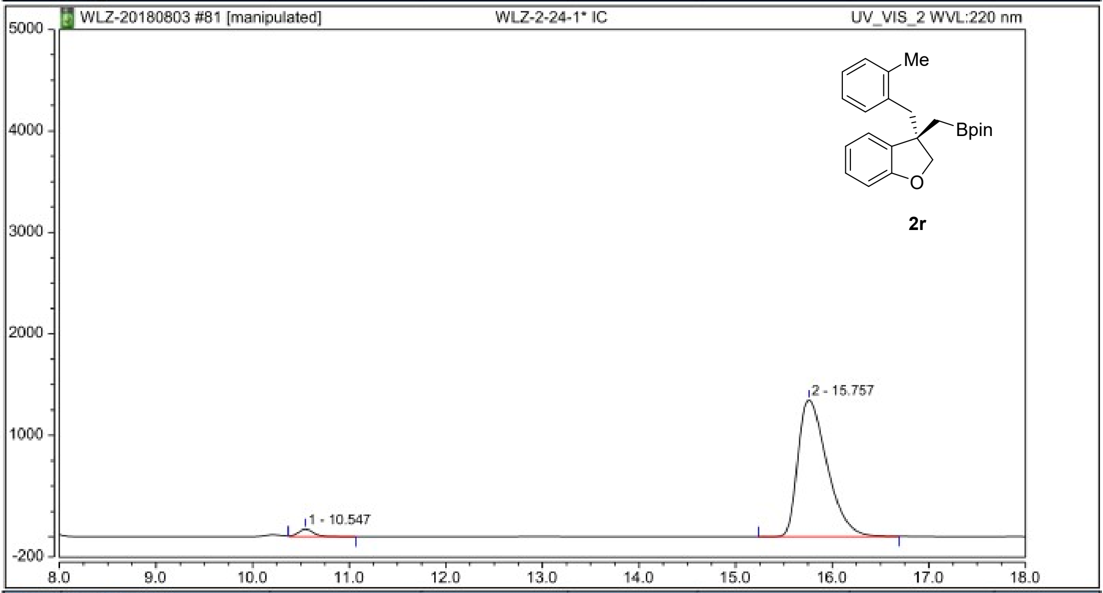


## Integration Results

| No. | Peak Name | Retention Time min | Area mAU*min | Height mAU | Relative Area % | Relative Height % | Amount n.a. |
|---|---|---|---|---|---|---|---|
| 1 | | 10.547 | 14.236 | 75.911 | 2.91 | 5.33 | n.a. |
| 2 | | 15.757 | 475.017 | 1349.233 | 97.09 | 94.67 | n.a. |
| **Total:** | | | **489.253** | **1425.143** | **100.00** | **100.00** | |

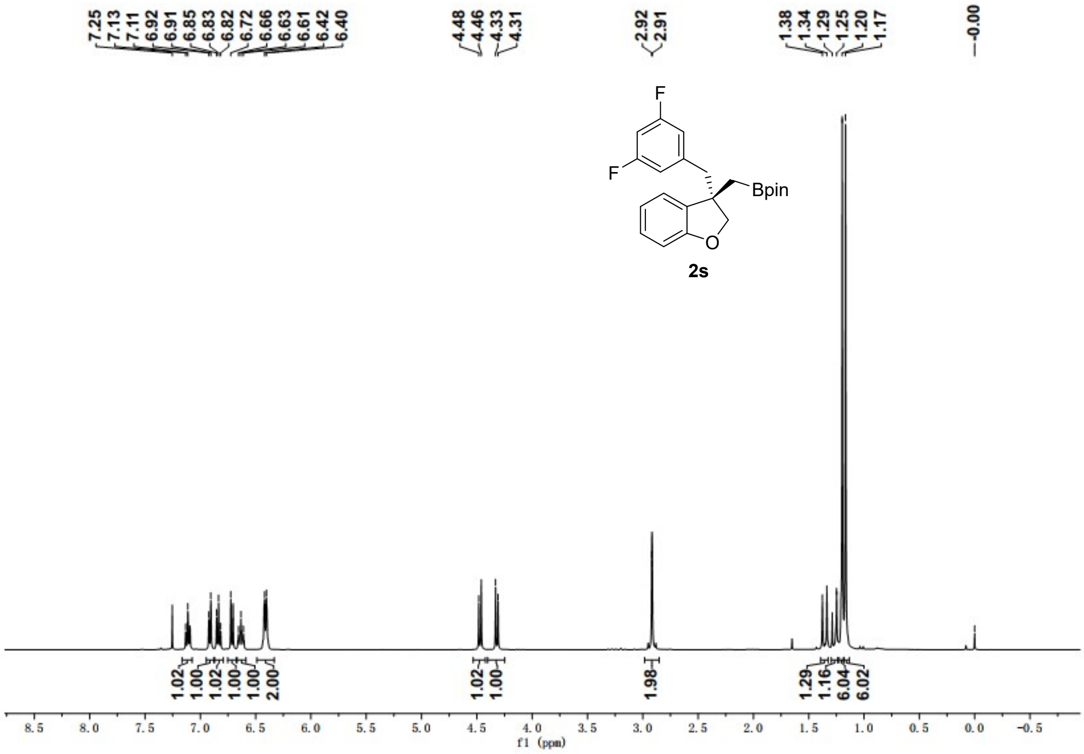
7.25
7.13
7.11
6.92
6.91
6.85
6.83
6.82
6.72
6.66
6.63
6.61
6.42
6.40
4.48
4.46
4.33
4.31
2.92
2.91
1.38
1.34
1.29
1.25
1.20
1.17
-0.00
F
F
Bpin
O
2s
1.02
1.00
1.02
1.00
1.00
2.00
1.02
1.00
1.98
1.29
1.16
6.04
6.02
f1 (ppm)

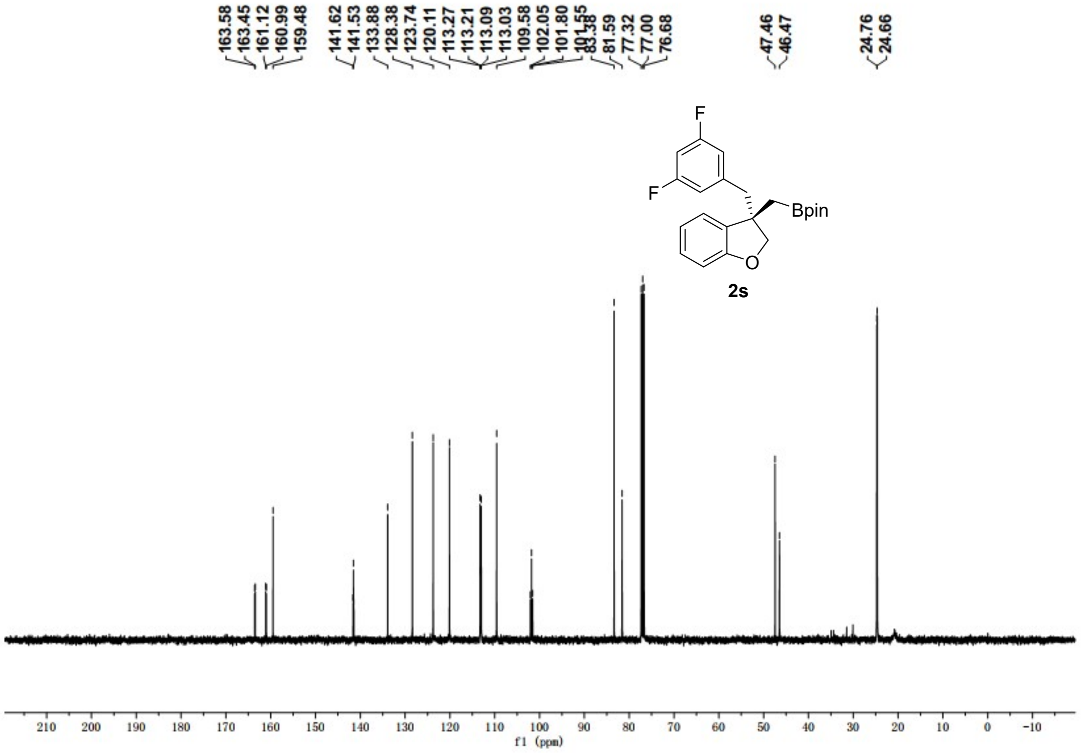
163.58
163.45
161.12
160.99
159.48
141.62
141.53
133.88
128.38
123.74
120.11
113.27
113.21
113.09
113.03
109.58
102.05
101.80
101.55
83.38
81.59
77.32
77.00
76.68
47.46
46.47
24.76
24.66
F
F
Bpin
O
2s
f1 (ppm)

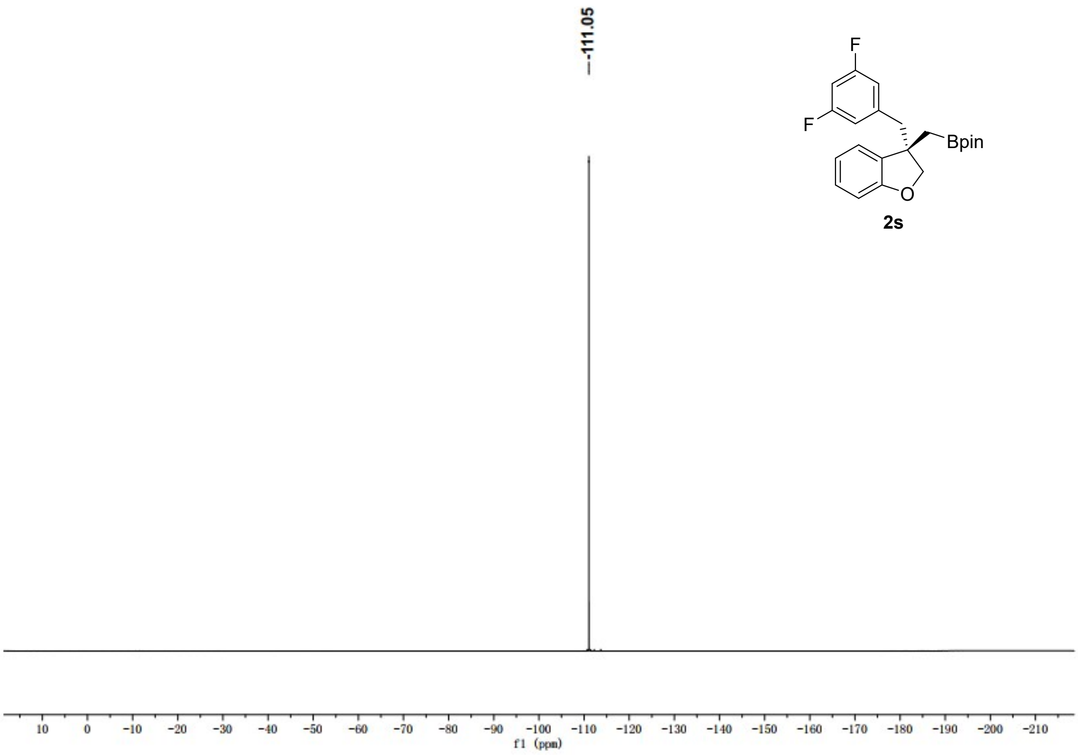


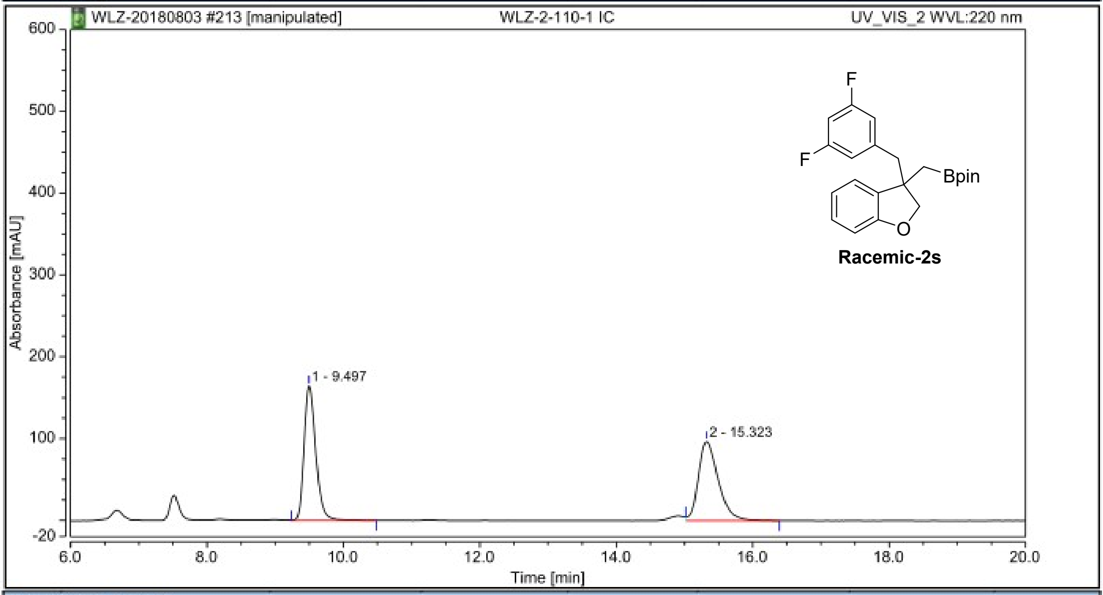


**Integration Results**

| No. | Peak Name | Retention Time min | Area mAU*min | Height mAU | Relative Area % | Relative Height % | Amount n.a. |
|---|---|---|---|---|---|---|---|
| 1 | | 9.497 | 32.681 | 164.554 | 49.82 | 62.94 | n.a. |
| 2 | | 15.323 | 32.923 | 96.911 | 50.18 | 37.06 | n.a. |
| **Total:** | | | **65.605** | **261.465** | **100.00** | **100.00** | |

## Chromatogram

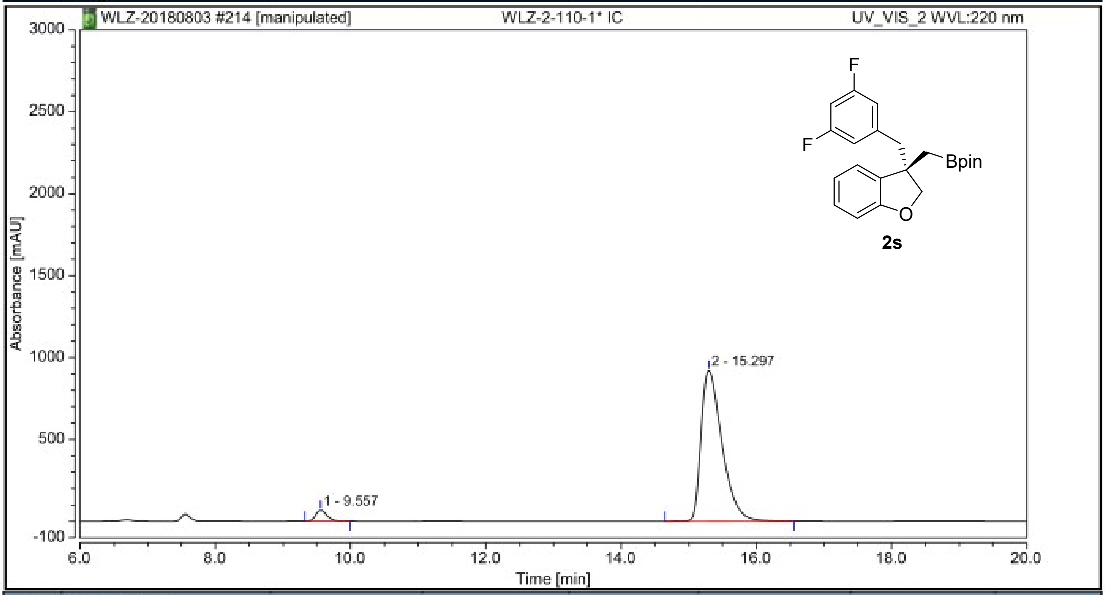


## Integration Results

| No. | Peak Name | Retention Time min | Area mAU*min | Height mAU | Relative Area % | Relative Height % | Amount n.a. |
|---|---|---|---|---|---|---|---|
| 1 | | 9.557 | 13.353 | 67.181 | 3.88 | 6.80 | n.a. |
| 2 | | 15.297 | 331.060 | 920.485 | 96.12 | 93.20 | n.a. |
| **Total:** | | | **344.413** | **987.666** | **100.00** | **100.00** | |

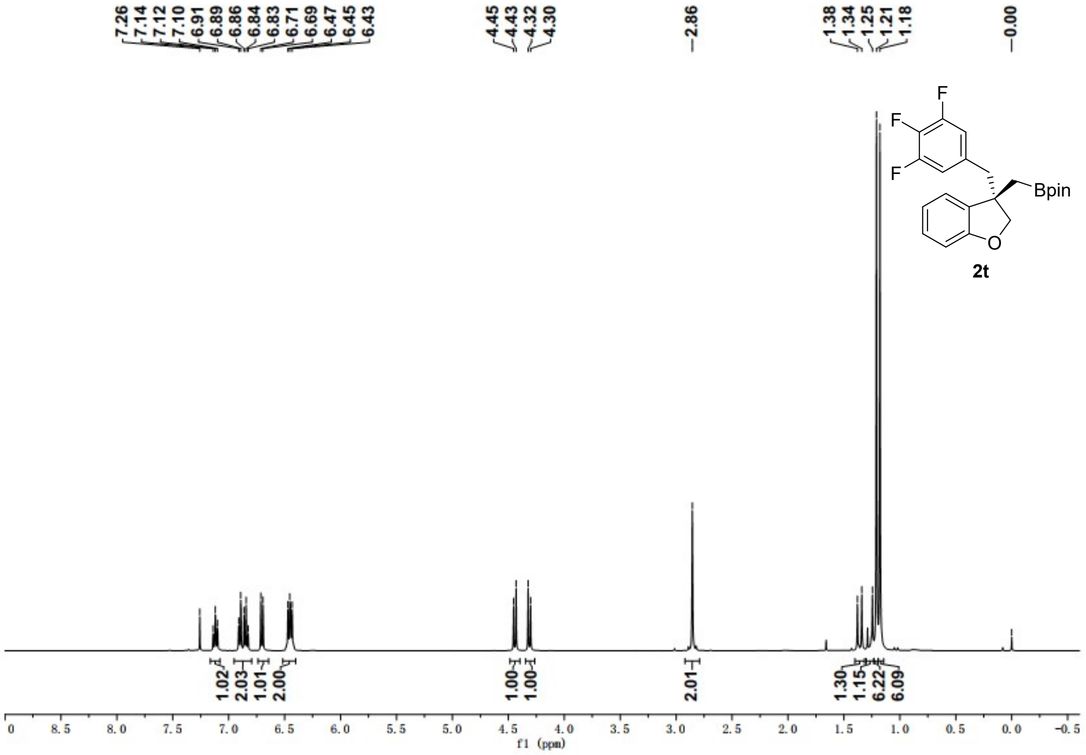

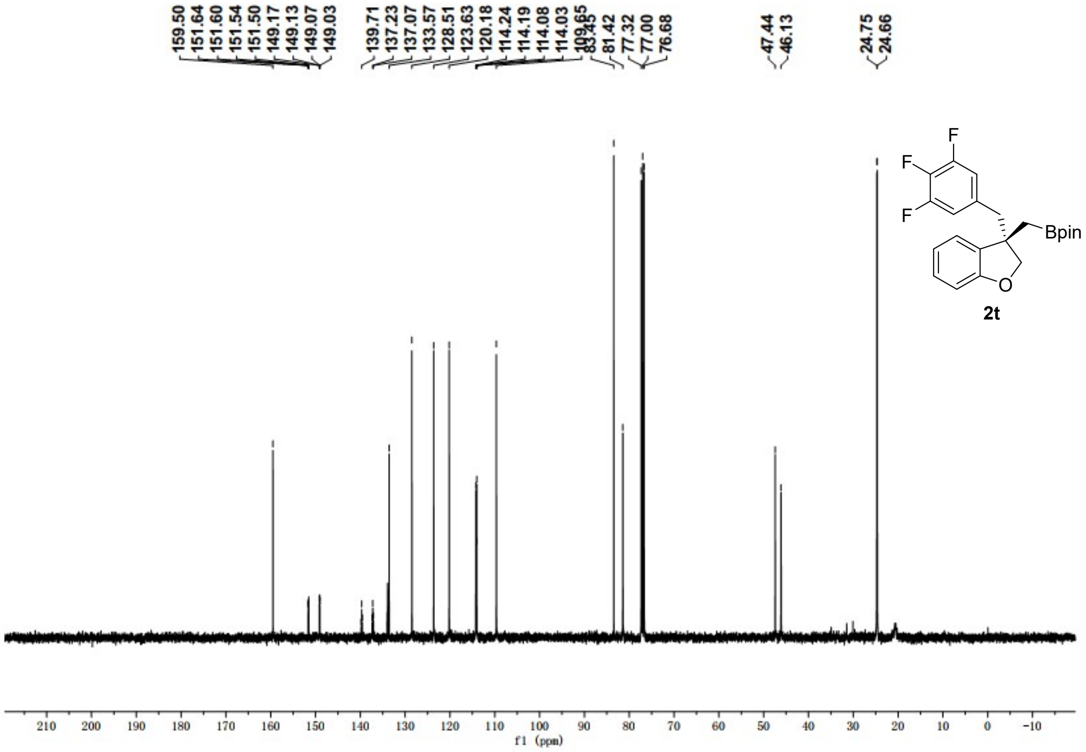
159.50
151.64
151.60
151.54
151.50
149.17
149.13
149.07
149.03
139.71
137.23
137.07
133.57
128.51
123.63
120.18
114.24
114.19
114.08
114.03
109.65
83.45
81.42
77.32
77.00
76.68
47.44
46.13
24.75
24.66
F
F
F
Bpin
O
2t
f1 (ppm)

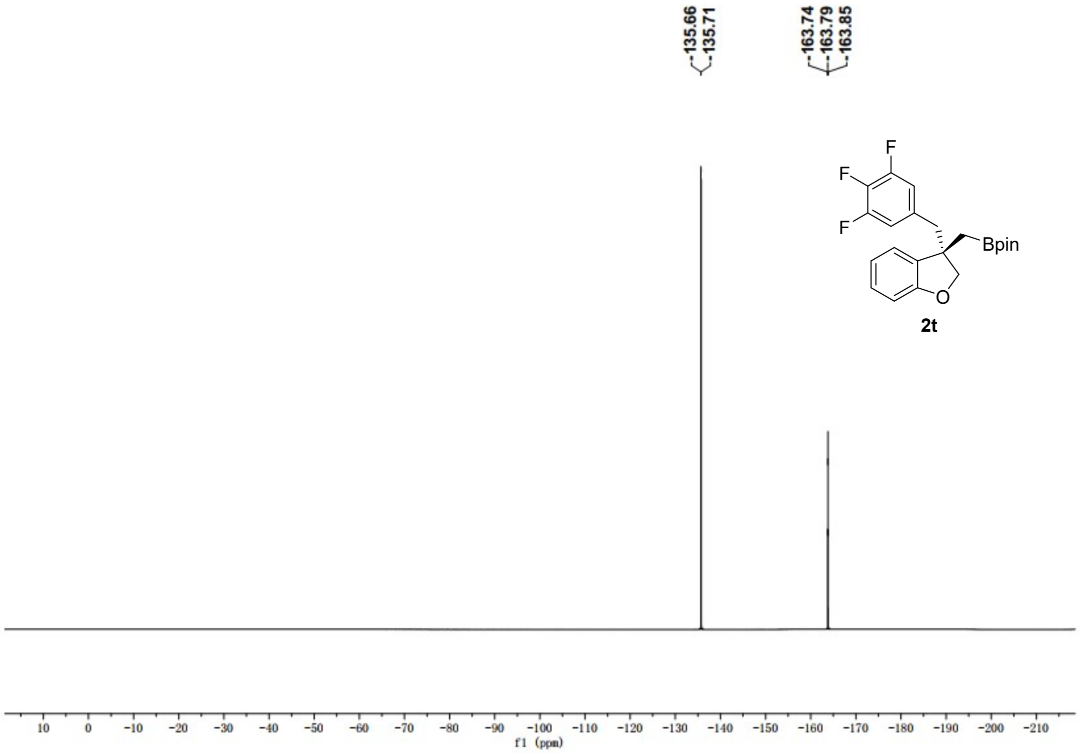
-135.66
-135.71
-163.74
-163.79
-163.85
F
F
F
Bpin
O
2t
f1 (ppm)

## Chromatogram

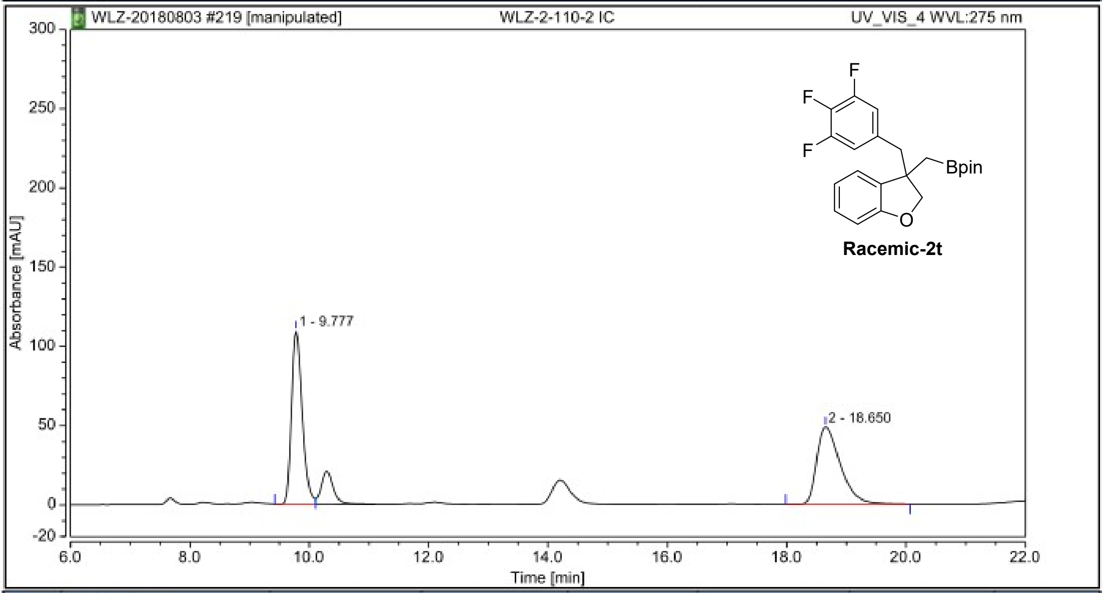


## Integration Results

| No. | Peak Name | Retention Time<br>min | Area<br>mAU*min | Height<br>mAU | Relative Area<br>% | Relative Height<br>% | Amount<br>n.a. |
|---|---|---|---|---|---|---|---|
| 1 | | 9.777 | 22.689 | 109.131 | 50.53 | 69.18 | n.a. |
| 2 | | 18.650 | 22.215 | 48.607 | 49.47 | 30.82 | n.a. |
| **Total:** | | | **44.904** | **157.739** | **100.00** | **100.00** | |

## Chromatogram

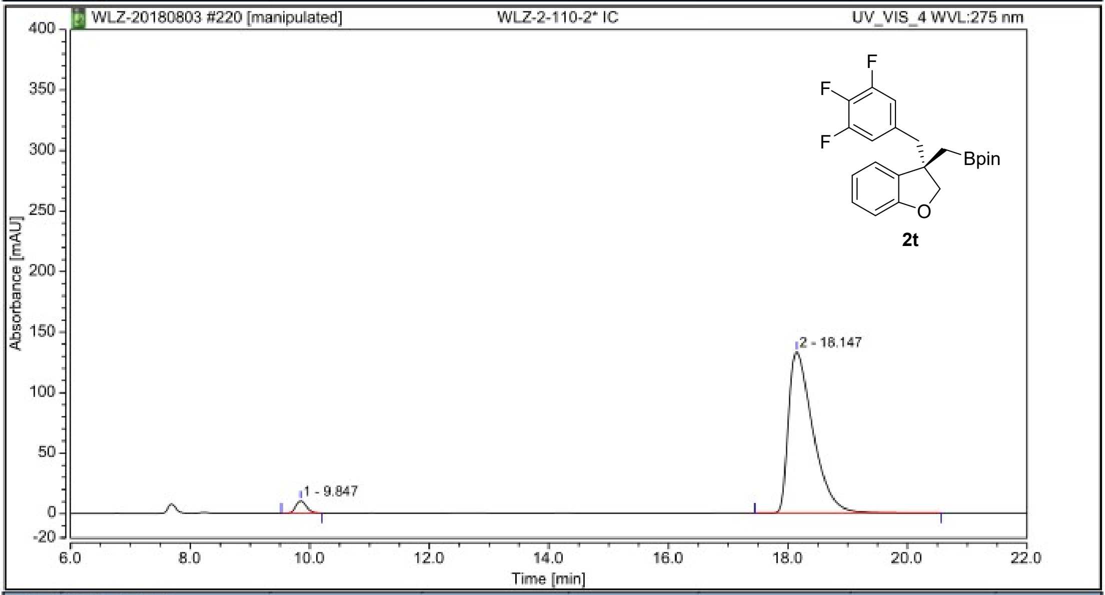


## Integration Results

| No. | Peak Name | Retention Time<br>min | Area<br>mAU*min | Height<br>mAU | Relative Area<br>% | Relative Height<br>% | Amount<br>n.a. |
|---|---|---|---|---|---|---|---|
| 1 | | 9.847 | 2.137 | 10.206 | 3.25 | 7.12 | n.a. |
| 2 | | 18.147 | 63.585 | 133.045 | 96.75 | 92.88 | n.a. |
| **Total:** | | | **65.722** | **143.251** | **100.00** | **100.00** | |

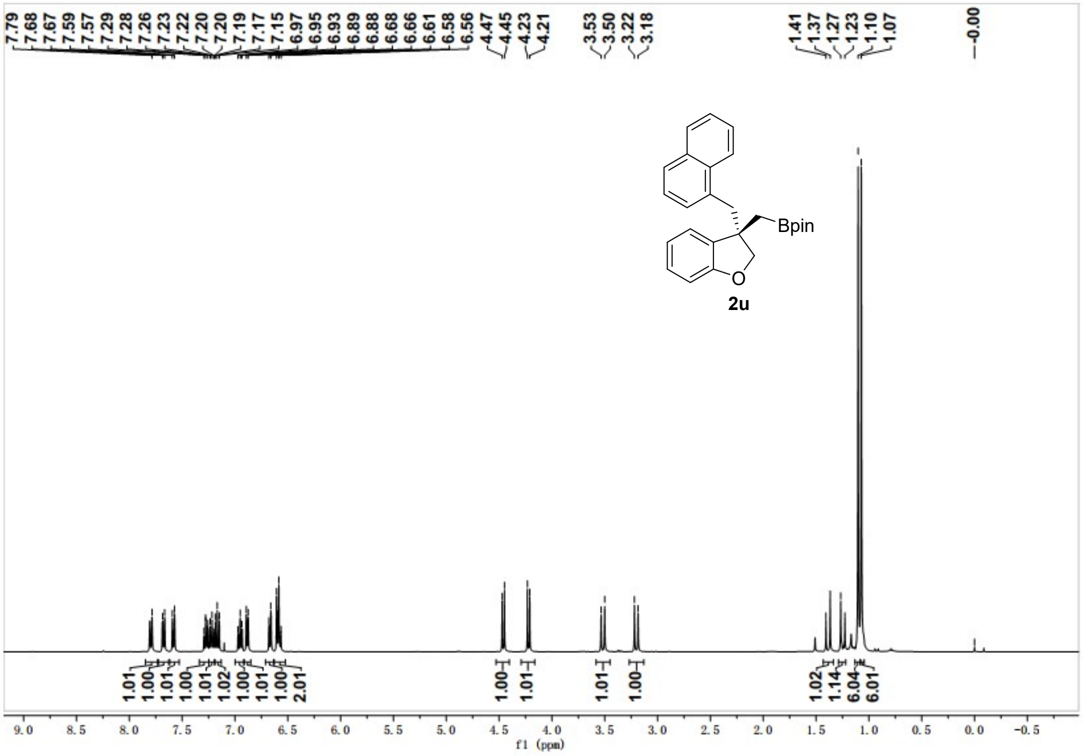
7.79 7.68 7.67 7.59 7.57 7.29 7.28 7.26 7.23 7.22 7.20 7.20 7.19 7.17 7.15 6.97 6.95 6.93 6.89 6.88 6.68 6.66 6.61 6.58 6.56 4.47 4.45 4.23 4.21 3.53 3.50 3.22 3.18 1.41 1.37 1.27 1.23 1.10 1.07 -0.00
Bpin
O
2u
1.01 1.00 1.01 1.00 1.01 1.02 1.00 1.01 1.00 2.01 1.00 1.01 1.01 1.00 1.02 1.14 6.04 6.01
f1 (ppm)

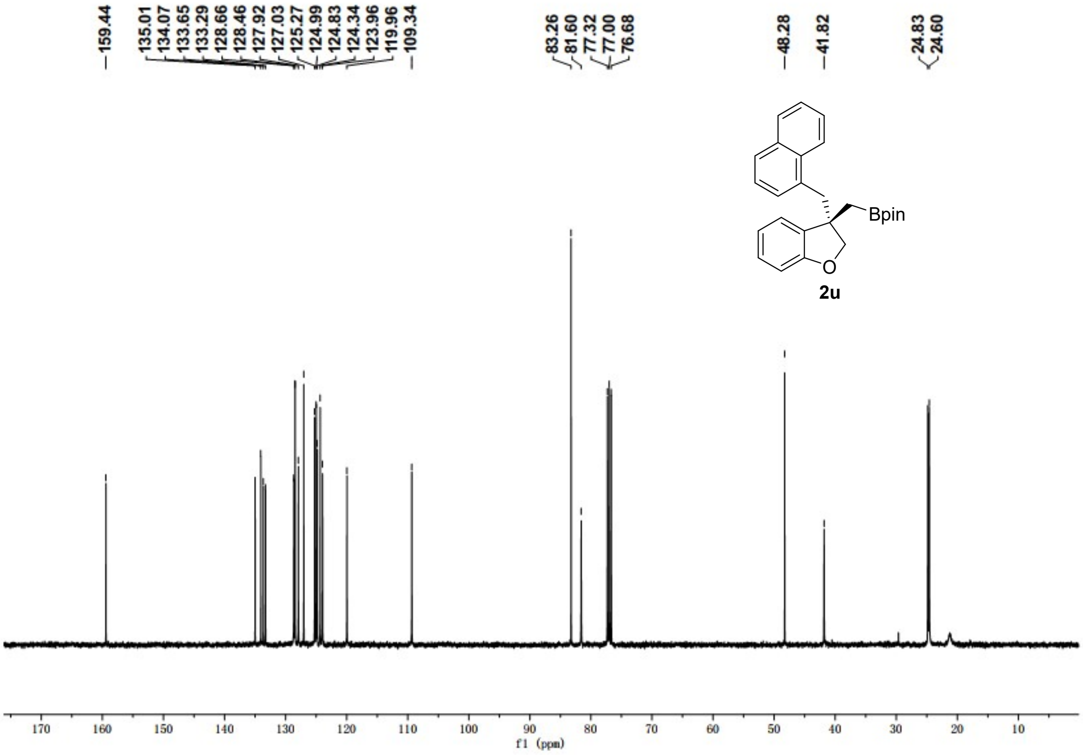
159.44 135.01 134.07 133.65 133.29 128.66 128.46 127.92 127.03 125.27 124.99 124.83 124.34 123.96 119.96 109.34 83.26 81.60 77.32 77.00 76.68 48.28 41.82 24.83 24.60
Bpin
O
2u
f1 (ppm)

## Chromatogram

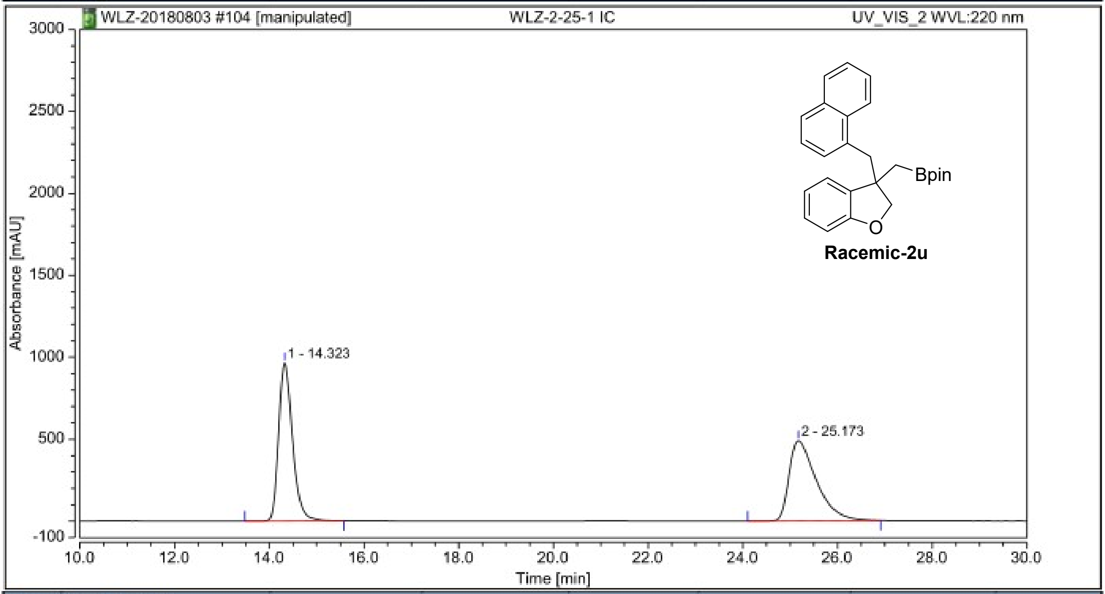


## Integration Results

| No. | Peak Name | Retention Time<br>min | Area<br>mAU*min | Height<br>mAU | Relative Area<br>% | Relative Height<br>% | Amount<br>n.a. |
|---|---|---|---|---|---|---|---|
| 1 | | 14.323 | 326.097 | 965.894 | 49.99 | 66.38 | n.a. |
| 2 | | 25.173 | 326.220 | 489.300 | 50.01 | 33.62 | n.a. |
| **Total:** | | | **652.317** | **1455.193** | **100.00** | **100.00** | |

## Chromatogram

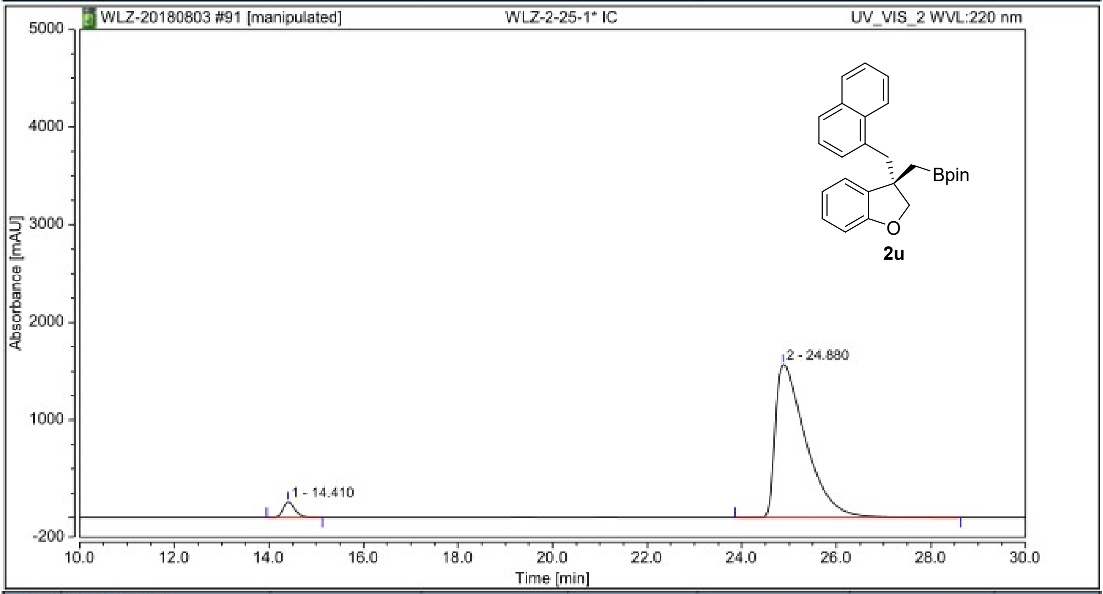


## Integration Results

| No. | Peak Name | Retention Time<br>min | Area<br>mAU*min | Height<br>mAU | Relative Area<br>% | Relative Height<br>% | Amount<br>n.a. |
|---|---|---|---|---|---|---|---|
| 1 | | 14.410 | 47.754 | 159.764 | 3.89 | 9.24 | n.a. |
| 2 | | 24.880 | 1179.456 | 1568.631 | 96.11 | 90.76 | n.a. |
| **Total:** | | | **1227.210** | **1728.395** | **100.00** | **100.00** | |

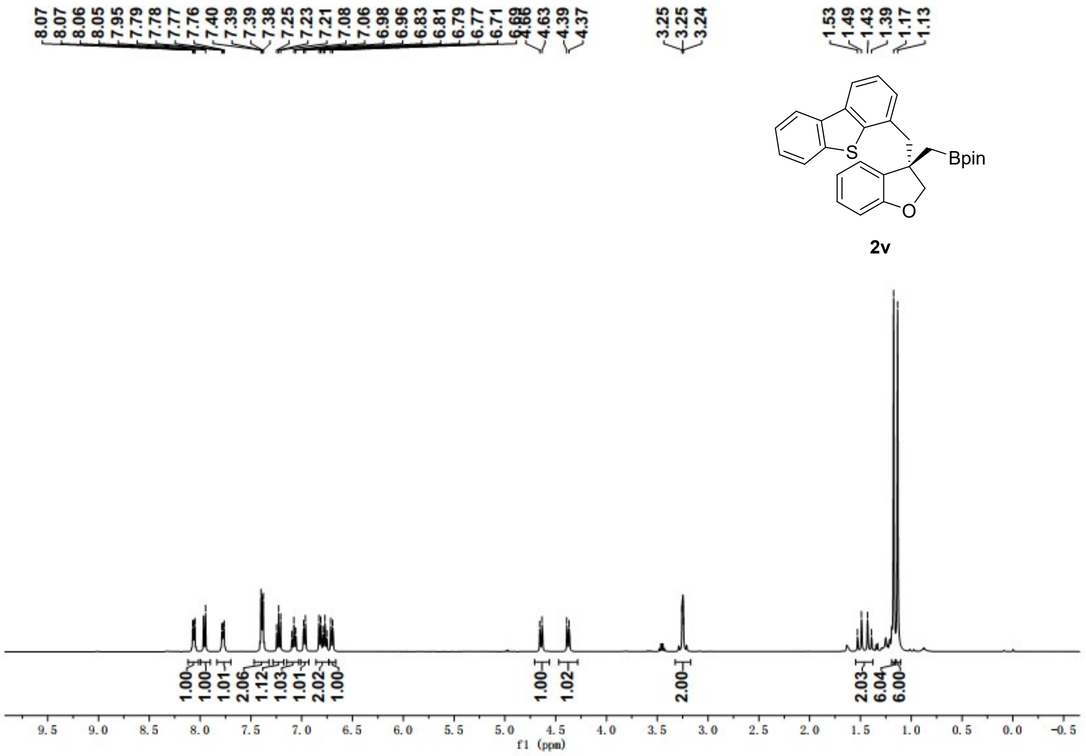
S
O
Bpin
2v
f1 (ppm)

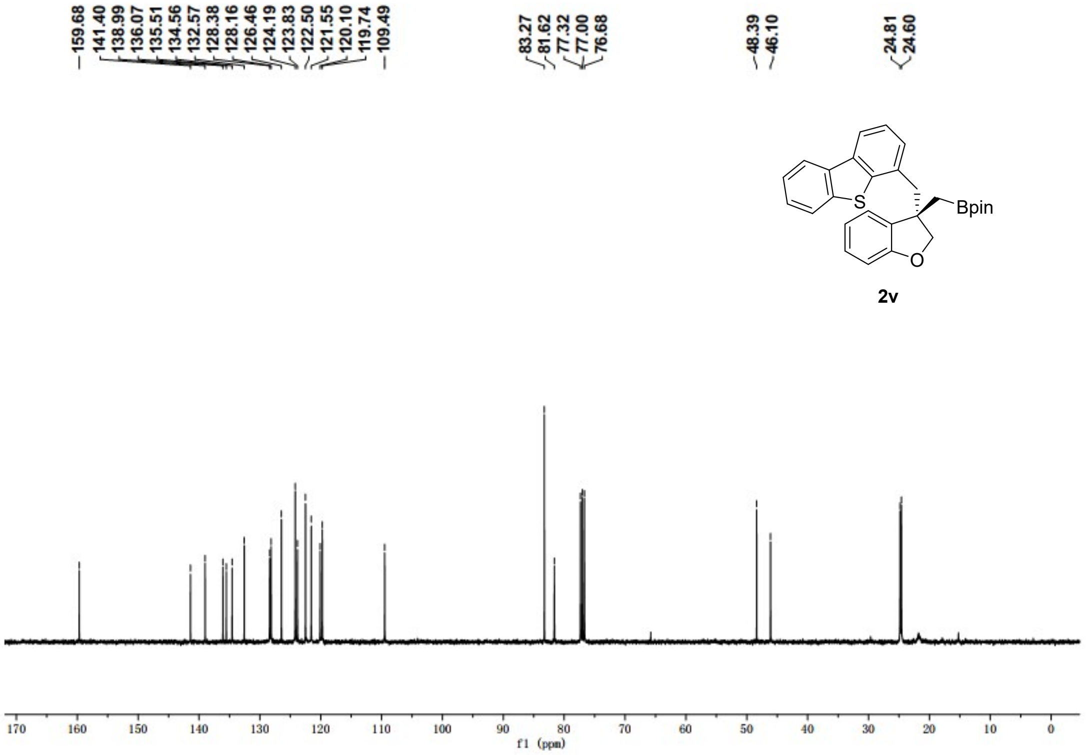
S
O
Bpin
2v
f1 (ppm)

## Chromatogram

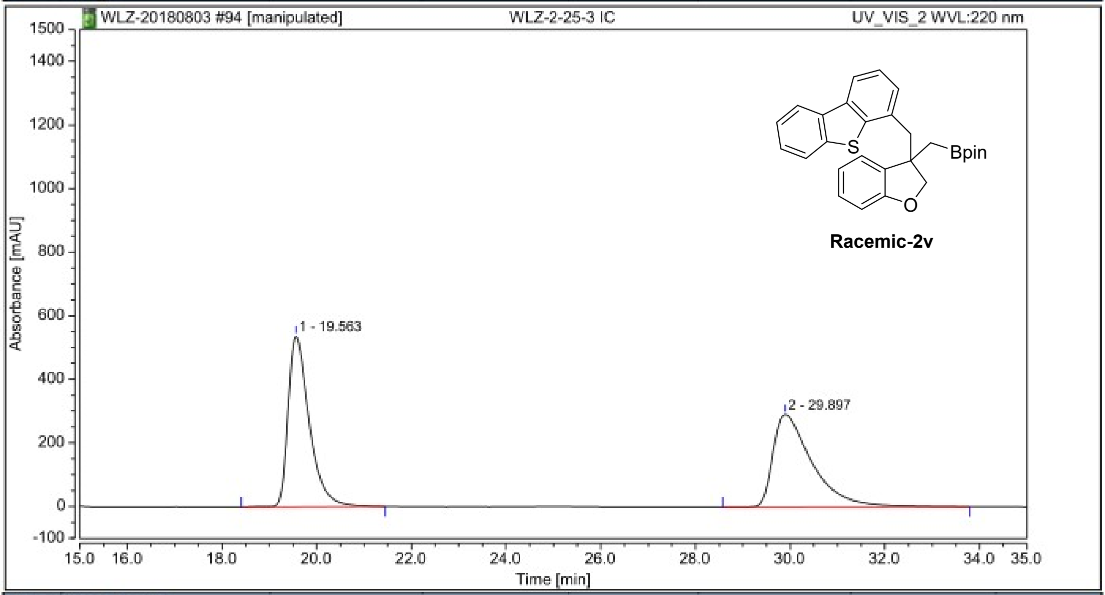


## Integration Results

| No. | Peak Name | Retention Time min | Area mAU*min | Height mAU | Relative Area % | Relative Height % | Amount n.a. |
|---|---|---|---|---|---|---|---|
| 1 | | 19.563 | 278.566 | 537.468 | 50.04 | 64.88 | n.a. |
| 2 | | 29.897 | 278.174 | 290.943 | 49.96 | 35.12 | n.a. |
| **Total:** | | | **556.740** | **828.411** | **100.00** | **100.00** | |

## Chromatogram

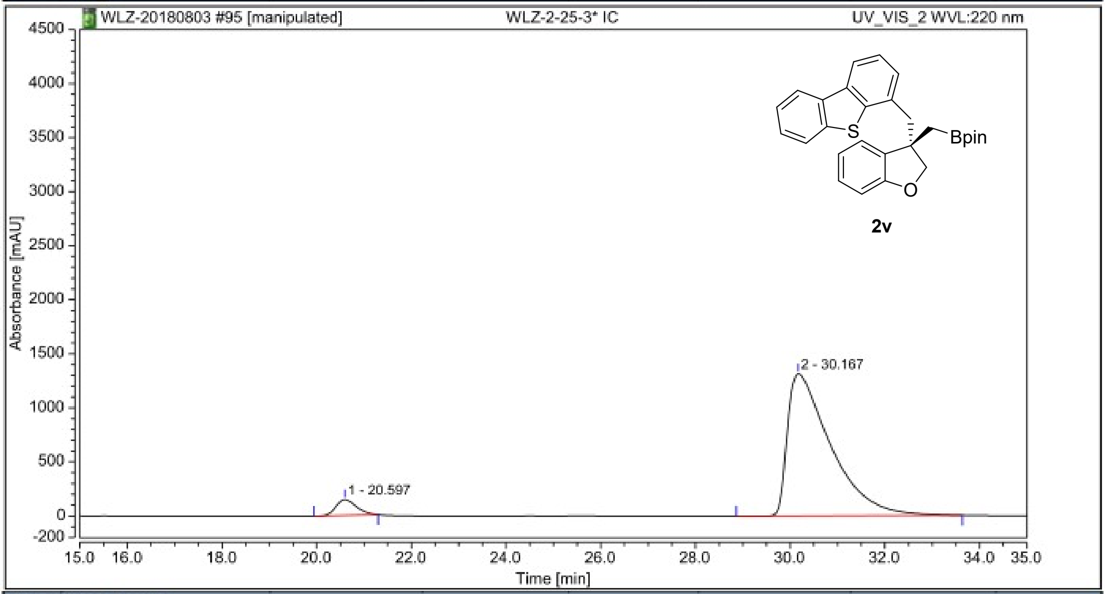


## Integration Results

| No. | Peak Name | Retention Time min | Area mAU*min | Height mAU | Relative Area % | Relative Height % | Amount n.a. |
|---|---|---|---|---|---|---|---|
| 1 | | 20.597 | 72.611 | 144.611 | 4.97 | 9.92 | n.a. |
| 2 | | 30.167 | 1388.033 | 1313.528 | 95.03 | 90.08 | n.a. |
| **Total:** | | | **1460.644** | **1458.138** | **100.00** | **100.00** | |

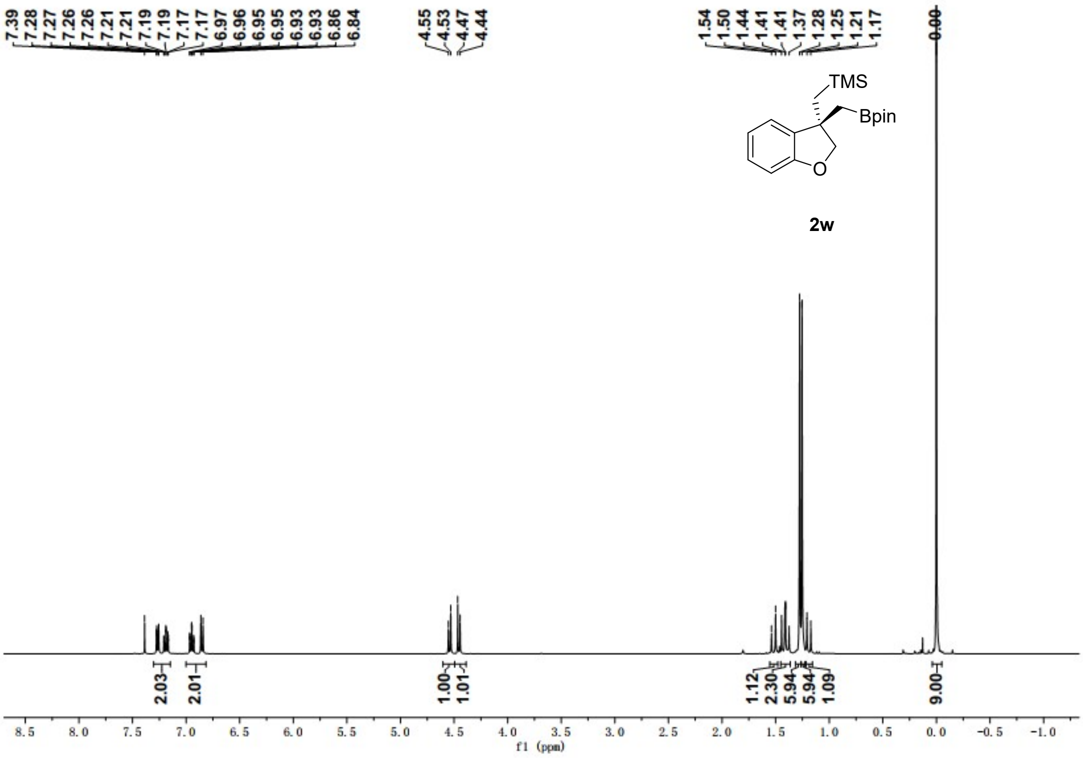
7.39
7.28
7.27
7.26
7.26
7.21
7.21
7.19
7.17
6.97
6.96
6.95
6.95
6.93
6.93
6.86
6.84
4.55
4.53
4.47
4.44
1.54
1.50
1.44
1.41
1.41
1.37
1.28
1.25
1.21
1.17
0.00
TMS
Bpin
O
2w
2.03
2.01
1.00
1.01
1.12
2.30
5.94
5.94
1.09
9.00
f1 (ppm)

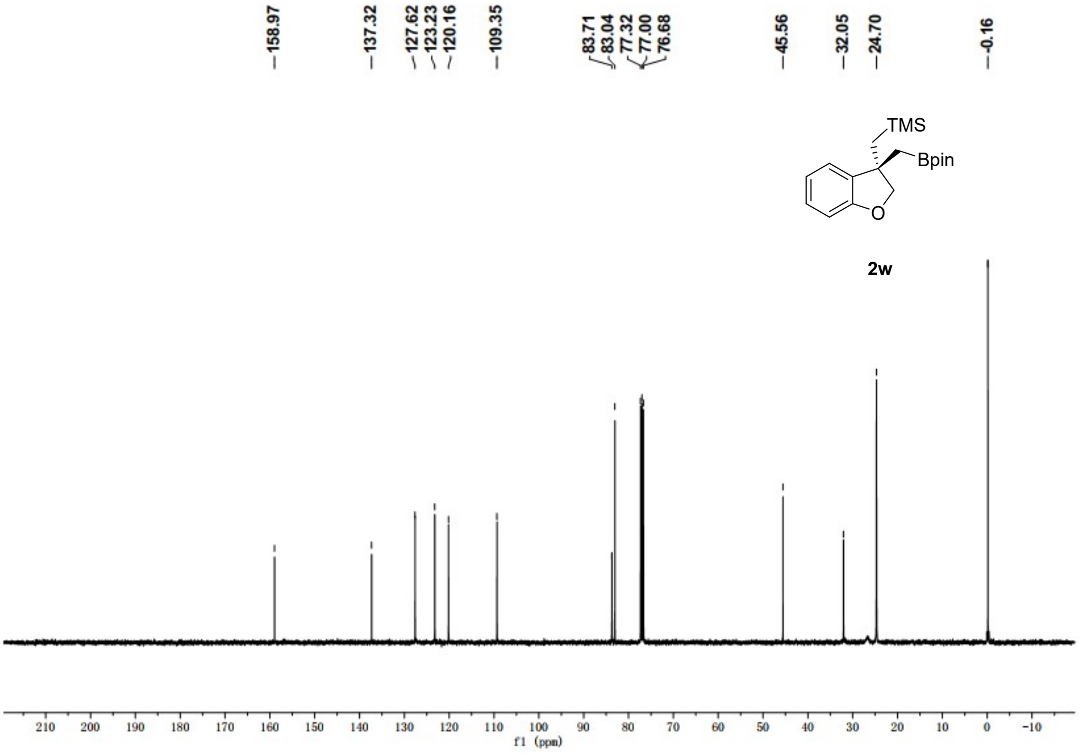
158.97
137.32
127.62
123.23
120.16
109.35
83.71
83.04
77.32
77.00
76.68
45.56
32.05
24.70
0.16
TMS
Bpin
O
2w
f1 (ppm)

## Chromatogram

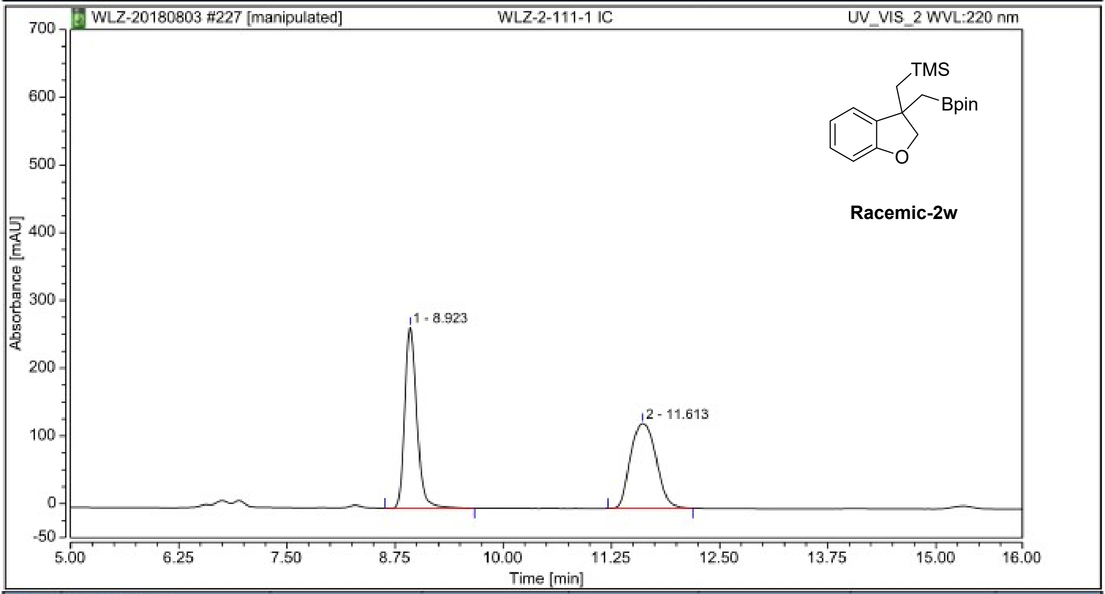


## Integration Results

| No. | Peak Name | Retention Time<br>min | Area<br>mAU*min | Height<br>mAU | Relative Area<br>% | Relative Height<br>% | Amount<br>n.a. |
|---|---|---|---|---|---|---|---|
| 1 | | 8.923 | 43.235 | 266.514 | 50.05 | 68.07 | n.a. |
| 2 | | 11.613 | 43.141 | 124.993 | 49.95 | 31.93 | n.a. |
| **Total:** | | | **86.375** | **391.507** | **100.00** | **100.00** | |

## Chromatogram

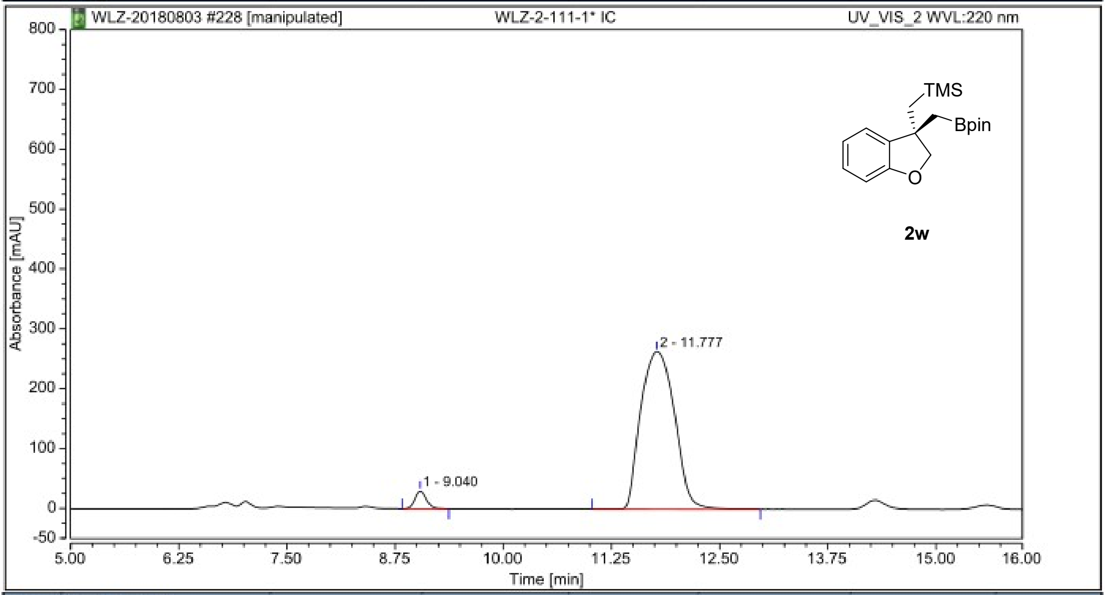


## Integration Results

| No. | Peak Name | Retention Time<br>min | Area<br>mAU*min | Height<br>mAU | Relative Area<br>% | Relative Height<br>% | Amount<br>n.a. |
|---|---|---|---|---|---|---|---|
| 1 | | 9.040 | 4.603 | 29.431 | 3.72 | 10.06 | n.a. |
| 2 | | 11.777 | 119.180 | 263.050 | 96.28 | 89.94 | n.a. |
| **Total:** | | | **123.783** | **292.481** | **100.00** | **100.00** | |

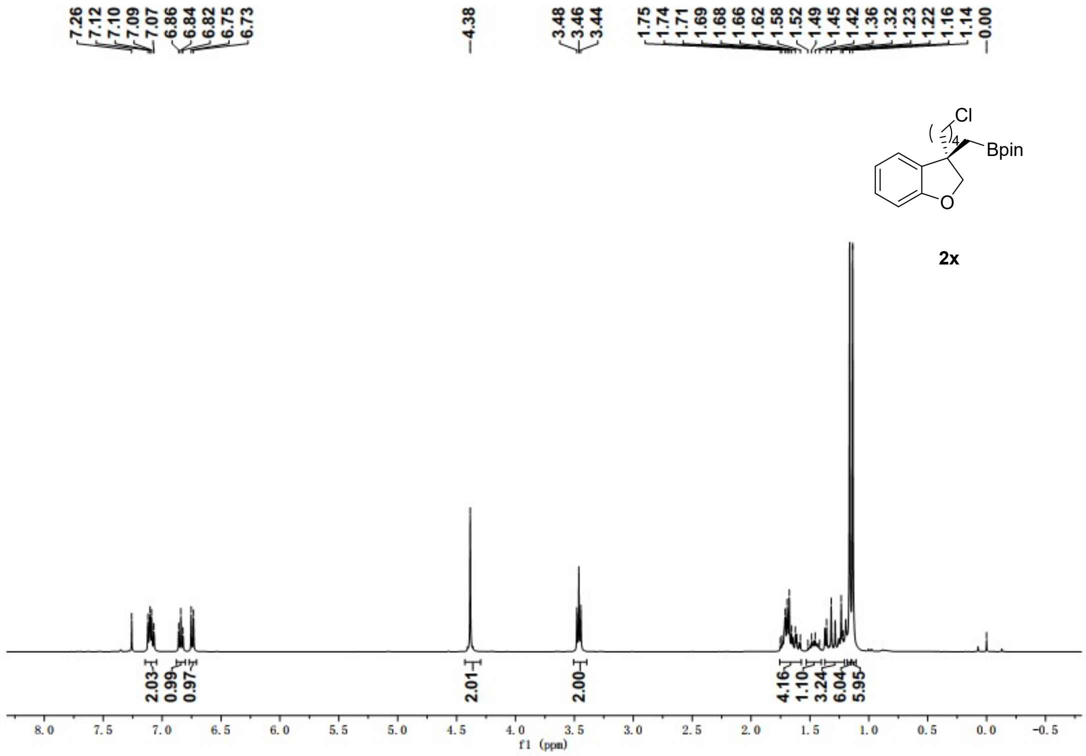
7.26 7.12 7.10 7.09 7.07 6.86 6.84 6.82 6.75 6.73
4.38
3.48 3.46 3.44
1.75 1.74 1.71 1.69 1.68 1.66 1.62 1.58 1.52 1.49 1.45 1.42 1.36 1.32 1.23 1.22 1.16 1.14
0.00
Cl
Bpin
O
2.03 0.99 0.97
2.01
2.00
4.16 1.10 3.24 6.04 5.95
f1 (ppm)

2x

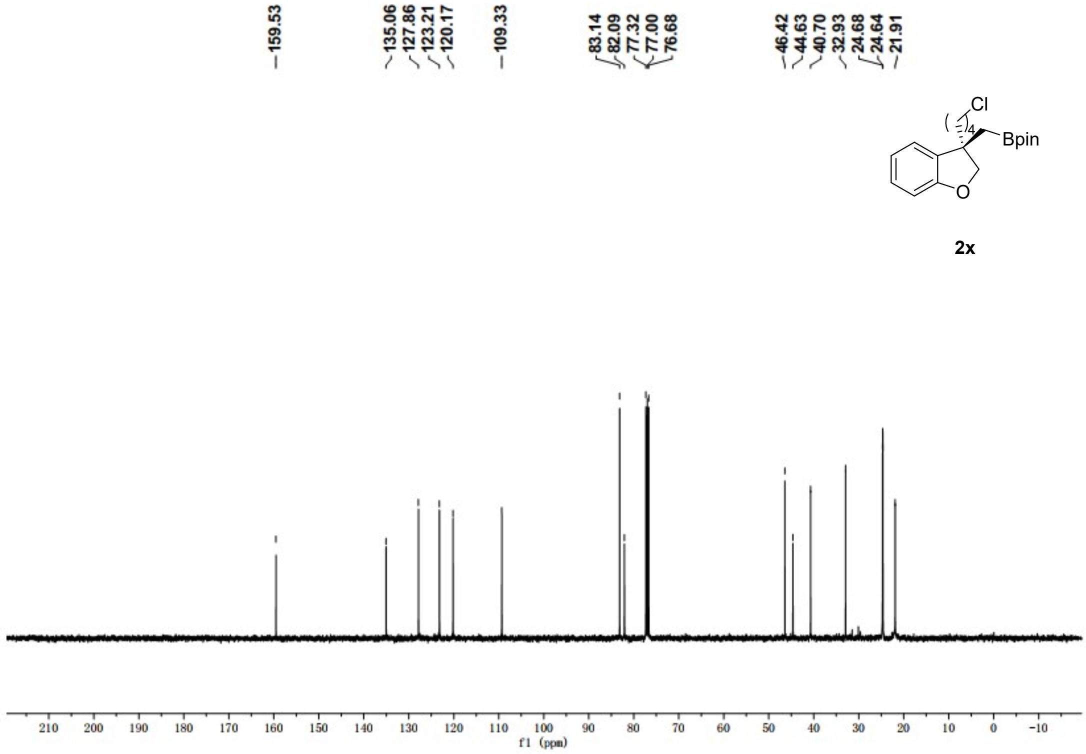
159.53
135.06 127.86 123.21 120.17
109.33
83.14 82.09 77.32 77.00 76.68
46.42 44.63 40.70 32.93 24.68 24.64 21.91
Cl
Bpin
O
f1 (ppm)

2x

**Chromatogram**

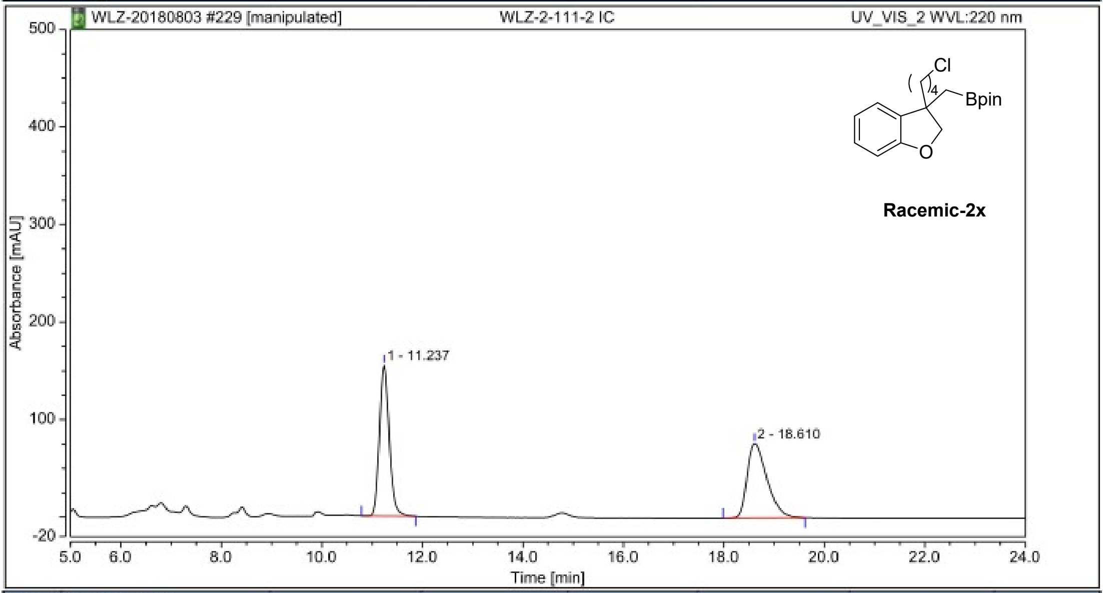


**Integration Results**

| No. | Peak Name | Retention Time min | Area mAU*min | Height mAU | Relative Area % | Relative Height % | Amount n.a. |
|---|---|---|---|---|---|---|---|
| 1 | | 11.237 | 34.953 | 154.683 | 49.90 | 66.92 | n.a. |
| 2 | | 18.610 | 35.090 | 76.477 | 50.10 | 33.08 | n.a. |
| **Total:** | | | **70.043** | **231.159** | **100.00** | **100.00** | |

**Chromatogram**

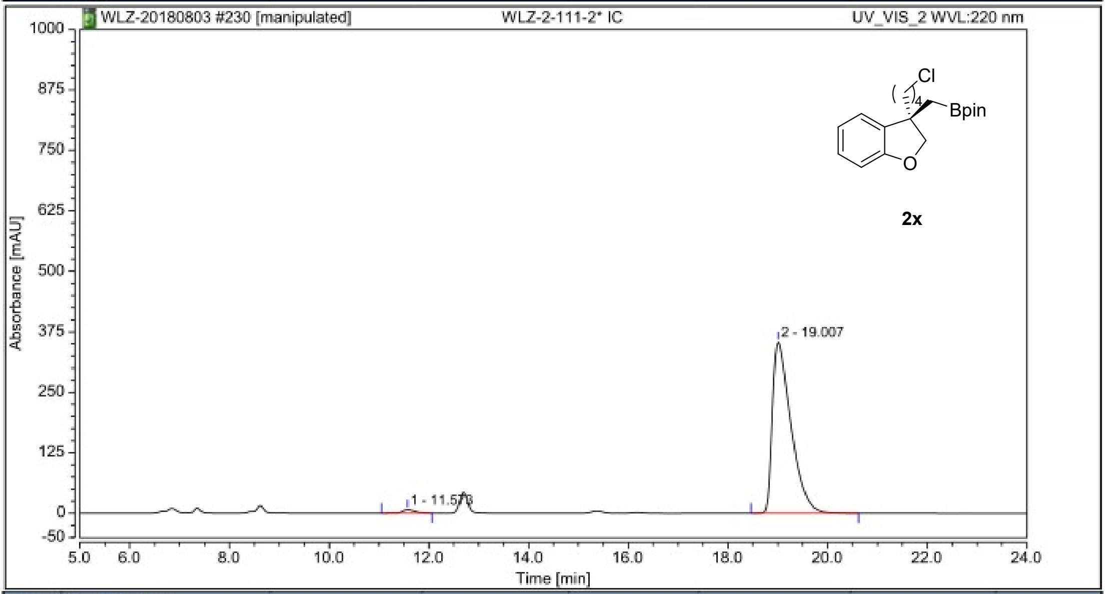


**Integration Results**

| No. | Peak Name | Retention Time min | Area mAU*min | Height mAU | Relative Area % | Relative Height % | Amount n.a. |
|---|---|---|---|---|---|---|---|
| 1 | | 11.573 | 2.207 | 7.141 | 1.42 | 1.98 | n.a. |
| 2 | | 19.007 | 153.517 | 354.423 | 98.58 | 98.02 | n.a. |
| **Total:** | | | **155.725** | **361.564** | **100.00** | **100.00** | |

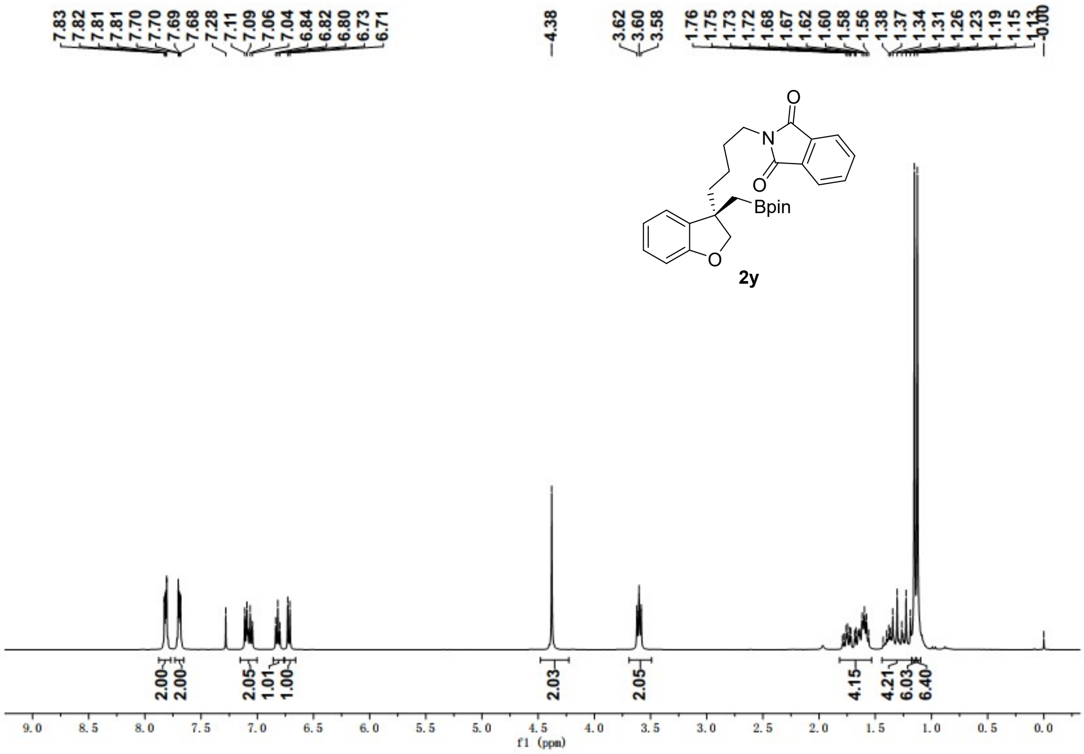
7.83 7.82 7.81 7.81 7.70 7.69 7.68 7.28 7.11 7.09 7.06 7.04 6.84 6.82 6.80 6.73 6.71
4.38
3.62 3.60 3.58
1.76 1.75 1.73 1.72 1.68 1.67 1.62 1.60 1.58 1.56 1.38 1.37 1.34 1.31 1.26 1.23 1.19 1.15 1.13 0.00
O
N
O
Bpin
O
2y
2.00 2.00 2.05 1.01 1.00 2.03 2.05 4.15 4.21 6.03 6.40
9.0 8.5 8.0 7.5 7.0 6.5 6.0 5.5 5.0 4.5 4.0 3.5 3.0 2.5 2.0 1.5 1.0 0.5 0.0
f1 (ppm)

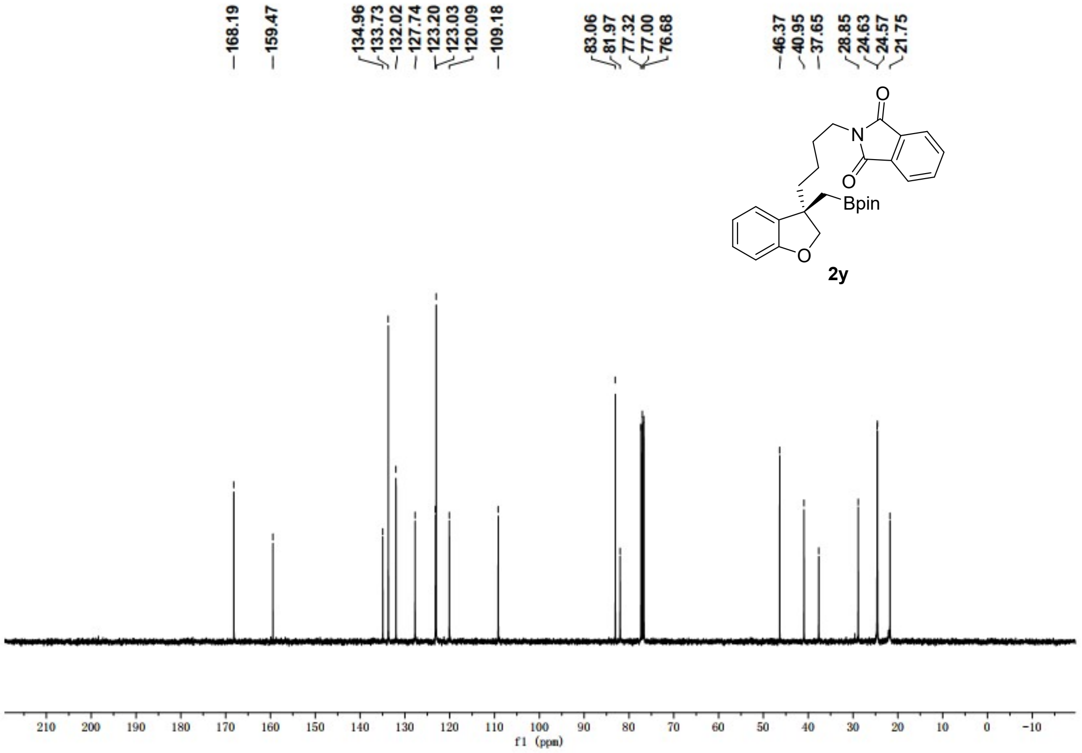
168.19
159.47
134.96 133.73 132.02 127.74 123.20 123.03 120.09 109.18
83.06 81.97 77.32 77.00 76.68
46.37 40.95 37.65 28.85 24.63 24.57 21.75
O
N
O
Bpin
O
2y
210 200 190 180 170 160 150 140 130 120 110 100 90 80 70 60 50 40 30 20 10 0 -10
f1 (ppm)

## Chromatogram

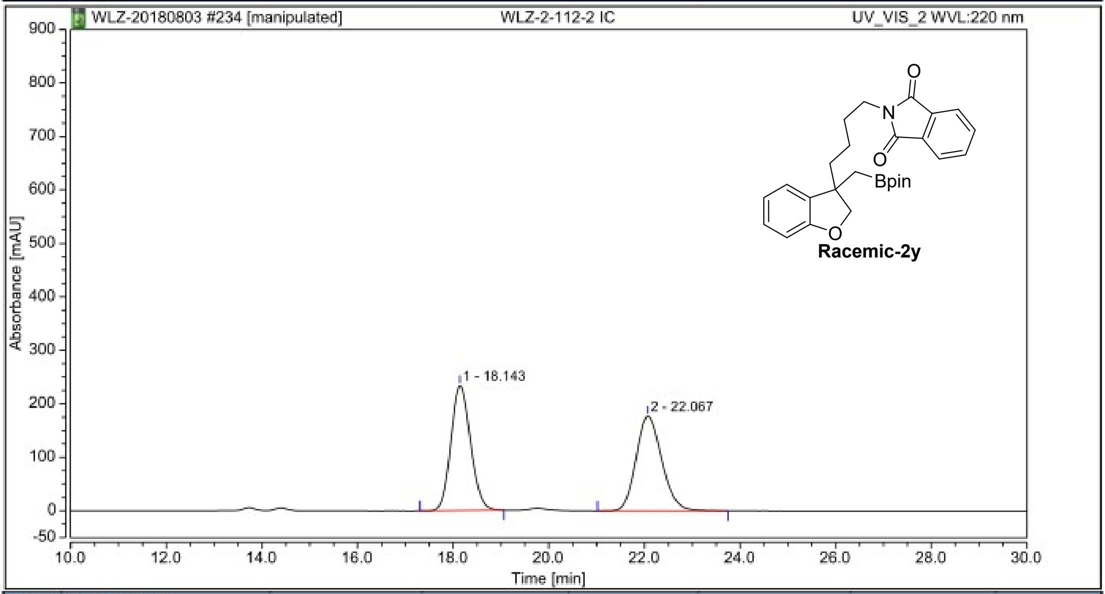


## Integration Results

| No. | Peak Name | Retention Time min | Area mAU*min | Height mAU | Relative Area % | Relative Height % | Amount n.a. |
|---|---|---|---|---|---|---|---|
| 1 | | 18.143 | 111.572 | 233.710 | 50.10 | 56.89 | n.a. |
| 2 | | 22.067 | 111.112 | 177.068 | 49.90 | 43.11 | n.a. |
| **Total:** | | | **222.684** | **410.778** | **100.00** | **100.00** | |

## Chromatogram

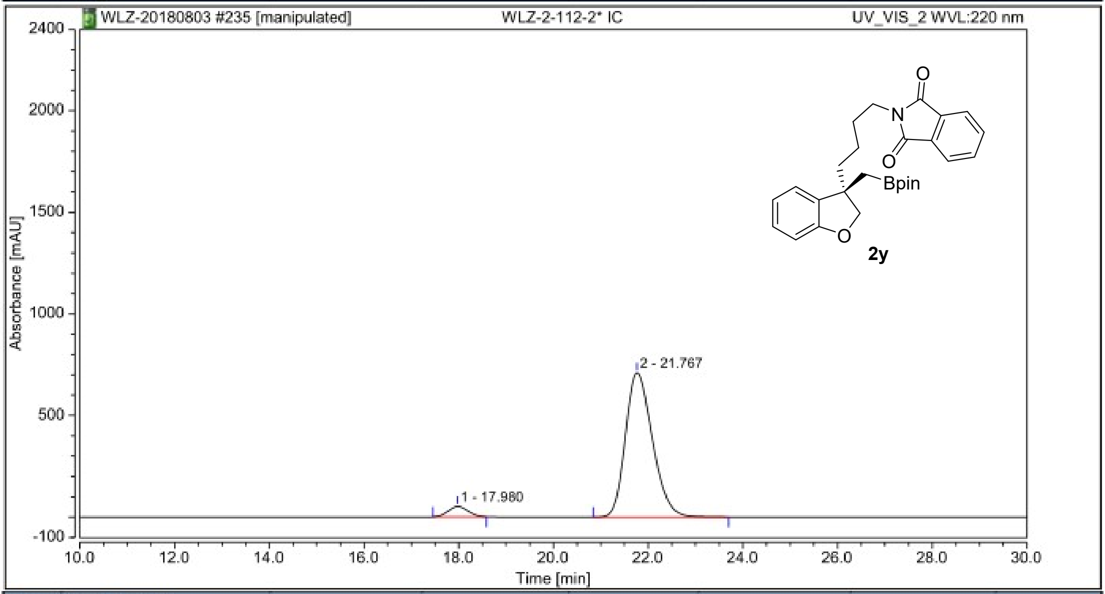


## Integration Results

| No. | Peak Name | Retention Time min | Area mAU*min | Height mAU | Relative Area % | Relative Height % | Amount n.a. |
|---|---|---|---|---|---|---|---|
| 1 | | 17.980 | 24.634 | 51.034 | 4.98 | 6.70 | n.a. |
| 2 | | 21.767 | 470.062 | 711.156 | 95.02 | 93.30 | n.a. |
| **Total:** | | | **494.696** | **762.190** | **100.00** | **100.00** | |

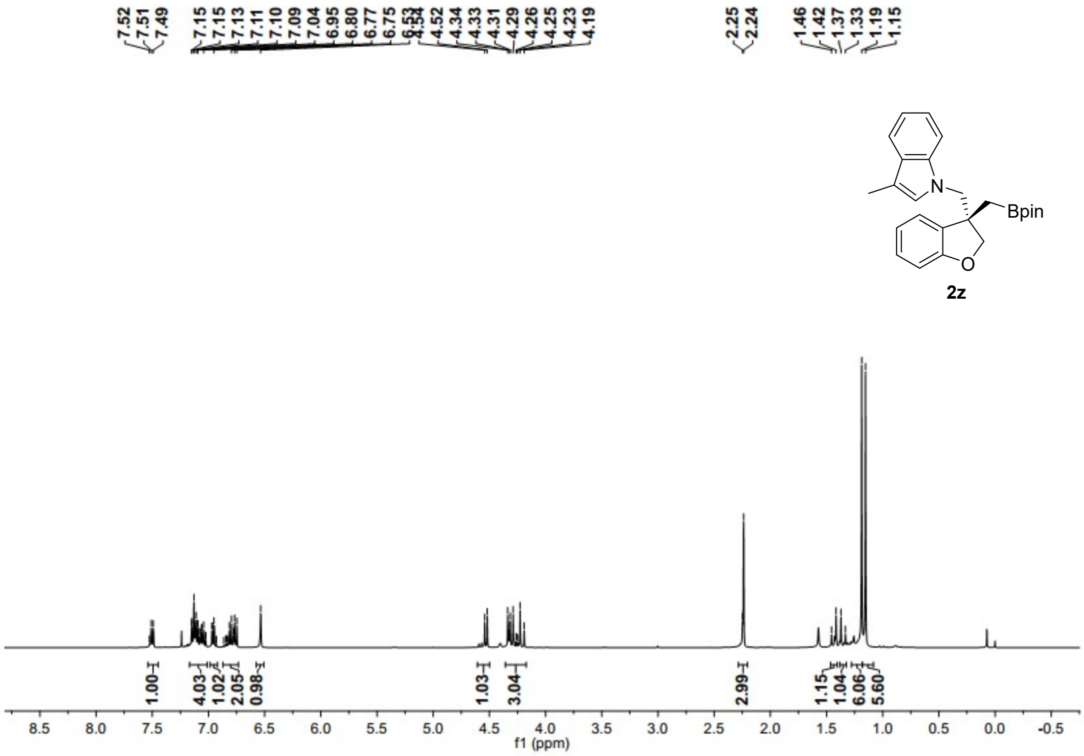
7.52 7.51 7.49 7.15 7.15 7.13 7.11 7.10 7.09 7.04 6.95 6.80 6.77 6.75 6.53 4.54 4.52 4.34 4.33 4.31 4.29 4.26 4.25 4.23 4.19 2.25 2.24 1.46 1.42 1.37 1.33 1.19 1.15
N
Bpin
O
1.00 4.03 1.02 2.05 0.98 1.03 3.04 2.99 1.15 1.04 6.06 5.60
8.5 8.0 7.5 7.0 6.5 6.0 5.5 5.0 4.5 4.0 3.5 3.0 2.5 2.0 1.5 1.0 0.5 0.0 -0.5
f1 (ppm)

2z

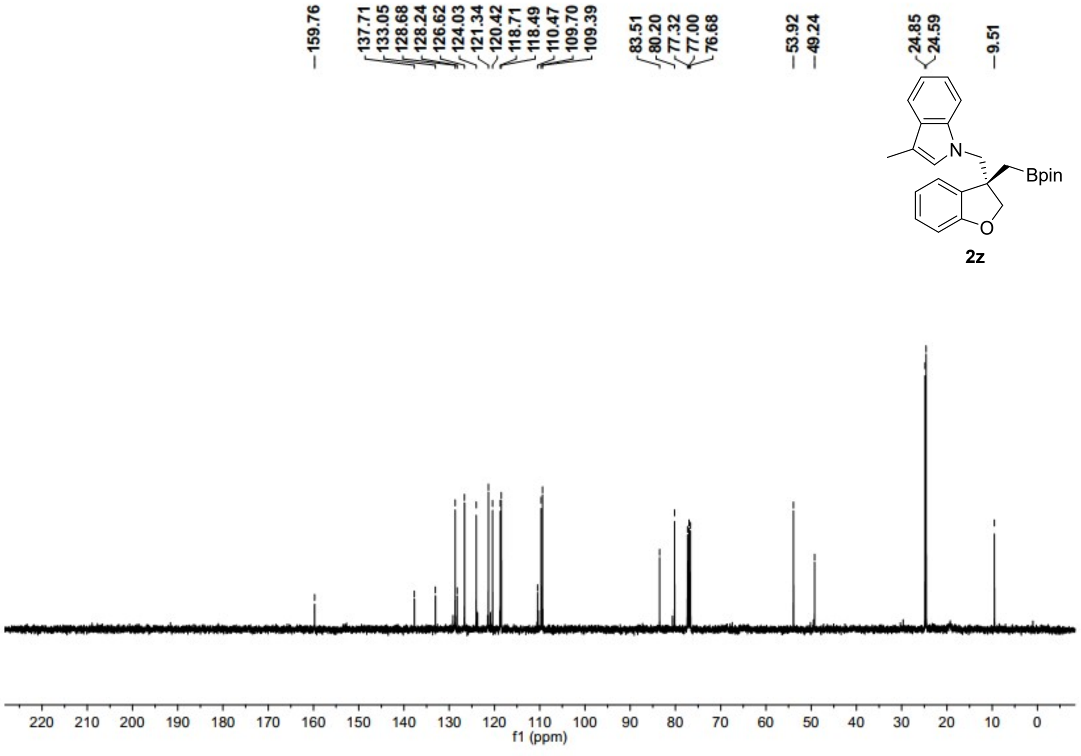
159.76 137.71 133.05 128.68 128.24 126.62 124.03 121.34 120.42 118.71 118.49 110.47 109.70 109.39 83.51 80.20 77.32 77.00 76.68 53.92 49.24 24.85 24.59 9.51
N
Bpin
O
220 210 200 190 180 170 160 150 140 130 120 110 100 90 80 70 60 50 40 30 20 10 0
f1 (ppm)

2z

## Chromatogram

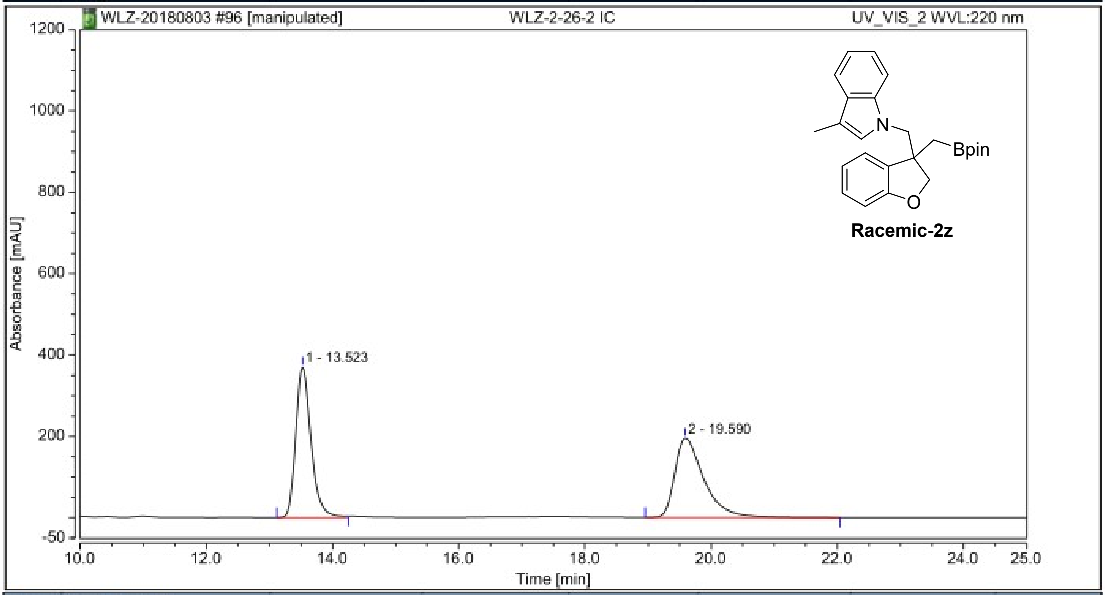


## Integration Results

| No. | Peak Name | Retention Time min | Area mAU*min | Height mAU | Relative Area % | Relative Height % | Amount n.a. |
|---|---|---|---|---|---|---|---|
| 1 | | 13.523 | 104.281 | 370.472 | 49.93 | 65.53 | n.a. |
| 2 | | 19.590 | 104.587 | 194.884 | 50.07 | 34.47 | n.a. |
| **Total:** | | | **208.869** | **565.356** | **100.00** | **100.00** | |

## Chromatogram

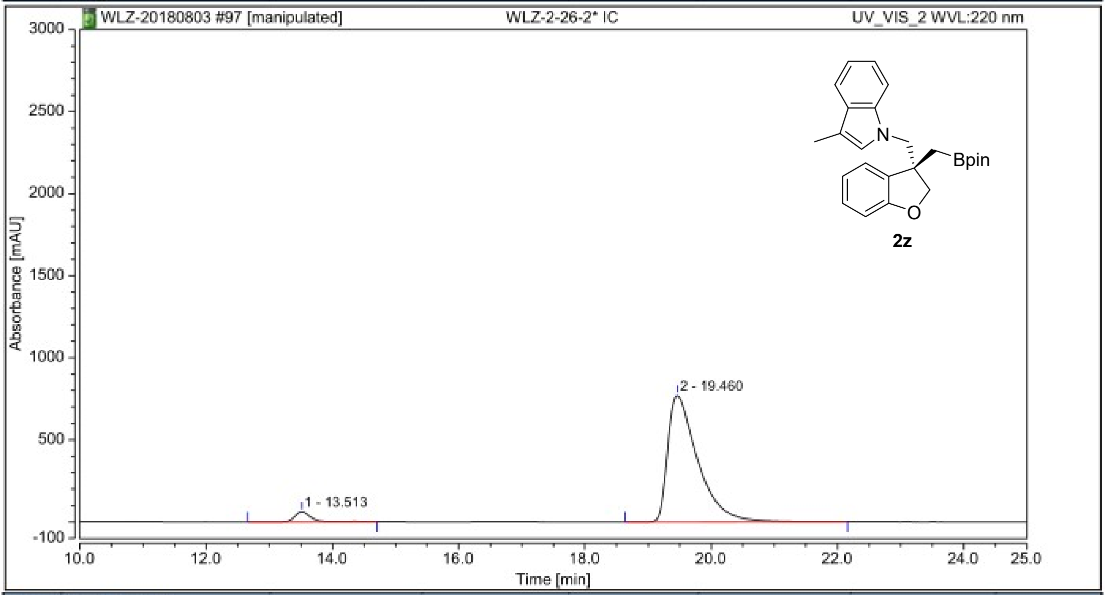


## Integration Results

| No. | Peak Name | Retention Time min | Area mAU*min | Height mAU | Relative Area % | Relative Height % | Amount n.a. |
|---|---|---|---|---|---|---|---|
| 1 | | 13.513 | 18.398 | 61.422 | 4.16 | 7.38 | n.a. |
| 2 | | 19.460 | 424.263 | 770.632 | 95.84 | 92.62 | n.a. |
| **Total:** | | | **442.660** | **832.054** | **100.00** | **100.00** | |

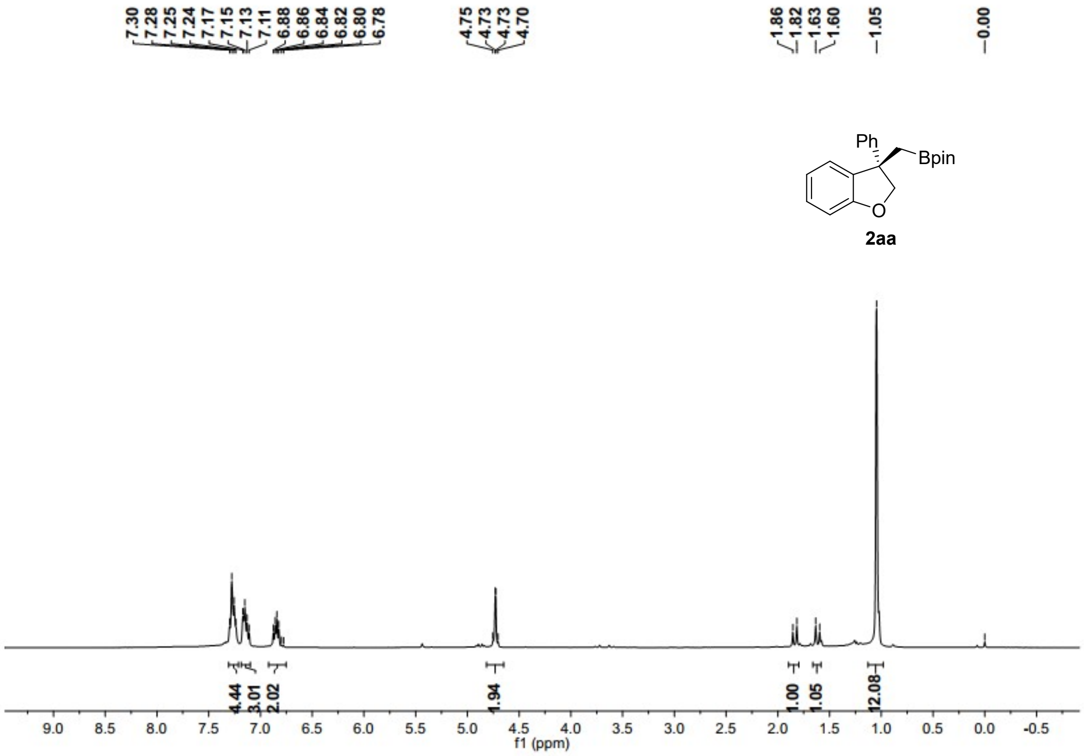
7.30
7.28
7.25
7.24
7.17
7.15
7.13
7.11
6.88
6.86
6.84
6.82
6.80
6.78
4.75
4.73
4.73
4.70
1.86
1.82
1.63
1.60
1.05
0.00
Ph
Bpin
O
2aa
4.44
3.01
2.02
1.94
1.00
1.05
12.08
9.0 8.5 8.0 7.5 7.0 6.5 6.0 5.5 5.0 4.5 4.0 3.5 3.0 2.5 2.0 1.5 1.0 0.5 0.0 -0.5
f1 (ppm)

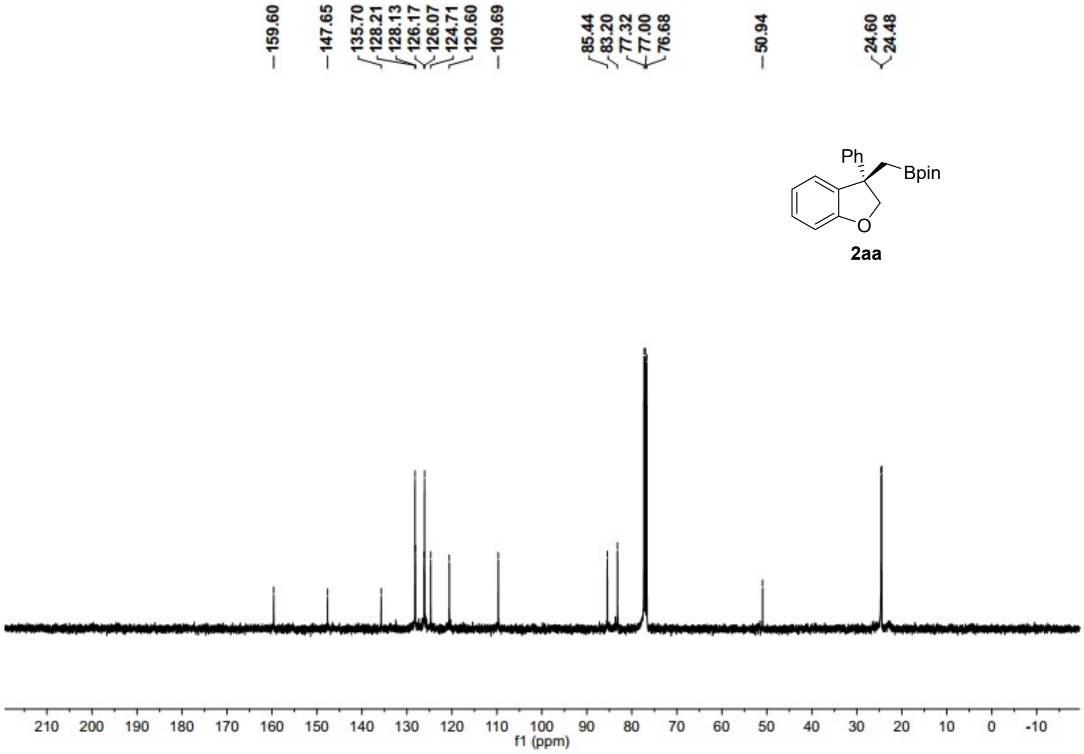
159.60
147.65
135.70
128.21
128.13
126.17
126.07
124.71
120.60
109.69
85.44
83.20
77.32
77.00
76.68
50.94
24.60
24.48
Ph
Bpin
O
2aa
210 200 190 180 170 160 150 140 130 120 110 100 90 80 70 60 50 40 30 20 10 0 -10
f1 (ppm)

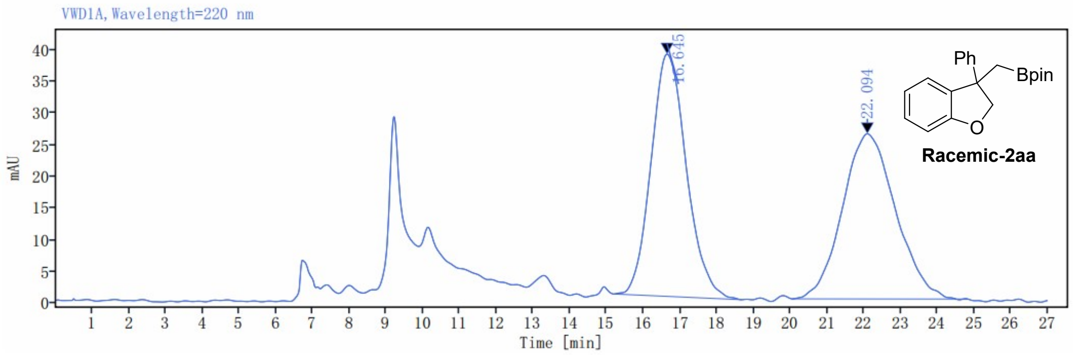


VWD1A, Wavelength=220 nm

| Ret. Time [min] | Area | Height | Height% | Area% |
|---|---|---|---|---|
| 16.645 | 2557.69 | 38.15 | 59.46 | 49.51 |
| 22.094 | 2608.13 | 26.01 | 40.54 | 50.49 |
| Total. | 5165.81 | 64.16 | 100.00 | 100.00 |

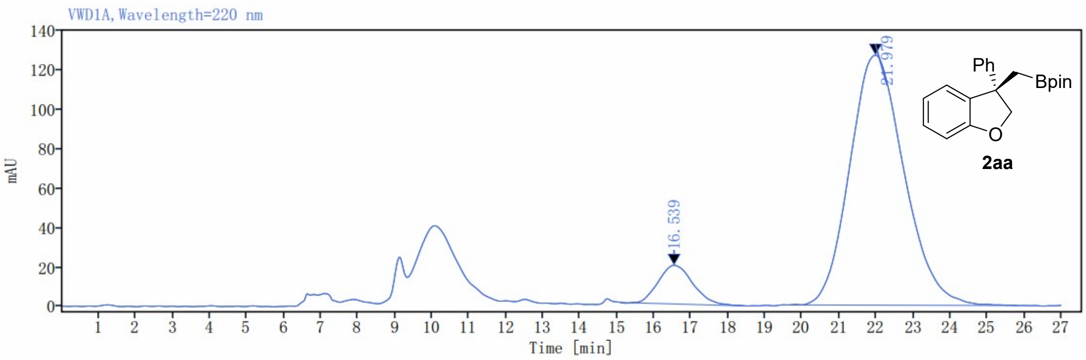


VWD1A, Wavelength=220 nm

| Ret. Time [min] | Area | Height | Height% | Area% |
|---|---|---|---|---|
| 16.539 | 1271.31 | 19.56 | 13.39 | 8.97 |
| 21.979 | 12897.34 | 126.53 | 86.61 | 91.03 |
| Total. | 14168.65 | 146.09 | 100.00 | 100.00 |

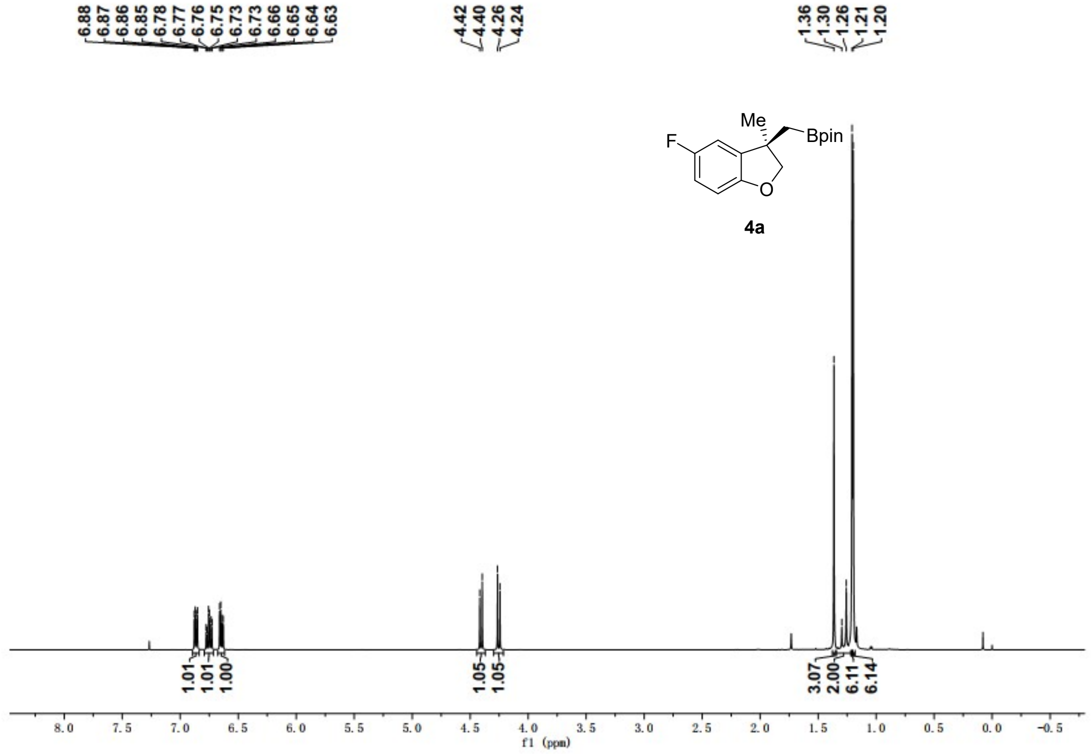
6.88 6.87 6.86 6.85 6.78 6.77 6.76 6.75 6.73 6.73 6.66 6.65 6.64 6.63
4.42 4.40 4.26 4.24
1.36 1.30 1.26 1.21 1.20
Me
Bpin
F
O
4a
1.01 1.01 1.00
1.05 1.05
3.07 2.00 6.11 6.14
8.0 7.5 7.0 6.5 6.0 5.5 5.0 4.5 4.0 3.5 3.0 2.5 2.0 1.5 1.0 0.5 0.0 -0.5
f1 (ppm)

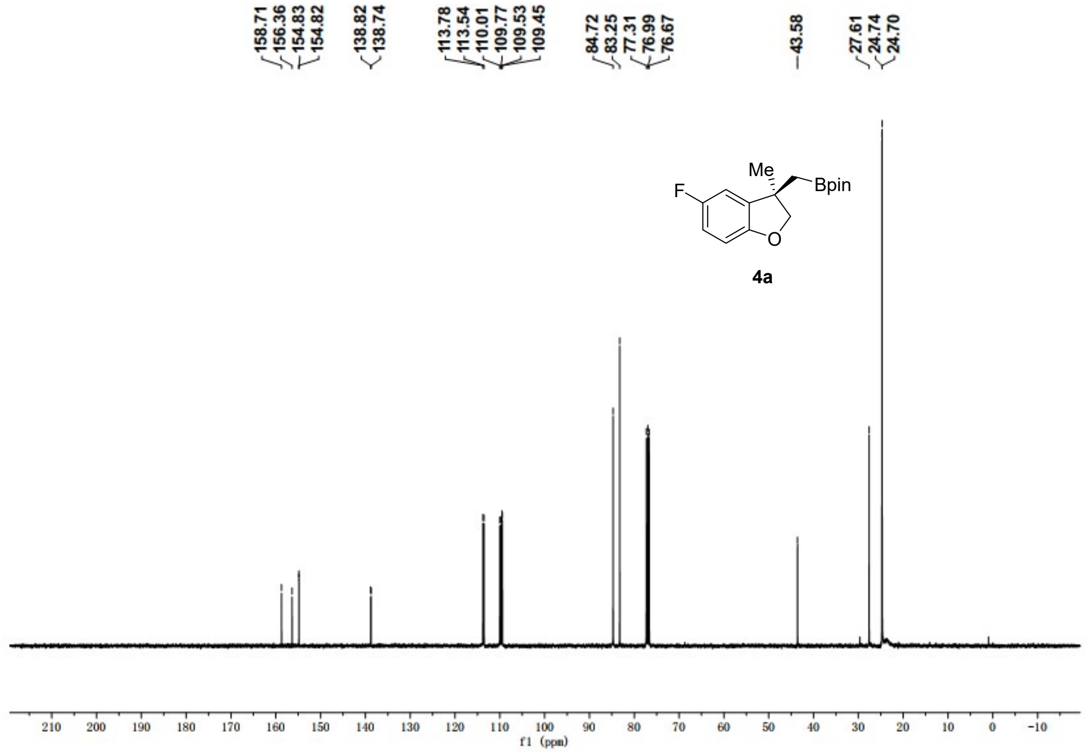
158.71 156.36 154.83 154.82
138.82 138.74
113.78 113.54 110.01 109.77 109.53 109.45
84.72 83.25 77.31 76.99 76.67
43.58
27.61 24.74 24.70
Me
Bpin
F
O
4a
210 200 190 180 170 160 150 140 130 120 110 100 90 80 70 60 50 40 30 20 10 0 -10
f1 (ppm)

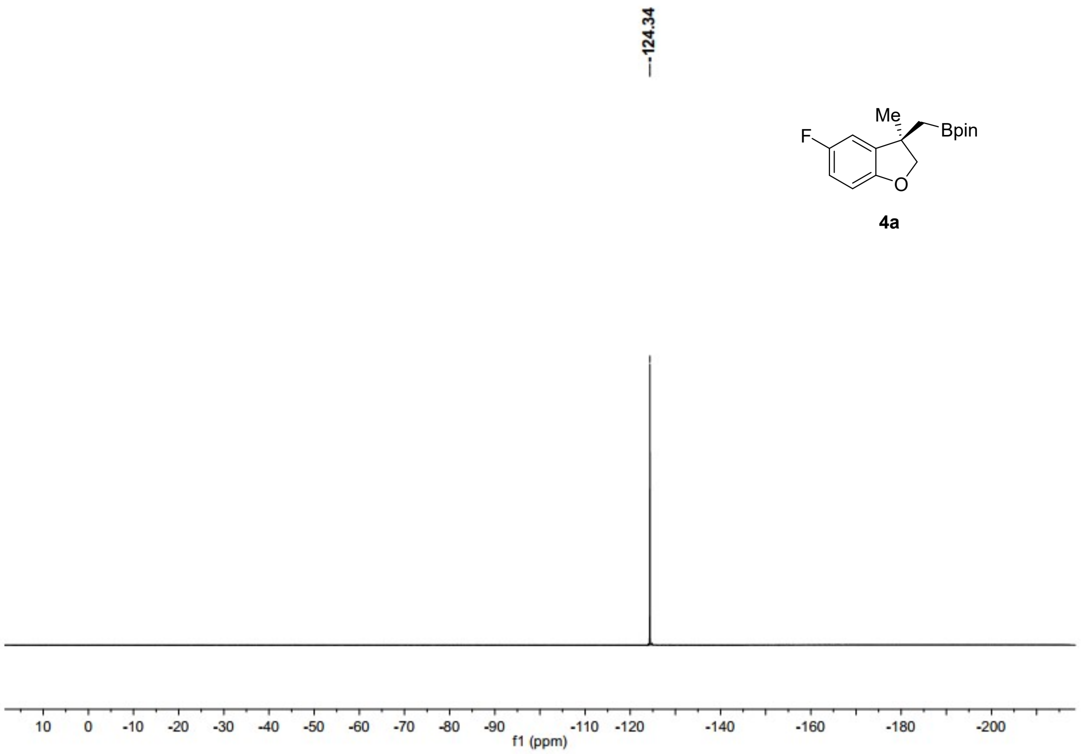


**Chromatogram**

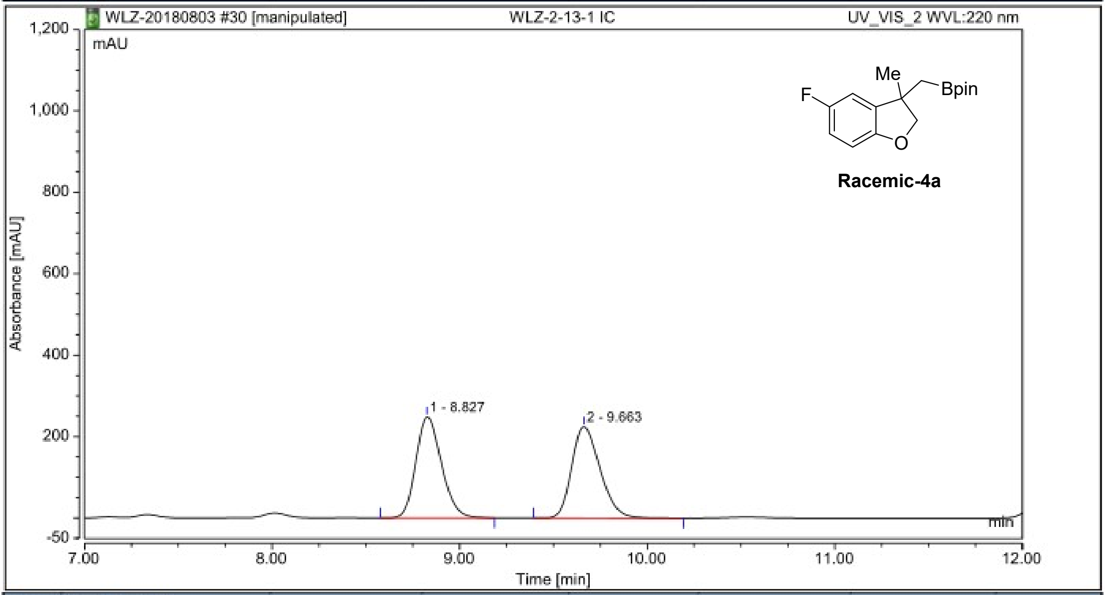


**Integration Results**

| No. | Peak Name | Retention Time min | Area mAU*min | Height mAU | Relative Area % | Relative Height % | Amount n.a. |
|---|---|---|---|---|---|---|---|
| 1 | | 8.827 | 39.297 | 248.570 | 49.99 | 52.55 | n.a. |
| 2 | | 9.663 | 39.305 | 224.470 | 50.01 | 47.45 | n.a. |
| **Total:** | | | **78.602** | **473.039** | **100.00** | **100.00** | |

## Chromatogram

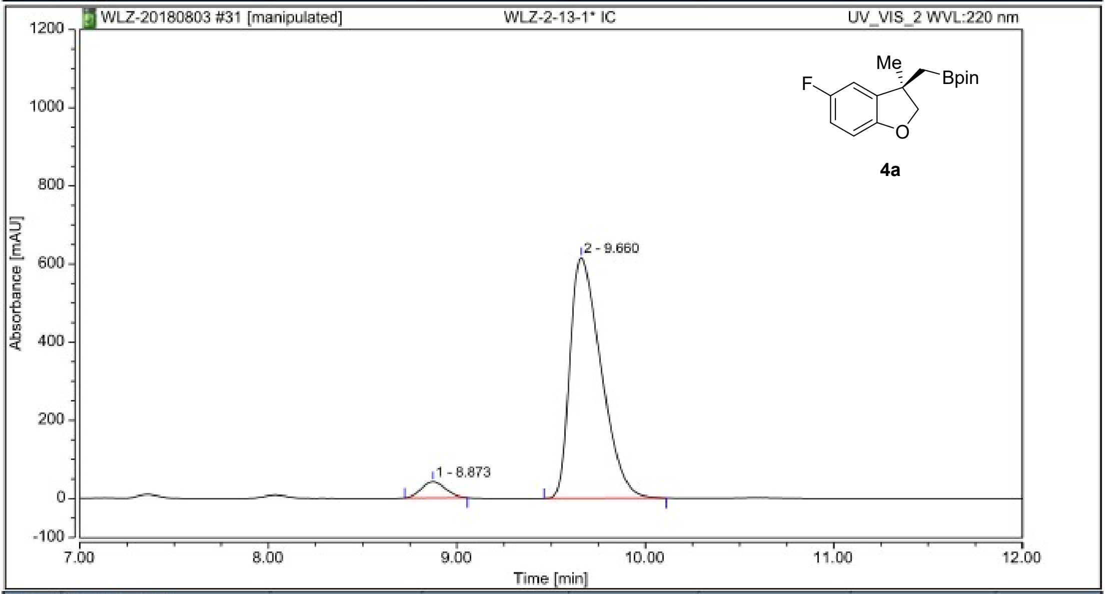


## Integration Results

| No. | Peak Name | Retention Time min | Area mAU*min | Height mAU | Relative Area % | Relative Height % | Amount n.a. |
|---|---|---|---|---|---|---|---|
| 1 | | 8.873 | 6.121 | 41.304 | 4.94 | 6.29 | n.a. |
| 2 | | 9.660 | 117.877 | 615.363 | 95.06 | 93.71 | n.a. |
| **Total:** | | | **123.998** | **656.667** | **100.00** | **100.00** | |

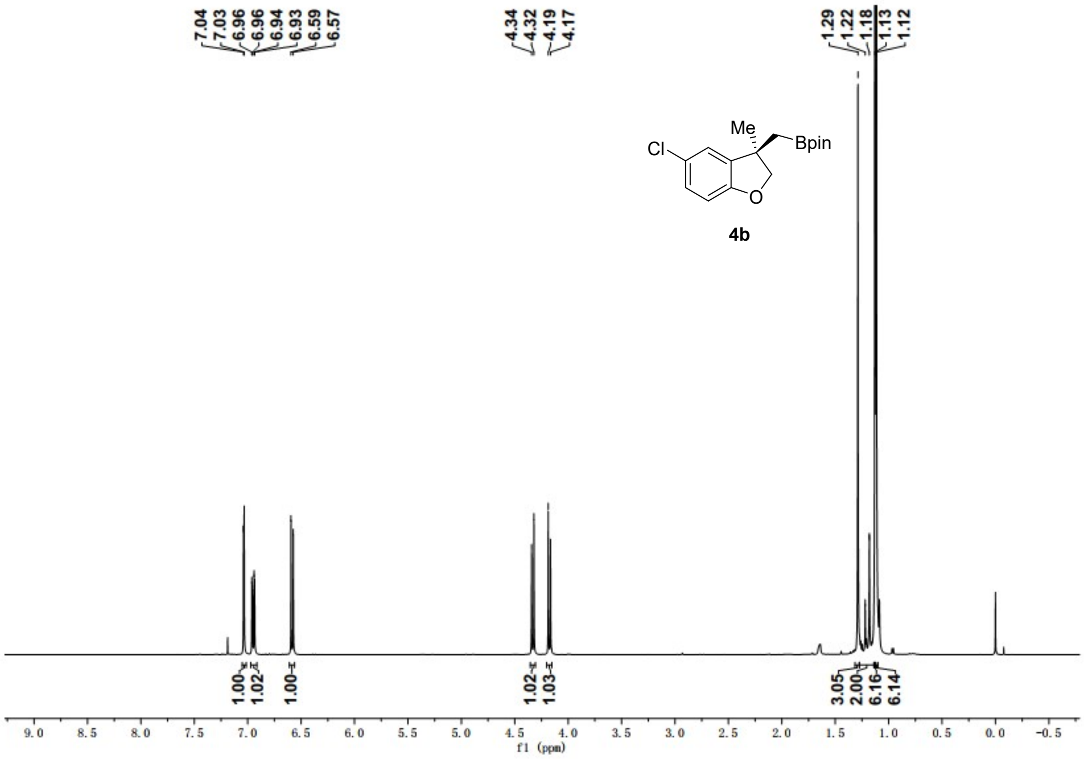

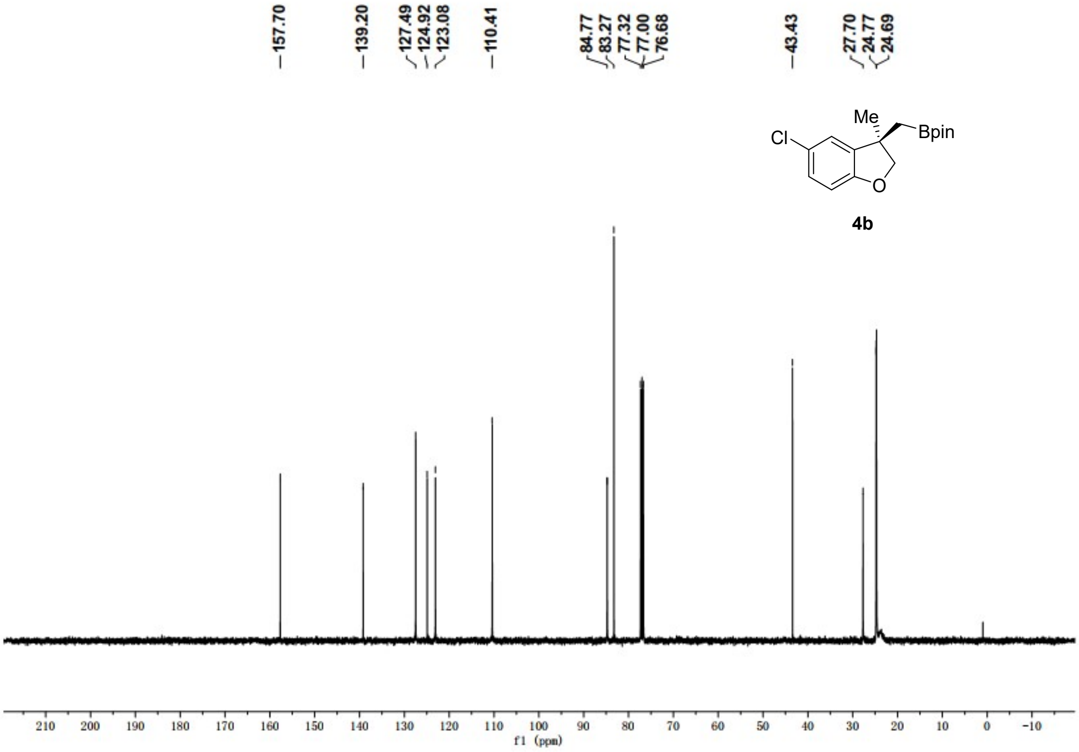


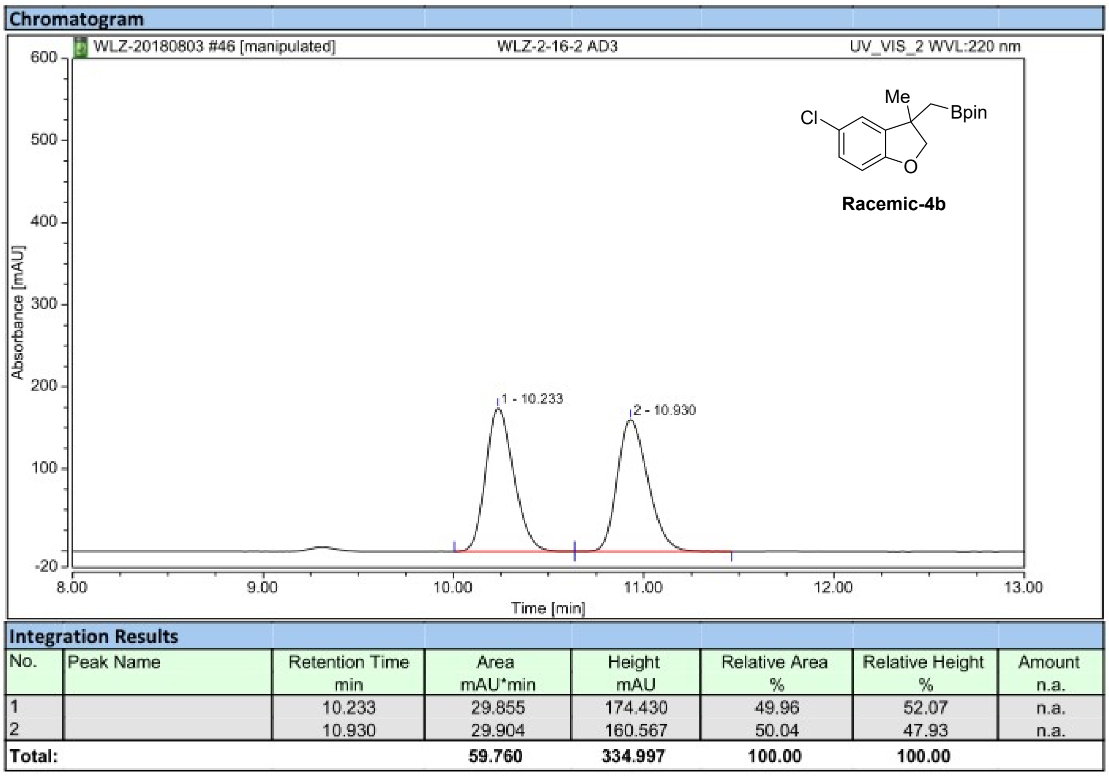


**Integration Results**

| No. | Peak Name | Retention Time min | Area mAU*min | Height mAU | Relative Area % | Relative Height % | Amount n.a. |
|---|---|---|---|---|---|---|---|
| 1 | | 10.233 | 29.855 | 174.430 | 49.96 | 52.07 | n.a. |
| 2 | | 10.930 | 29.904 | 160.567 | 50.04 | 47.93 | n.a. |
| **Total:** | | | **59.760** | **334.997** | **100.00** | **100.00** | |

**Chromatogram**

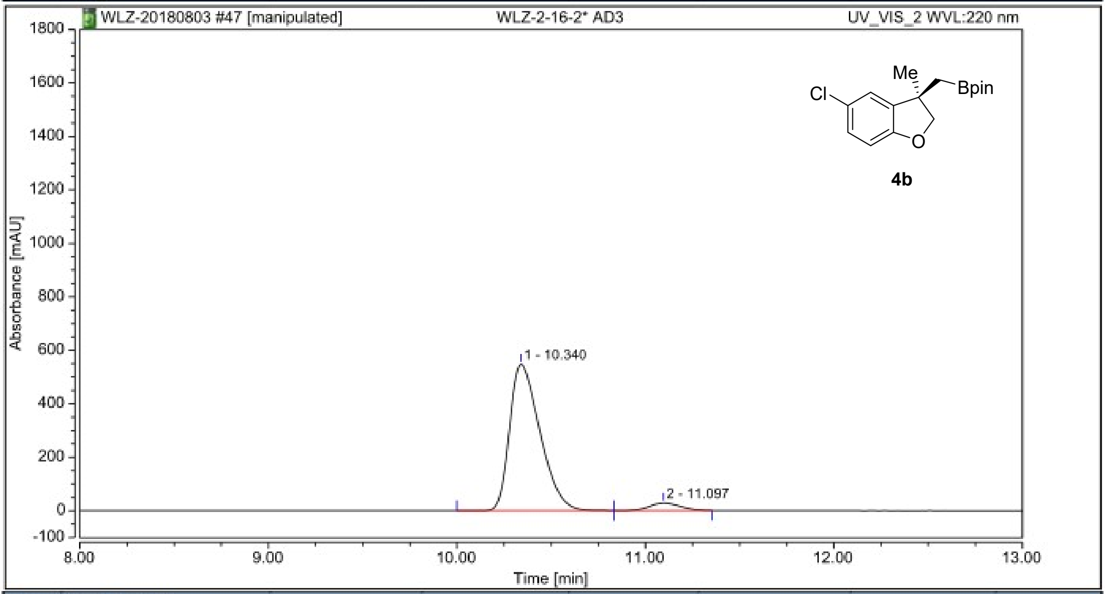


**Integration Results**

| No. | Peak Name | Retention Time min | Area mAU*min | Height mAU | Relative Area % | Relative Height % | Amount n.a. |
|---|---|---|---|---|---|---|---|
| 1 | | 10.340 | 102.165 | 547.571 | 95.07 | 95.14 | n.a. |
| 2 | | 11.097 | 5.293 | 27.999 | 4.93 | 4.86 | n.a. |
| **Total:** | | | **107.458** | **575.570** | **100.00** | **100.00** | |

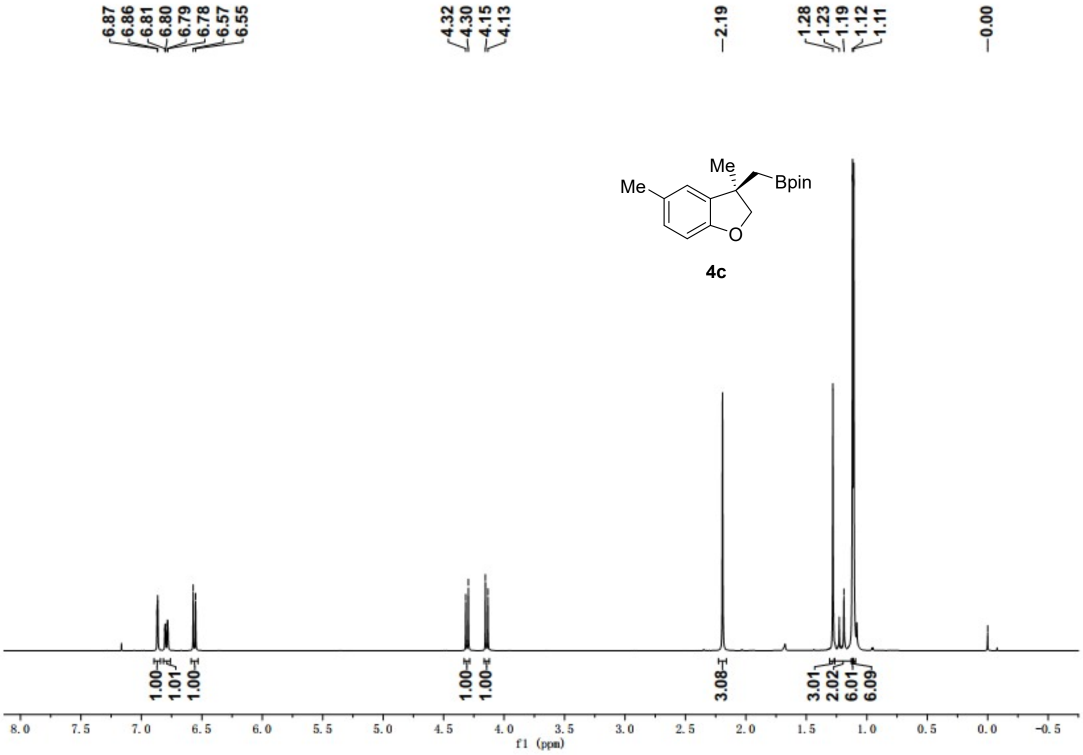

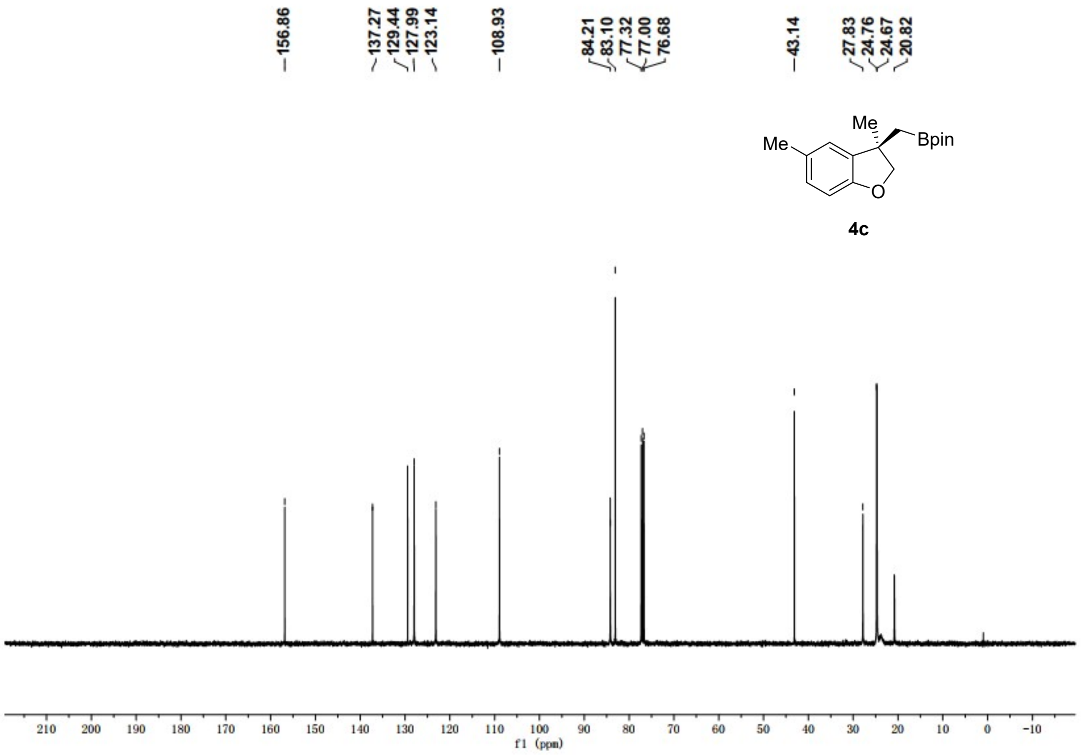


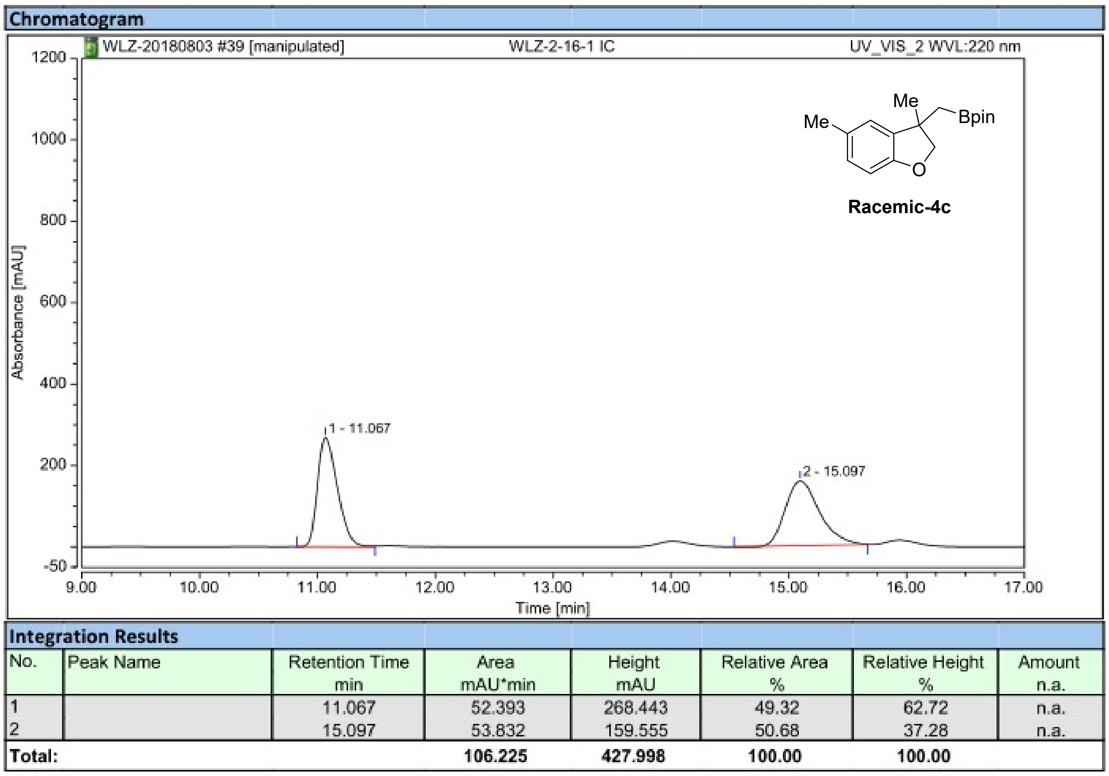


**Integration Results**

| No. | Peak Name | Retention Time min | Area mAU*min | Height mAU | Relative Area % | Relative Height % | Amount n.a. |
|---|---|---|---|---|---|---|---|
| 1 | | 11.067 | 52.393 | 268.443 | 49.32 | 62.72 | n.a. |
| 2 | | 15.097 | 53.832 | 159.555 | 50.68 | 37.28 | n.a. |
| **Total:** | | | **106.225** | **427.998** | **100.00** | **100.00** | |

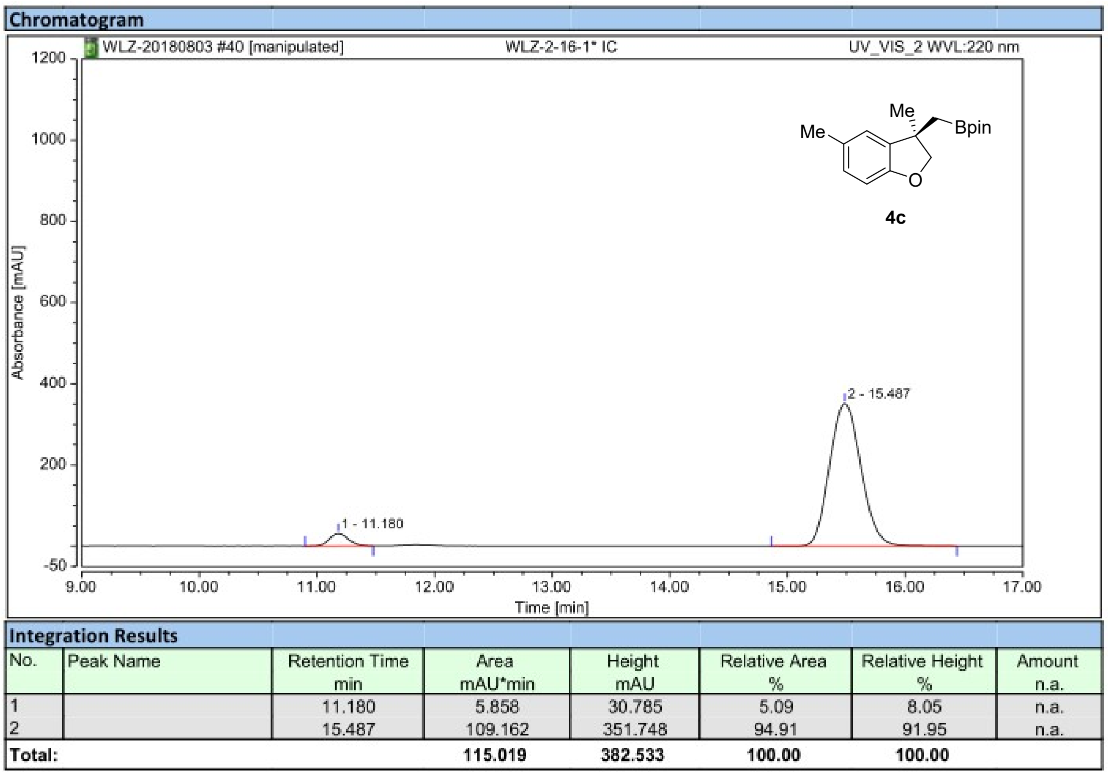


**Integration Results**

| No. | Peak Name | Retention Time min | Area mAU*min | Height mAU | Relative Area % | Relative Height % | Amount n.a. |
|---|---|---|---|---|---|---|---|
| 1 | | 11.180 | 5.858 | 30.785 | 5.09 | 8.05 | n.a. |
| 2 | | 15.487 | 109.162 | 351.748 | 94.91 | 91.95 | n.a. |
| **Total:** | | | **115.019** | **382.533** | **100.00** | **100.00** | |

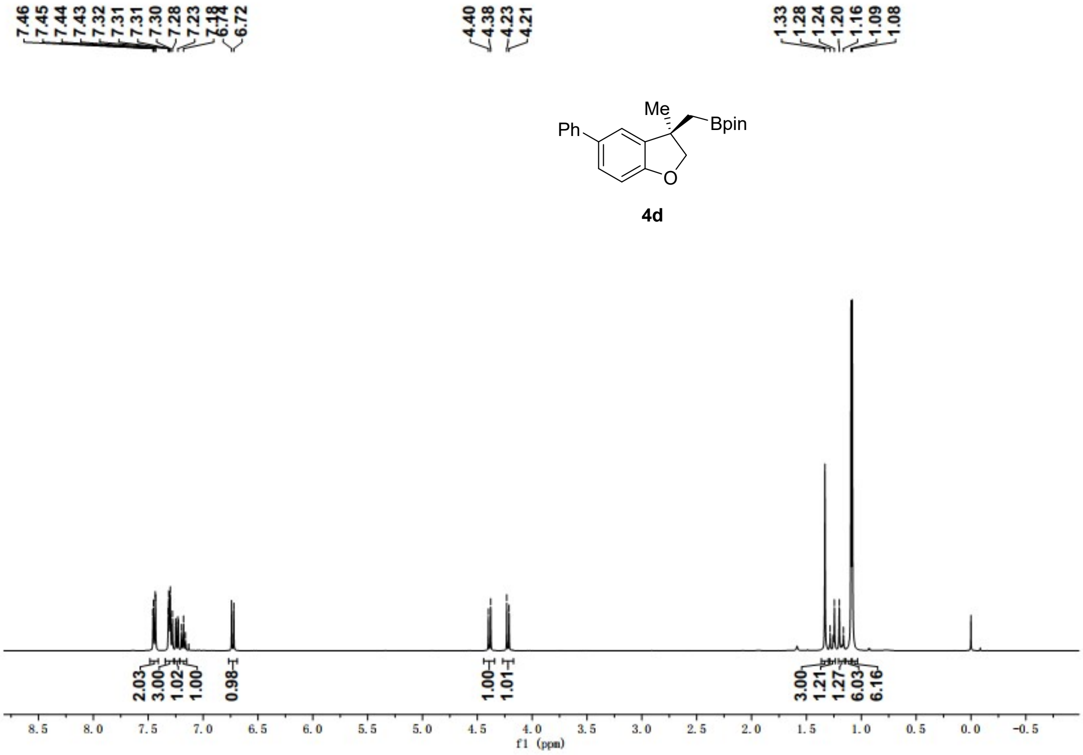

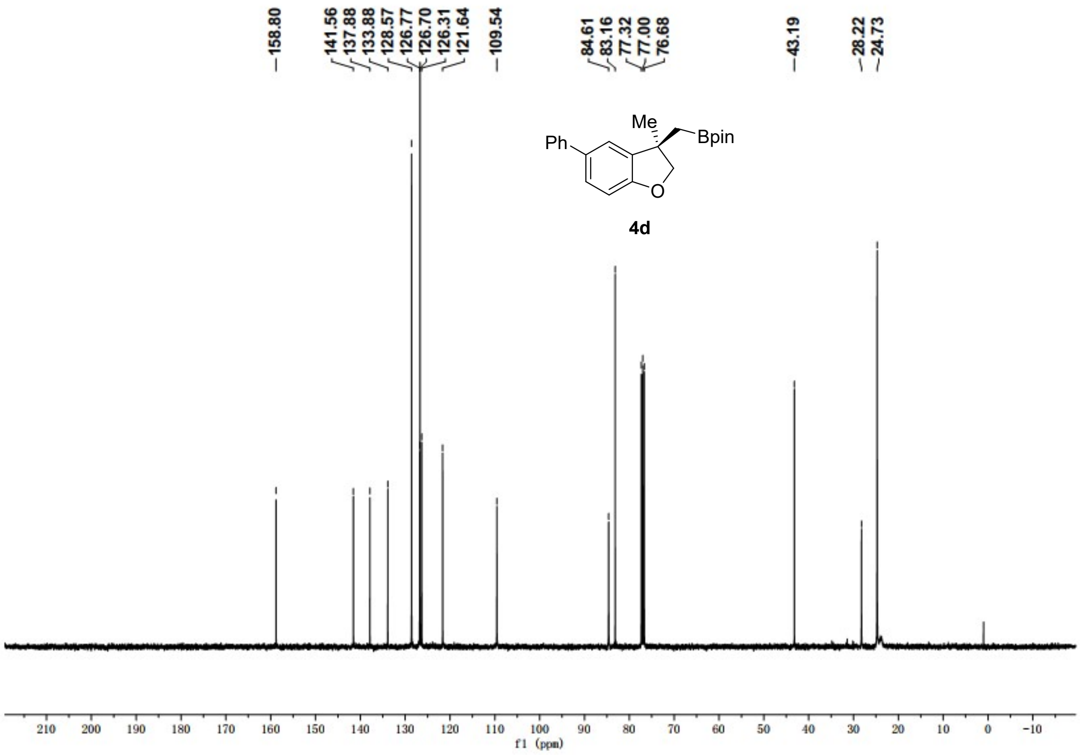


## Chromatogram

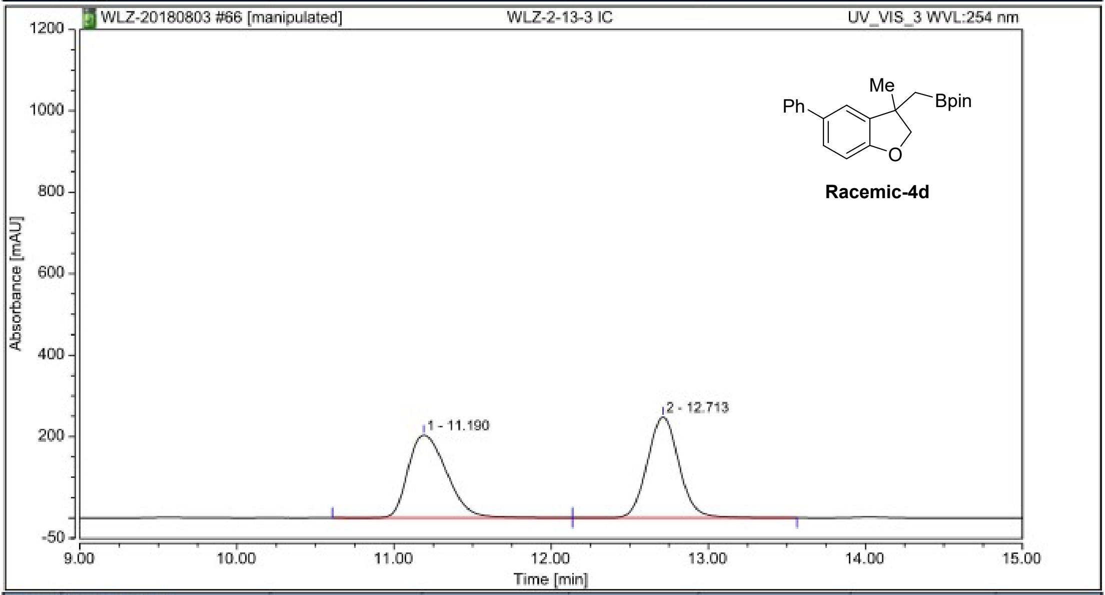


## Integration Results

| No. | Peak Name | Retention Time min | Area mAU*min | Height mAU | Relative Area % | Relative Height % | Amount n.a. |
|---|---|---|---|---|---|---|---|
| 1 | | 11.190 | 57.844 | 203.283 | 49.97 | 45.09 | n.a. |
| 2 | | 12.713 | 57.908 | 247.557 | 50.03 | 54.91 | n.a. |
| **Total:** | | | **115.752** | **450.840** | **100.00** | **100.00** | |

## Chromatogram

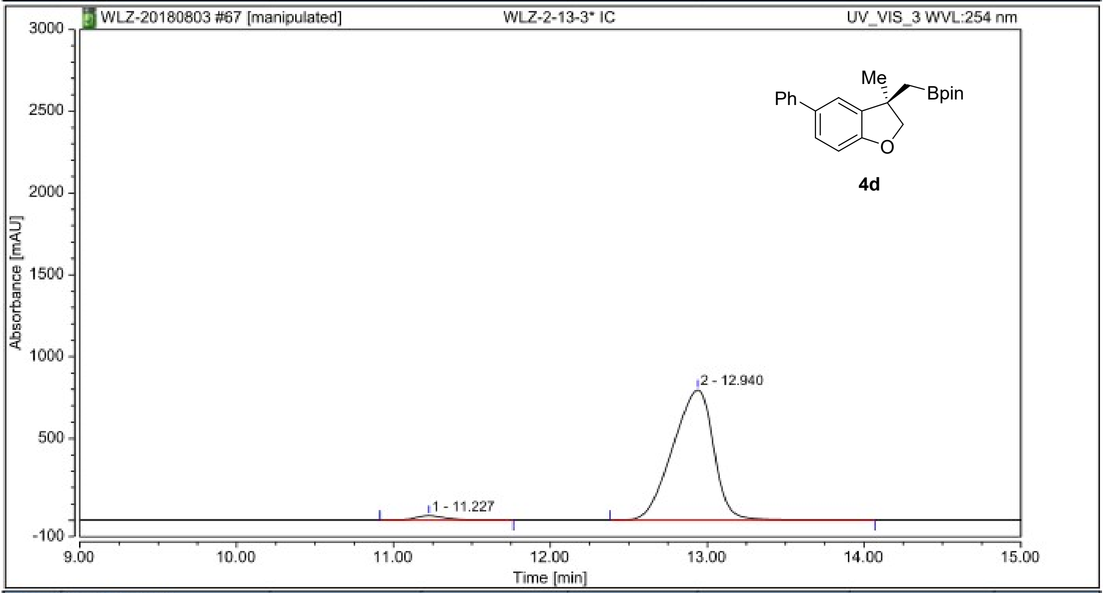


## Integration Results

| No. | Peak Name | Retention Time min | Area mAU*min | Height mAU | Relative Area % | Relative Height % | Amount n.a. |
|---|---|---|---|---|---|---|---|
| 1 | | 11.227 | 6.188 | 27.572 | 2.49 | 3.35 | n.a. |
| 2 | | 12.940 | 242.190 | 796.496 | 97.51 | 96.65 | n.a. |
| **Total:** | | | **248.378** | **824.068** | **100.00** | **100.00** | |

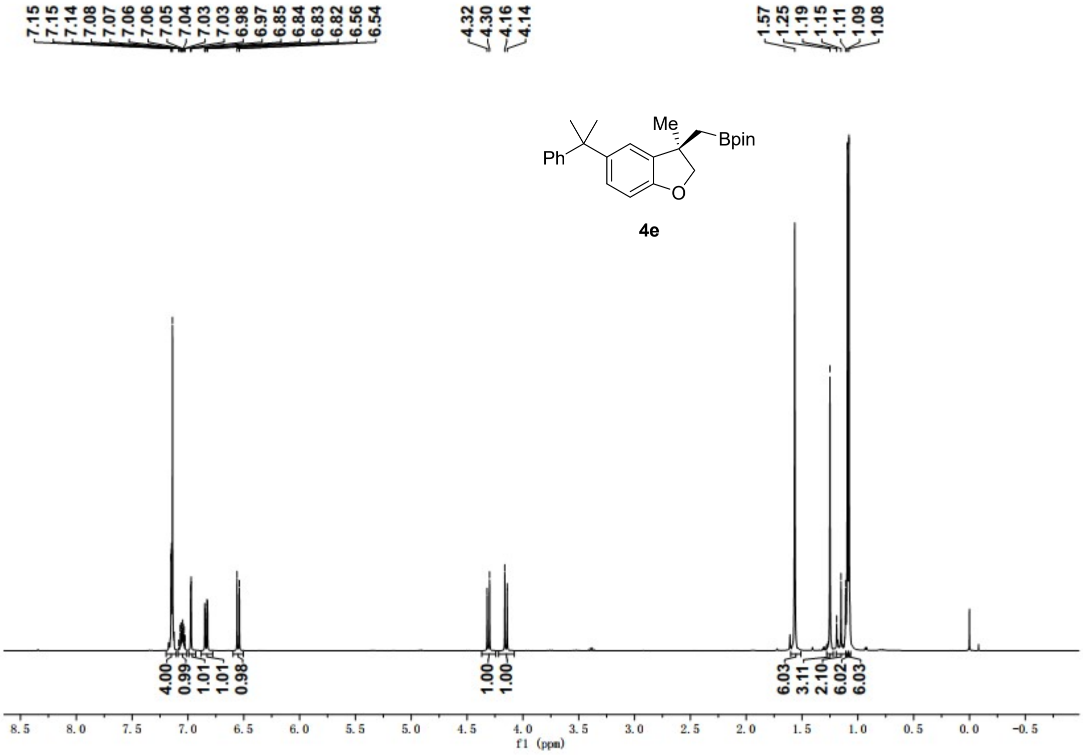

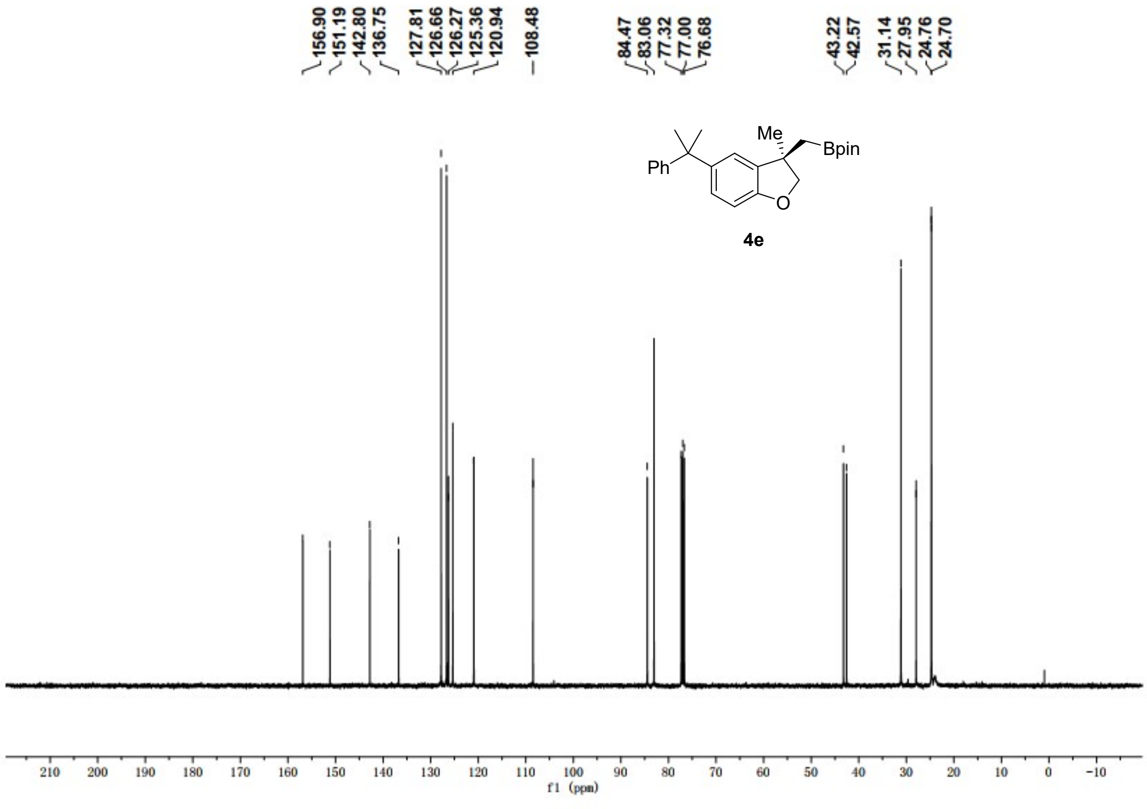


## <Chromatogram>

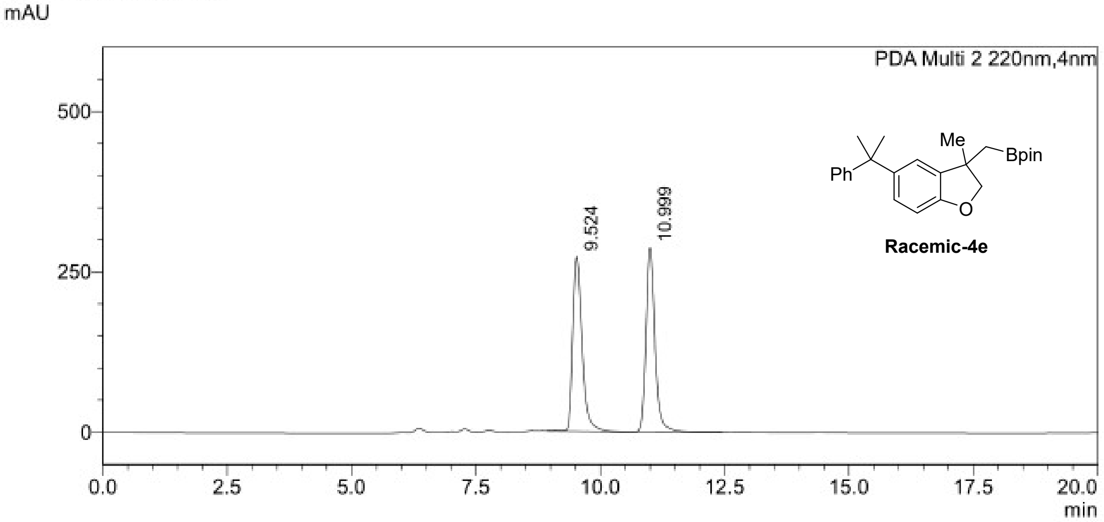


## <Peak Table>

PDA Ch2 220nm

| Peak# | Ret. Time | Area | Area% | Height | Height% |
|---|---|---|---|---|---|
| 1 | 9.524 | 3591441 | 49.845 | 272658 | 48.687 |
| 2 | 10.999 | 3613770 | 50.155 | 287364 | 51.313 |
| Total | | 7205211 | 100.000 | 560023 | 100.000 |

## <Chromatogram>

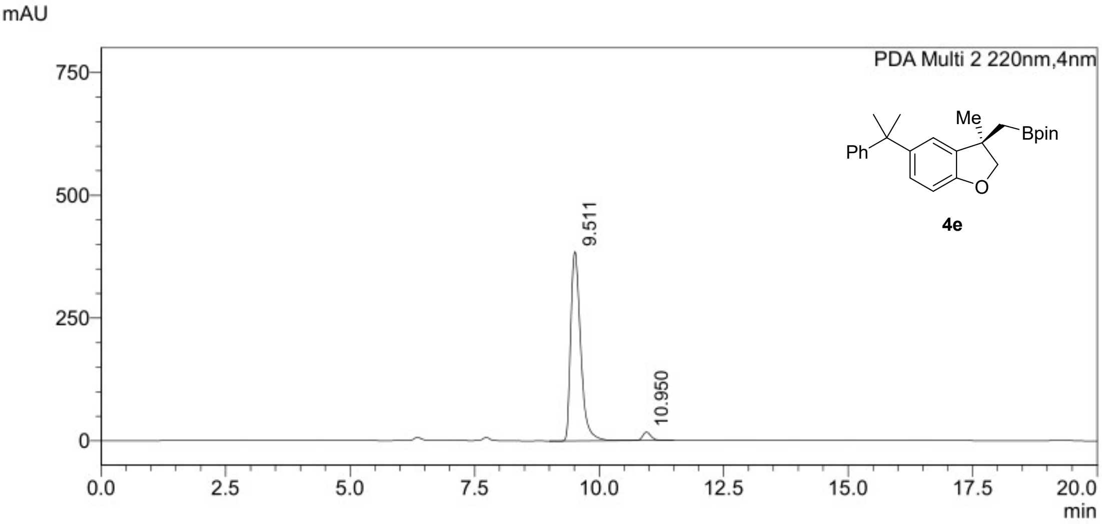


## <Peak Table>

PDA Ch2 220nm

| Peak# | Ret. Time | Area | Area% | Height | Height% |
|---|---|---|---|---|---|
| 1 | 9.511 | 5360592 | 96.165 | 385696 | 95.688 |
| 2 | 10.950 | 213761 | 3.835 | 17382 | 4.312 |
| Total | | 5574353 | 100.000 | 403078 | 100.000 |

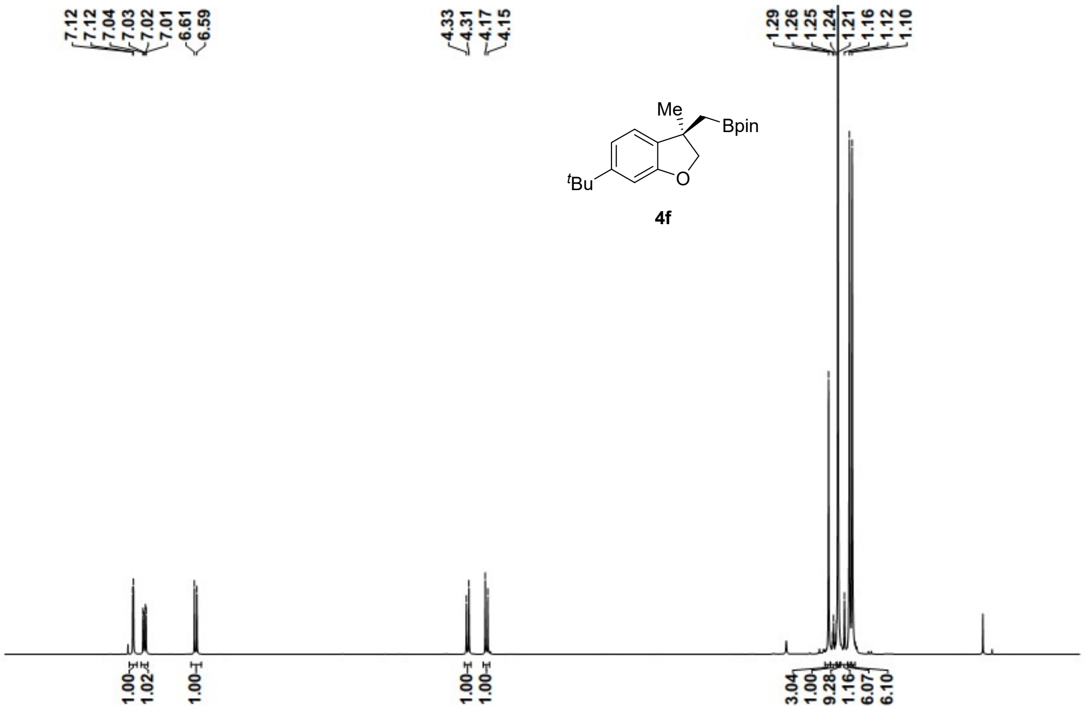

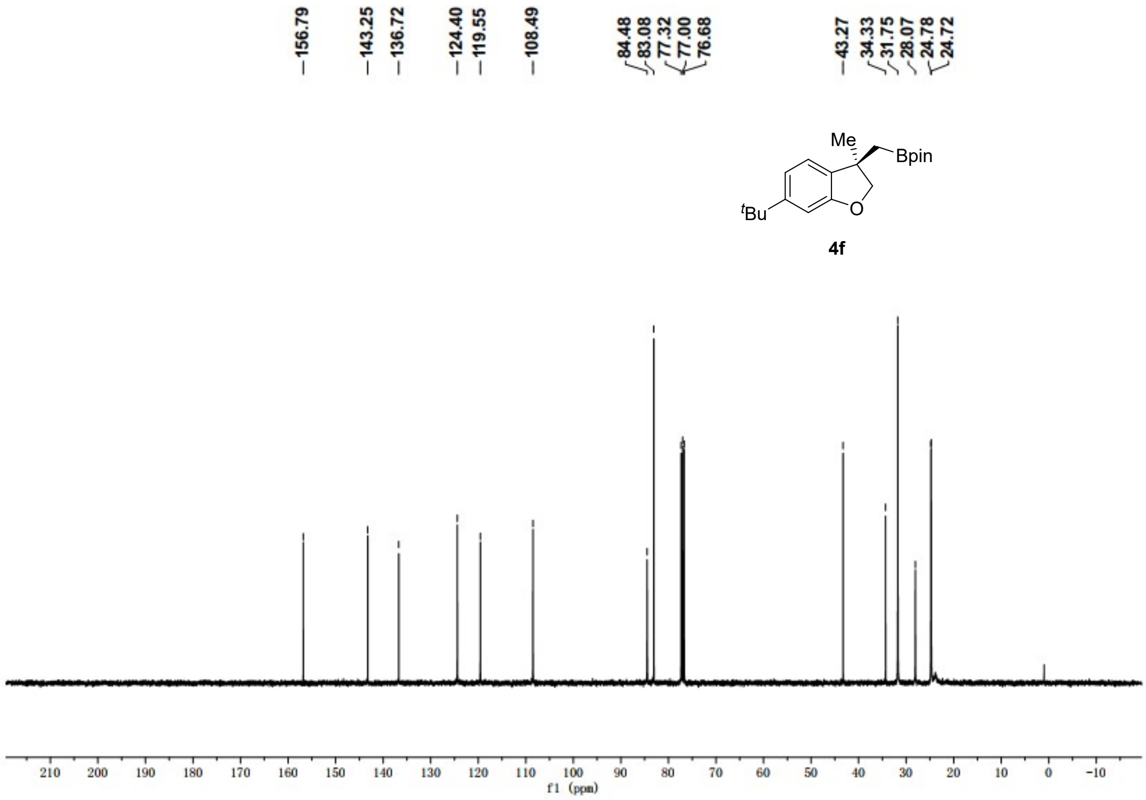


**Chromatogram**

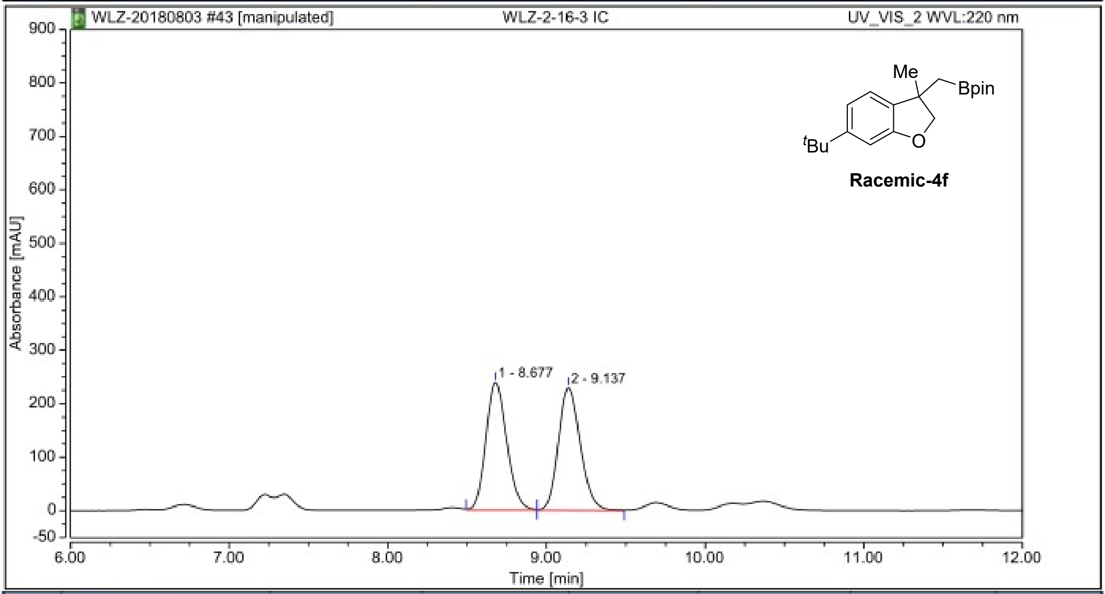


**Integration Results**

| No. | Peak Name | Retention Time min | Area mAU*min | Height mAU | Relative Area % | Relative Height % | Amount n.a. |
|---|---|---|---|---|---|---|---|
| 1 | | 8.677 | 36.770 | 239.580 | 49.88 | 51.09 | n.a. |
| 2 | | 9.137 | 36.945 | 229.392 | 50.12 | 48.91 | n.a. |
| **Total:** | | | **73.715** | **468.972** | **100.00** | **100.00** | |

## Chromatogram

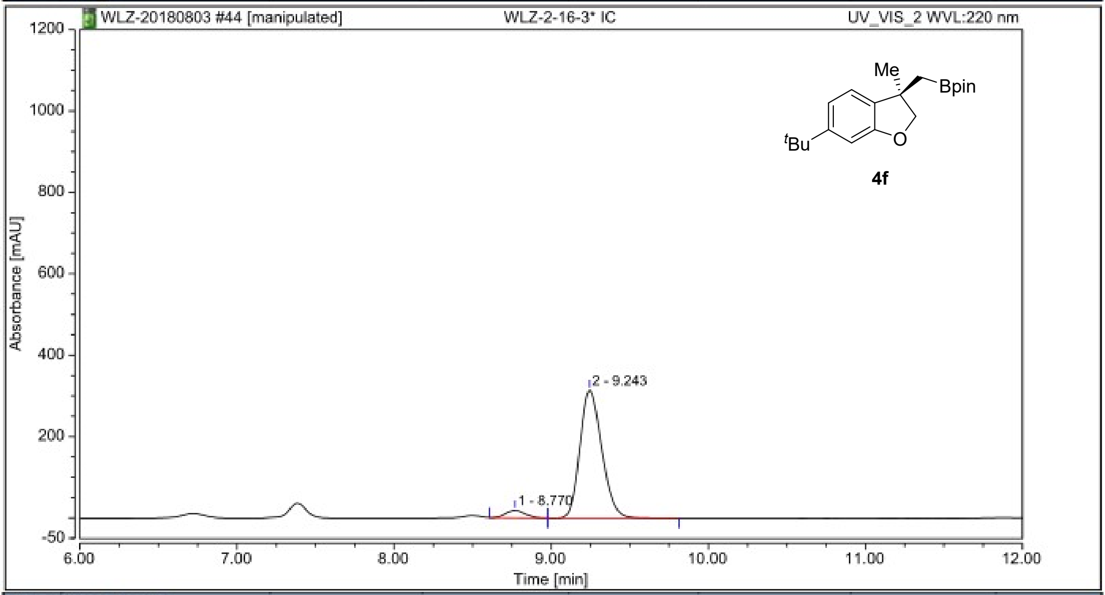


## Integration Results

| No. | Peak Name | Retention Time min | Area mAU*min | Height mAU | Relative Area % | Relative Height % | Amount n.a. |
|---|---|---|---|---|---|---|---|
| 1 | | 8.770 | 2.646 | 18.442 | 4.94 | 5.54 | n.a. |
| 2 | | 9.243 | 50.952 | 314.707 | 95.06 | 94.46 | n.a. |
| **Total:** | | | **53.598** | **333.149** | **100.00** | **100.00** | |

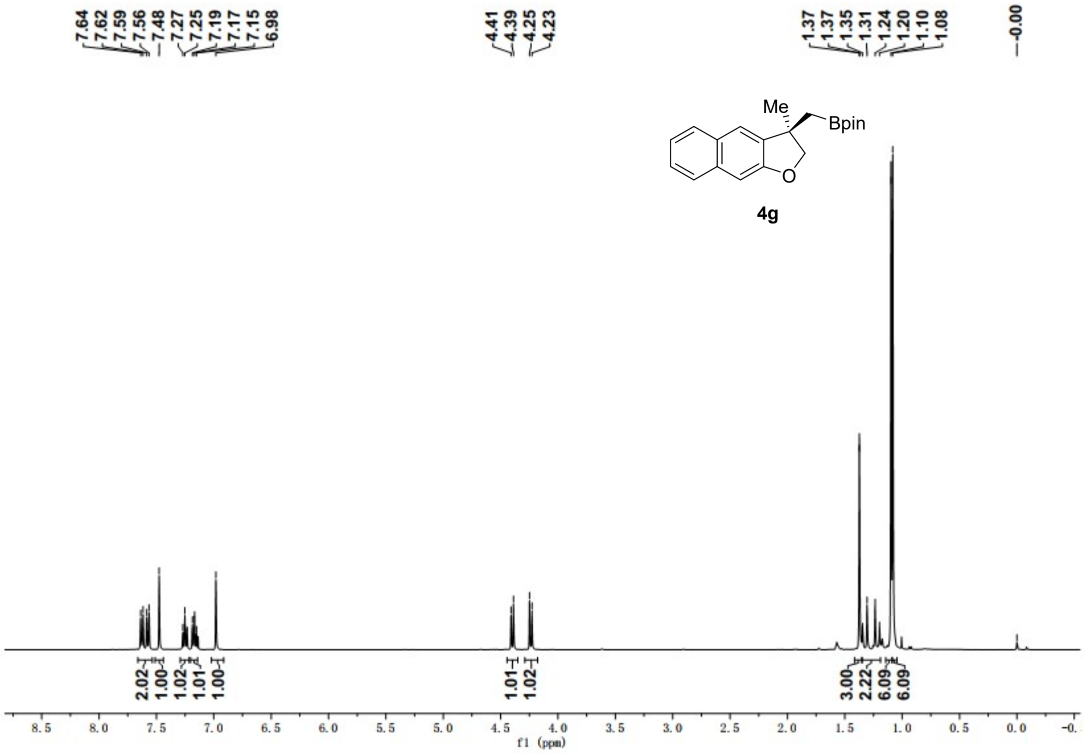

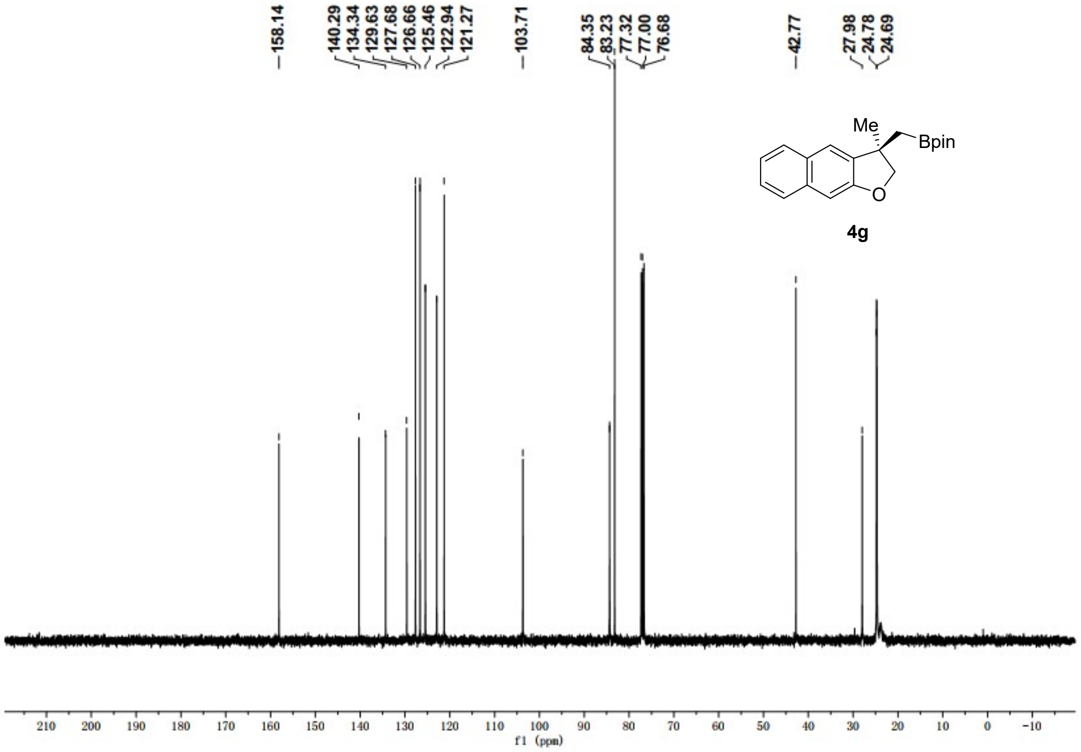


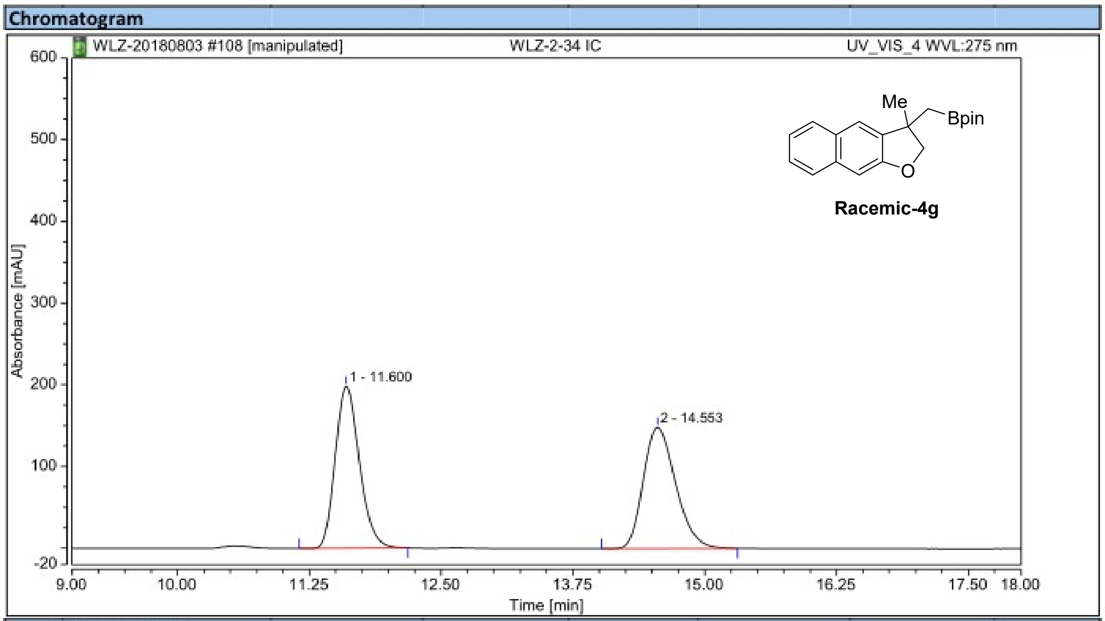


**Integration Results**

| No. | Peak Name | Retention Time min | Area mAU*min | Height mAU | Relative Area % | Relative Height % | Amount n.a. |
|---|---|---|---|---|---|---|---|
| 1 | | 11.600 | 52.713 | 198.166 | 49.91 | 57.18 | n.a. |
| 2 | | 14.553 | 52.908 | 148.375 | 50.09 | 42.82 | n.a. |
| **Total:** | | | **105.621** | **346.541** | **100.00** | **100.00** | |

## Chromatogram

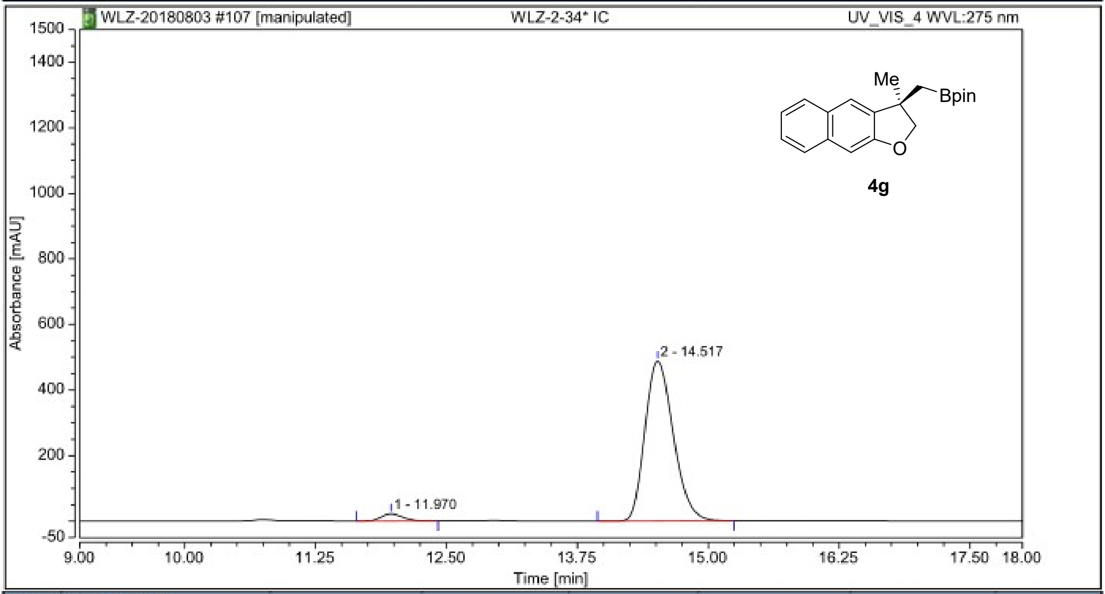


## Integration Results

| No. | Peak Name | Retention Time min | Area mAU*min | Height mAU | Relative Area % | Relative Height % | Amount n.a. |
|---|---|---|---|---|---|---|---|
| 1 | | 11.970 | 5.427 | 22.232 | 3.41 | 4.36 | n.a. |
| 2 | | 14.517 | 153.737 | 487.964 | 96.59 | 95.64 | n.a. |
| **Total:** | | | **159.164** | **510.196** | **100.00** | **100.00** | |

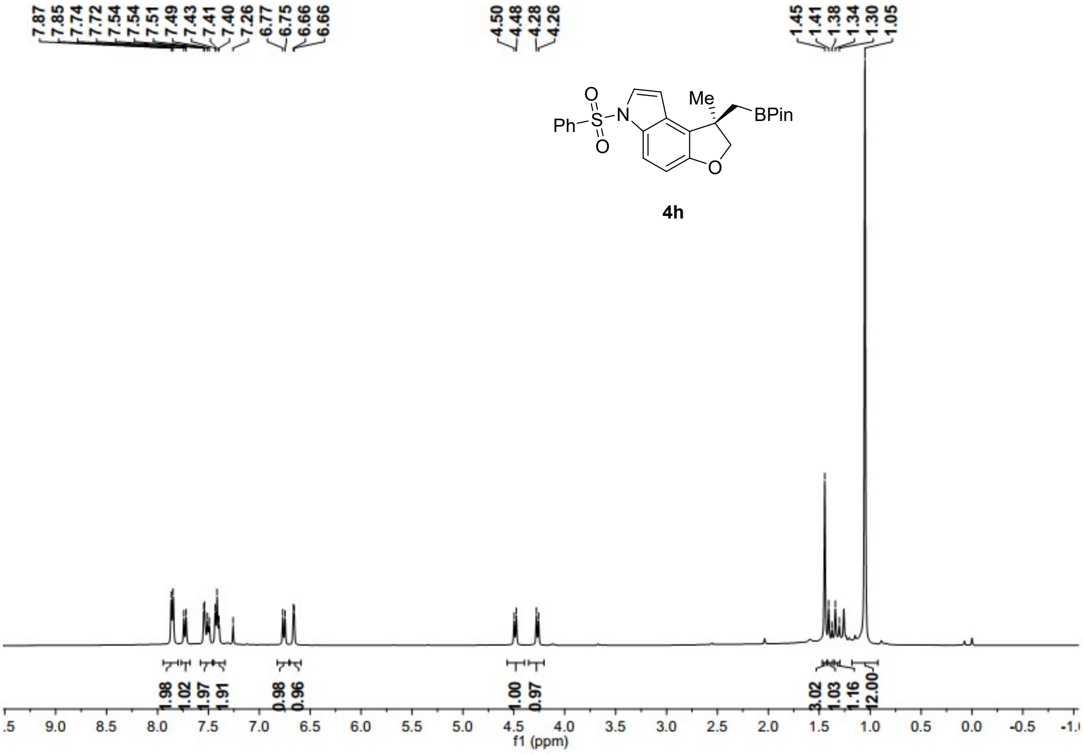

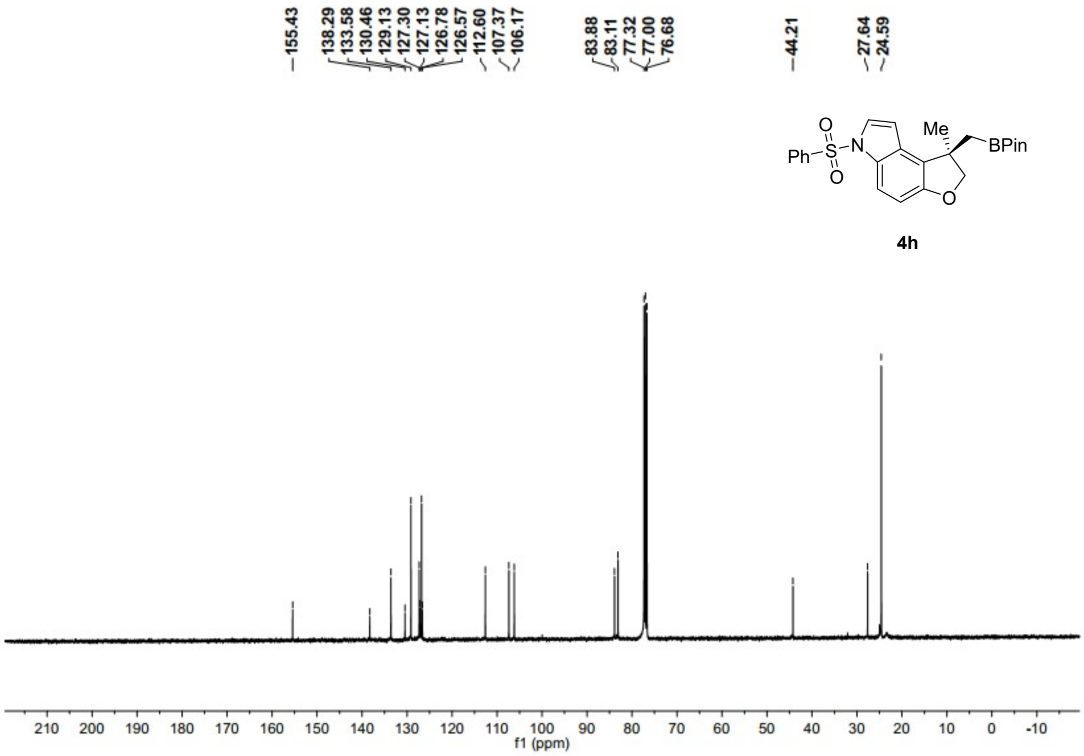


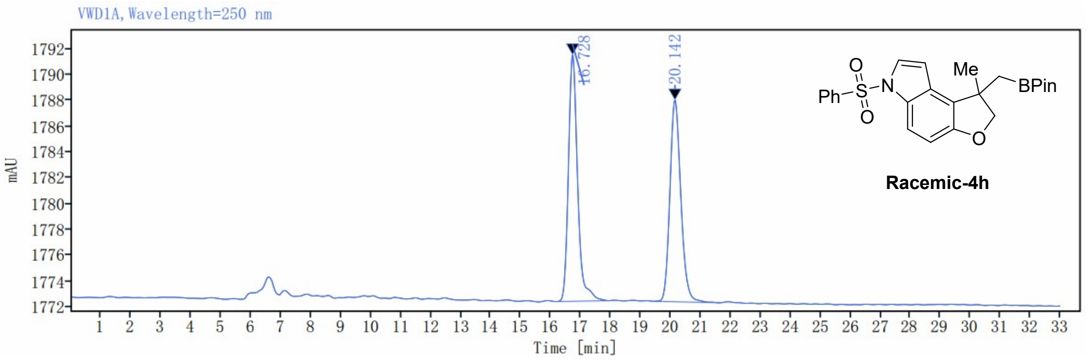


VWD1A, Wavelength=250 nm

| Ret. Time [min] | Area | Height | Height% | Area% |
|---|---|---|---|---|
| 16.728 | 407.03 | 19.09 | 55.00 | 50.67 |
| 20.142 | 396.21 | 15.62 | 45.00 | 49.33 |
| Total. | 803.24 | 34.71 | 100.00 | 100.00 |

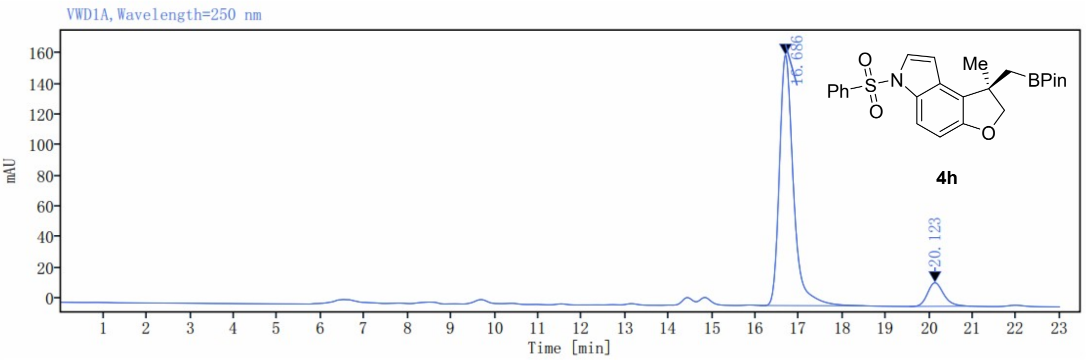


VWD1A, Wavelength=250 nm

| Ret. Time [min] | Area | Height | Height% | Area% |
|---|---|---|---|---|
| 16.686 | 3669.47 | 163.65 | 91.38 | 90.17 |
| 20.123 | 400.01 | 15.43 | 8.62 | 9.83 |
| Total. | 4069.48 | 179.08 | 100.00 | 100.00 |

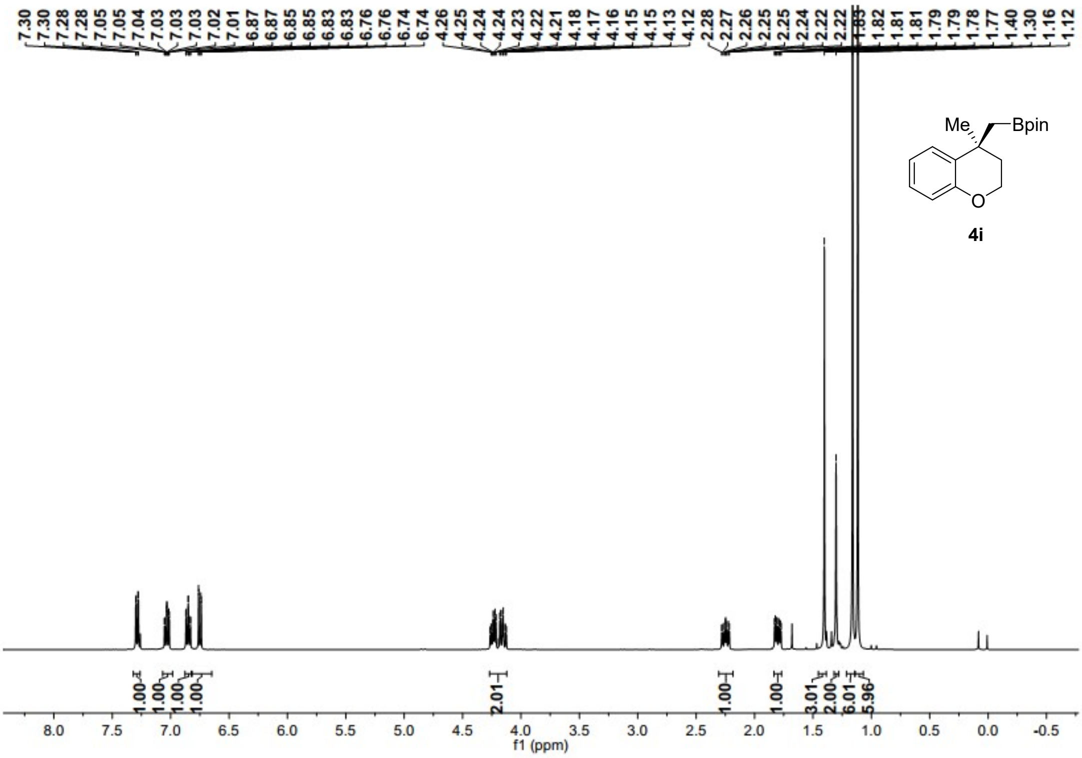

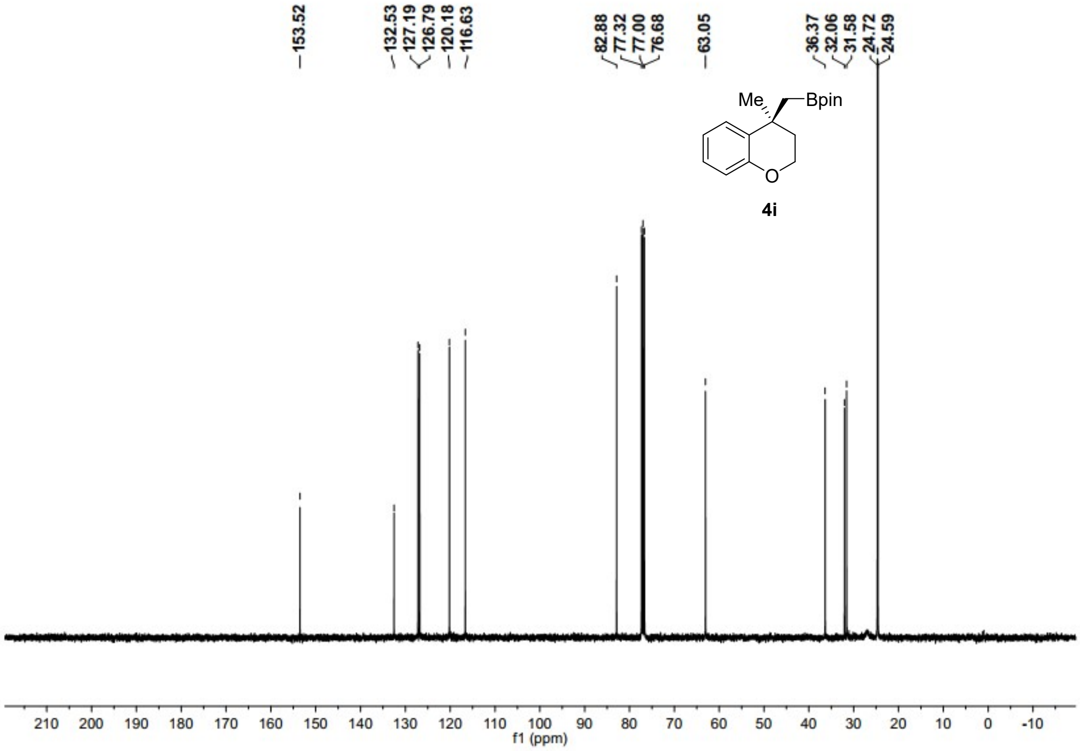


## <Chromatogram>

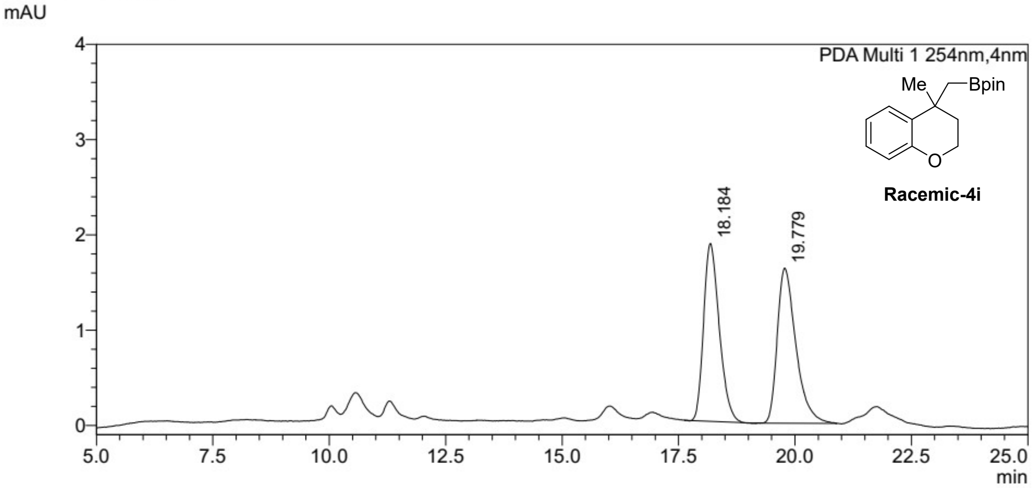


## <Peak Table>

PDA Ch1 254nm

| Peak# | Ret. Time | Area | Area% | Height | Height% |
|---|---|---|---|---|---|
| 1 | 18.184 | 43596 | 49.008 | 1868 | 53.413 |
| 2 | 19.779 | 45362 | 50.992 | 1629 | 46.587 |
| Total | | 88958 | 100.000 | 3497 | 100.000 |

<Chromatogram>

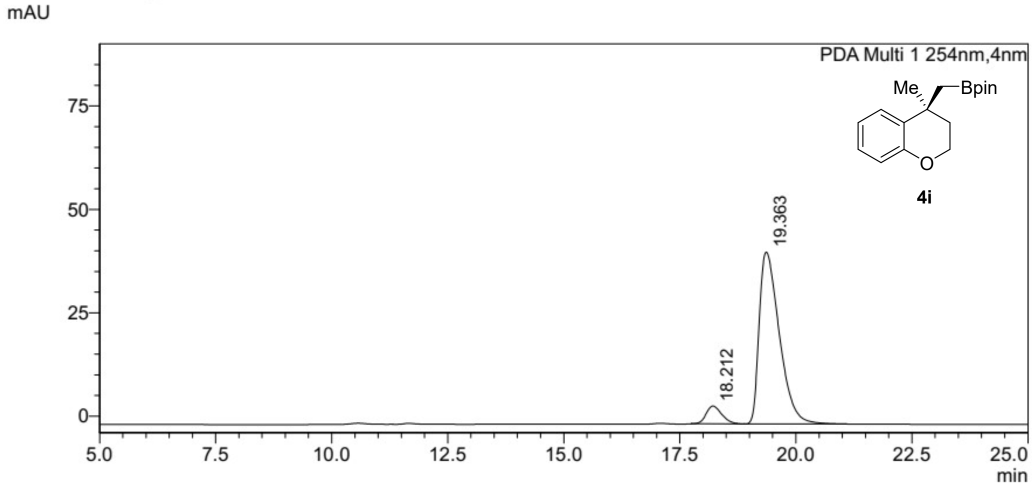


<Peak Table>

PDA Ch1 254nm

| Peak# | Ret. Time | Area | Area% | Height | Height% |
|---|---|---|---|---|---|
| 1 | 18.212 | 101465 | 7.464 | 4314 | 9.405 |
| 2 | 19.363 | 1257906 | 92.536 | 41560 | 90.595 |
| Total | | 1359371 | 100.000 | 45874 | 100.000 |

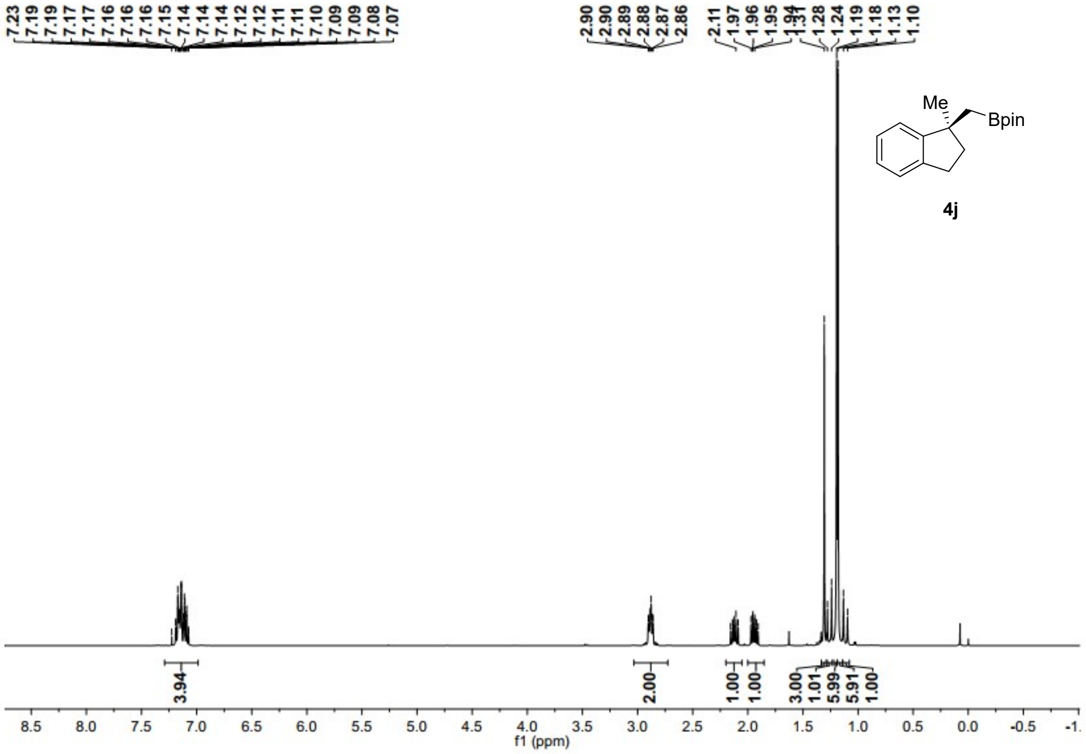

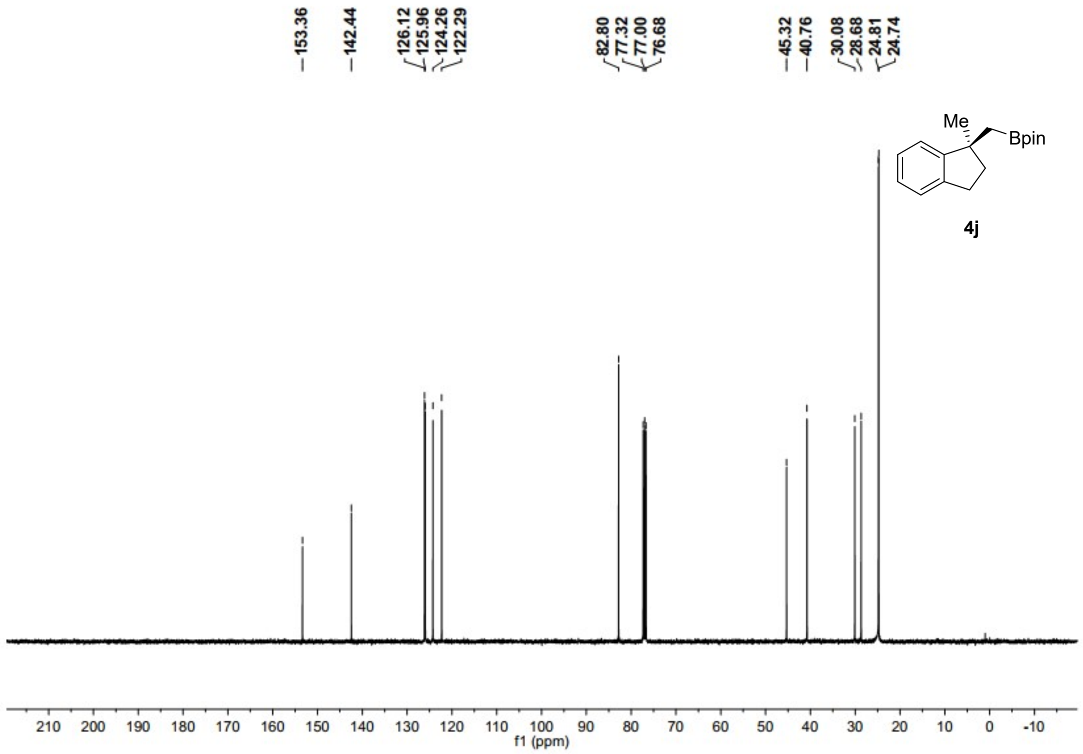


## <Chromatogram>

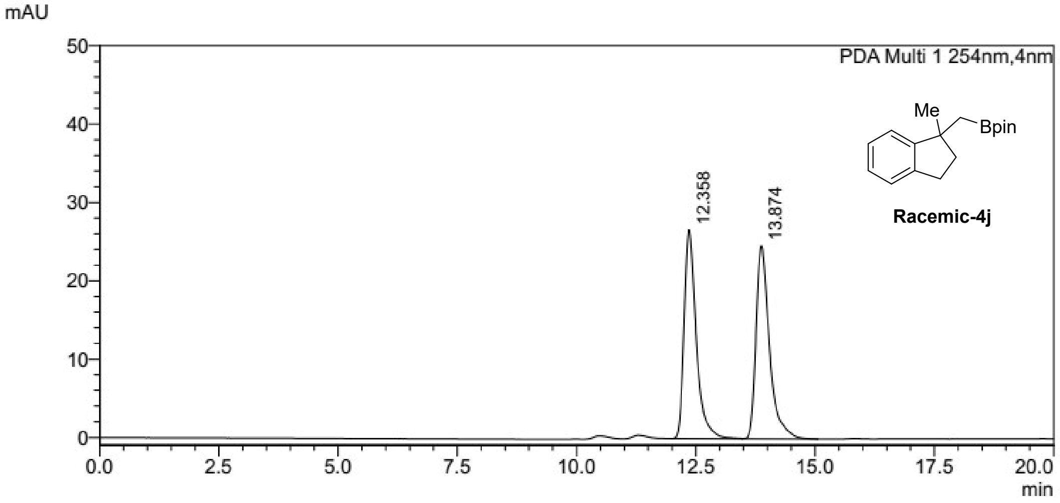


## <Peak Table>

PDA Ch1 254nm

| Peak# | Ret. Time | Area | Area% | Height | Height% |
|---|---|---|---|---|---|
| 1 | 12.358 | 474361 | 49.449 | 26692 | 51.991 |
| 2 | 13.874 | 484928 | 50.551 | 24648 | 48.009 |
| Total | | 959289 | 100.000 | 51340 | 100.000 |

## <Chromatogram>

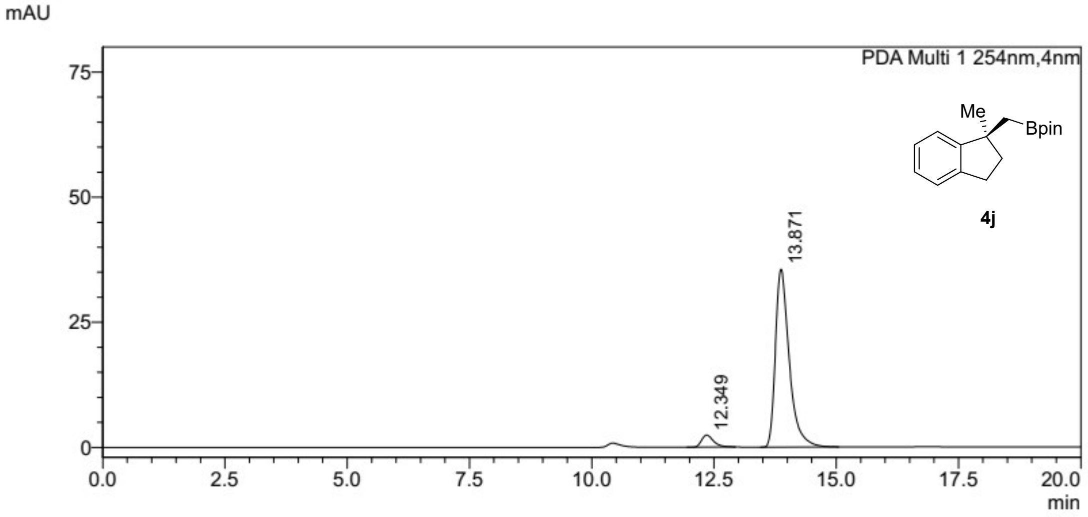


## <Peak Table>

PDA Ch1 254nm

| Peak# | Ret. Time | Area | Area% | Height | Height% |
|---|---|---|---|---|---|
| 1 | 12.349 | 41346 | 5.577 | 2420 | 6.369 |
| 2 | 13.871 | 700075 | 94.423 | 35572 | 93.631 |
| Total | | 741421 | 100.000 | 37992 | 100.000 |

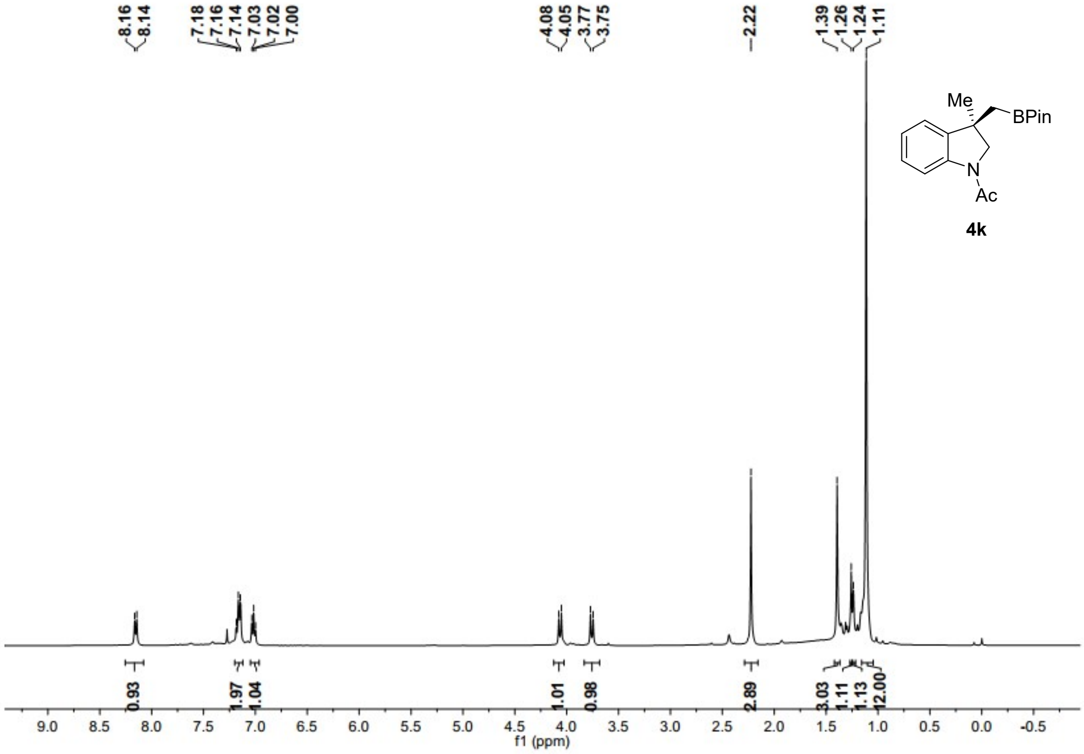

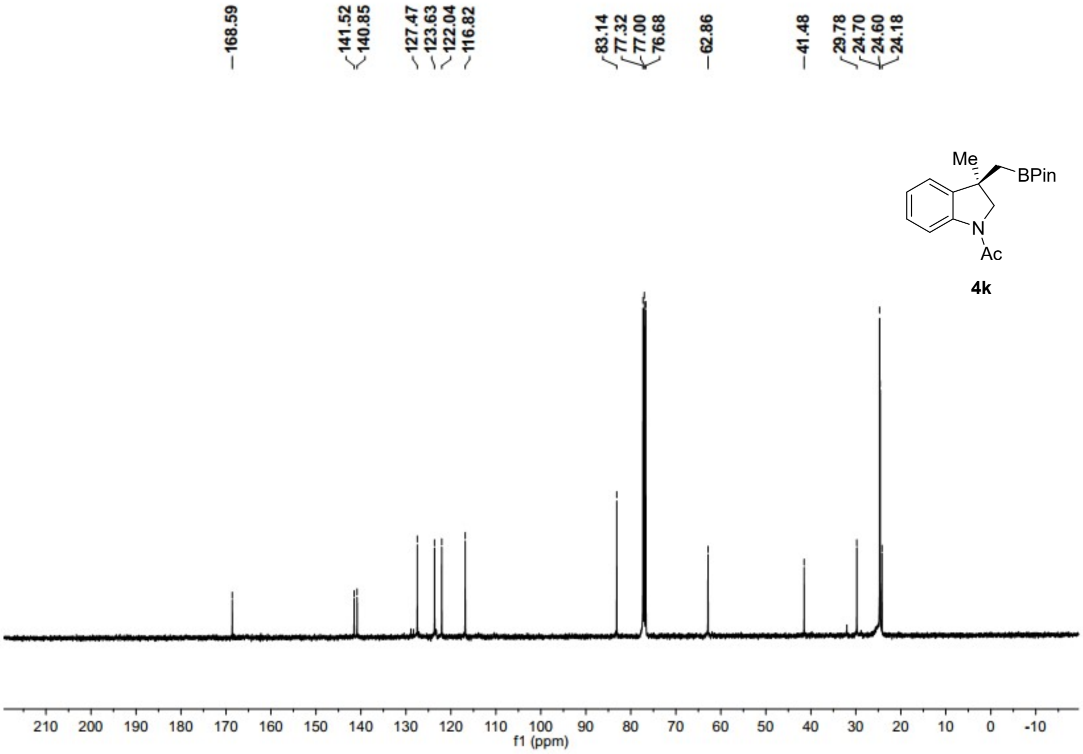


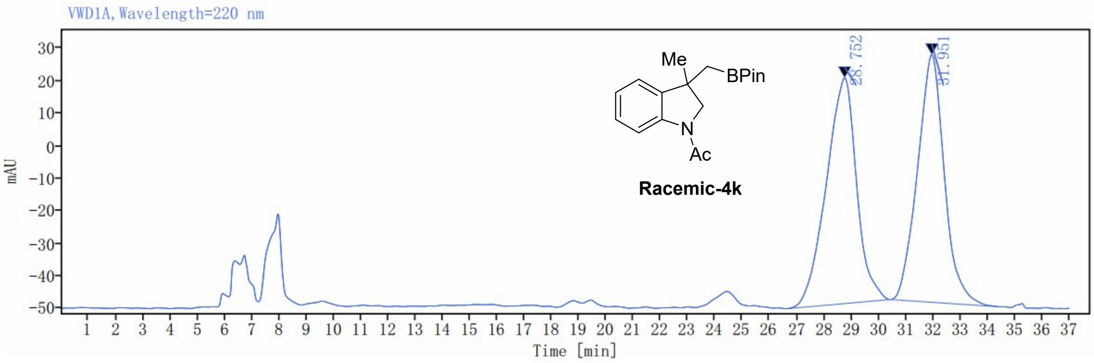


VWD1A, Wavelength=220 nm

| Ret. Time [min] | Area | Height | Height% | Area% |
|---|---|---|---|---|
| 28.752 | 5373.05 | 69.33 | 47.69 | 50.75 |
| 31.951 | 5213.97 | 76.04 | 52.31 | 49.25 |
| Total. | 10587.02 | 145.38 | 100.00 | 100.00 |

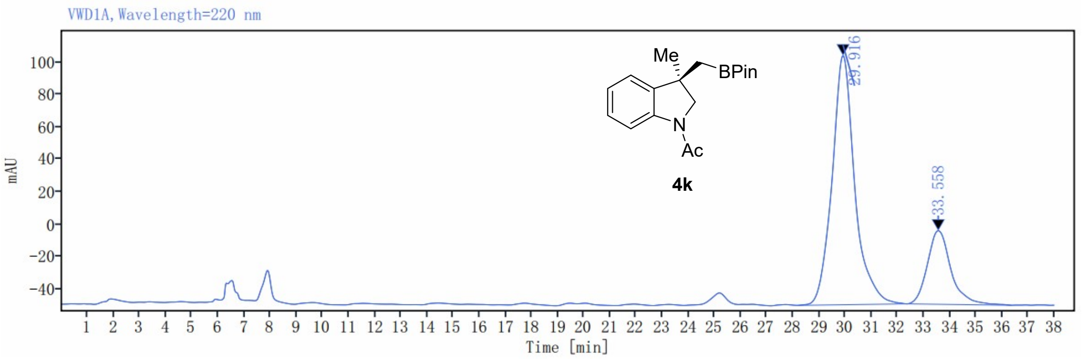


VWD1A, Wavelength=220 nm

| Ret. Time [min] | Area | Height | Height% | Area% |
| --- | --- | --- | --- | --- |
| 29.916 | 9252.23 | 153.44 | 77.21 | 76.49 |
| 33.558 | 2844.20 | 45.30 | 22.79 | 23.51 |
| Total. | 12096.42 | 198.74 | 100.00 | 100.00 |

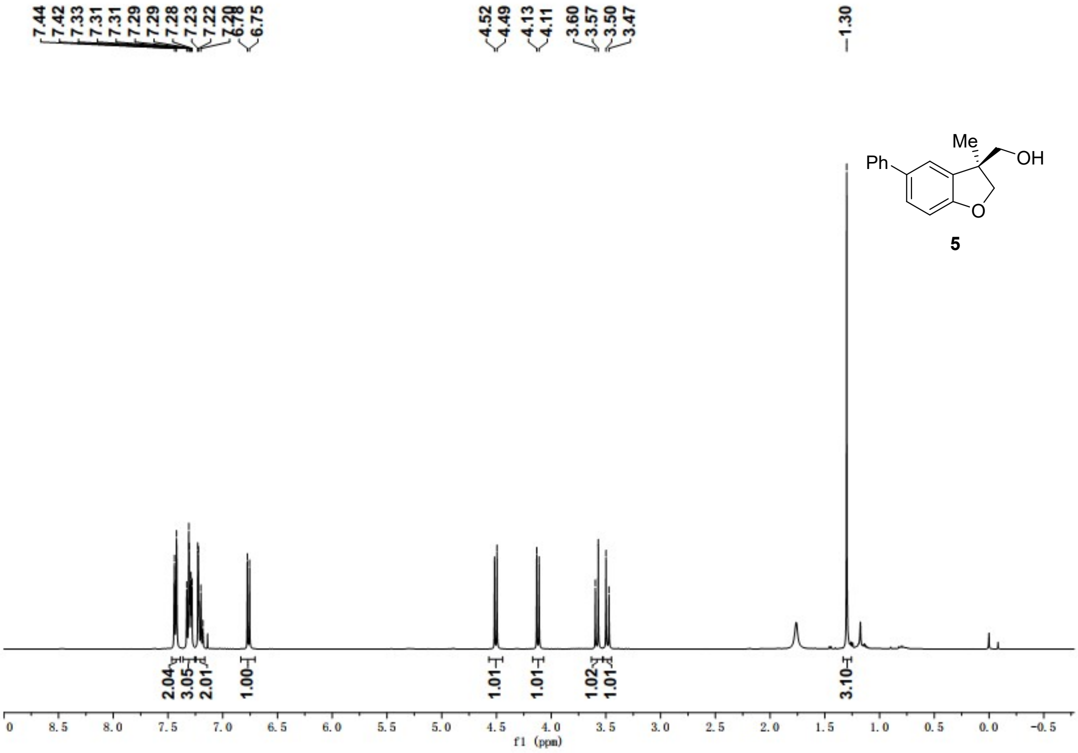

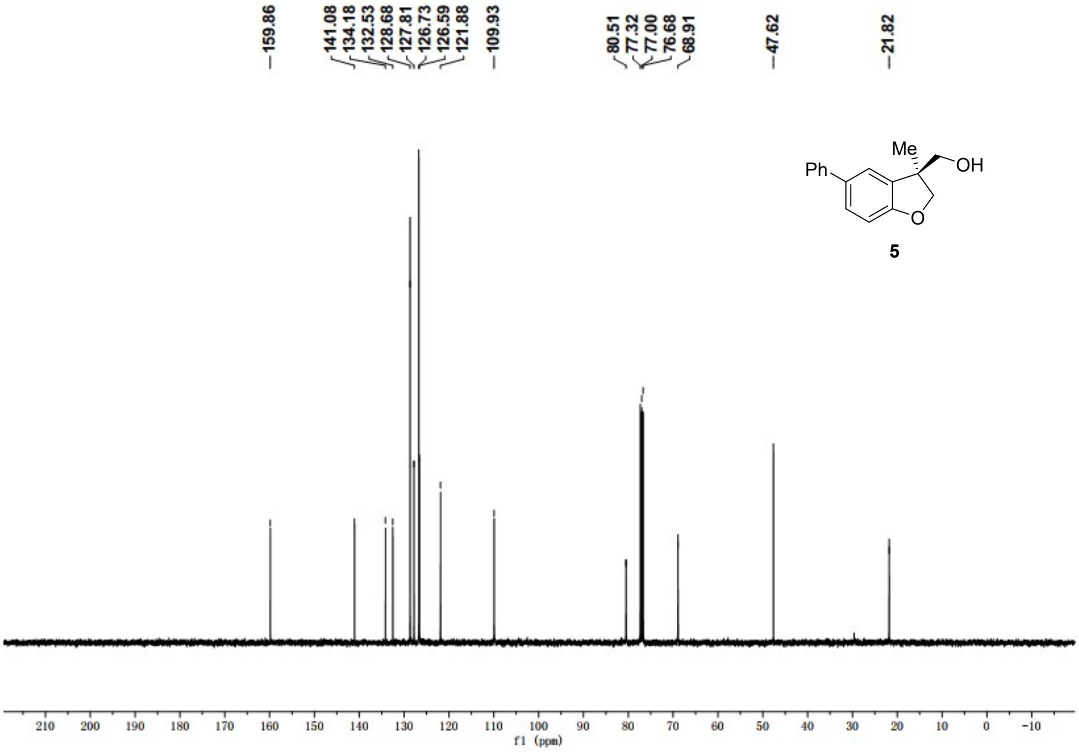


Chromatogram

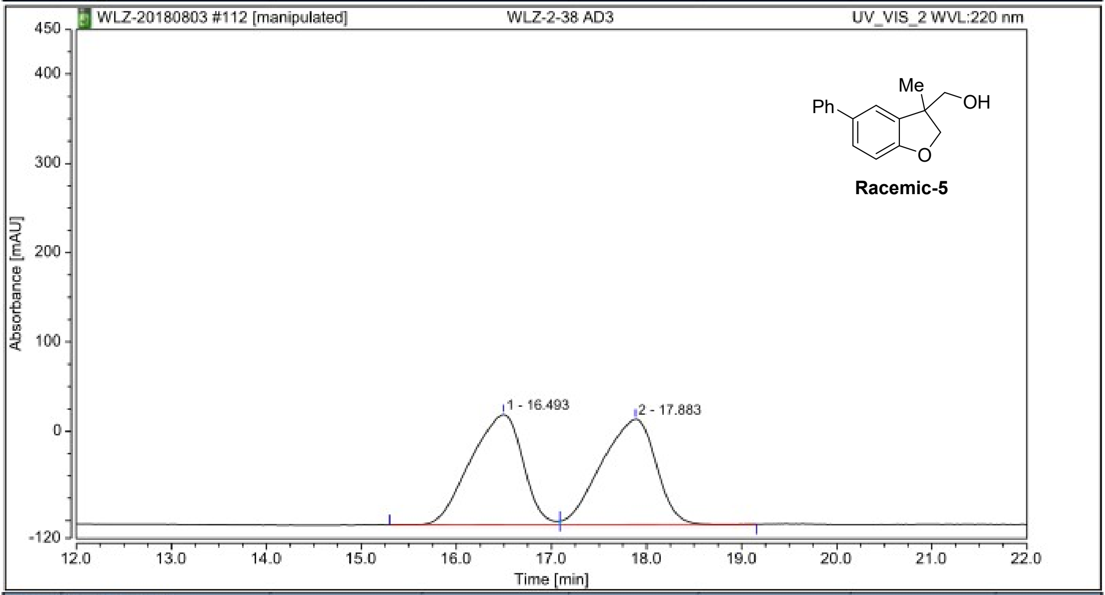


Integration Results

| No. | Peak Name | Retention Time min | Area mAU*min | Height mAU | Relative Area % | Relative Height % | Amount n.a. |
|---|---|---|---|---|---|---|---|
| 1 | | 16.493 | 78.578 | 123.332 | 50.22 | 51.17 | n.a. |
| 2 | | 17.883 | 77.904 | 117.714 | 49.78 | 48.83 | n.a. |
| **Total:** | | | **156.482** | **241.046** | **100.00** | **100.00** | |

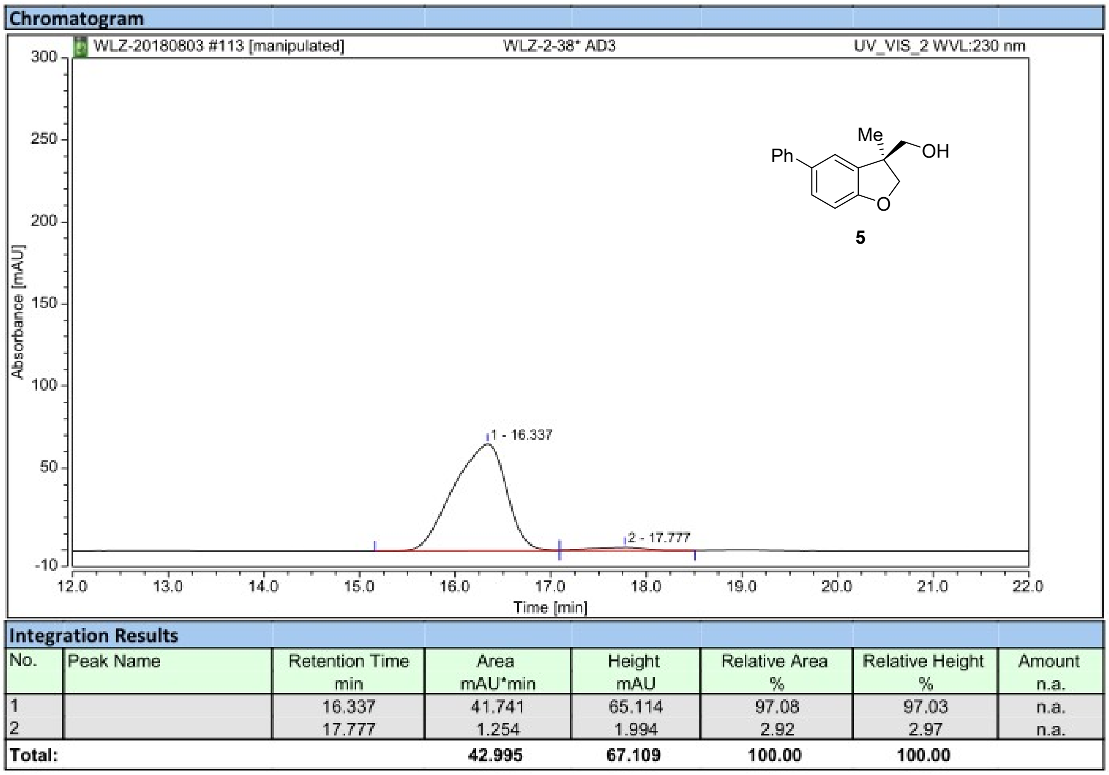


## Integration Results

| No. | Peak Name | Retention Time min | Area mAU*min | Height mAU | Relative Area % | Relative Height % | Amount n.a. |
|---|---|---|---|---|---|---|---|
| 1 | | 16.337 | 41.741 | 65.114 | 97.08 | 97.03 | n.a. |
| 2 | | 17.777 | 1.254 | 1.994 | 2.92 | 2.97 | n.a. |
| Total: | | | 42.995 | 67.109 | 100.00 | 100.00 | |

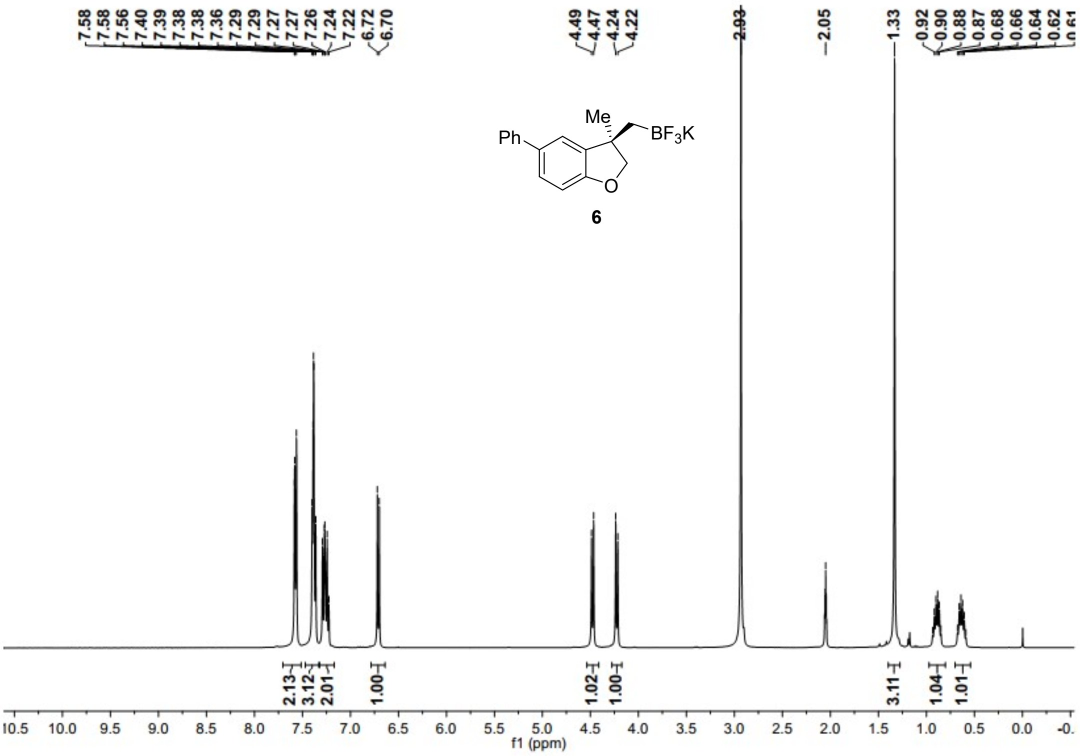

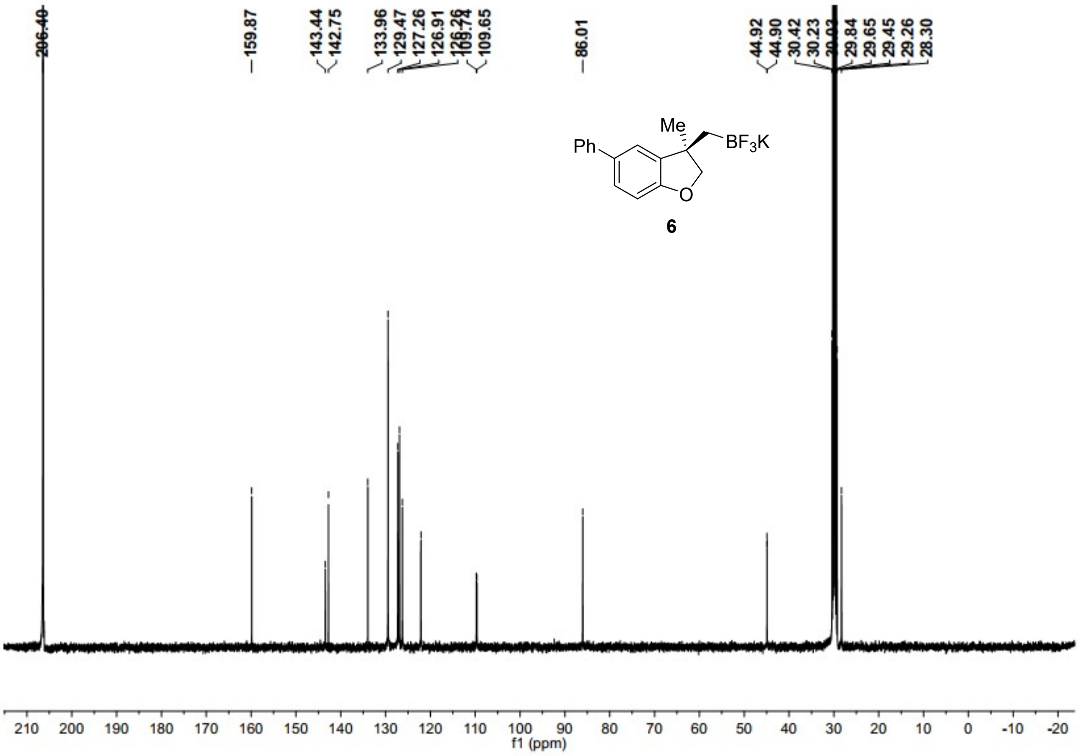
159.87
143.44
142.75
133.96
129.47
127.26
126.91
109.65
86.01
44.92
44.90
30.42
30.23
29.84
29.65
29.45
29.26
28.30
Me
Ph
BF3K
O
6
f1 (ppm)

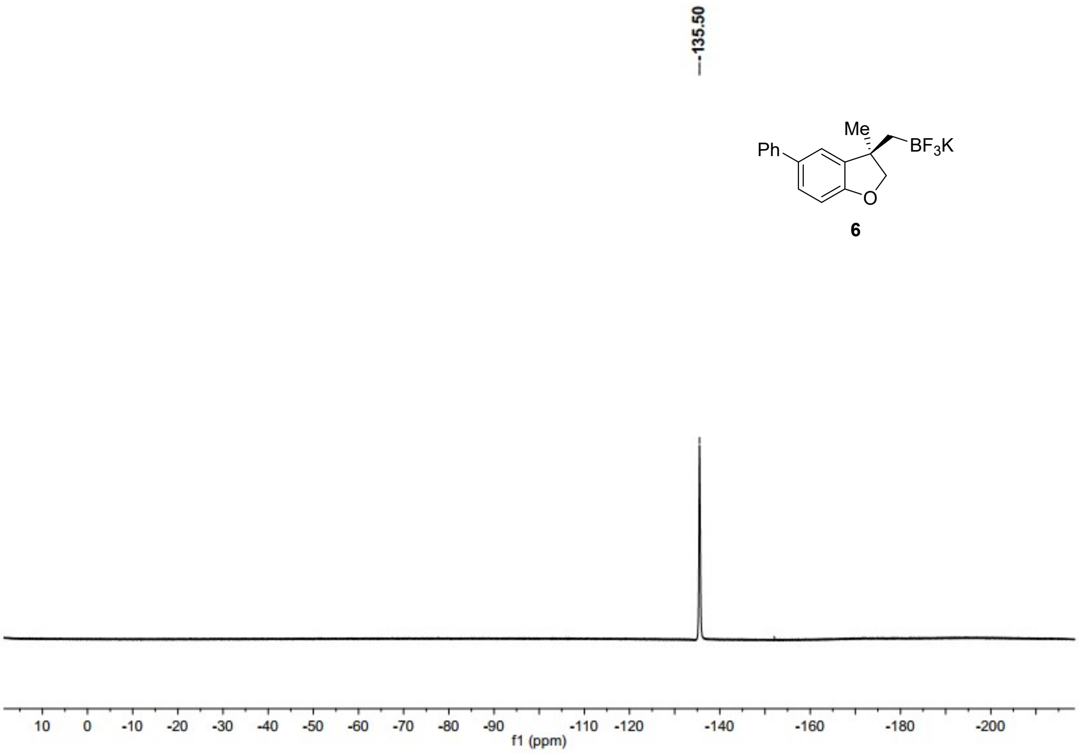
-135.50
Me
Ph
BF3K
O
6
f1 (ppm)

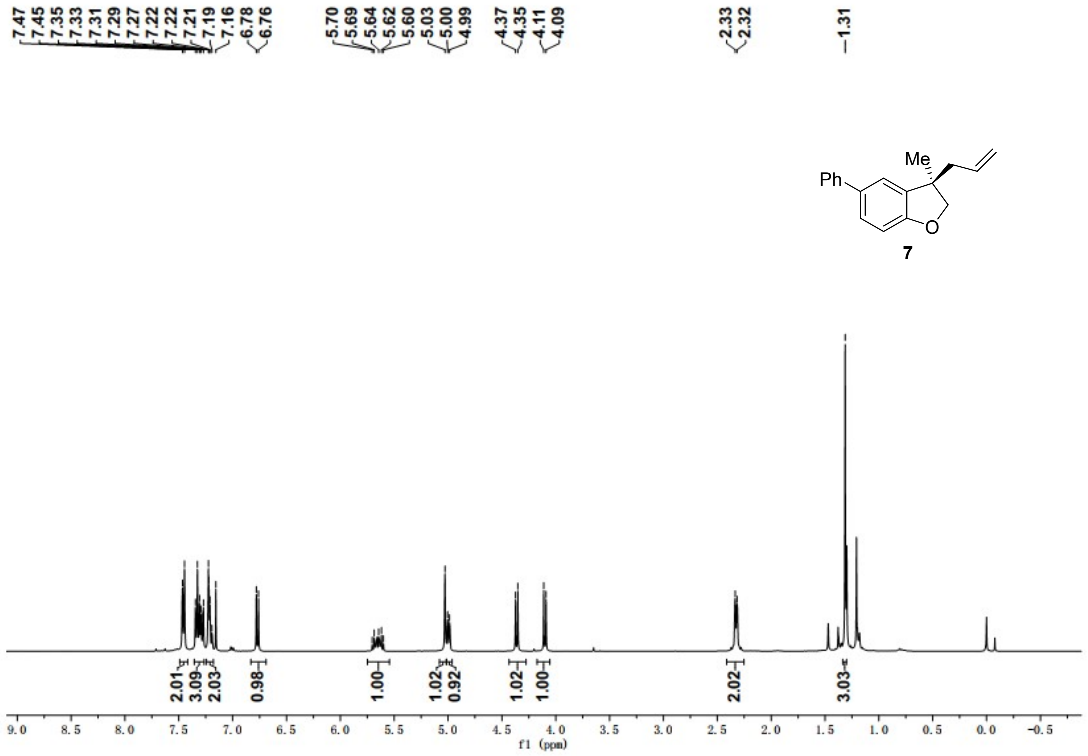
7.47
7.45
7.35
7.33
7.31
7.29
7.27
7.22
7.22
7.21
7.19
7.16
6.78
6.76
5.70
5.69
5.64
5.62
5.60
5.03
5.00
4.99
4.37
4.35
4.11
4.09
2.33
2.32
1.31
Me
Ph
O
7
2.01
3.09
2.03
0.98
1.00
1.02
0.92
1.02
1.00
2.02
3.03
f1 (ppm)

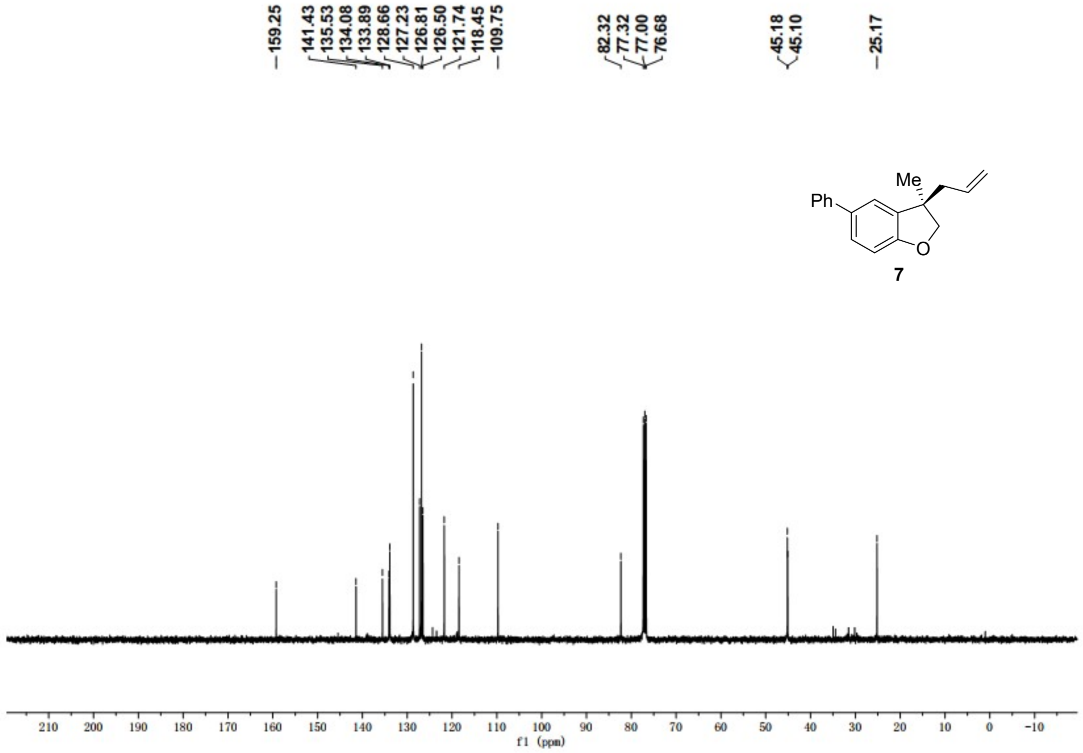
159.25
141.43
135.53
134.08
133.89
128.66
127.23
126.81
126.50
121.74
118.45
109.75
82.32
77.32
77.00
76.68
45.18
45.10
25.17
Me
Ph
O
7
f1 (ppm)

## <Chromatogram>

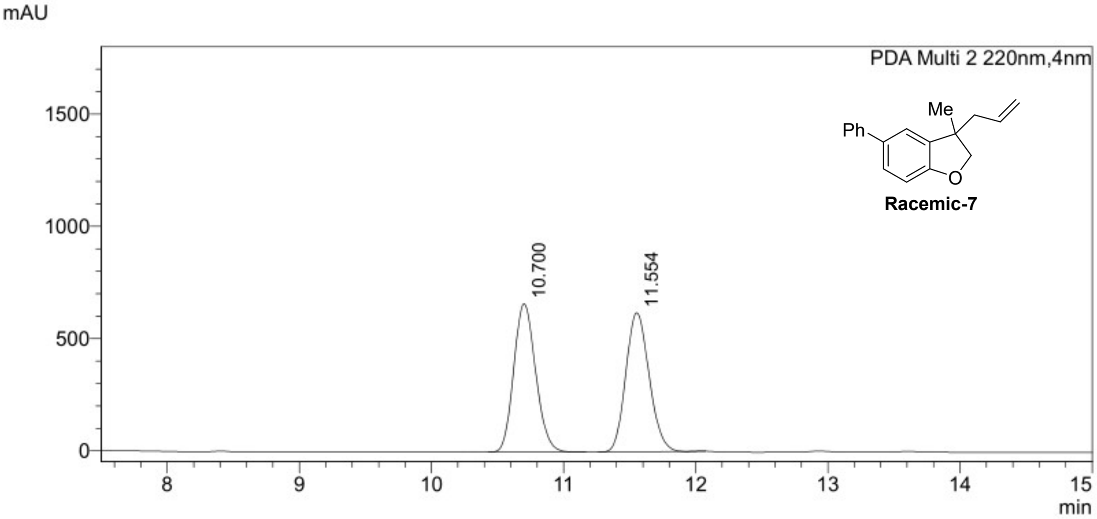


## <Peak Table>

PDA Ch2 220nm

| Peak# | Ret. Time | Area | Area% | Height | Height% |
|---|---|---|---|---|---|
| 1 | 10.700 | 7654150 | 50.047 | 661981 | 51.596 |
| 2 | 11.554 | 7639885 | 49.953 | 621037 | 48.404 |
| Total | | 15294036 | 100.000 | 1283018 | 100.000 |

## <Chromatogram>

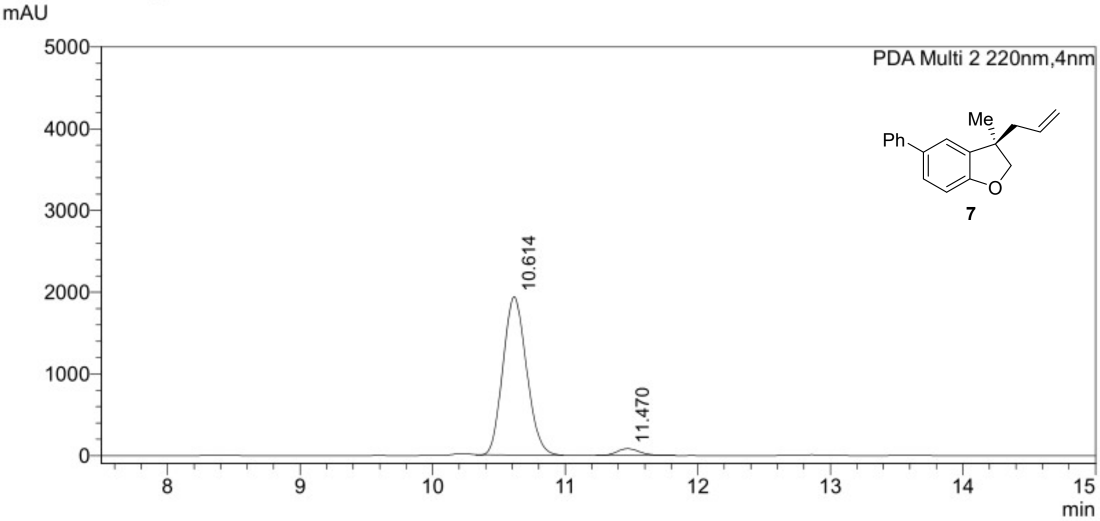


## <Peak Table>

PDA Ch2 220nm

| Peak# | Ret. Time | Area | Area% | Height | Height% |
|---|---|---|---|---|---|
| 1 | 10.614 | 23993482 | 95.883 | 1939038 | 95.804 |
| 2 | 11.470 | 1030228 | 4.117 | 84917 | 4.196 |
| Total | | 25023710 | 100.000 | 2023954 | 100.000 |

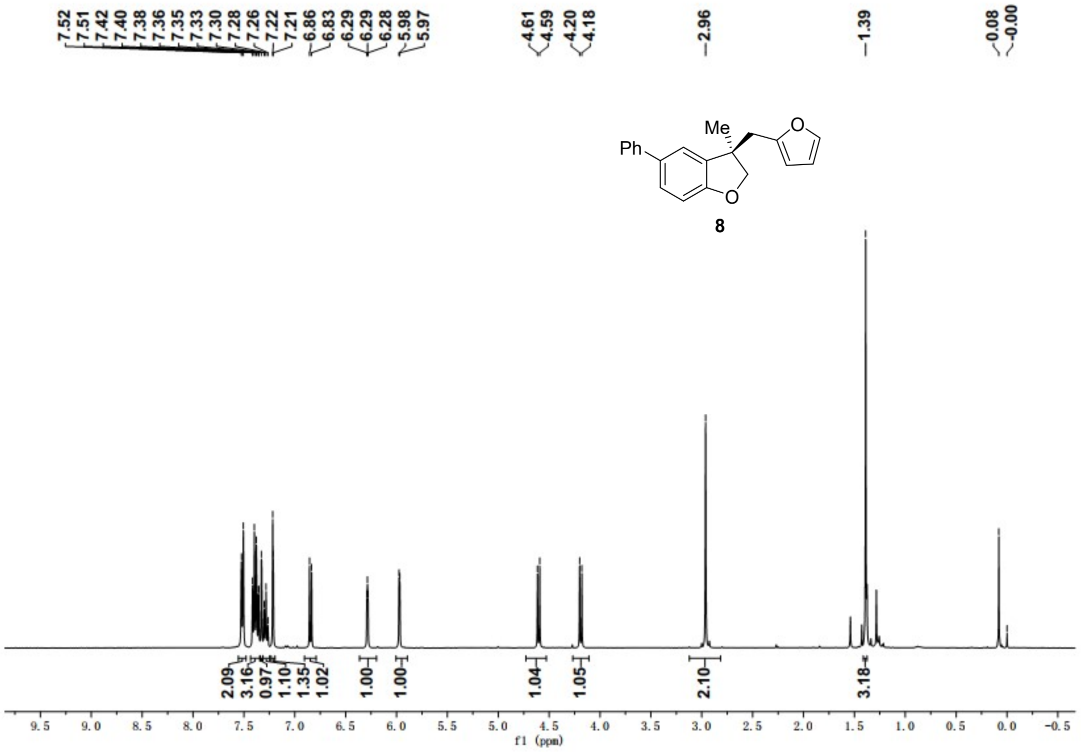

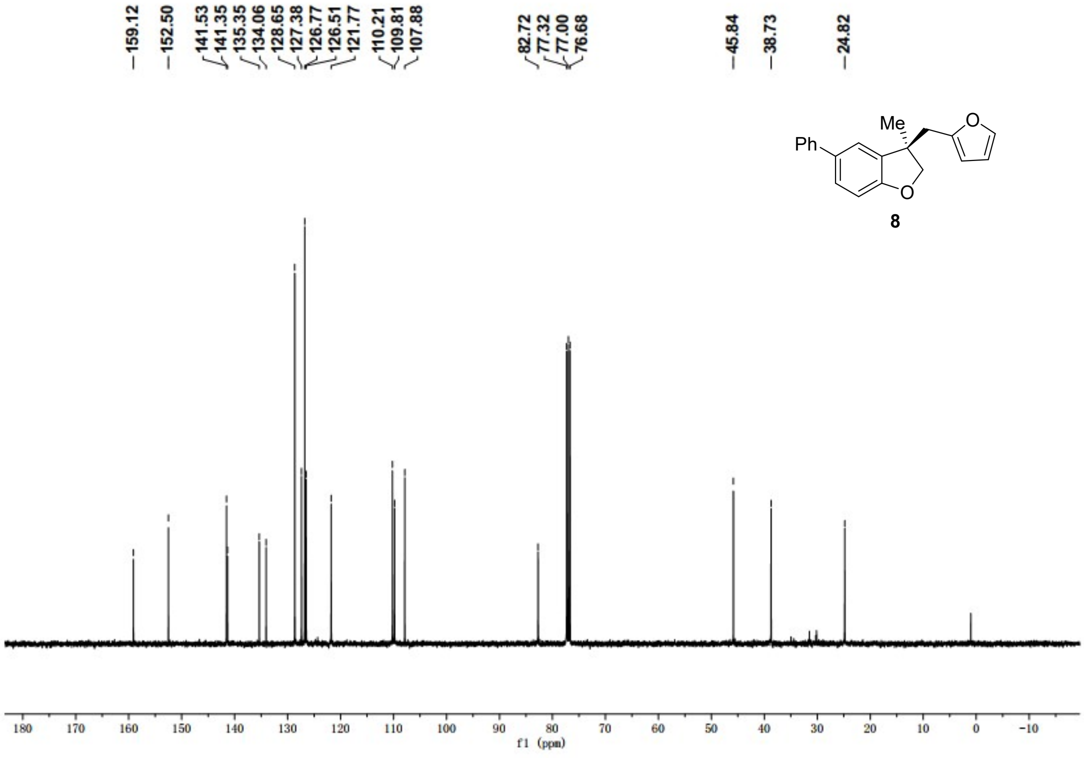

## <Chromatogram>

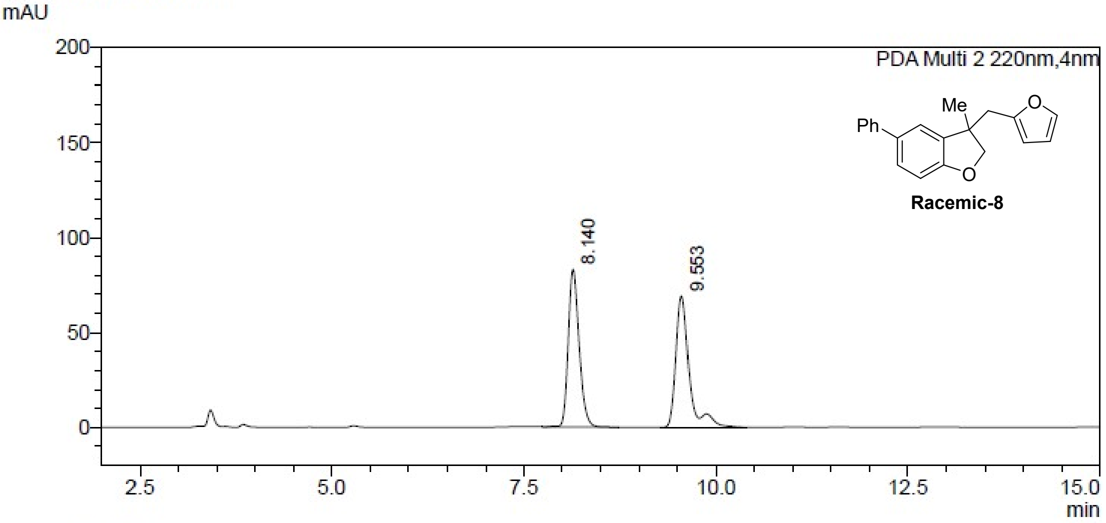


## <Peak Table>

PDA Ch2 220nm

| Peak# | Ret. Time | Area | Area% | Height | Height% |
|---|---|---|---|---|---|
| 1 | 8.140 | 815402 | 49.977 | 83032 | 54.629 |
| 2 | 9.553 | 816138 | 50.023 | 68961 | 45.371 |
| Total | | 1631540 | 100.000 | 151994 | 100.000 |

## <Chromatogram>

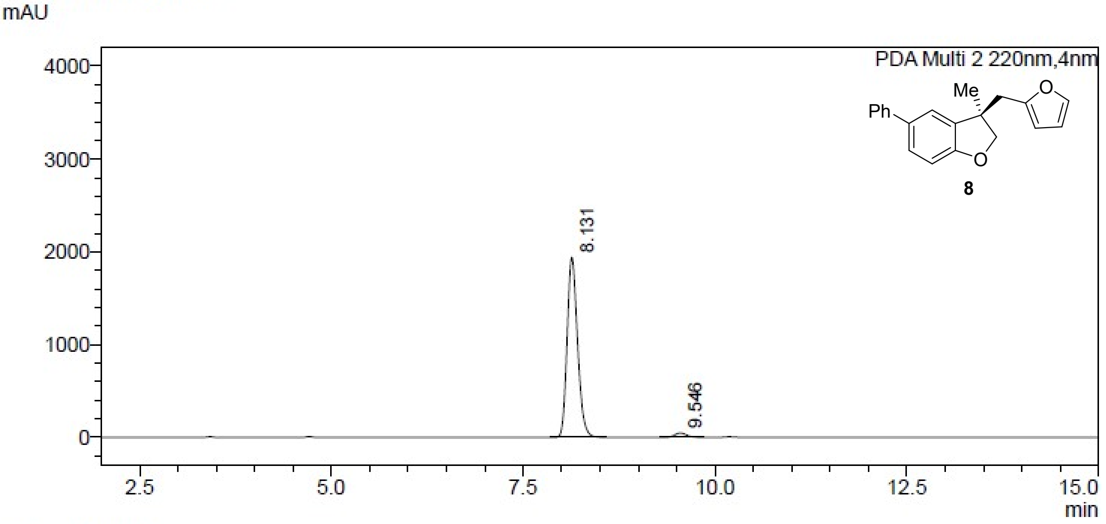


## <Peak Table>

PDA Ch2 220nm

| Peak# | Ret. Time | Area | Area% | Height | Height% |
|---|---|---|---|---|---|
| 1 | 8.131 | 18254957 | 97.318 | 1942884 | 97.611 |
| 2 | 9.546 | 503172 | 2.682 | 47555 | 2.389 |
| Total | | 18758129 | 100.000 | 1990439 | 100.000 |

# 8. References


1. L. Wang, M. Chen, P. Zhang, W. Li and J. Zhang, *J. Am. Chem. Soc.,* 2018, **140**, 3467-3473.
2. W. Zhou, P. Chen, M. Tao, X. Su, Q. Zhao and J. Zhang, *Chem. Commun.,* 2016, **52**, 7612-7615.
3. H. Hu, Y. Wang, D. Qian, Z. Zhang, L. Liu and J. Zhang, *Org. Chem. Front.,* 2016, **3**, 759-763.
4. Z. Zhang, P. Chen, W. Li, Y. Niu, X. Zhao and J. Zhang, *Angew. Chem., Int. Ed.,* 2014, **53**, 4350-4354.
5. Z. Zhang, B. Xu, Y. Qian, L. Wu, Y. Wu, L. Zhou, Y. Liu and J. Zhang, *Angew. Chem., Int. Ed.,* 2018, **57**, 10373-10377.
6. Z. Zhang, B. Xu, L. Wu, Y. Wu, Y. Qian, L. Zhou, Y. Liu and J. Zhang, *Angew. Chem., Int. Ed.,* 2019, **58**, 14653-14659.
7. O. M. Saavedra, D. Karila, D. Brossard, A. Rojas, D. Dupuis, A. Gohier, C. Mannoury la Cour, M. J. Millan, J. Ortuno and S. Hanessian, *Bioorg. Med. Chem.,* 2017, **25**, 38-52.
8. Y. Feng, S. Yang, S. Zhao, D. Zhang, X. Li, H. Liu, Y. Dong, F. Sun. *Org. Lett*., 2020, **22**, 6734-6738.
9. A. Whyte, K. I. Burton, J. Zhang and M. Lautens, *Angew. Chem., Int. Ed.,* 2018, **57**, 13927-13930.
10. D. D. Vachhani, H. H. Butani, N. Sharma, U. C. Bhoya, A. K. Shah and E. V. Van der Eycken, *Chem. Commun*., 2015, **51**, 14862-14865.
11. W. Wang, C. Ding and G. Yin, *Nat. Catal.,* 2020, **3**, 951-958.
